# Supplementary material for: Allosteric activation of the SPRTN protease by ubiquitin maintains genome stability
Source: Nat Commun. 2025 Jul 21;16:5422. doi: 10.1038/s41467-025-61224-z (PMC12279946; doi:10.1038/s41467-025-61224-z)
Supplement: Supplementary file 1 — Supplementary Information [file 41467_2025_61224_MOESM1_ESM.pdf]

Supplementary Information

Allosteric activation of the SPRTN protease by ubiquitin maintains genome stability

Sophie Dürauer[1,2], Hyun-Seo Kang[3,4], Christian Wiebeler[5], Yuka Machida[6], Dina S Schnapka[1,2], Denitsa Yaneva[1,2], Christian Renz[7], Maximilian J Götz[1,2], Pedro Weickert[1,2], Abigail C Major[5], Aldwin S Rahmanto[7,8], Sophie M Gutenthaler-Tietze[9,10], Lena J Daumann[9], Petra Beli[7,8], Helle D Ulrich[7], Michael Sattler[3,4], Yuichi J Machida[6], Nadine Schwierz[5] and Julian Stingele[1,2,*]

[1] Gene Center, Ludwig-Maximilians-Universität München, Munich, Germany
[2] Department of Biochemistry, Ludwig-Maximilians-Universität München, Munich, Germany
[3] Institute of Structural Biology, Molecular Targets and Therapeutics Center, Helmholtz Munich, Neuherberg, Germany
[4] Bavarian NMR Center and Department of Bioscience, TUM School of Natural Sciences, Technical University of Munich, Garching, Germany
[5] Institute of Physics, University of Augsburg, Augsburg, Germany
[6] Developmental Therapeutics Branch, Center for Cancer Research, National Cancer Institute, Bethesda, MD, USA
[7] Institute of Molecular Biology gGmbH, Mainz, Germany
[8] Institute of Developmental Biology and Neurobiology (IDN), Johannes Gutenberg-Universität Mainz, Germany
[9] Chair of Bioinorganic Chemistry, Heinrich-Heine Universität Düsseldorf, Düsseldorf, Germany
[10] Department of Chemistry, Ludwig-Maximilians-Universität München, Munich, Germany
* Correspondence: stingele@genzentrum.lmu.de

SUPPLEMENTARY FIGURE 1

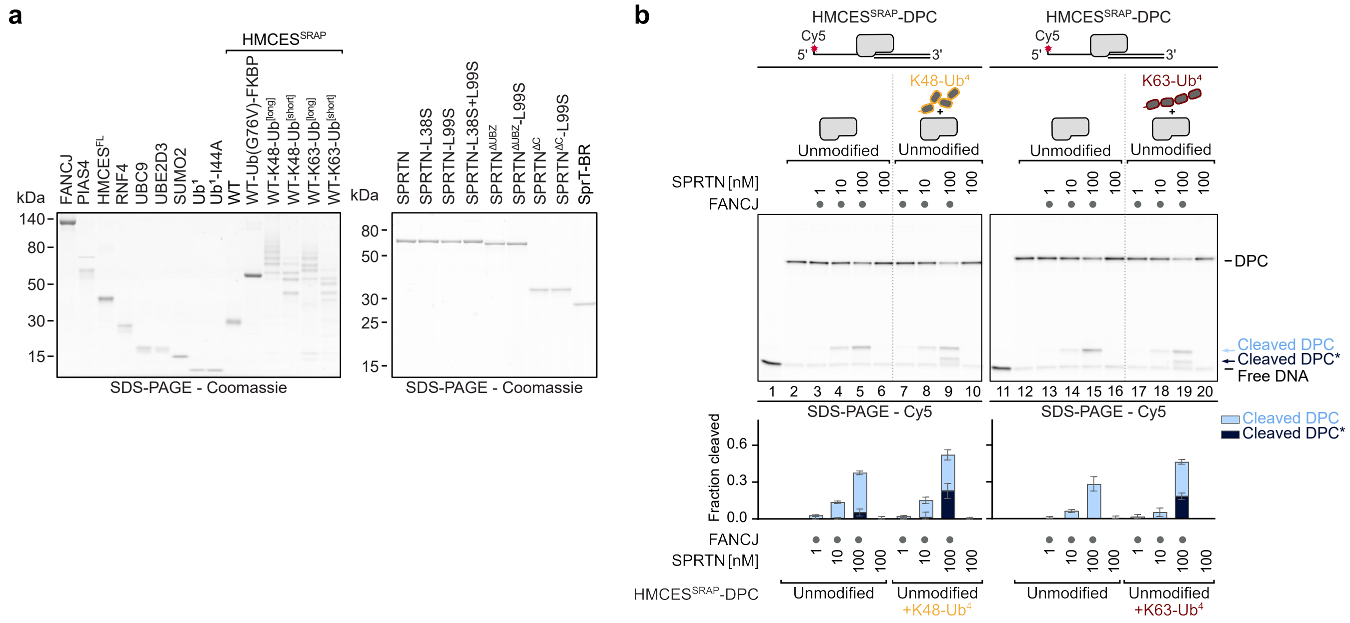

Supplementary Fig. 1. Ubiquitin boosts DPC cleavage by SPRTN

(a) Coomassie stained SDS-PAGE gel, showing equimolar amounts of purified recombinant human FANCJ, PIAS4, HMCES^{FL}, RNF4, UBC9, UBE2D3, SUMO2, mono-ubiquitin (Ub^{1}) and Ub^{1}-I44A, HMCES^{SRAP}, HMCES^{SRAP}-Ub(G76V)-FKBP, HMCES^{SRAP}-K48-Ub$^{[long]}$, HMCES^{SRAP}-K48-Ub$^{[short]}$, HMCES^{SRAP}-K63-Ub$^{[long]}$ and HMCES^{SRAP}-K63-Ub$^{[short]}$, SPRTN, SPRTN-L38S, SPRTN-L99S, SPRTN-L38S+L99S, SPRTN$^{\Delta UBZ}$, SPRTN$^{\Delta UBZ}$-L99S, SPRTN$^{\Delta C}$, SPRTN$^{\Delta C}$-L99S, and SprT-BR used for *in vitro* assays. Source data are provided as a Source Data file.

(b) HMCES^{SRAP}-DPCs (10 nM) were incubated alone or in the presence of FANCJ (100 nM), K48-tetra-ubiquitin (Ub^{4}) or K63-Ub^{4} (400 nM, referring to the concentration of individual ubiquitin moieties) and indicated concentrations of SPRTN (1-100 nM) for 1 h at 30°C. Quantification: bar graphs represent the mean ± SD of three independent experiments. All samples derive from the same experiment and gels were processed in parallel. Values for cleavage of unmodified HMCES^{SRAP}-DPC are the same as in Fig. 1e. Source data are provided as a Source Data file.

SUPPLEMENTARY FIGURE 2

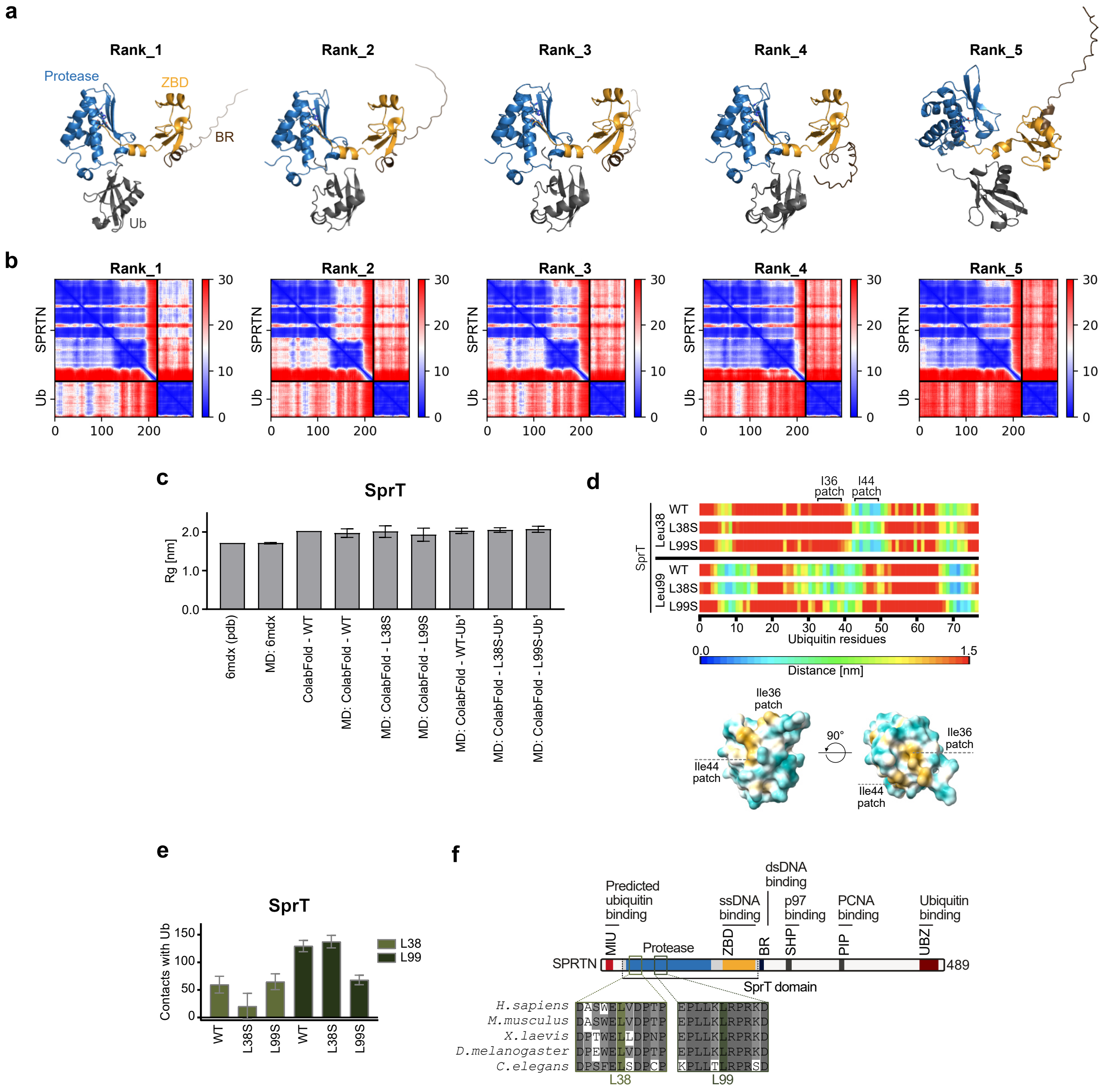

Supplementary Fig. 2. A novel ubiquitin binding interface at the SprT domain

(a) Structures of SprT-BR-ubiquitin complex predicted by ColabFold using AlphaFold2_ptm. All five models (Rank_1-5) are shown (left to right), highlighting SPRTN's protease domain (blue), Zinc-binding domain (ZBD) (orange) and basic region (BR) (brown). Ubiquitin (Ub^1) is shown in grey.

(b) Predicted aligned error (PAE) blots of predicted SprT-BR-Ub^1 complexes shown in (a).

(c) Bar charts showing radius of gyration (Rg) before and after molecular dynamics (MD) simulations for SprT (PDB: 6mdx and ColabFold predicted) and SprT-Ub^1 (ColabFold predicted). In case of MD simulations, the mean ± SD of snapshots from three independent 400 ns MD trajectories is given and the first 100 ns of each trajectory were discarded for equilibration. Source data are provided as a Source Data file.

(d) Heat map indicating minimum distances of all amino acids in ubiquitin to residues at positions 38 (upper part) and 99 (lower part) of SPRTN, including wild-type (WT) conditions (L38, L99) and mutated states (L38S, L99S) (top). Structure of ubiquitin colored by hydrophobicity (bottom). Ile36 and Ile44 patches are highlighted.

(e) Average number of contacts between SprT residues 38 and 99 and ubiquitin, defined as interatomic distance <0.6 nm between side chain or backbone. Contacts for multiple atoms of the SprT residue with an atom of ubiquitin are only counted once. For the WT protein: forms approximately 60 (residue 38) and approximately 130 (residue 99) contacts. In the L38S variant contacts are reduced to approximately 20 at residue 38 and largely unchanged at residue 99. In L99S variant contacts at residue 99 decrease to around 68, while interactions at residue 38 remain similar to the WT. Source data are provided as a Source Data file.

(f) Schematic of SPRTN's domain structure with sequence alignment highlighting key residues in the SprT domain in *H. sapiens*, *M. musculus*, *X. laevis*, *D. melanogaster* and *C. elegans* SPRTN homologues (L38 = light green, L99 = dark green).

SUPPLEMENTARY FIGURE 3

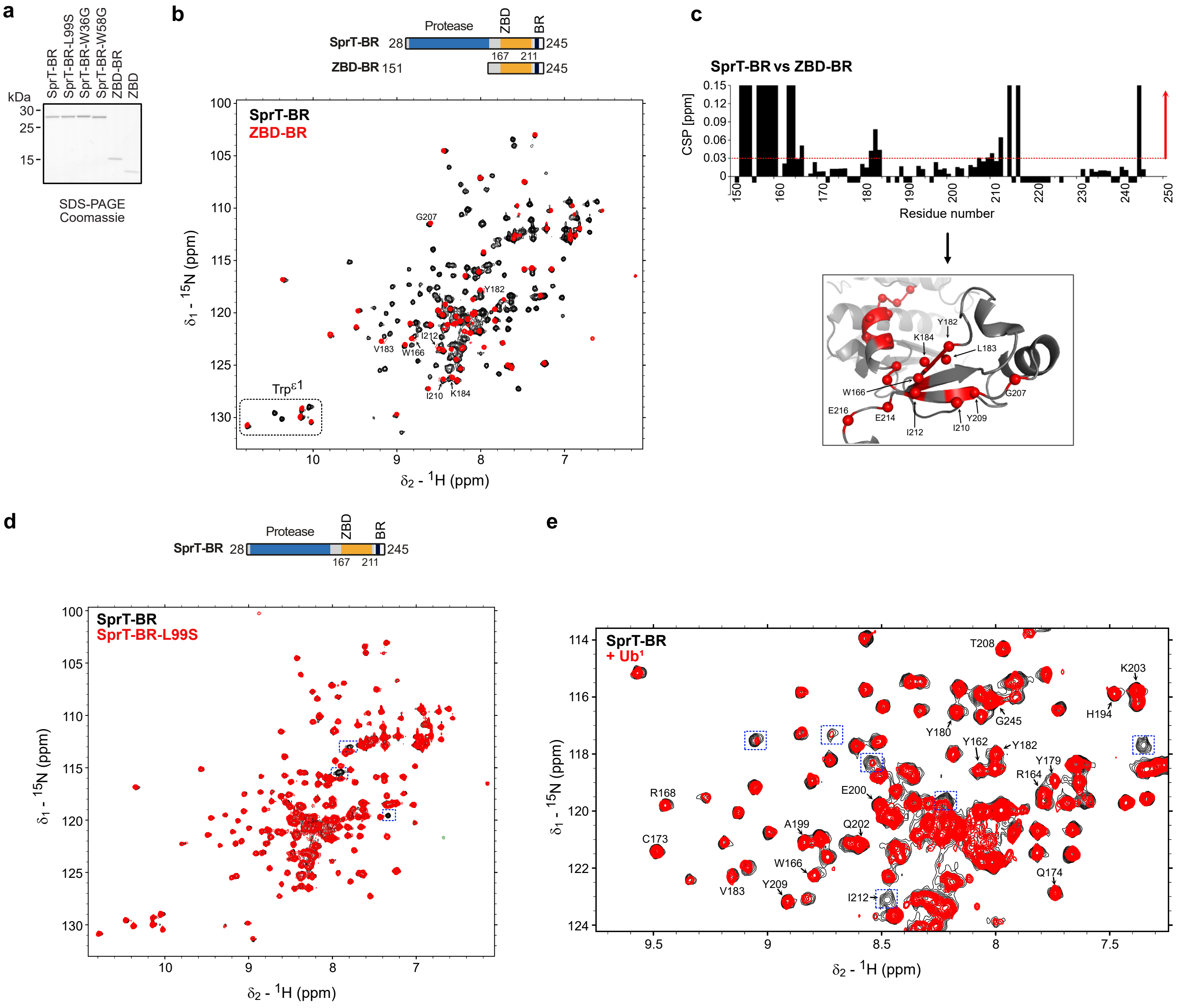

Supplementary Fig. 3. NMR analysis of SPRTN's SprT domain

(a) Coomassie stained SDS-PAGE gel, showing equimolar amounts of purified recombinant human SprT-BR, SprT-BR- L99S, SprT-BR-W36G, SprT-BR-W58G, ZBD-BR, and ZBD used for NMR measurements.

(b) Comparison of NMR spectra of SprT-BR (aa28-245) (black) and ZBD-BR (aa151-245) (red). Some representative peaks with Chemical Shift Perturbation (CSP) > 0.3 ppm are annotated on the spectrum. The crowded middle region corresponds to the unstructured linkers and the *C*-terminal BR region.

(c) CSP of SprT-BR against ZBD-BR. Residues with CSP > 0.3 ppm (red arrow) are highlighted in the structure as red spheres with labels for some representative residues (bottom). Negative values indicate proline or unassigned residues. Large changes, which could not be traced are given a CSP value of 1.5 ppm. CSP differences between SprT-BR and ZBD-BR highlight some differences in the linker between protease and ZBD (aa151-160) and on the β-sheet fold of the ZBD. Nonetheless, we were able to transfer many chemical shifts from our previous analysis of the ZBD-BR construct[21]. The large CSPs of residues aa151-160, presumably reflect interaction of this region with the protease domain. Due to the non-optimal sample stability of SprT-BR, we could not assign the individual resonances of the protease domain. However, by exclusion and introducing mutations of the tryptophanes in the protease domain (W36G and W58G), the resonances belonging to the protease domain could readily be distinguished from those in the ZBD and BR (see Fig. 3 for the Trp ε1 resonances).

(d) Comparison of NMR spectra of SprT-BR (black) and SprT-BR-L99S (red). Spectral differences are highlighted with blue boxes.

(e) Comparison of NMR spectra of SprT-BR alone (black) and with mono-ubiquitin (Ub^1) (5x molar excess) (red). Some resonances corresponding to the zinc-binding domain (ZBD) are labeled for the dispersed region. Unlabeled resonances in the dispersed region generally correspond to the protease domain. The broadened signals in the presence of Ub^1 are highlighted with blue boxes. Full spectrum shown in Fig. 3a.

SUPPLEMENTARY FIGURE 4

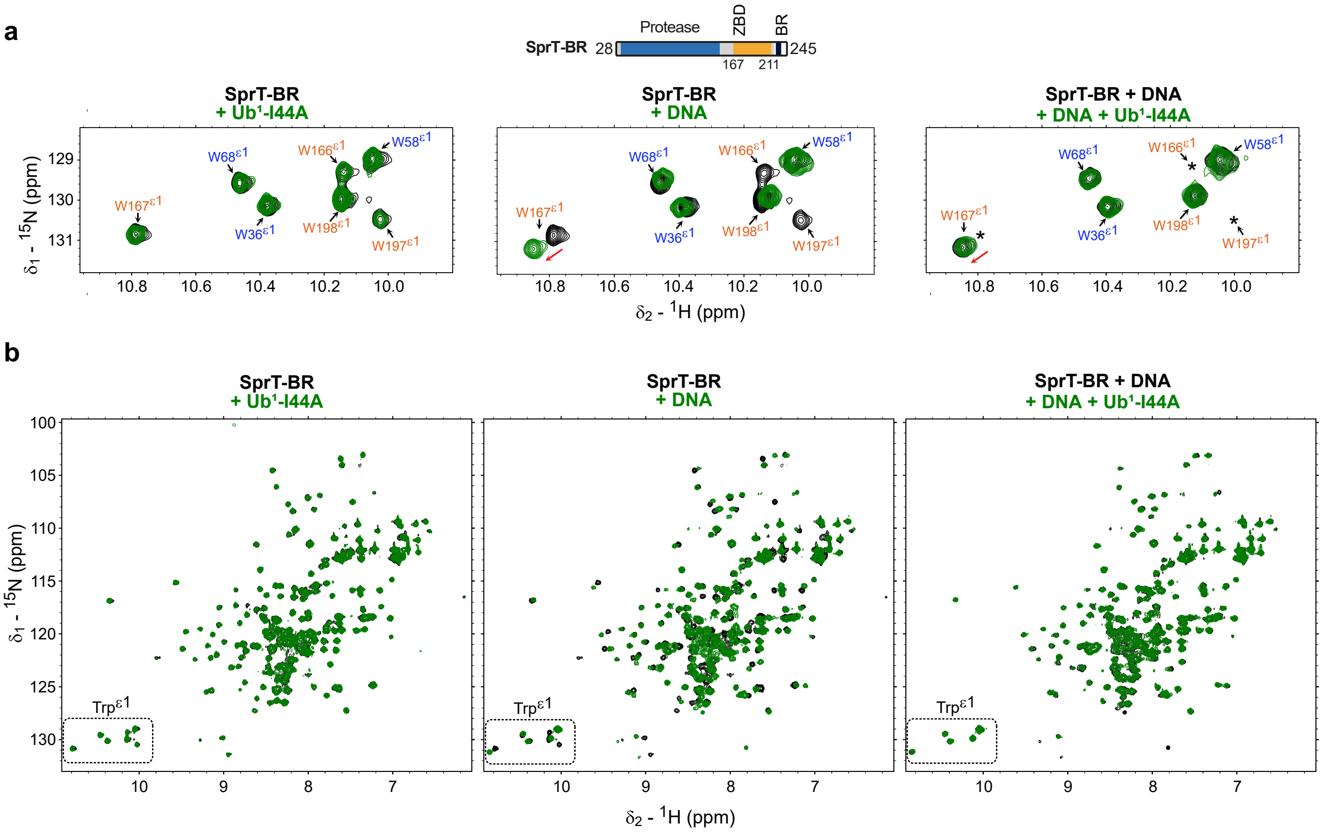

Supplementary Fig. 4. Ubiquitin binding at the USD depends on ubiquitin's Ile44 patch

(a) Comparison of NMR spectra (Trp ε1 amide signals in ^{1}H,^{15}N-HSQC experiments) of SprT-BR alone (black) (=Apo), with mono-ubiquitin (Ub^1)-I44A (5x molar excess) (green) (left) or with dsDNA (2x molar excess) (green) (middle). Right panel shows superimpositions of SprT-BR in the presence of dsDNA (2x molar excess) (black) and of both dsDNA (2x molar excess) and Ub^1-I44A (5x molar excess) (green). Resonances corresponding to the Trp ε1's in the ZBD are labeled in orange or shown as asterisk when broadened. Trp ε1's in the protease domain are labeled in blue. Full spectra are shown in (b).

(b) Full NMR spectra of Trp ε1 amide signals shown in (a). Trp ε1 region is boxed.

SUPPLEMENTARY FIGURE 5

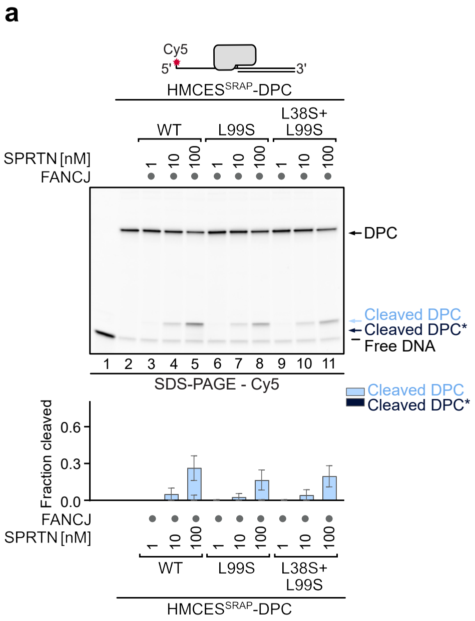

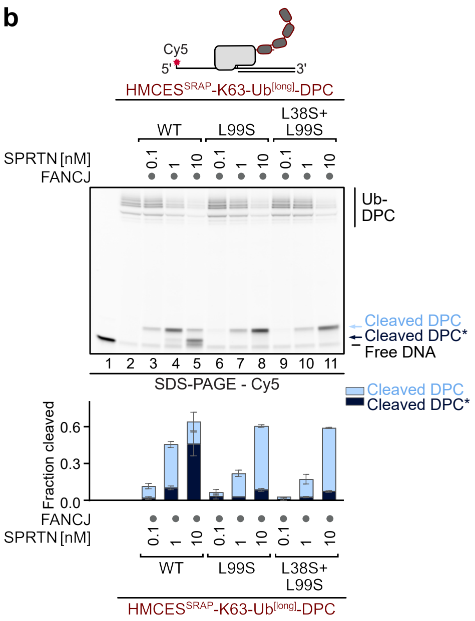

Supplementary Fig. 5. The USD promotes cleavage of ubiquitylated DPC by SPRTN.
(a-b) Indicated HMCES^{SRAP}-DPCs (10 nM) were incubated alone or in the presence of FANCJ (100 nM) and indicated concentrations (0.1-100 nM) and variants of SPRTN (WT, L99S, L38S+L99S) for 1 h at 30°C. Quantification: bar graphs represent the mean ± SD of three independent experiments. Source data are provided as a Source Data file.

SUPPLEMENTARY FIGURE 6

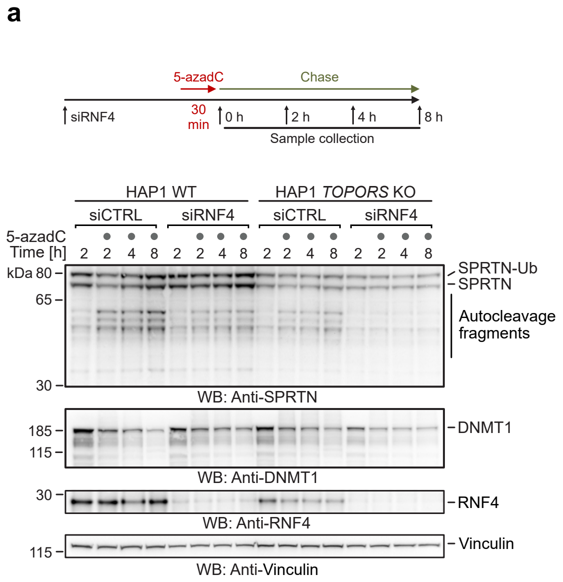

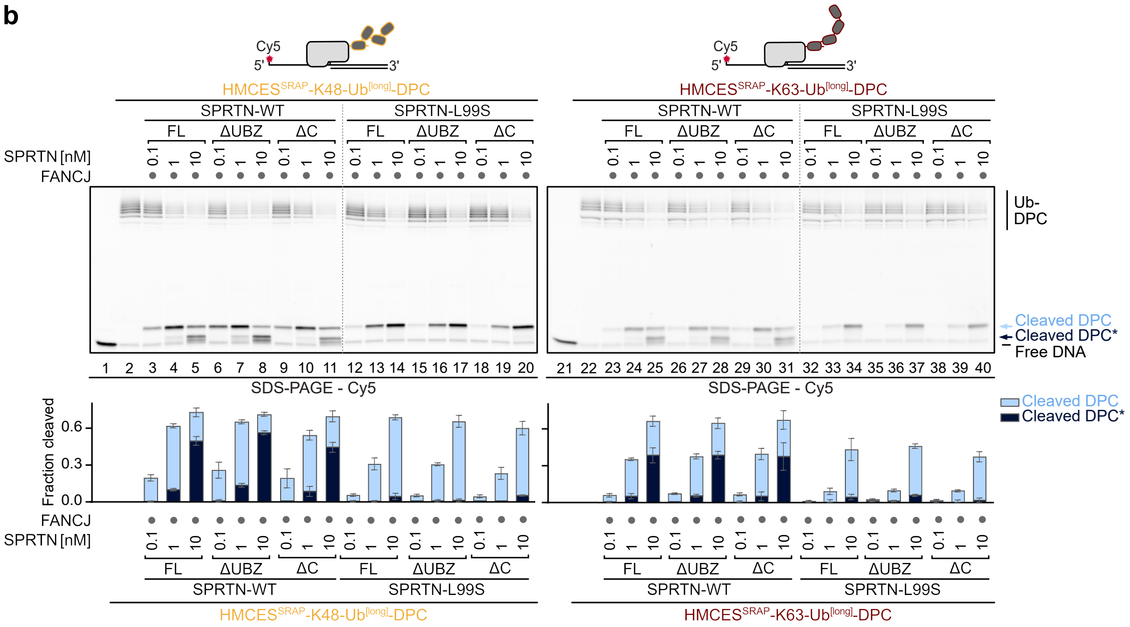

Supplementary Fig. 6. The USD is especially important in hypomorphic SPRTN variants

(a) HAP1 wild-type (WT) or HAP1 *TOPORS* knock-out (KO) cells transfected with indicated siRNAs were treated with 5-azadC (10 µM) and harvested as depicted (top). Whole cell lysates were analyzed by immunoblotting (bottom). Source data are provided as a Source Data file.

(b) Indicated HMCES^{SRAP}-Ub$^{[long]}$-DPCs (10 nM) were incubated alone or in the presence of recombinant FANCJ (100 nM) and indicated concentrations (0.1-10 nM) and variants of SPRTN (FL-WT/L99S, ΔUBZ-WT/L99S, ΔC-WT/L99S) for 1 h at 30°C. Quantification: bar graphs represent the mean ± SD of three independent experiments. All samples derive from the same experiment and gels were processed in parallel. Source data are provided as a Source Data file.

SUPPLEMENTARY FIGURE 7

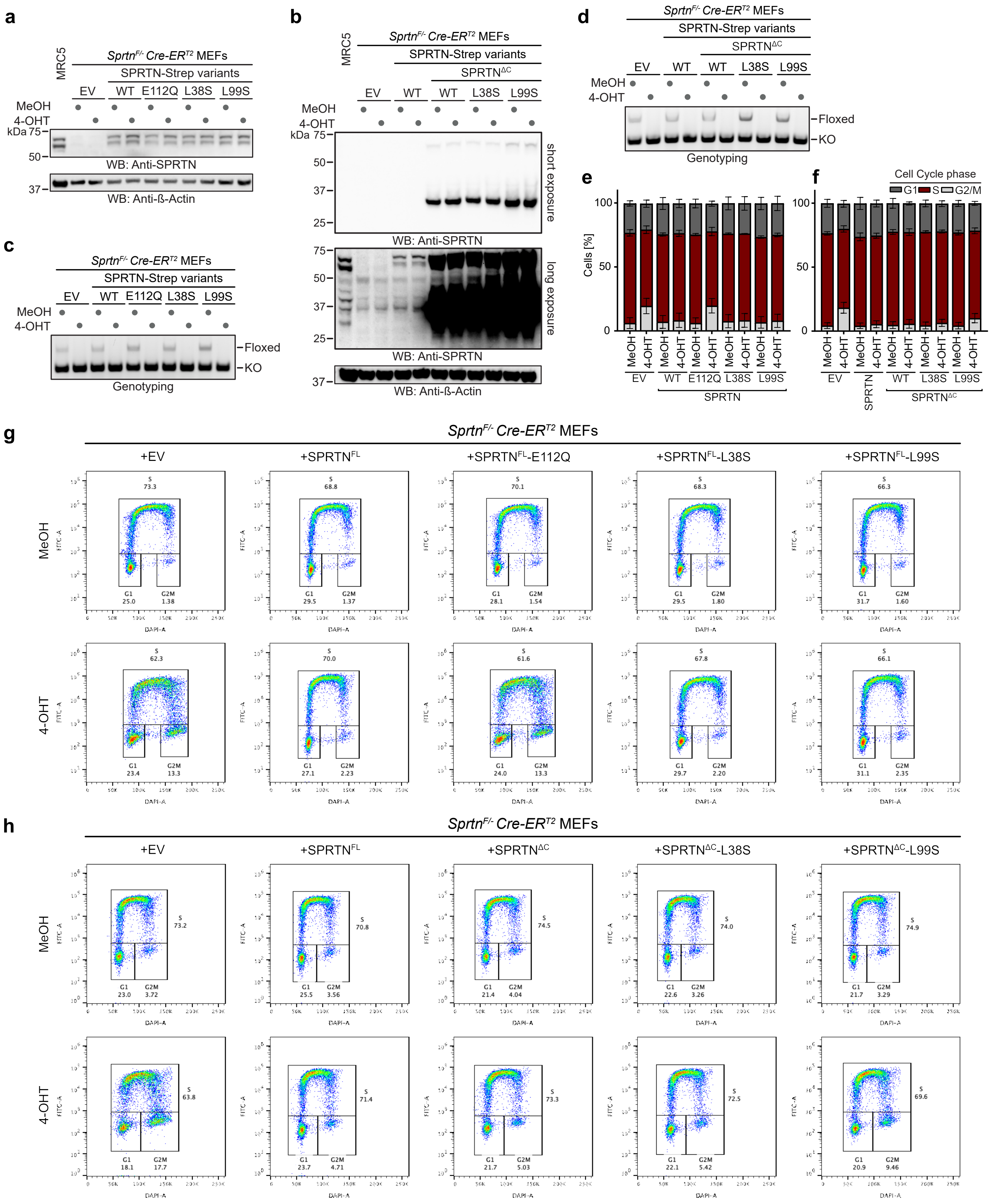

Supplementary Fig. 7. Ubiquitin-dependent activation of SPRTN maintains genome stability in Ruijs-Aalfs syndrome.

(a-b) Expression of indicated SPRTN variants or empty vector (EV) (pMSCV) in *Sprtn*$^{F/-}$ Cre-ER^{T2} mouse embryonic fibroblasts (MEFs) treated with methanol (MeOH) or (Z)-4-hydroxytamoxifen (4-OHT) (2 μM) for 48 h. Whole cell lysates were anlayzed by immunoblotting. Source data are provided as a Source Data file.

(c-d) PCR-based genotyping of Sprtn alleles in *Sprtn*$^{F/-}$ Cre-ER^{T2} MEFs, complemented with indicated SPRTN variants or EV (pMSCV), treated with MeOH or 4-OHT (2 μM) for 48 h. Source data are provided as a Source Data file.

(e-h) Cell cycle profiling of *Sprtn*$^{F/-}$ Cre-ER^{T2} MEFs, complemented with indicated SPRTN variants or EV (pMSCV), treated with MeOH or 4-OHT (2 μM) for 48 h. Cells were labeled with EdU for 45 min and analyzed by flow cytometry. Bar charts represent the mean ± SD of three independent experiments (e-f). Flow charts show a representative of these three experiments (g-h). Source data are provided as a Source Data file.

Supplementary Table 1

Sample	MW (kDa)	Input		Resuspended in (mL)	Con		Measurement (mean of all measured wavelengths)							
		µL	mg		mg/mL	µM	Co		Fe		Mn		Zn	
							µg/mL	µM	µg/mL	µM	µg/mL	µM	µg/mL	µM
TB	-	160	-	10	-	**-**	0.0035	**-**	0.081	**-**	0.001667	**-**	0.075333	**-**
SPRTN	55.2	160	0.95	10	0.095	**1.72**	0.002	**0.03**	0.03125	**0.56**	<LOD	**<LOD**	0.348	**5.32**

Atomic mass metals	
Fe	55.845
Zn	65.380
Mn	54.938
Co	58.933

LOD = Limit of Detection

Supplementary Table 1. ICP-OES measurements.

Inductively Coupled Plasma Optical Emission Spectrometry (ICP-OES) measurement results for recombinant SPRTN and the used expression media (TB). The file contains: The Molecular weight (kDa), input volume (µL) and mass (mg), volume for resuspension (mL), concentration of sample (mg/mL and µM) and corresponding measured amounts (mg/mL and µM) for Co, Fe, Mn and Zn (mean of all measured wavelengths. Additionally, the atomic mass for Co, Fe, Mn and Zn are given. LOD = Limit of Detection.

Supplementary Table 2

Overview:

Model	$n_{Cluster}$	Fraction Σ Top 3-Cluster
**SprT - PDB: 6mdx**	1	1.00
**SprT - ColabFold**	9	0.98
**SprT-L38S - ColabFold**	19	0.77
**SprT-L99S - ColabFold**	8	0.95
**SprT+Ub[1] - ColabFold**	9	0.97
**SprT-L38S+Ub[1] - ColabFold**	9	0.98
**SprT-L99S+Ub[1] - ColabFold**	3	1.00

Details:

Model	Cluster	Structures	Prob.	Rg [nm]	SD [nm]	n: 90003
**SprT - PDB: 6mdx**	1	90003	1.00	1.71	0.02	
**SprT - ColabFold**	1	37295	0.41	2.02	0.06	
	2	30001	0.33	1.84	0.04	
	3	21072	0.23	2.05	0.06	
	All	90003	1.00	1.97	0.11	
**SprT-L38S - ColabFold**	1	43149	0.48	2.06	0.08	
	2	18669	0.21	1.78	0.07	
	3	7375	0.08	2.16	0.04	
	All	90003	1.00	2.01	0.15	
**SprT-L99S - ColabFold**	1	34661	0.39	2.08	0.08	
	2	30916	0.34	1.75	0.05	
	3	19954	0.22	1.92	0.07	
	All	90003	1.00	1.93	0.17	
**SprT+Ub1 - ColabFold**	1	79430	0.88	2.03	0.07	
	2	5367	0.06	2.05	0.04	
	3	2796	0.03	2.05	0.06	
	All	90003	1.00	2.03	0.07	
**SprT-L38S+Ub1 - ColabFold**	1	71075	0.79	2.05	0.05	
	2	10972	0.12	2.06	0.03	
	3	6318	0.07	2.02	0.07	
	All	90003	1.00	2.05	0.06	
**SprT-L99S+Ub1 - ColabFold**	1	76721	0.85	2.09	0.05	
	2	10649	0.12	1.91	0.10	
	3	2633	0.03	2.08	0.05	
	All	90003	1.00	2.07	0.08	

Supplementary Table 2. Clustering overview MD-simulations.

Summary of numbers of clusters from molecular dynamics (MD) simulations of SprT (PDB: 6mdx), SprT (ColabFold), SprT-L38S (ColabFold), SprT-L99S (ColabFold), SprT-Ub[1] (ColabFold), SprT-L38S+Ub[1] (ColabFold), SprT-L99S+Ub[1] (ColabFold). The file contains: number of clusters (n) and the fraction Σ of the top 3 cluster, for each model (Overview); On the second sheet (Detail): for each model, the three largest clusters (SprT – PDB: 6mdx only showed one cluster) are listed along with their probability (Prob.;based on the number of structures), their radius of gyration (Rg; nm) including standard deviation (SD; nm), as well as the radius of gyration when all structures of a model are considered (All).

Log_2-transformed normalised intensities of all proteins measured and depicted in Fig. 1d-e. The file contains: The Uniprot identifier (UniprotID), HGNC gene symbol (Gene name), the normalised log_2-transformed intensity in each replicate of the respective conditions (FA_Rn: formaldehyde-treated replicate n, FABenzCrtl_Rn: Formaldehyde-treated replicate n nuclease control, Untr_Rn: untreated replicate n and UntrBenzCrtl_Rn: untreated replicate n nuclease control. n indicates the replicate numbers 1-6). NA accounts for non-detected.
