# Supplementary material for: Allosteric activation of the SPRTN protease by ubiquitin maintains genome stability
Source: Nat Commun. 2025 Jul 21;16:5422. doi: 10.1038/s41467-025-61224-z (PMC12279946; doi:10.1038/s41467-025-61224-z)
Supplement: Supplementary file 2 — Description of Additional Supplementary Files [file 41467_2025_61224_MOESM2_ESM.pdf]

Description of Additional Supplementary Files

File name: Supplementary Data 1
Description: Model for SprT based on Alphafold 2 protein structure prediction and protonation states predicted by H++ server.

File name: Supplementary Data 2
Description: Model for SprT-L38S based on Alphafold 2 protein structure prediction and protonation states predicted by H++ server.

File name: Supplementary Data 3
Description: Model for SprT-L99S based on Alphafold 2 protein structure prediction and protonation states predicted by H++ server.

File name: Supplementary Movie 1
Description: MD-Simulation of SprT (PDB:6mdx)

File name: Supplementary Movie 2
Description: MD-Simulation of SprT, open conformation (ColabFold)

File name: Supplementary Movie 3
Description: MD-Simulation of SprT-Ub^1 (ColabFold)
