# Supplementary material for: Allosteric activation of the SPRTN protease by ubiquitin maintains genome stability
Source: Nat Commun. 2025 Jul 21;16:5422. doi: 10.1038/s41467-025-61224-z (PMC12279946; doi:10.1038/s41467-025-61224-z)
Supplement: Supplementary file 9 — Reporting Summary [file 41467_2025_61224_MOESM9_ESM.pdf]

nature portfolio

Corresponding author(s): Julian Stingele

Last updated by author(s): Jun 3, 2025

Reporting Summary

Nature Portfolio wishes to improve the reproducibility of the work that we publish. This form provides structure for consistency and transparency in reporting. For further information on Nature Portfolio policies, see our Editorial Policies and the Editorial Policy Checklist.

Statistics

For all statistical analyses, confirm that the following items are present in the figure legend, table legend, main text, or Methods section.

n/a	Confirmed	
☐	☒	The exact sample size (*n*) for each experimental group/condition, given as a discrete number and unit of measurement
☐	☒	A statement on whether measurements were taken from distinct samples or whether the same sample was measured repeatedly
☐	☒	The statistical test(s) used AND whether they are one- or two-sided<br>*Only common tests should be described solely by name; describe more complex techniques in the Methods section.*
☒	☐	A description of all covariates tested
☐	☒	A description of any assumptions or corrections, such as tests of normality and adjustment for multiple comparisons
☐	☒	A full description of the statistical parameters including central tendency (e.g. means) or other basic estimates (e.g. regression coefficient) AND variation (e.g. standard deviation) or associated estimates of uncertainty (e.g. confidence intervals)
☐	☒	For null hypothesis testing, the test statistic (e.g. *F*, *t*, *r*) with confidence intervals, effect sizes, degrees of freedom and *P* value noted<br>*Give P values as exact values whenever suitable.*
☒	☐	For Bayesian analysis, information on the choice of priors and Markov chain Monte Carlo settings
☒	☐	For hierarchical and complex designs, identification of the appropriate level for tests and full reporting of outcomes
☒	☐	Estimates of effect sizes (e.g. Cohen's *d*, Pearson's *r*), indicating how they were calculated

Our web collection on statistics for biologists contains articles on many of the points above.

Software and code

Policy information about availability of computer code

Data collection	ImageLab (Bio-Rad) Version 5.2, FACSDivaTM v9.7, NMRPIPE, TOPSPIN 3.7, Gen5 v3.14,
Data analysis	• ColabFold v1.5.5<br>• AlphaFold2<br>• Graph Pad Prism 10.3.0<br>• ImageJ v1.54f<br>• PyMOL v3.0.3<br>• Affinity Designer v1.10.6.1665<br>• FlowJo v10.10<br>• MaxQuant v1.5.2.8<br>• Gromacs simulation package v2020.7<br>• NMRFAM-SPARKY

For manuscripts utilizing custom algorithms or software that are central to the research but not yet described in published literature, software must be made available to editors and reviewers. We strongly encourage code deposition in a community repository (e.g. GitHub). See the Nature Portfolio guidelines for submitting code & software for further information.

Data

Policy information about availability of data

All manuscripts must include a data availability statement. This statement should provide the following information, where applicable:

- Accession codes, unique identifiers, or web links for publicly available datasets
- A description of any restrictions on data availability
- For clinical datasets or third party data, please ensure that the statement adheres to our policy

Mass spectrometry data reported in this manuscript were deposited to the ProteomeXchange Consortium (www.proteomexchange.org) via the Proteomics Identification Database (PRIDE) partner repository with the dataset identifier: PXD063921.
Source data are provided with this paper. All other data supporting this study are available from the corresponding author upon request.

Research involving human participants, their data, or biological material

Policy information about studies with human participants or human data. See also policy information about sex, gender (identity/presentation), and sexual orientation and race, ethnicity and racism.

Reporting on sex and gender	N/A
Reporting on race, ethnicity, or other socially relevant groupings	N/A
Population characteristics	N/A
Recruitment	N/A
Ethics oversight	N/A

Note that full information on the approval of the study protocol must also be provided in the manuscript.

Field-specific reporting

Please select the one below that is the best fit for your research. If you are not sure, read the appropriate sections before making your selection.

☒ Life sciences ☐ Behavioural & social sciences ☐ Ecological, evolutionary & environmental sciences

For a reference copy of the document with all sections, see nature.com/documents/nr-reporting-summary-flat.pdf

Life sciences study design

All studies must disclose on these points even when the disclosure is negative.

Sample size	No statistical methods were used to pre-determine sample sizes, which were chosen based on previous experience with these type of experiments. For quantified results, the number of replicates is indicated in the figure legends.
Data exclusions	No data was excluded from this study.
Replication	Figures display representative results from one independent experiment. Every experiment was performed n≥3 as independent experiments with similar results. Quantifications of experimental data correspond to n≥3 independent experiments.
Randomization	In each experiment, samples were processed together and reactions and treatments were randomly allocated.
Blinding	Blinding was performed for relevant experiments and is indicated in Methods section.

Reporting for specific materials, systems and methods

We require information from authors about some types of materials, experimental systems and methods used in many studies. Here, indicate whether each material, system or method listed is relevant to your study. If you are not sure if a list item applies to your research, read the appropriate section before selecting a response.

Materials & experimental systems

n/a	Involved in the study	
☐	☒	Antibodies
☐	☒	Eukaryotic cell lines
☒	☐	Palaeontology and archaeology
☒	☐	Animals and other organisms
☒	☐	Clinical data
☒	☐	Dual use research of concern
☒	☐	Plants

Methods

n/a	Involved in the study	
☒	☐	ChIP-seq
☐	☒	Flow cytometry
☒	☐	MRI-based neuroimaging

Antibodies

Antibodies used

Anti-DNMT1 (D63A6) antibody (1:1000) (#5032, Cell Signaling)
Anti-Actin antibody (1:1000) (Sc-47778, Santa Cruz Biotechnology)
Anti-K48-Ubiquitin (D9D5) antibody (1:1000) (#8081S, Cell Signaling)
Anti-K63-Ubiquitin (D7A11) antibody (1:1000) (#5621S, Cell Signaling)
Anti-SPRTN antibody (1:500) (6F2) Stingele lab, Zhao et al 2021
Anti-RNF4 antibody (1:500) (AF7964, R&D systems)
Anti-Vinculin (1:1000) (sc-73614, Santa Cruz.)

Validation

All commercial antibodies were validated by suppliers and/or prior references. Antibodies were additionally validated as indicated below:

Anti-DNMT1 (D63A6) antibody (#5032, Cell Signaling): https://www.cellsignal.de/products/primary-antibodies/dnmt1-d63a6-xp-rabbit-mab/5032?Ntk=Products
Anti-Actin antibody (Sc-47778, Santa Cruz Biotechnology): https://www.scbt.com/p/beta-actin-antibody-c4
Anti-K48-Ubiquitin (D9D5) antibody (1:1000) (#8081S, Cell Signaling): https://www.cellsignal.com/products/primary-antibodies/k48-linkage-specific-polyubiquitin-d9d5-rabbit-mab/8081
Anti-K63-Ubiquitin (D7A11) antibody (1:1000) (#5621S, Cell Signaling): https://www.cellsignal.com/products/primary-antibodies/k63-linkage-specific-polyubiquitin-d7a11-rabbit-mab/5621
Anti-SPRTN antibody (6F2): Stingele lab, Zhao et al 2021, validated by overexpression
Anti-RNF4 antibody (AF7964, R&D systems): https://www.rndsystems.com/products/human-rnf4-antibody_af7964, further validated by siRNA.
Anti-Vinculin (sc-73614, Santa Cruz): https://www.scbt.com/p/vinculin-antibody-7f9

Eukaryotic cell lines

Policy information about cell lines and Sex and Gender in Research

Cell line source(s)

HeLa TREx Flp-In SPRTNΔC were generated by Weichert et al. 2023.
HAP1 WT cells (Horizon, cat. no. C631) and HAP1 TOPORS KO cells (Horizon cat. no. HZGHC008005c006) were purchased from Horizon.
SprtnF/- mouse embryonic fibroblasts (MEFs) (H7) were generated by Maskey et. al. 2014.
HEK 293T/17 cells were purchased from ATCC (ATCC cat. no. CRL-11268).

Authentication

HeLa T-REx Flp-In cells were originally obtained from The Francis Crick Institute and HeLa TREx Flp-In SPRTNΔC were generated by Weichert et al. 2023 and not further validated.
HAP1 cells (Horizon, WT: cat. no. C631, TOPORS KO: HZGHC008005c006) were purchased and not further validated.
SprtnF/- mouse embryonic fibroblasts (MEFs) (H7) were generated by Maskey et. al. 2014 and not further validated.
HEK 293T/17 cells (ATCC cat. no. CRL-11268) were purchased and not further validated.

Mycoplasma contamination

All cells tested negative for mycoplasma contamination with the Mycoplasma PCR Detetion Kit (abm, G238).

Commonly misidentified lines
(See ICLAC register)

No commonly misidentified cell lines were used in this study.

Plants

Seed stocks	N/A
Novel plant genotypes	N/A
Authentication	N/A

Flow Cytometry

Plots

Confirm that:

☒ The axis labels state the marker and fluorochrome used (e.g. CD4-FITC).

☒ The axis scales are clearly visible. Include numbers along axes only for bottom left plot of group (a 'group' is an analysis of identical markers).

☒ All plots are contour plots with outliers or pseudocolor plots.

☒ A numerical value for number of cells or percentage (with statistics) is provided.

Methodology

Sample preparation	Cells were labeled with EdU (10 µM) for 45 min. EdU staining was performed with the Click-iT EdU Alexa Fluor 488 Flow Cytometry Assay Kit (Thermo Scientific) following the manufacturer's protocol. Cells were next stained with 4',6-diamidino-2-phenylindole (DAPI) (4 µg/mL) (Thermo Scientific) and analyzed.
Instrument	BD LSRFortessa cell analyzer (BD Biosciences)
Software	FACSDivaTM software (ver. 6.2)
Cell population abundance	N/A
Gating strategy	N/A

☒ Tick this box to confirm that a figure exemplifying the gating strategy is provided in the Supplementary Information.
