# Supplementary figures and images for: Allosteric activation of the SPRTN protease by ubiquitin maintains genome stability (part 2 of 2)
Source: Nat Commun. 2025 Jul 21;16:5422. doi: 10.1038/s41467-025-61224-z (PMC12279946; doi:10.1038/s41467-025-61224-z)

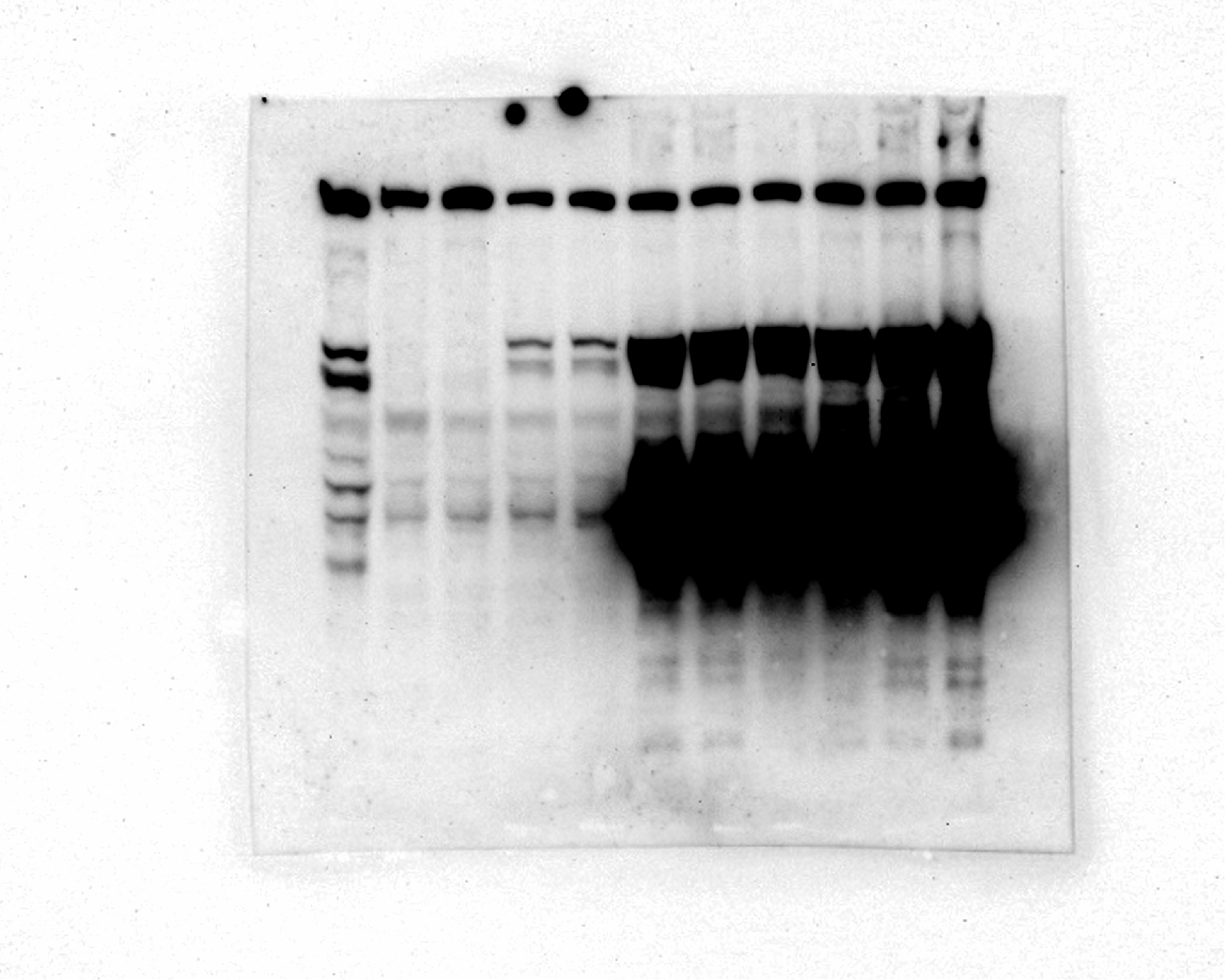

Supplement: Supplementary file 11 — Source Data [file 41467_2025_61224_MOESM11_ESM.zip › Source data/Uncropped scans of all blots and gels/Supplementary Fig. 7/Supplementary Fig. 7b/SPRTN/SPRTN_long.tif]

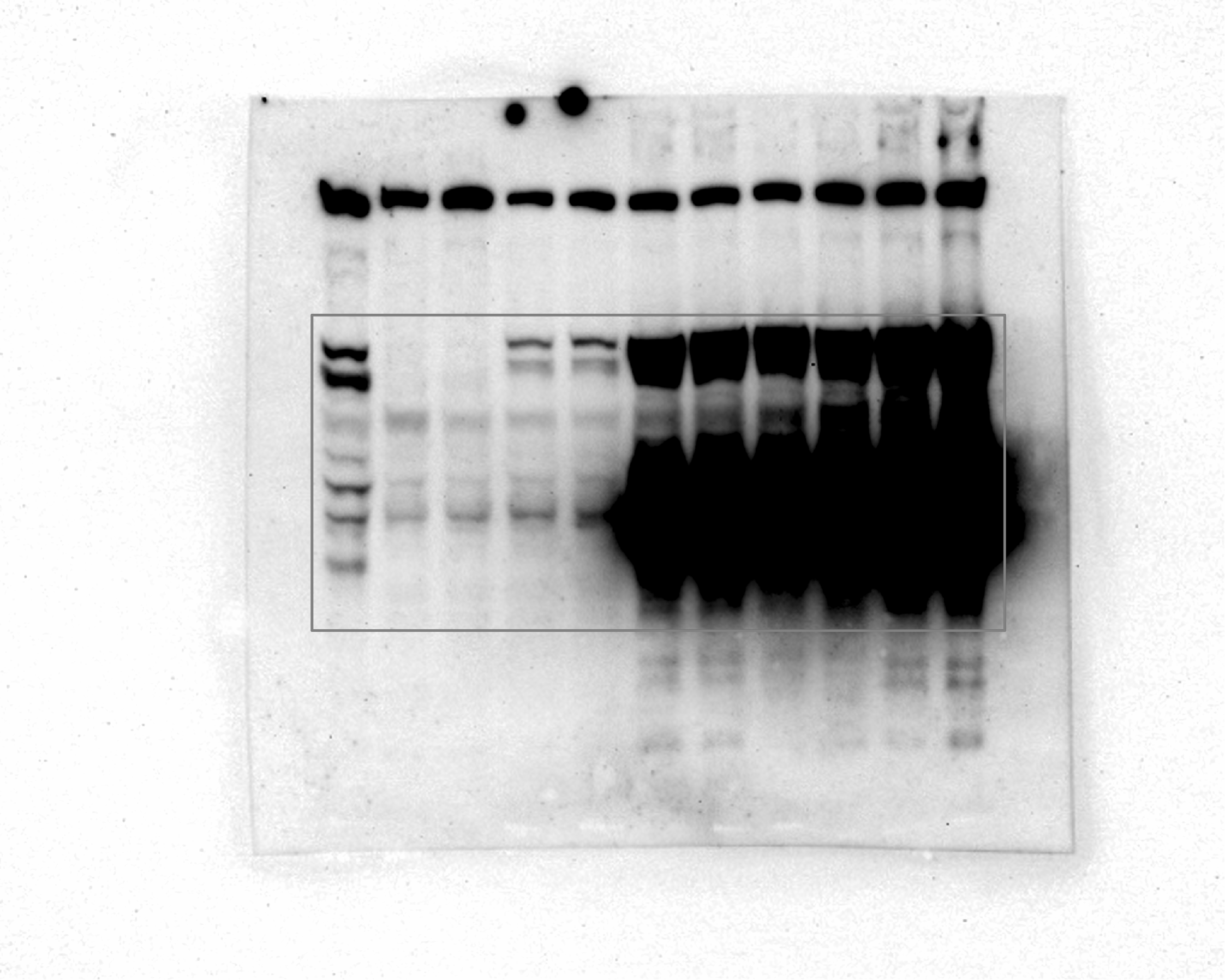

Supplement: Supplementary file 11 — Source Data [file 41467_2025_61224_MOESM11_ESM.zip › Source data/Uncropped scans of all blots and gels/Supplementary Fig. 7/Supplementary Fig. 7b/SPRTN/SPRTN_long_label.tiff]

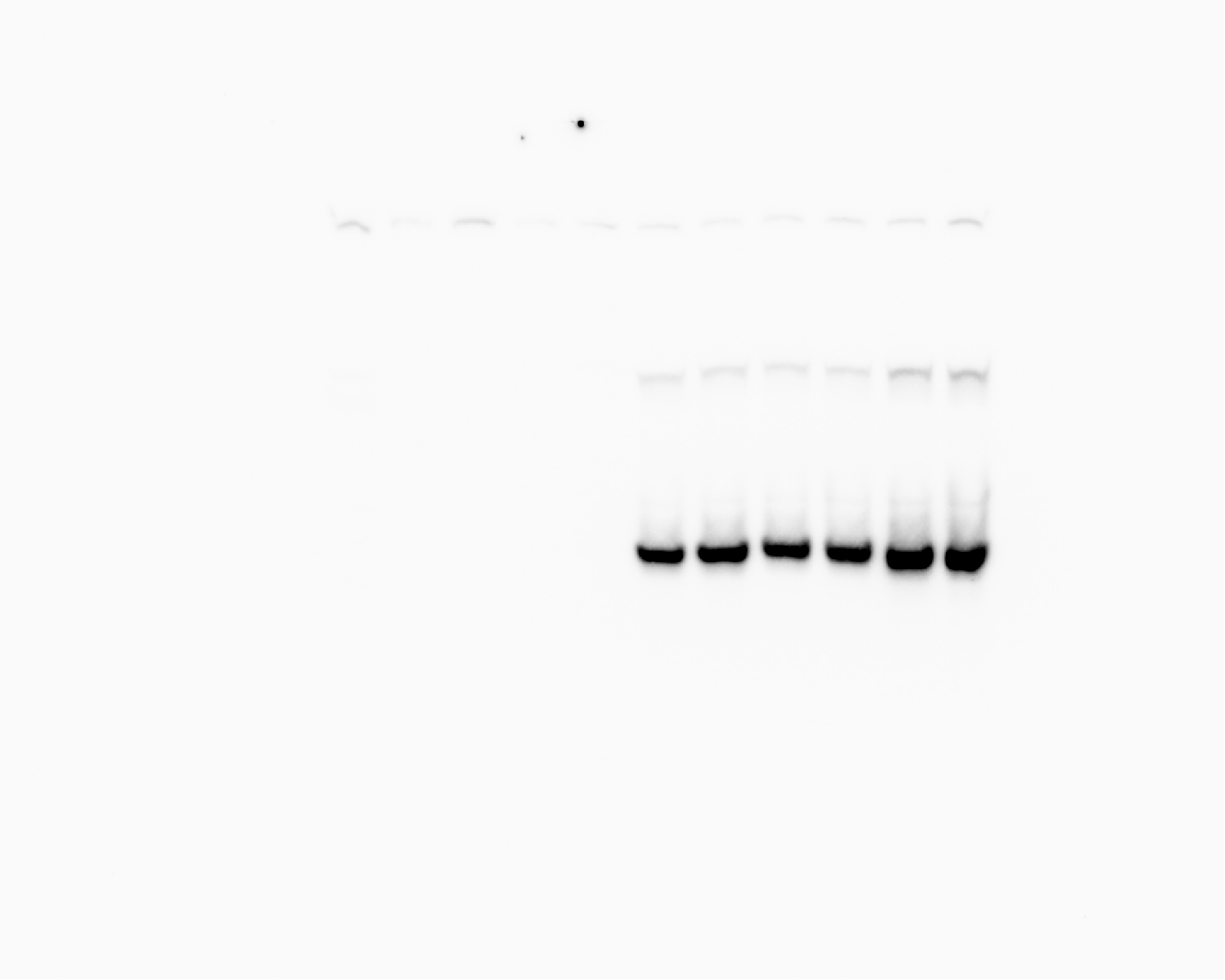

Supplement: Supplementary file 11 — Source Data [file 41467_2025_61224_MOESM11_ESM.zip › Source data/Uncropped scans of all blots and gels/Supplementary Fig. 7/Supplementary Fig. 7b/SPRTN/SPRTN_short.tif]

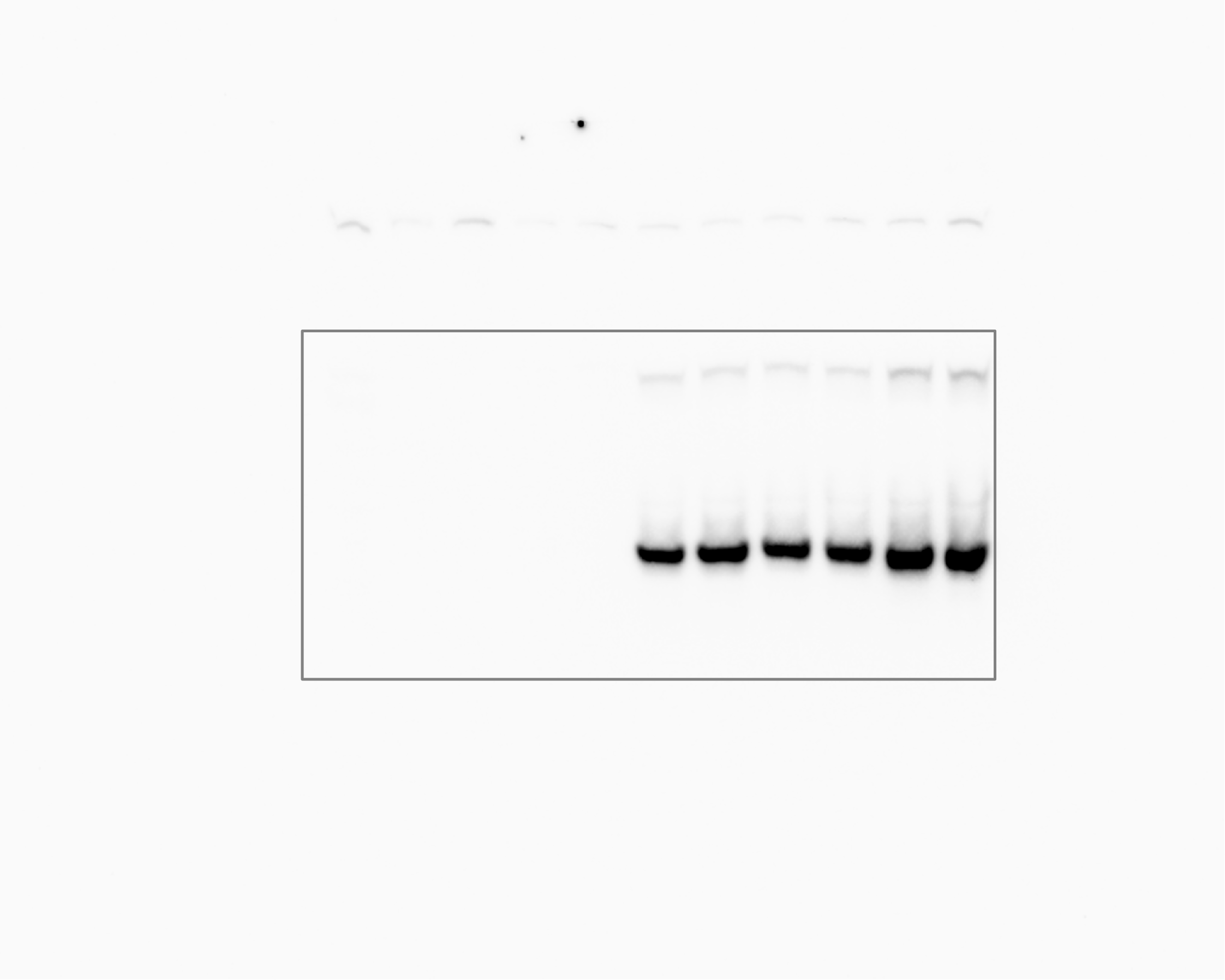

Supplement: Supplementary file 11 — Source Data [file 41467_2025_61224_MOESM11_ESM.zip › Source data/Uncropped scans of all blots and gels/Supplementary Fig. 7/Supplementary Fig. 7b/SPRTN/SPRTN_short_label.tiff]

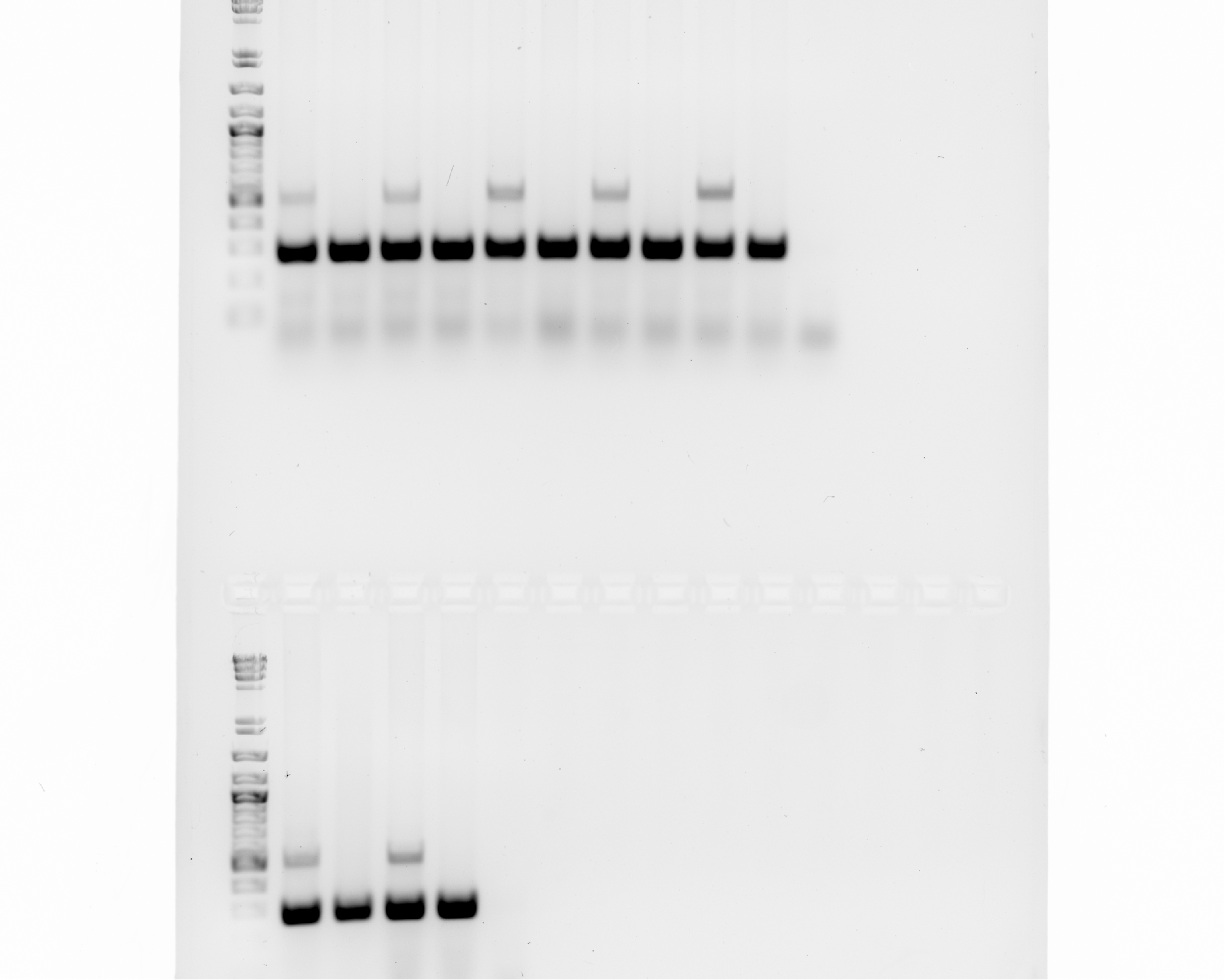

Supplement: Supplementary file 11 — Source Data [file 41467_2025_61224_MOESM11_ESM.zip › Source data/Uncropped scans of all blots and gels/Supplementary Fig. 7/Supplementary Fig. 7c/Genotyping.tif]

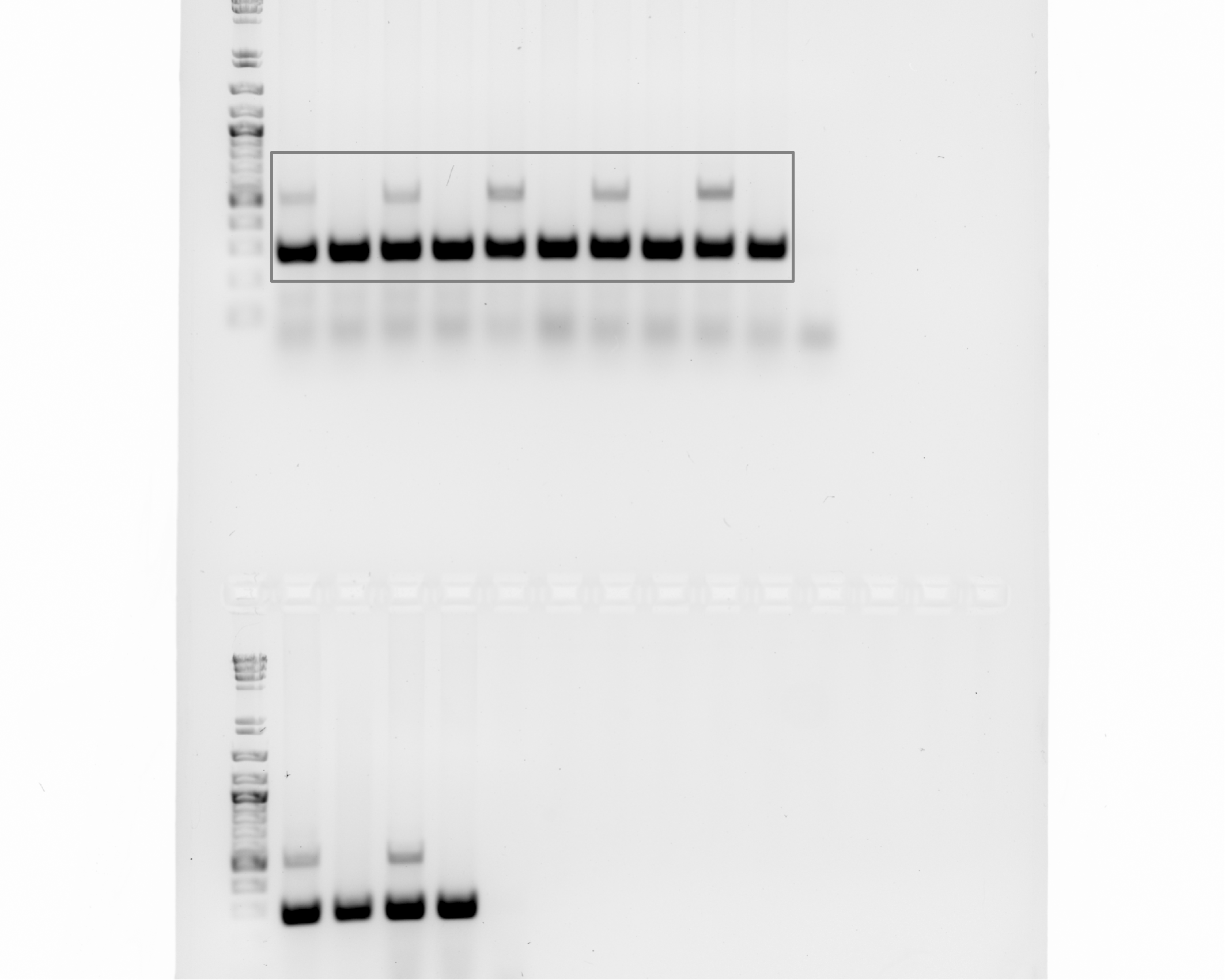

Supplement: Supplementary file 11 — Source Data [file 41467_2025_61224_MOESM11_ESM.zip › Source data/Uncropped scans of all blots and gels/Supplementary Fig. 7/Supplementary Fig. 7c/Genotyping_label.tiff]

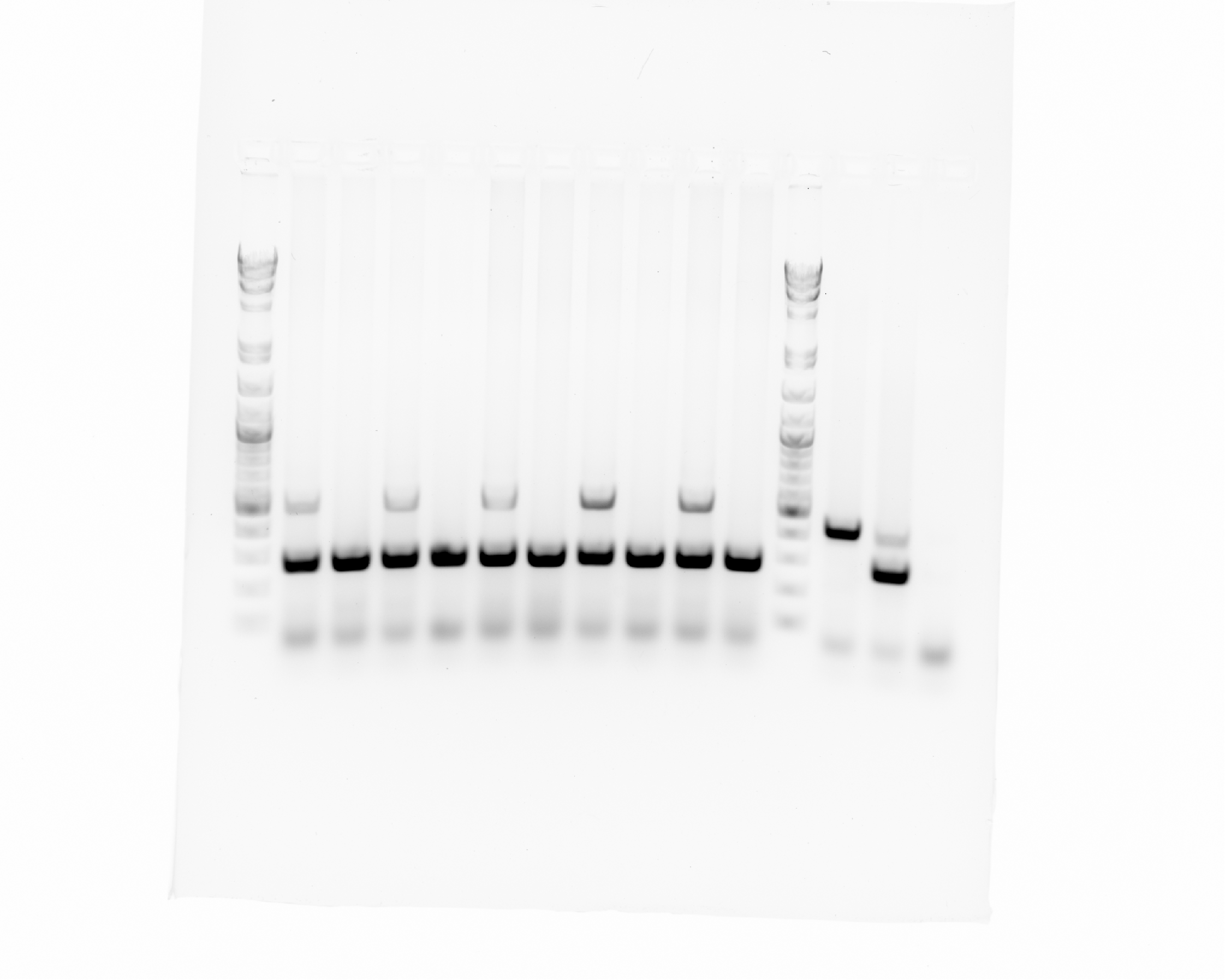

Supplement: Supplementary file 11 — Source Data [file 41467_2025_61224_MOESM11_ESM.zip › Source data/Uncropped scans of all blots and gels/Supplementary Fig. 7/Supplementary Fig. 7d/Genotyping.tif]

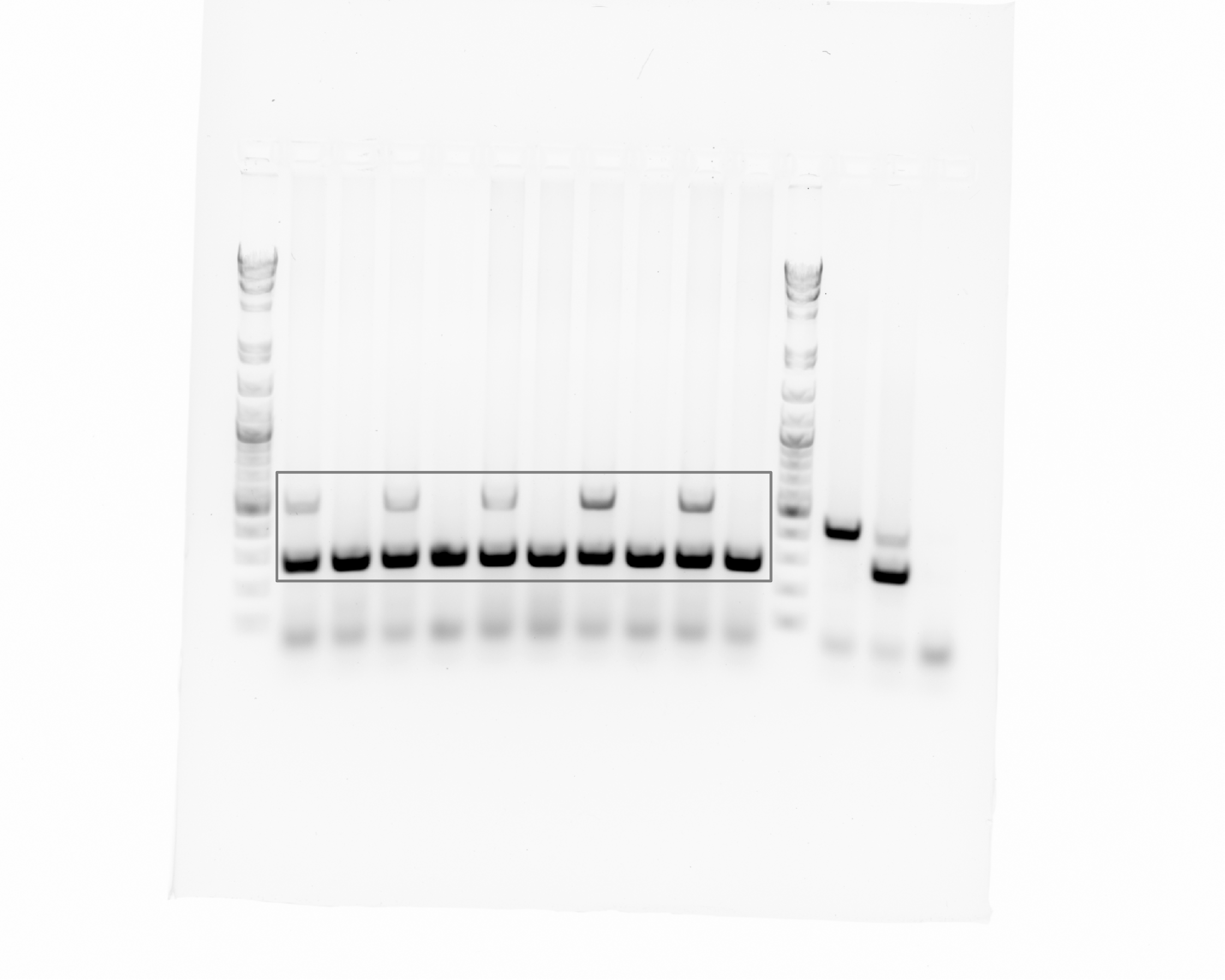

Supplement: Supplementary file 11 — Source Data [file 41467_2025_61224_MOESM11_ESM.zip › Source data/Uncropped scans of all blots and gels/Supplementary Fig. 7/Supplementary Fig. 7d/Genotyping_label.tiff]
