# Supplementary figures and images for: Allosteric activation of the SPRTN protease by ubiquitin maintains genome stability (part 1 of 2)
Source: Nat Commun. 2025 Jul 21;16:5422. doi: 10.1038/s41467-025-61224-z (PMC12279946; doi:10.1038/s41467-025-61224-z)

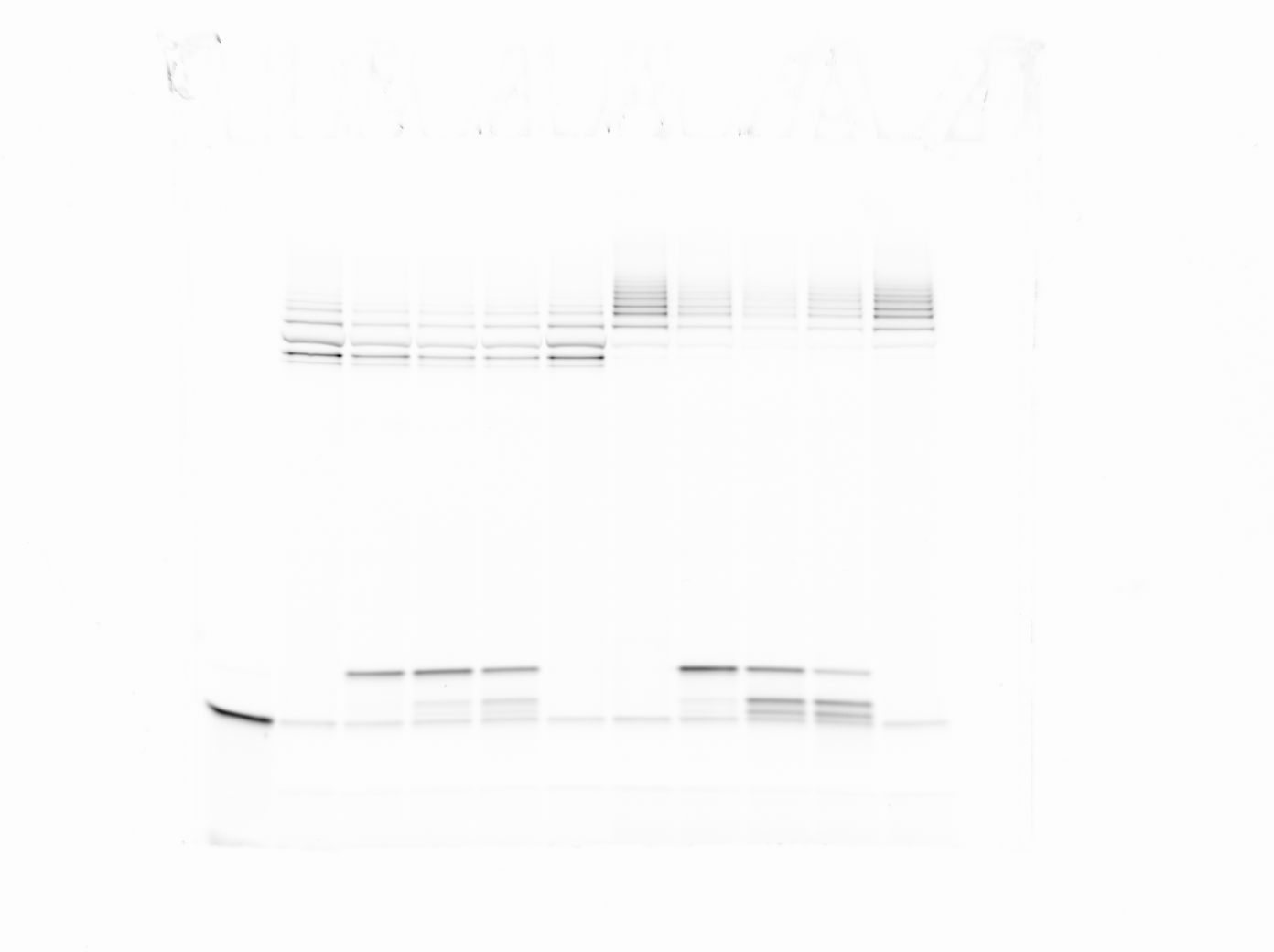

Supplement: Supplementary file 11 — Source Data [file 41467_2025_61224_MOESM11_ESM.zip › Source data/Uncropped scans of all blots and gels/Fig. 1/Fig. 1e/K48/K48-SRAP_Cy5.tif]

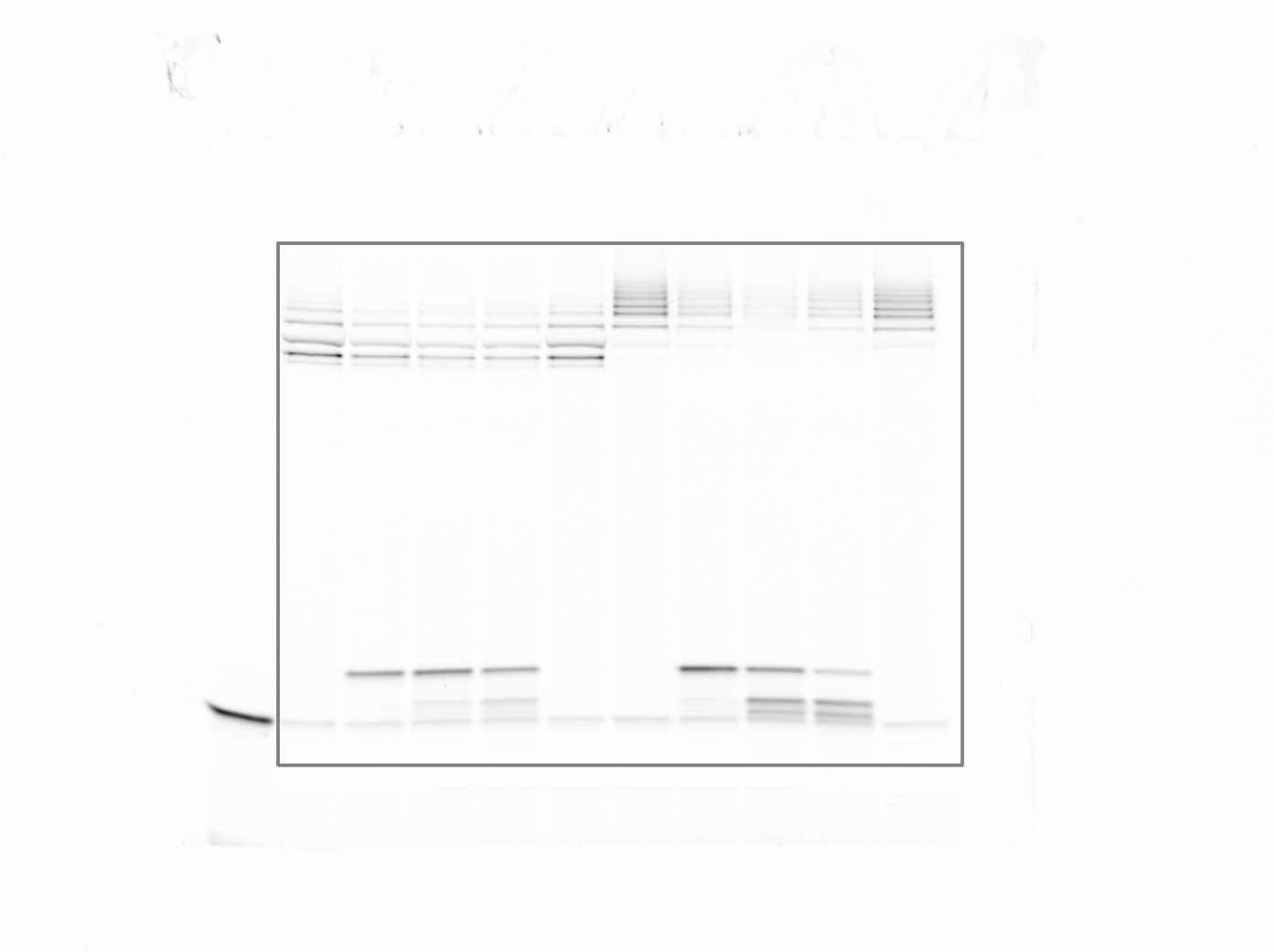

Supplement: Supplementary file 11 — Source Data [file 41467_2025_61224_MOESM11_ESM.zip › Source data/Uncropped scans of all blots and gels/Fig. 1/Fig. 1e/K48/K48-SRAP_label_Cy5.tiff]

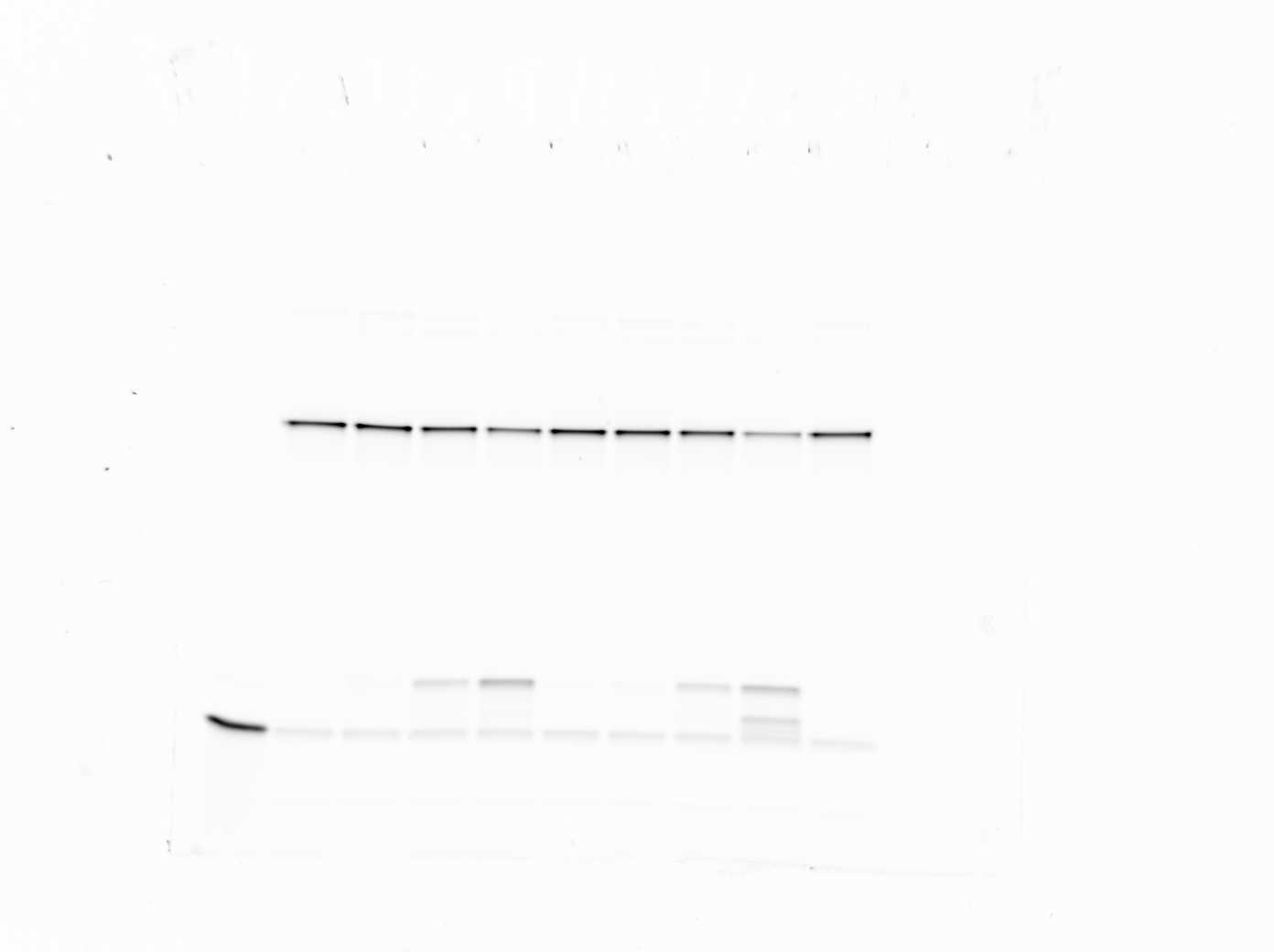

Supplement: Supplementary file 11 — Source Data [file 41467_2025_61224_MOESM11_ESM.zip › Source data/Uncropped scans of all blots and gels/Fig. 1/Fig. 1e/K48/Unmodified-SRAP_Cy5.tif]

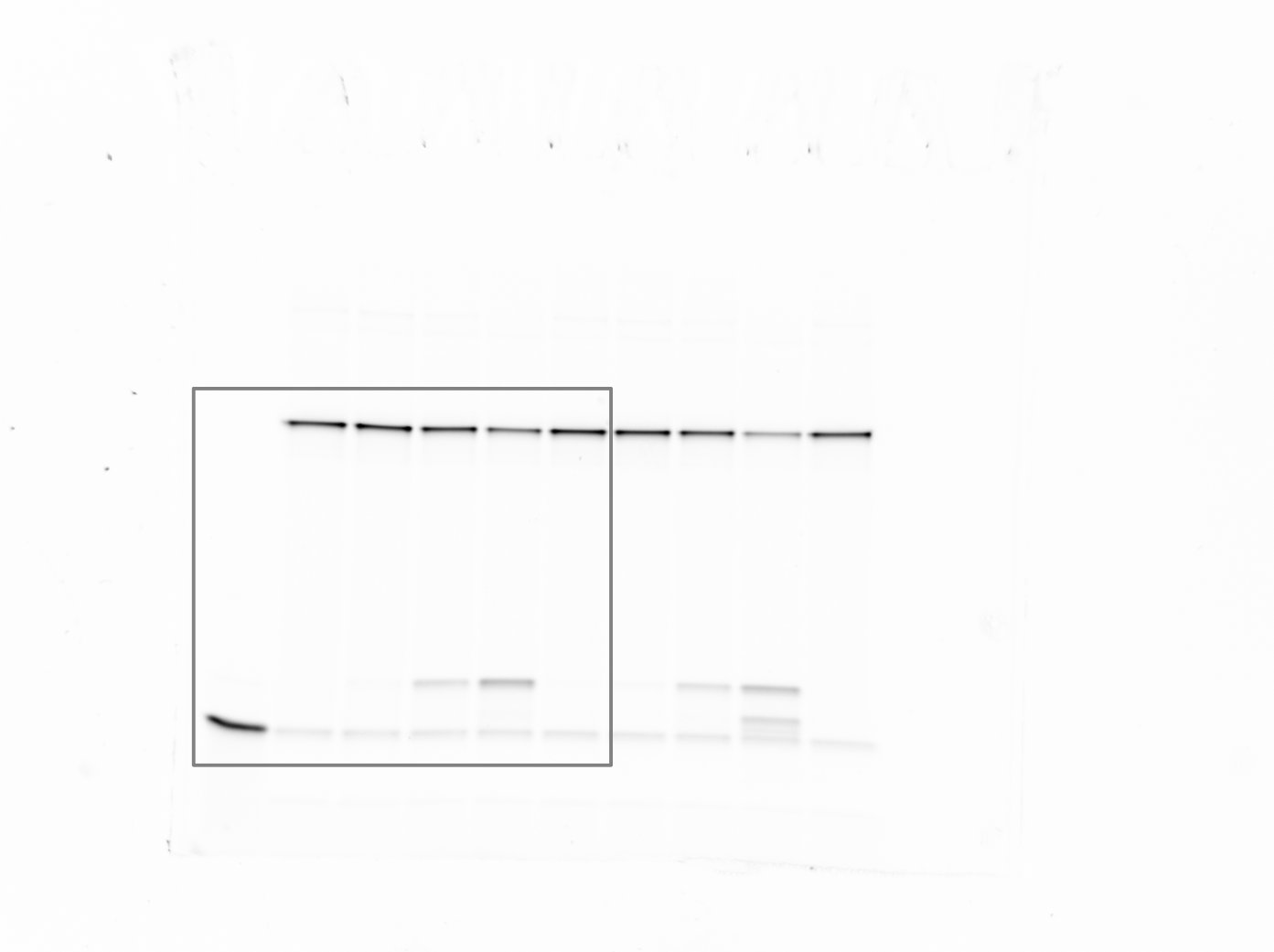

Supplement: Supplementary file 11 — Source Data [file 41467_2025_61224_MOESM11_ESM.zip › Source data/Uncropped scans of all blots and gels/Fig. 1/Fig. 1e/K48/Unmodified-SRAP_label_Cy5.tiff]

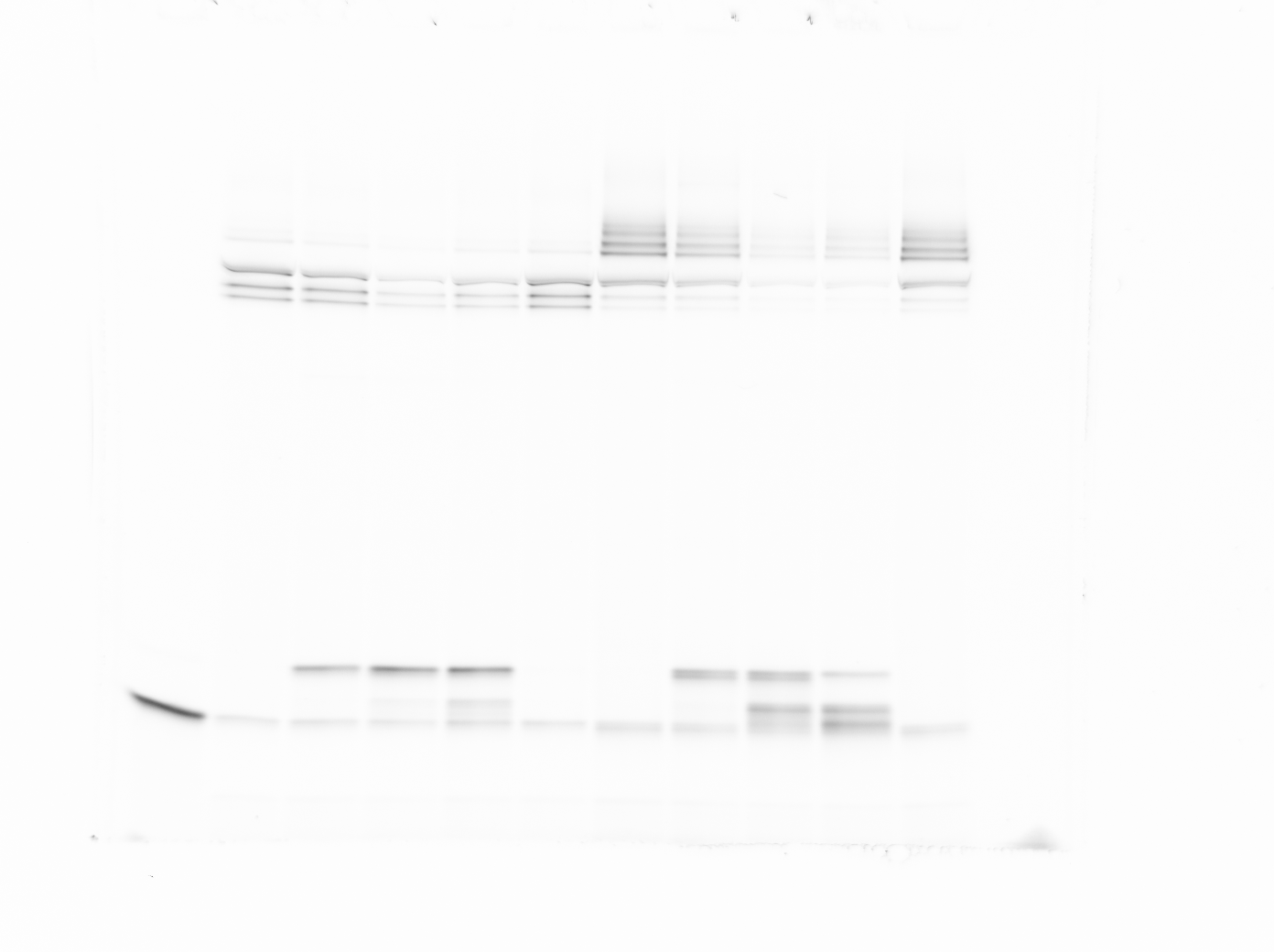

Supplement: Supplementary file 11 — Source Data [file 41467_2025_61224_MOESM11_ESM.zip › Source data/Uncropped scans of all blots and gels/Fig. 1/Fig. 1e/K63/K63-SRAP_Cy5.tif]

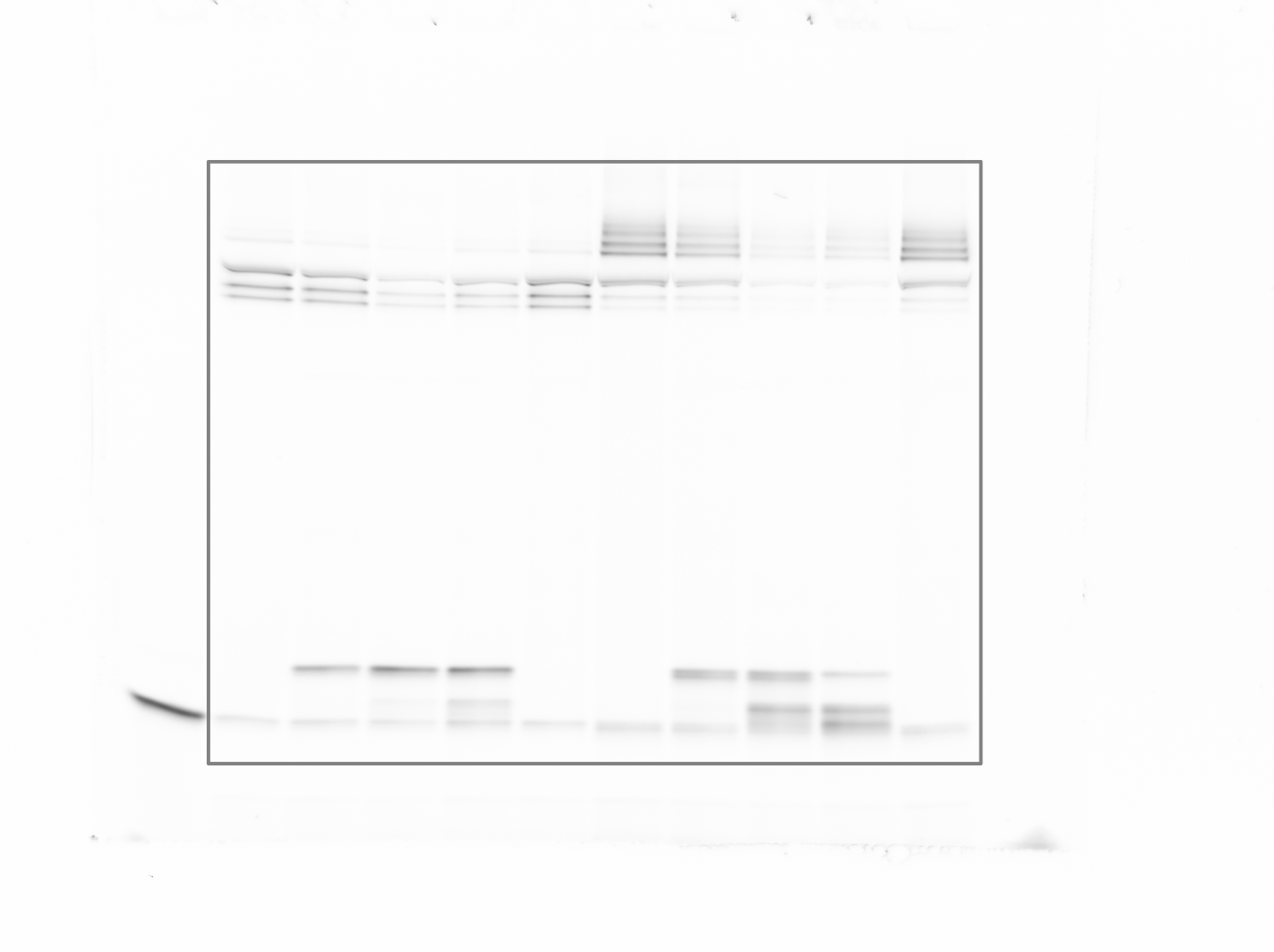

Supplement: Supplementary file 11 — Source Data [file 41467_2025_61224_MOESM11_ESM.zip › Source data/Uncropped scans of all blots and gels/Fig. 1/Fig. 1e/K63/K63-SRAP_label_Cy5.tiff]

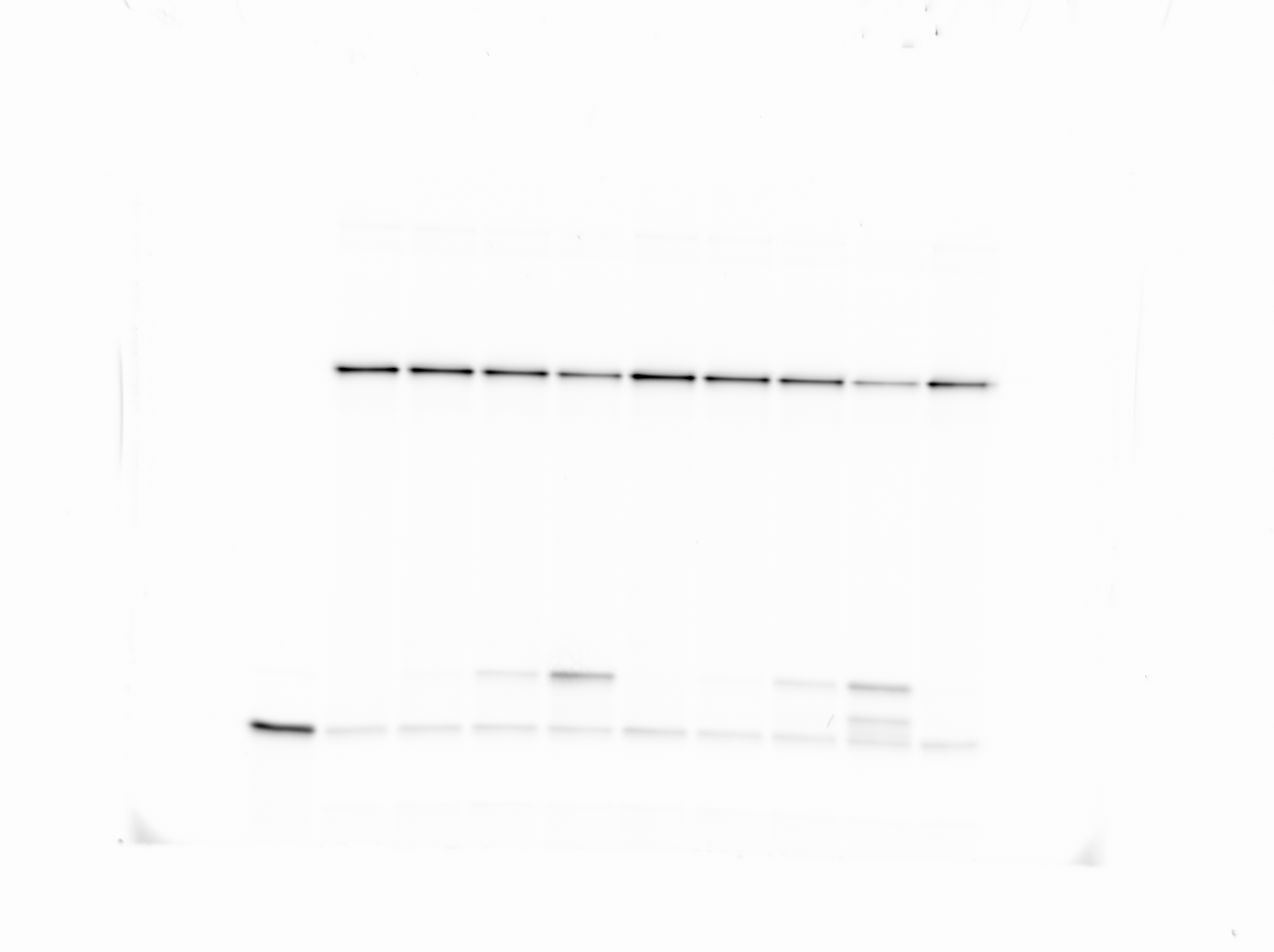

Supplement: Supplementary file 11 — Source Data [file 41467_2025_61224_MOESM11_ESM.zip › Source data/Uncropped scans of all blots and gels/Fig. 1/Fig. 1e/K63/Unmodified-SRAP_Cy5.tif]

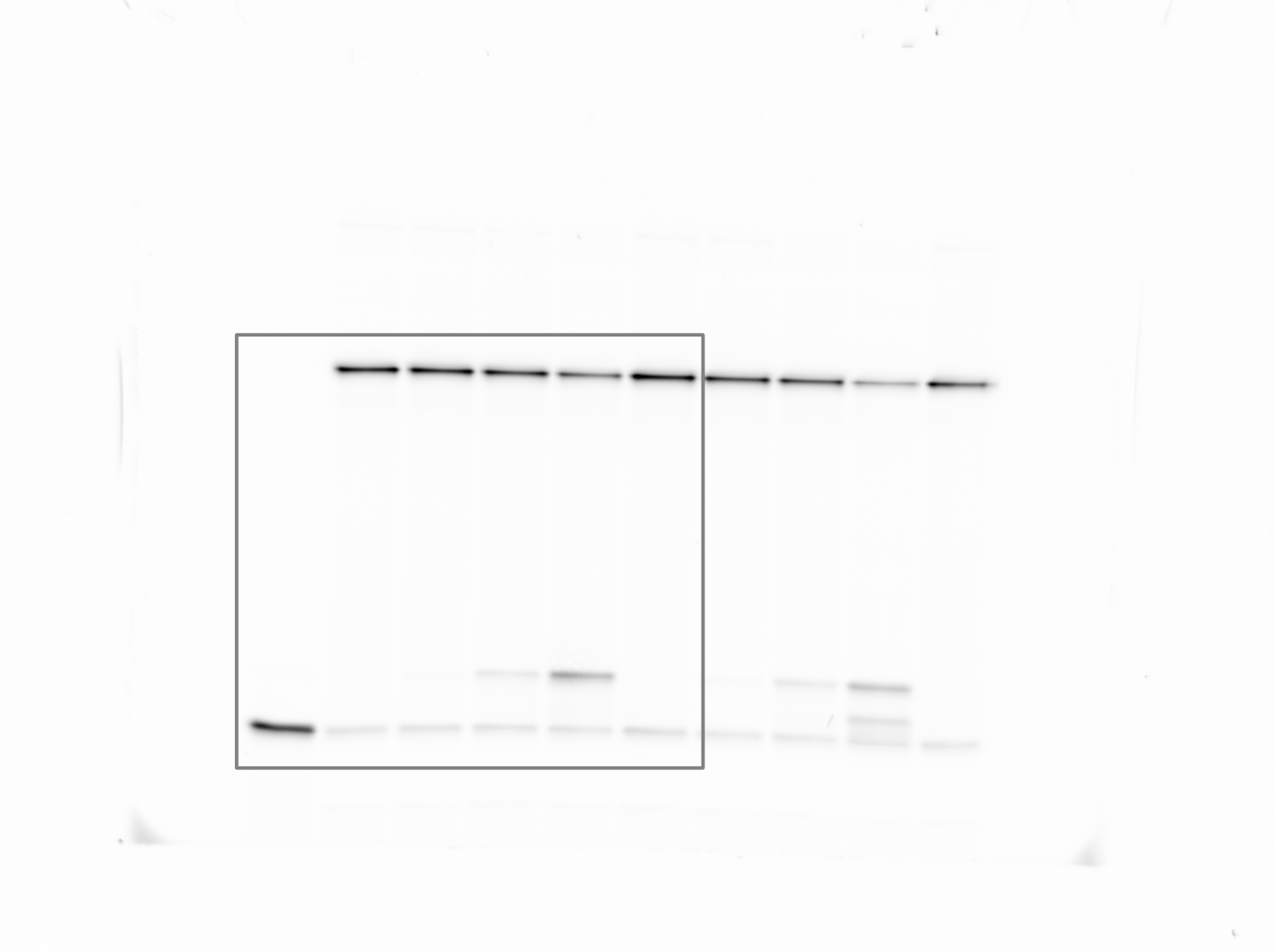

Supplement: Supplementary file 11 — Source Data [file 41467_2025_61224_MOESM11_ESM.zip › Source data/Uncropped scans of all blots and gels/Fig. 1/Fig. 1e/K63/Unmodified-SRAP_label_Cy5.tiff]

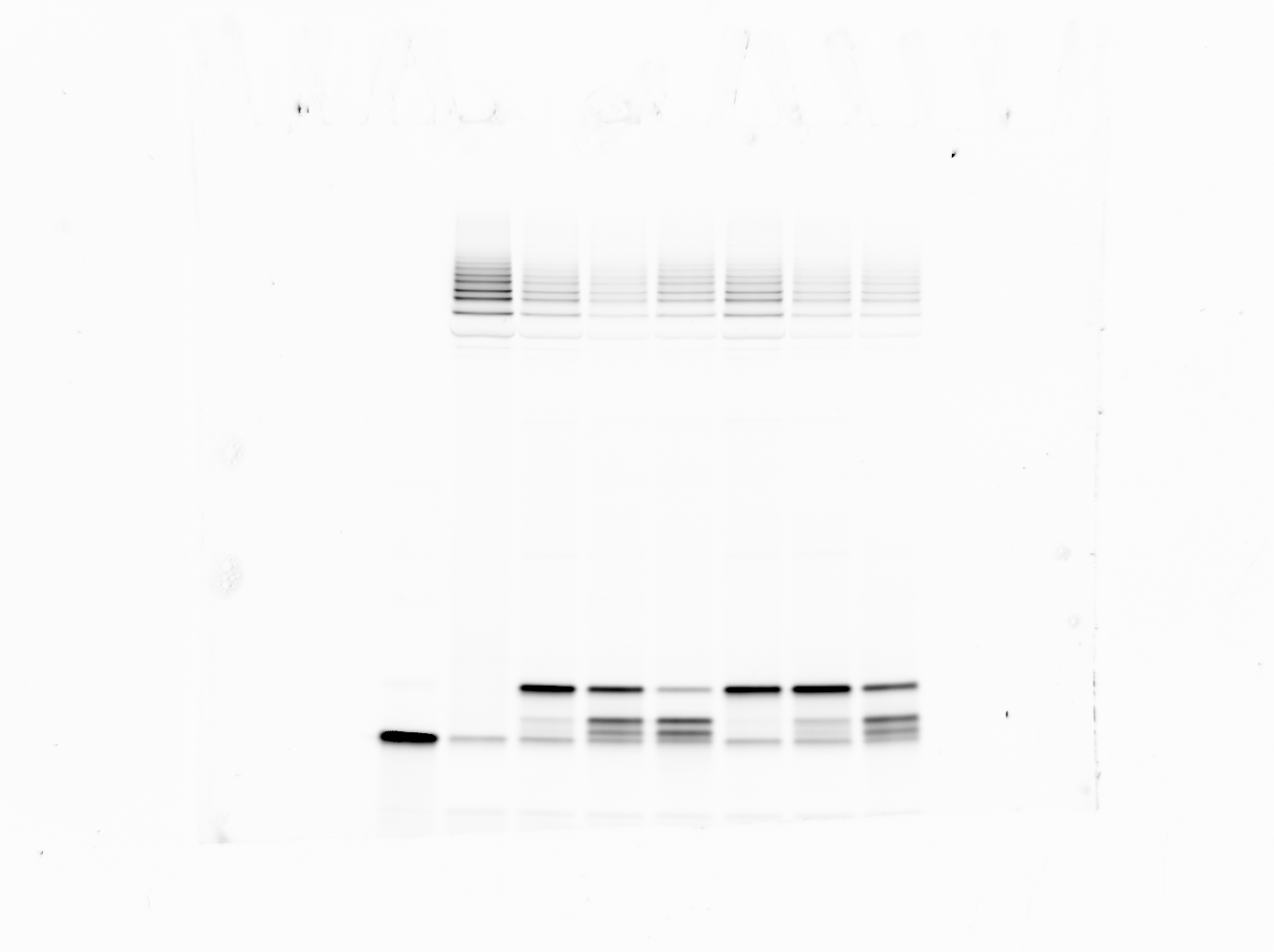

Supplement: Supplementary file 11 — Source Data [file 41467_2025_61224_MOESM11_ESM.zip › Source data/Uncropped scans of all blots and gels/Fig. 1/Fig. 1f/K48-SRAP_Cy5.tif]

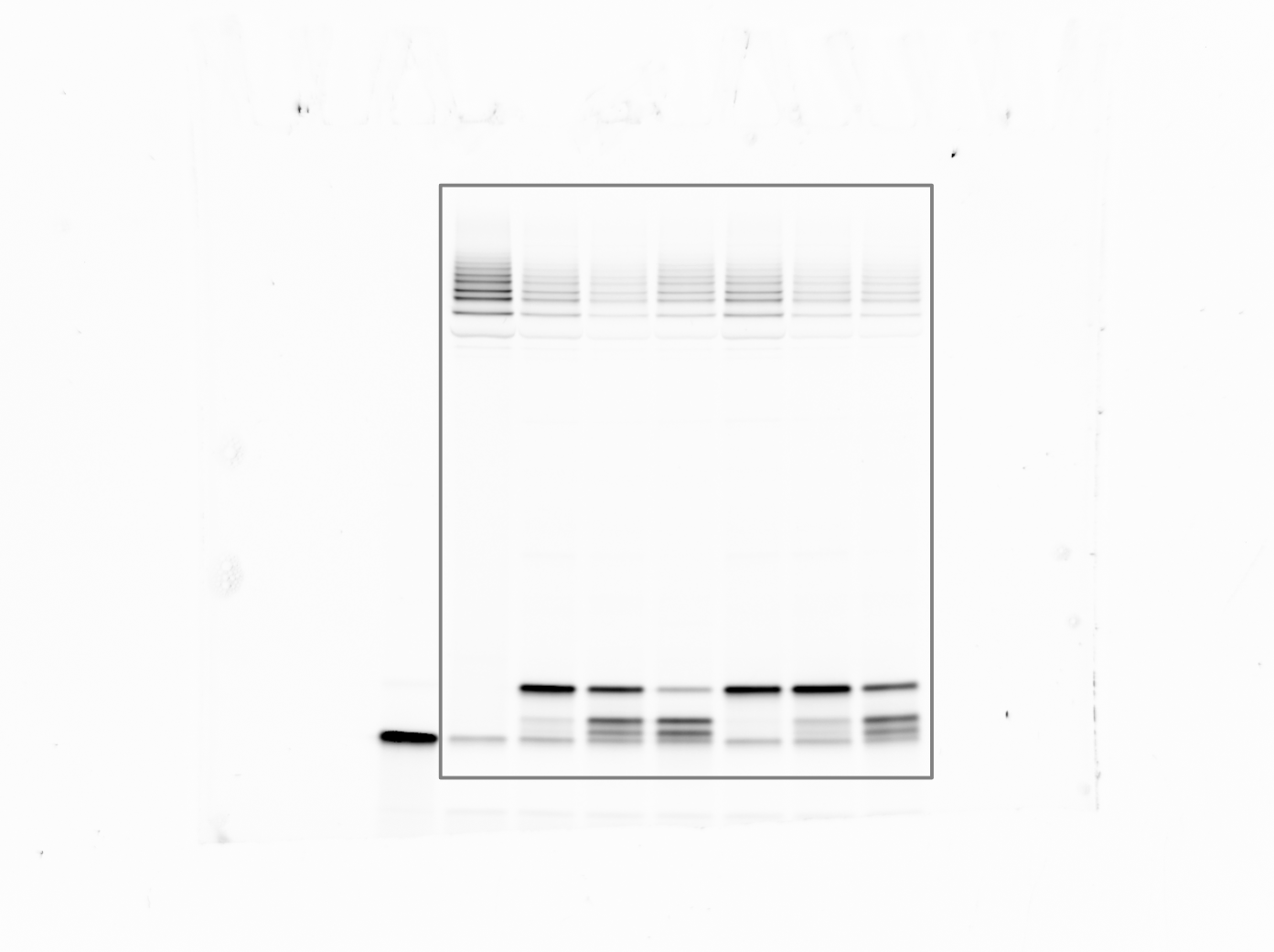

Supplement: Supplementary file 11 — Source Data [file 41467_2025_61224_MOESM11_ESM.zip › Source data/Uncropped scans of all blots and gels/Fig. 1/Fig. 1f/K48-SRAP_label_Cy5.tiff]

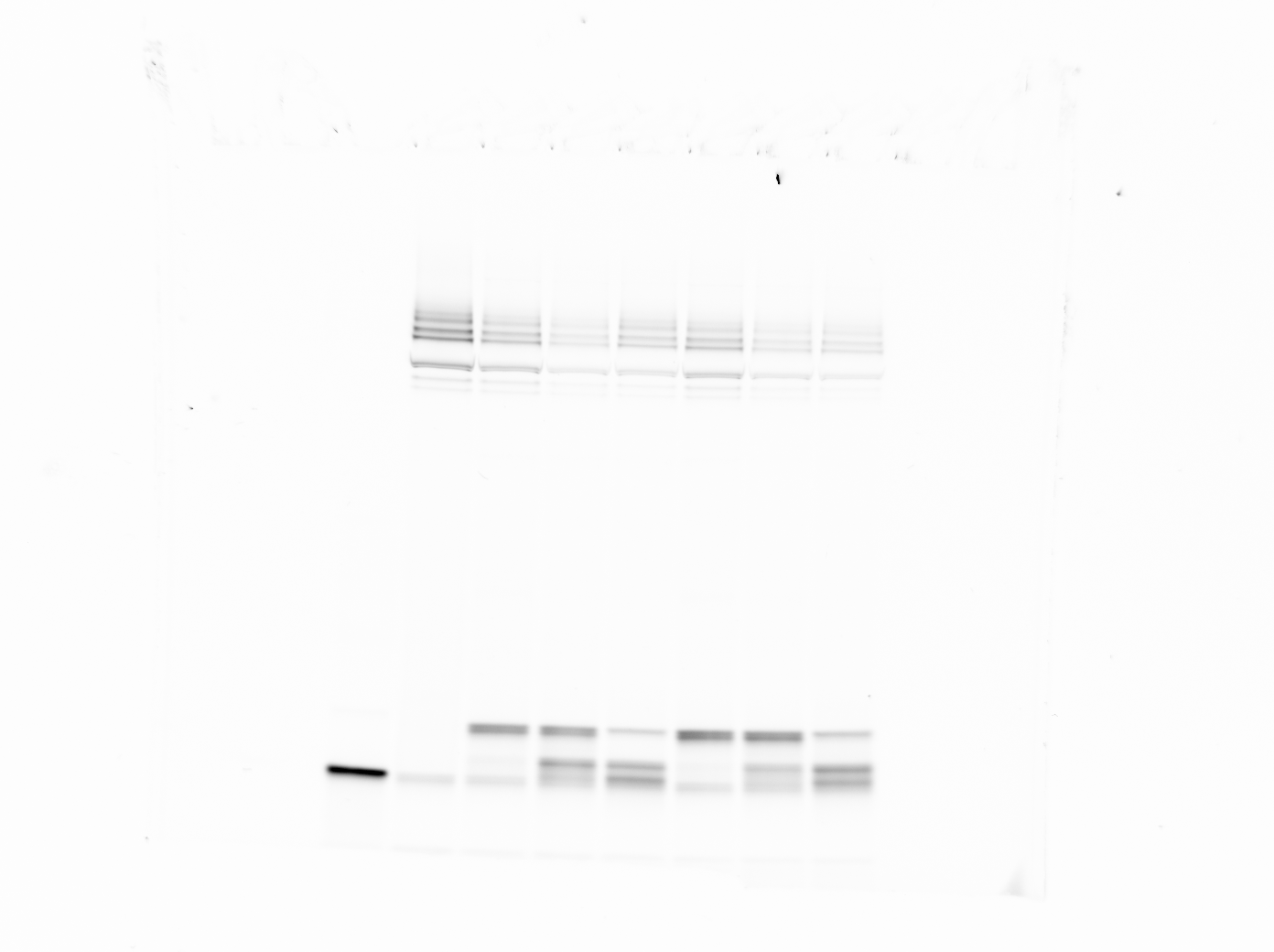

Supplement: Supplementary file 11 — Source Data [file 41467_2025_61224_MOESM11_ESM.zip › Source data/Uncropped scans of all blots and gels/Fig. 1/Fig. 1f/K63-SRAP_Cy5.tif]

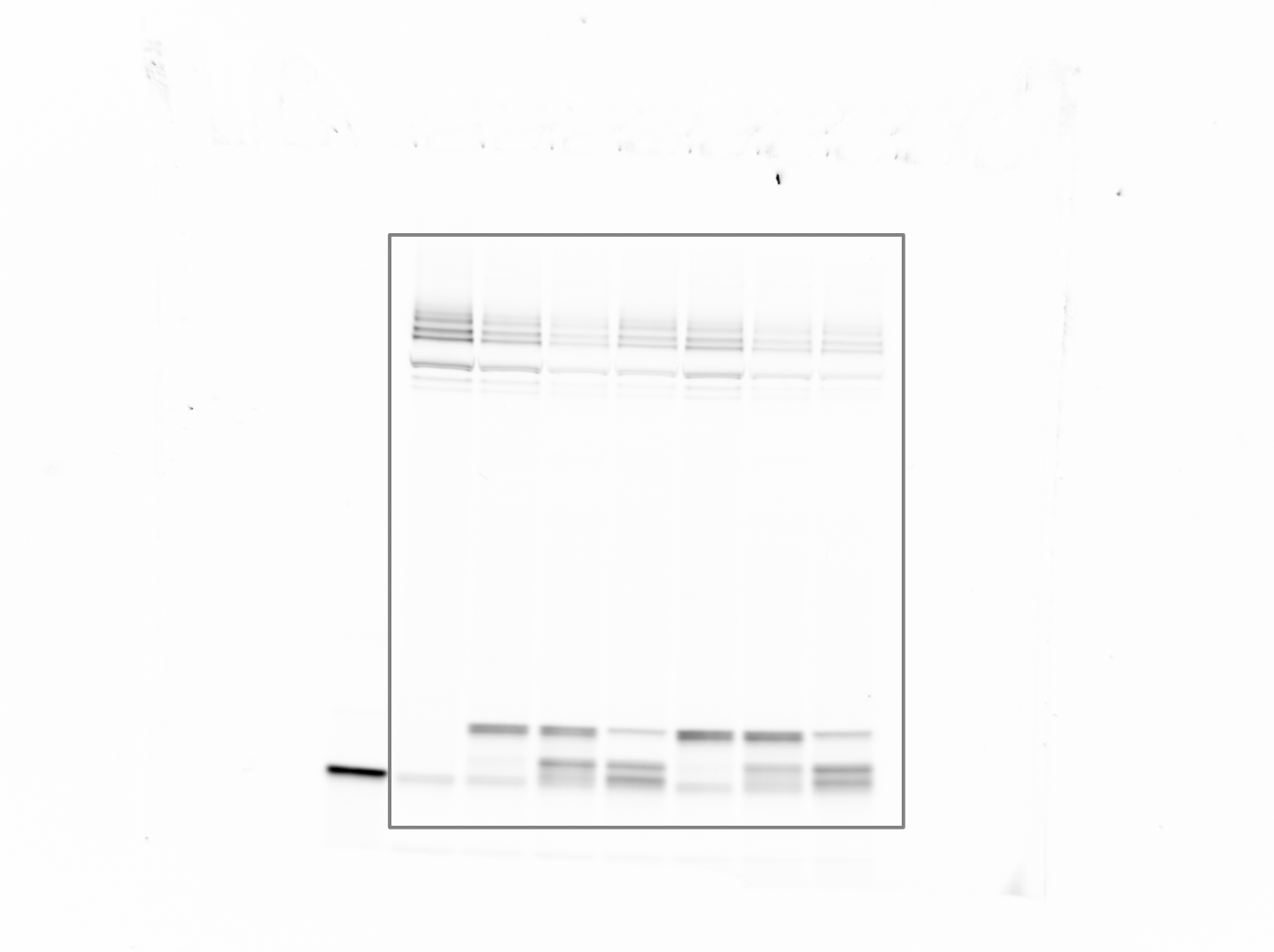

Supplement: Supplementary file 11 — Source Data [file 41467_2025_61224_MOESM11_ESM.zip › Source data/Uncropped scans of all blots and gels/Fig. 1/Fig. 1f/K63-SRAP_label_Cy5.tiff]

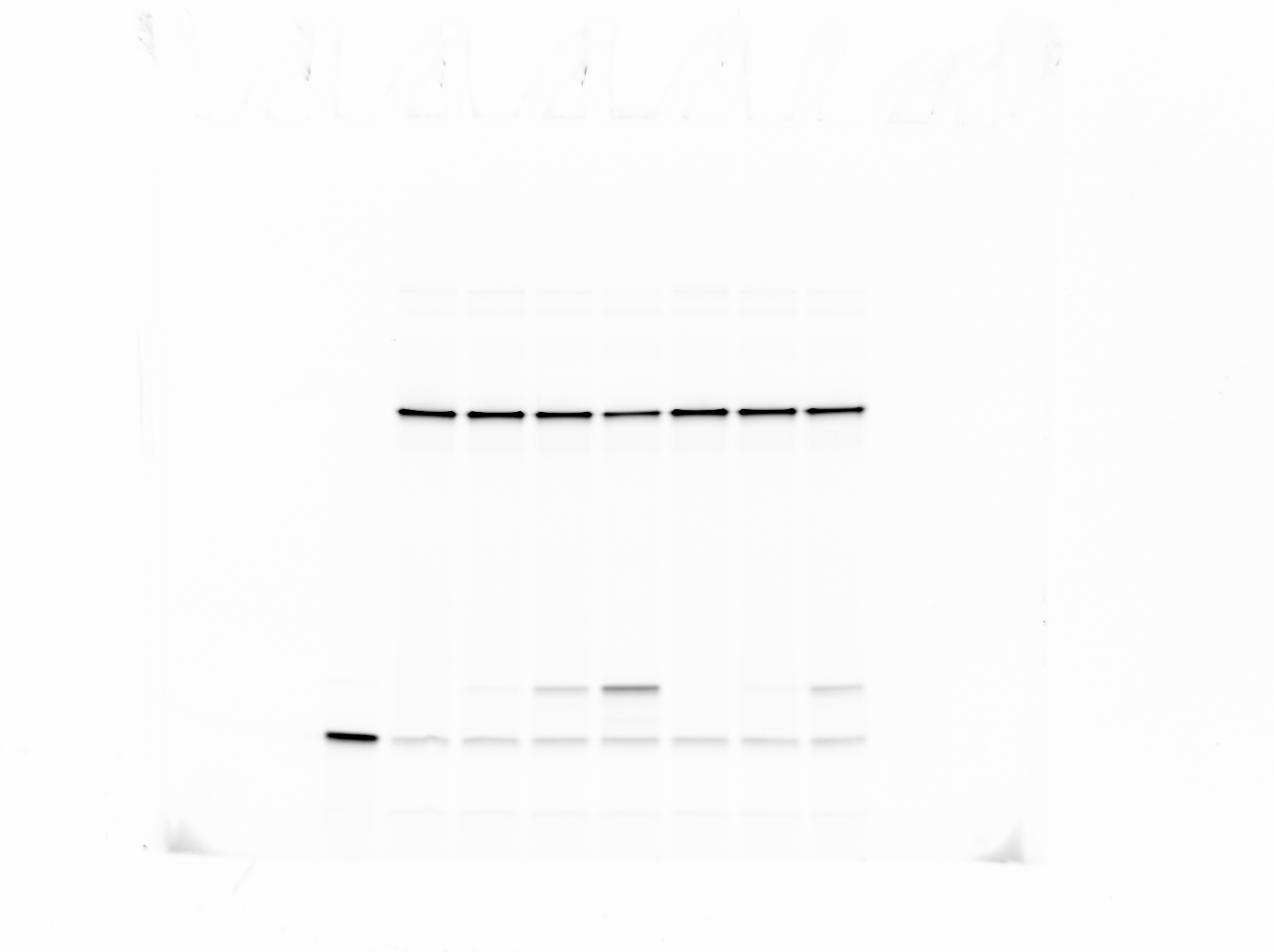

Supplement: Supplementary file 11 — Source Data [file 41467_2025_61224_MOESM11_ESM.zip › Source data/Uncropped scans of all blots and gels/Fig. 1/Fig. 1f/Unmodified-SRAP_Cy5.tif]

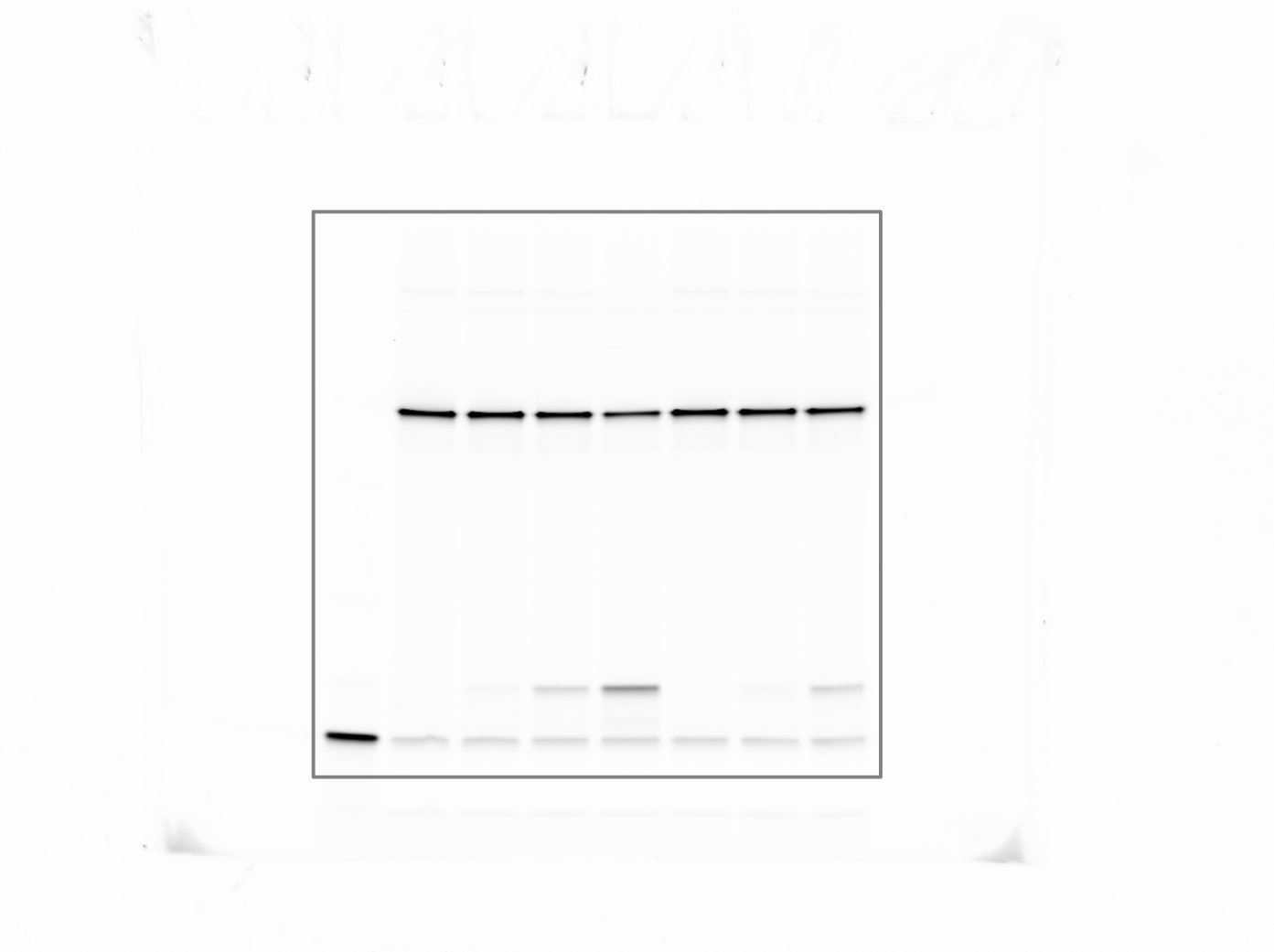

Supplement: Supplementary file 11 — Source Data [file 41467_2025_61224_MOESM11_ESM.zip › Source data/Uncropped scans of all blots and gels/Fig. 1/Fig. 1f/Unmodified-SRAP_label_Cy5.tiff]

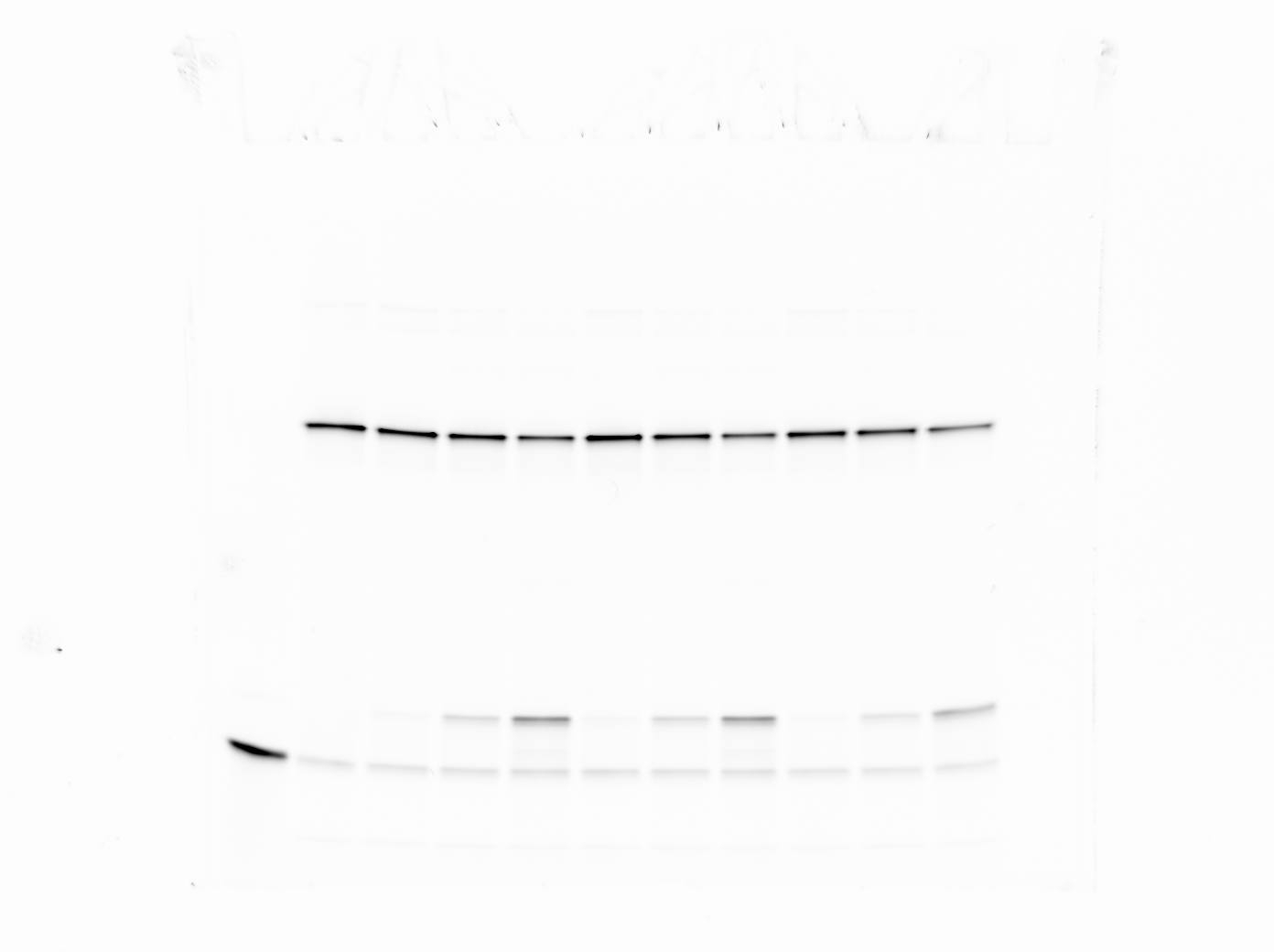

Supplement: Supplementary file 11 — Source Data [file 41467_2025_61224_MOESM11_ESM.zip › Source data/Uncropped scans of all blots and gels/Fig. 4/Fig. 4a/Cy5.tif]

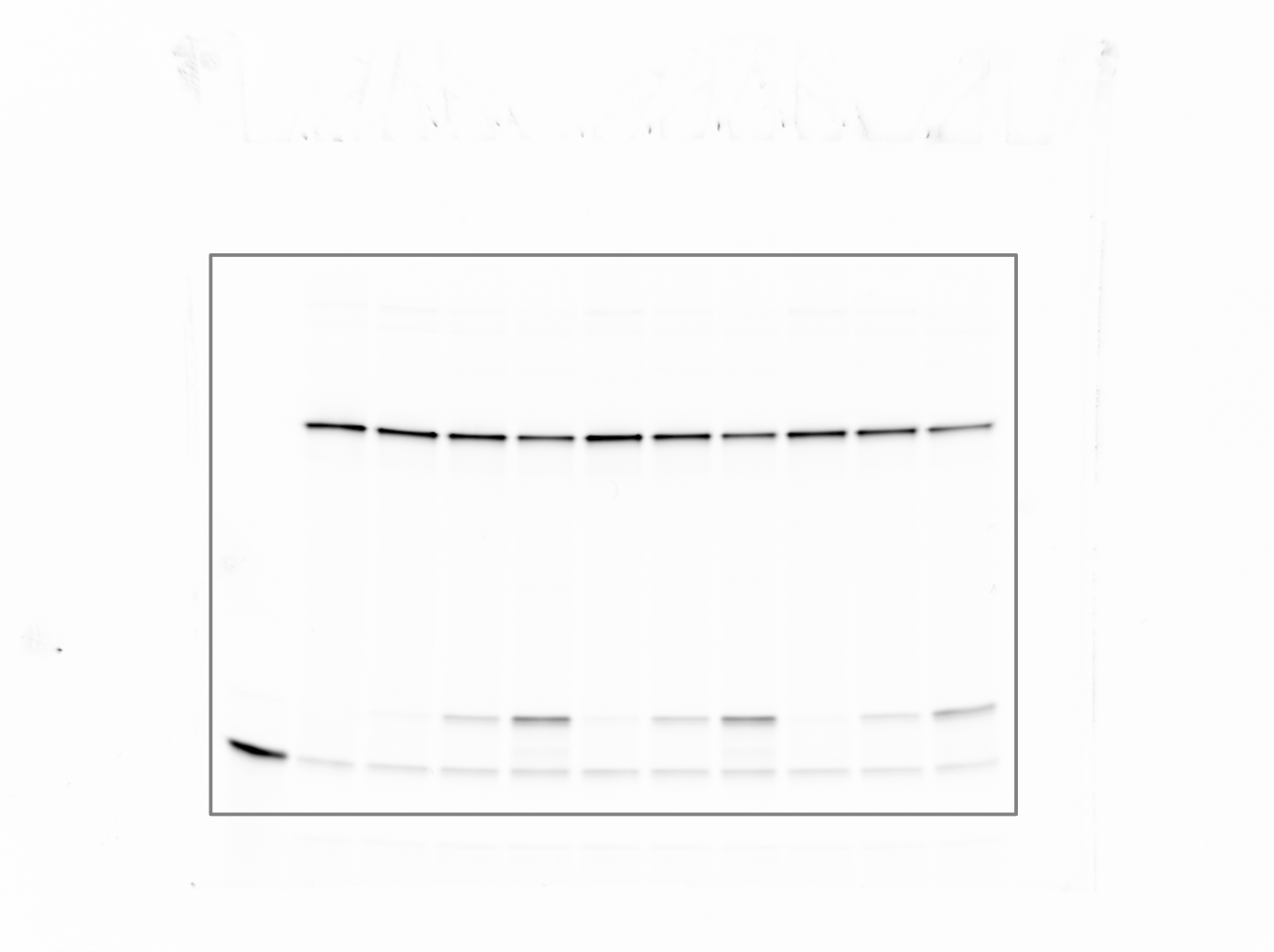

Supplement: Supplementary file 11 — Source Data [file 41467_2025_61224_MOESM11_ESM.zip › Source data/Uncropped scans of all blots and gels/Fig. 4/Fig. 4a/Cy5_label.tiff]

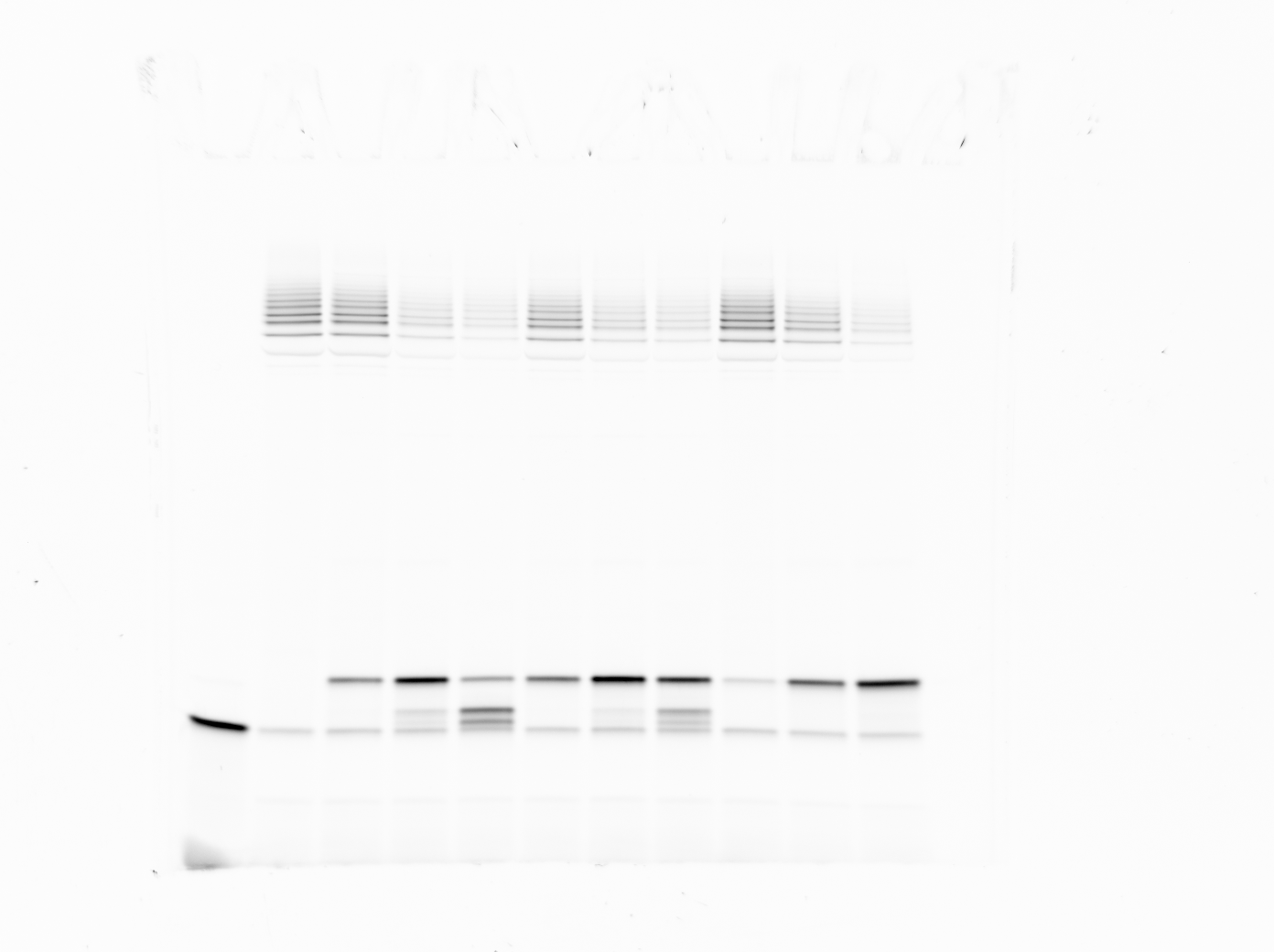

Supplement: Supplementary file 11 — Source Data [file 41467_2025_61224_MOESM11_ESM.zip › Source data/Uncropped scans of all blots and gels/Fig. 4/Fig. 4b/Cy5.tif]

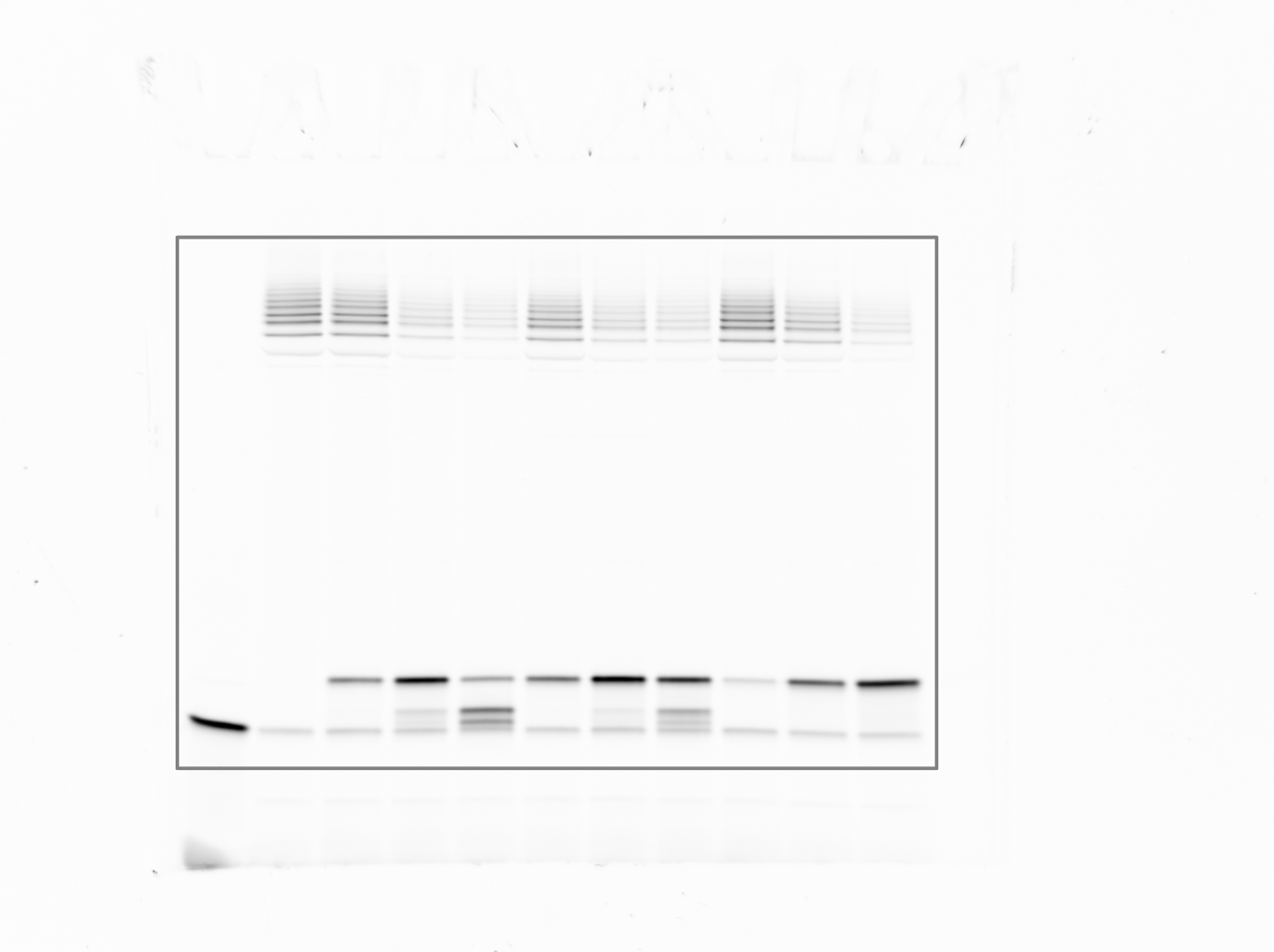

Supplement: Supplementary file 11 — Source Data [file 41467_2025_61224_MOESM11_ESM.zip › Source data/Uncropped scans of all blots and gels/Fig. 4/Fig. 4b/Cy5_label.tiff]

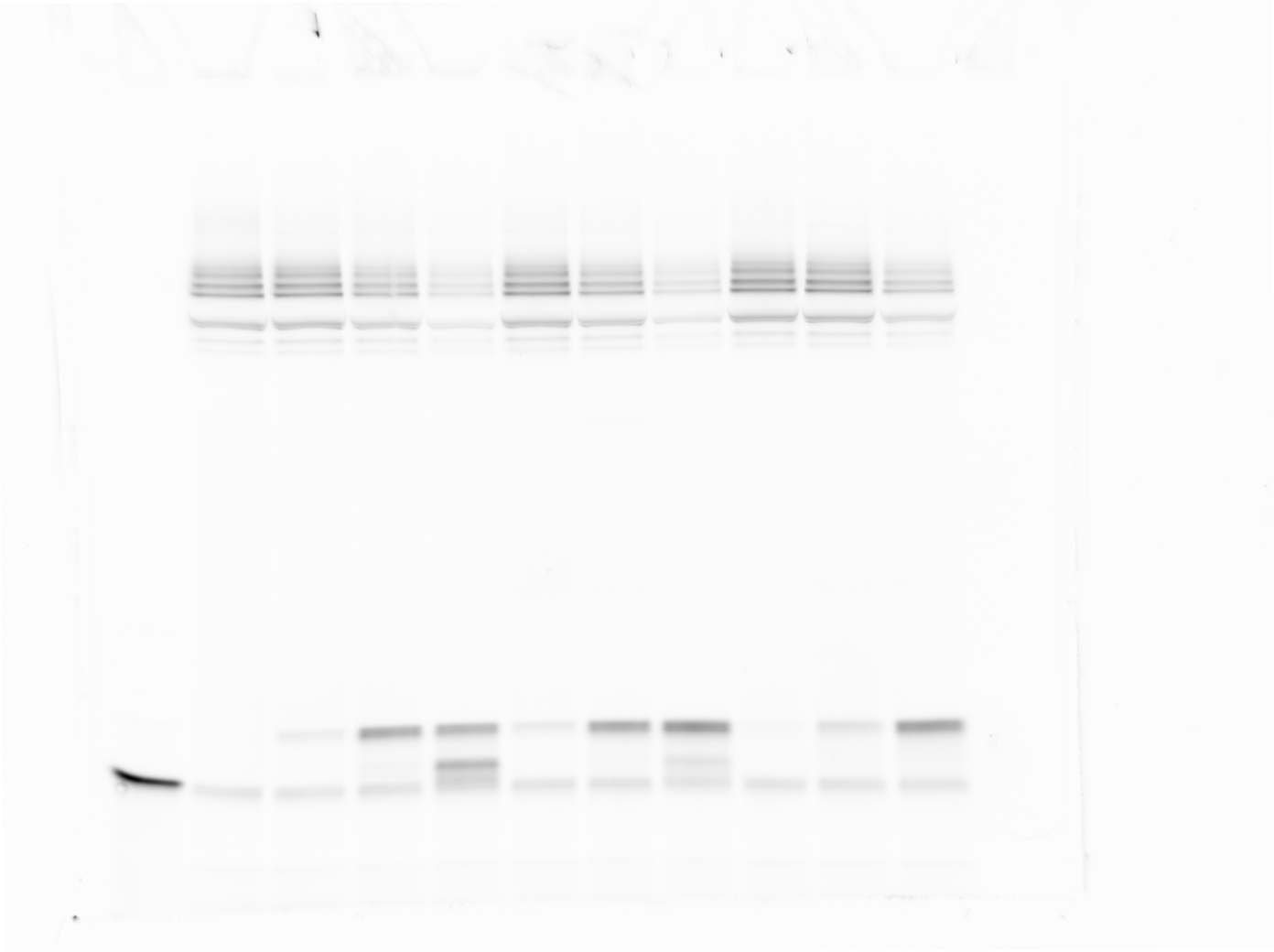

Supplement: Supplementary file 11 — Source Data [file 41467_2025_61224_MOESM11_ESM.zip › Source data/Uncropped scans of all blots and gels/Fig. 4/Fig. 4c/Cy5.tif]

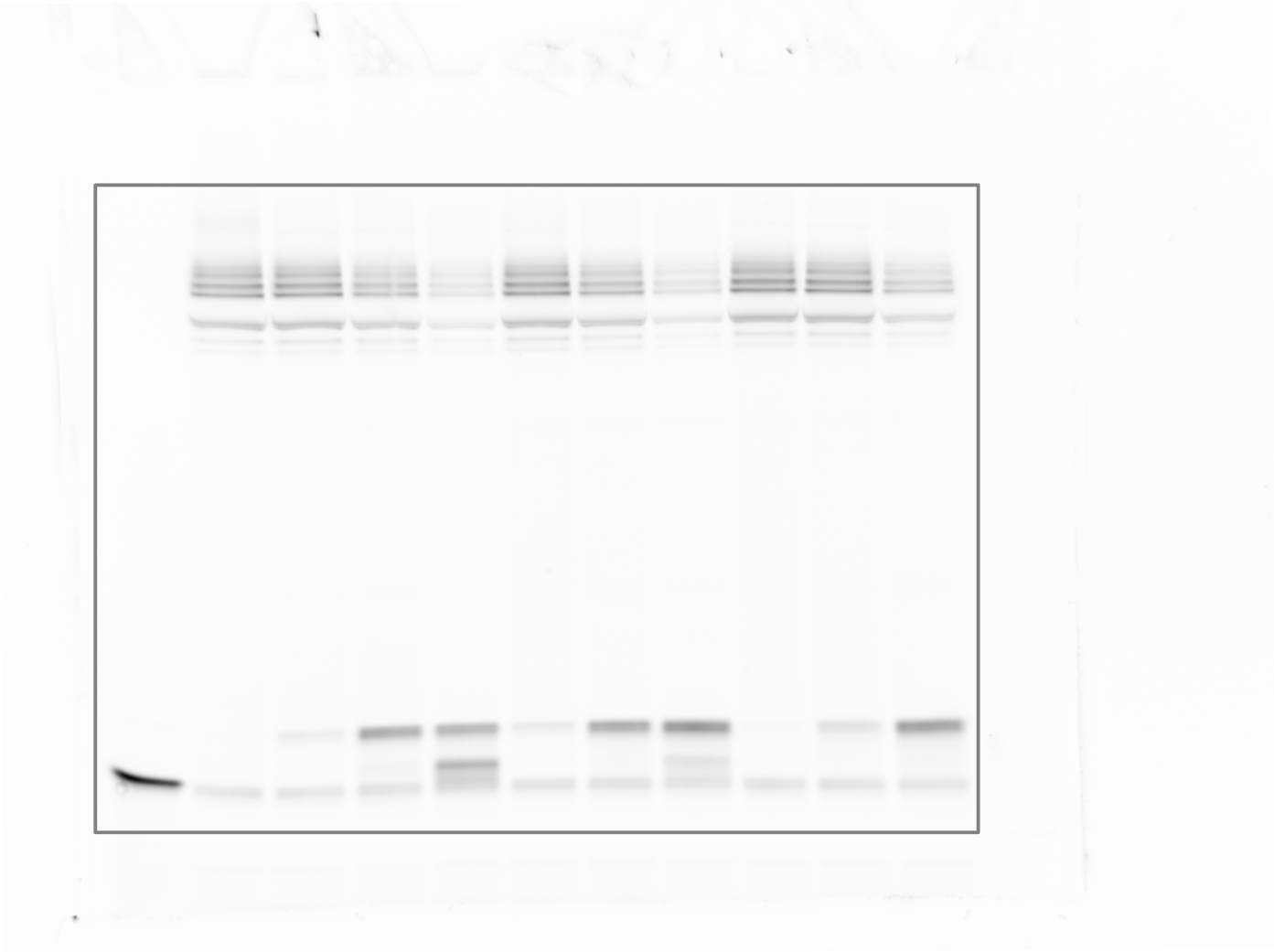

Supplement: Supplementary file 11 — Source Data [file 41467_2025_61224_MOESM11_ESM.zip › Source data/Uncropped scans of all blots and gels/Fig. 4/Fig. 4c/Cy5_label.tiff]

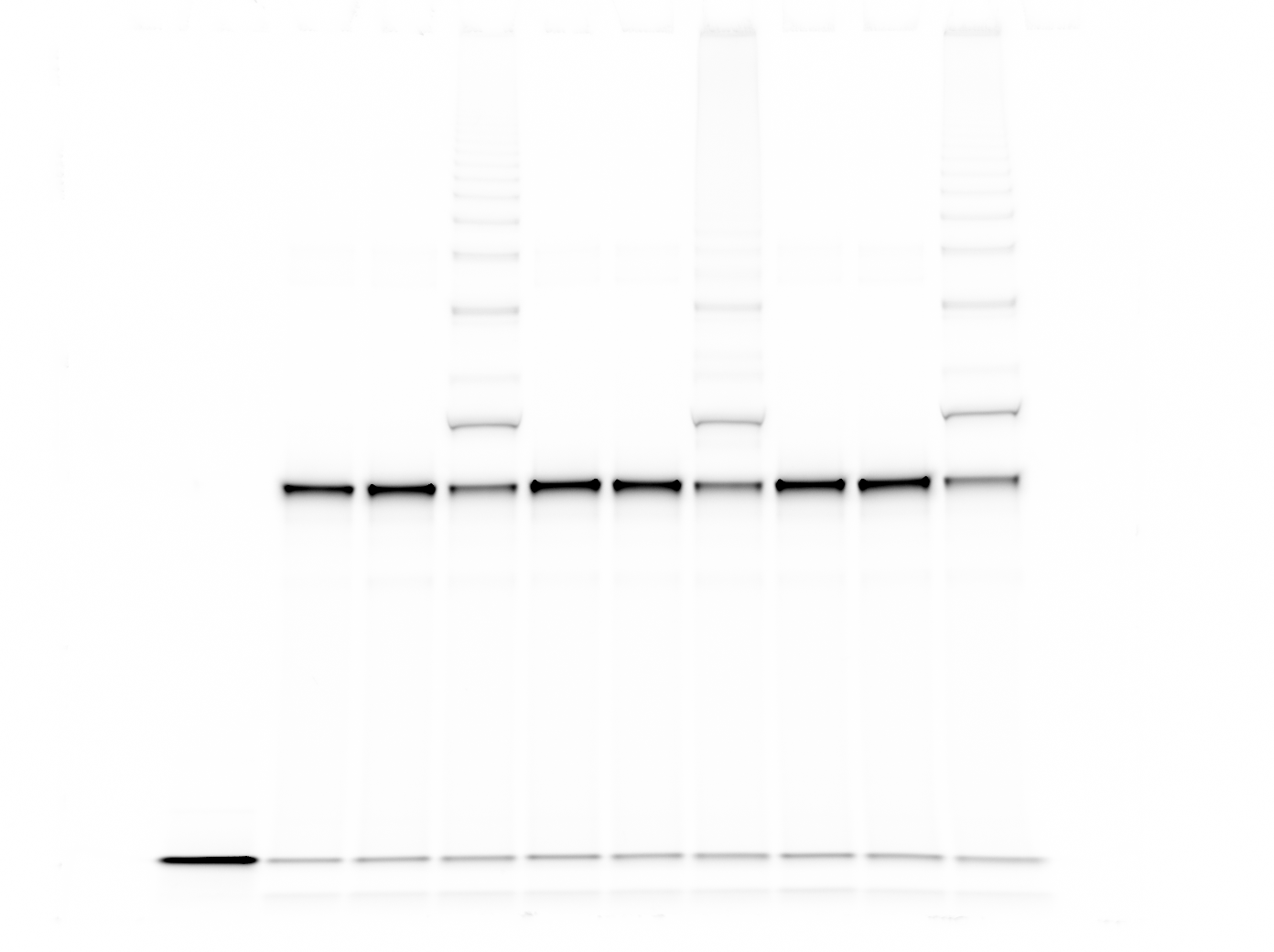

Supplement: Supplementary file 11 — Source Data [file 41467_2025_61224_MOESM11_ESM.zip › Source data/Uncropped scans of all blots and gels/Fig. 5/Fig. 5b/Cy5.tif]

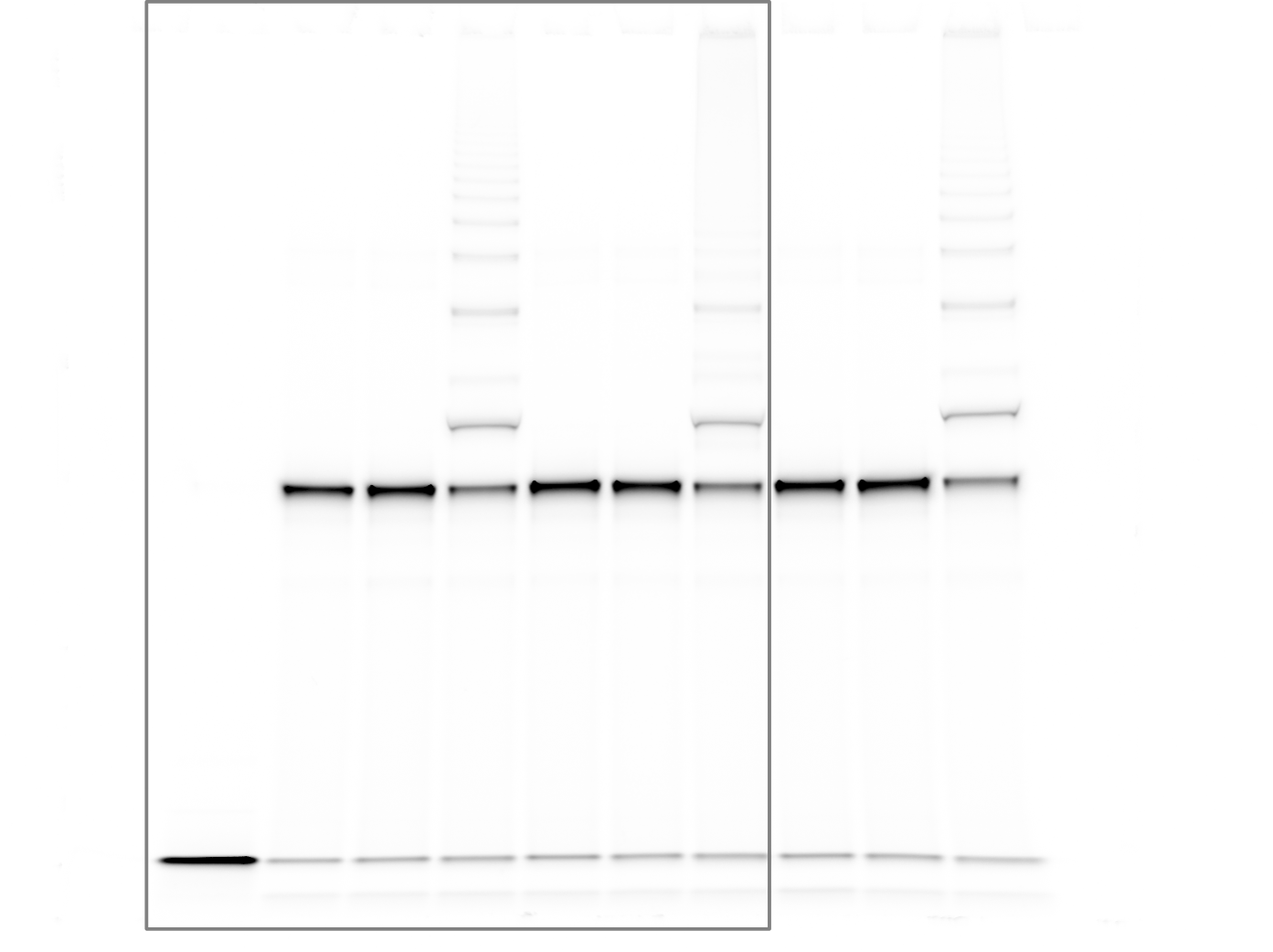

Supplement: Supplementary file 11 — Source Data [file 41467_2025_61224_MOESM11_ESM.zip › Source data/Uncropped scans of all blots and gels/Fig. 5/Fig. 5b/Cy5_label.tiff]

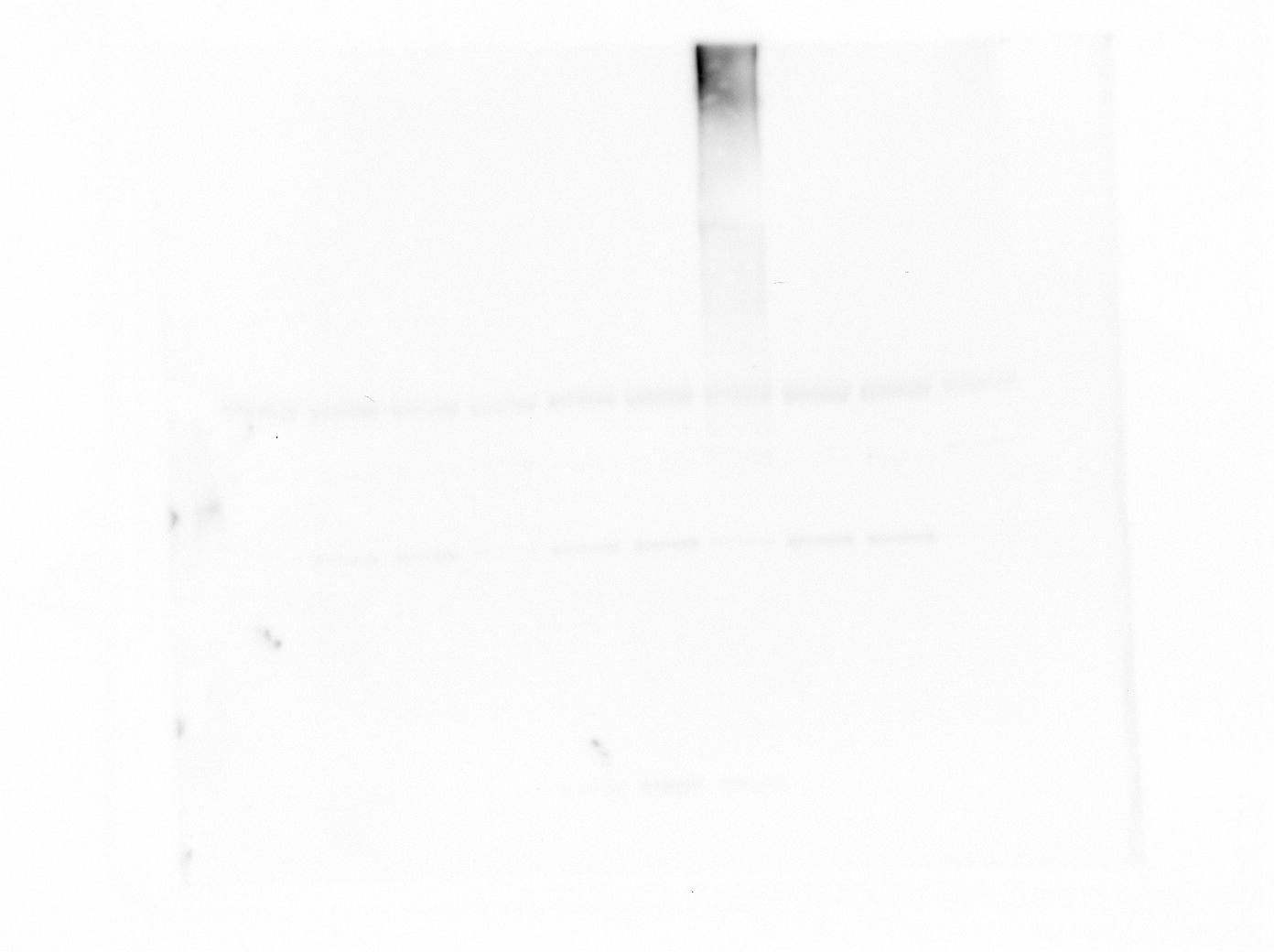

Supplement: Supplementary file 11 — Source Data [file 41467_2025_61224_MOESM11_ESM.zip › Source data/Uncropped scans of all blots and gels/Fig. 5/Fig. 5b/K48/K48.tif]

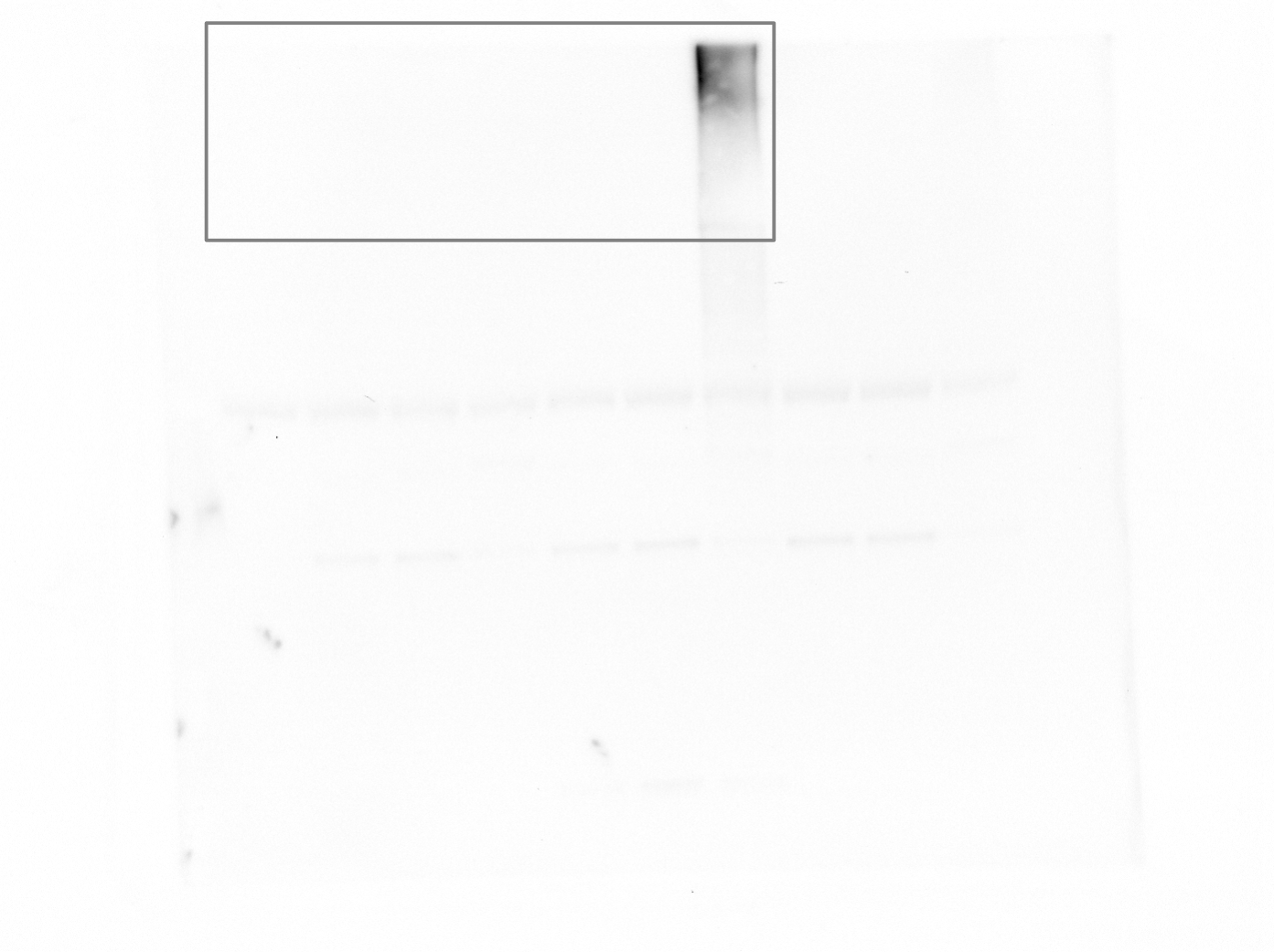

Supplement: Supplementary file 11 — Source Data [file 41467_2025_61224_MOESM11_ESM.zip › Source data/Uncropped scans of all blots and gels/Fig. 5/Fig. 5b/K48/K48_label.tiff]

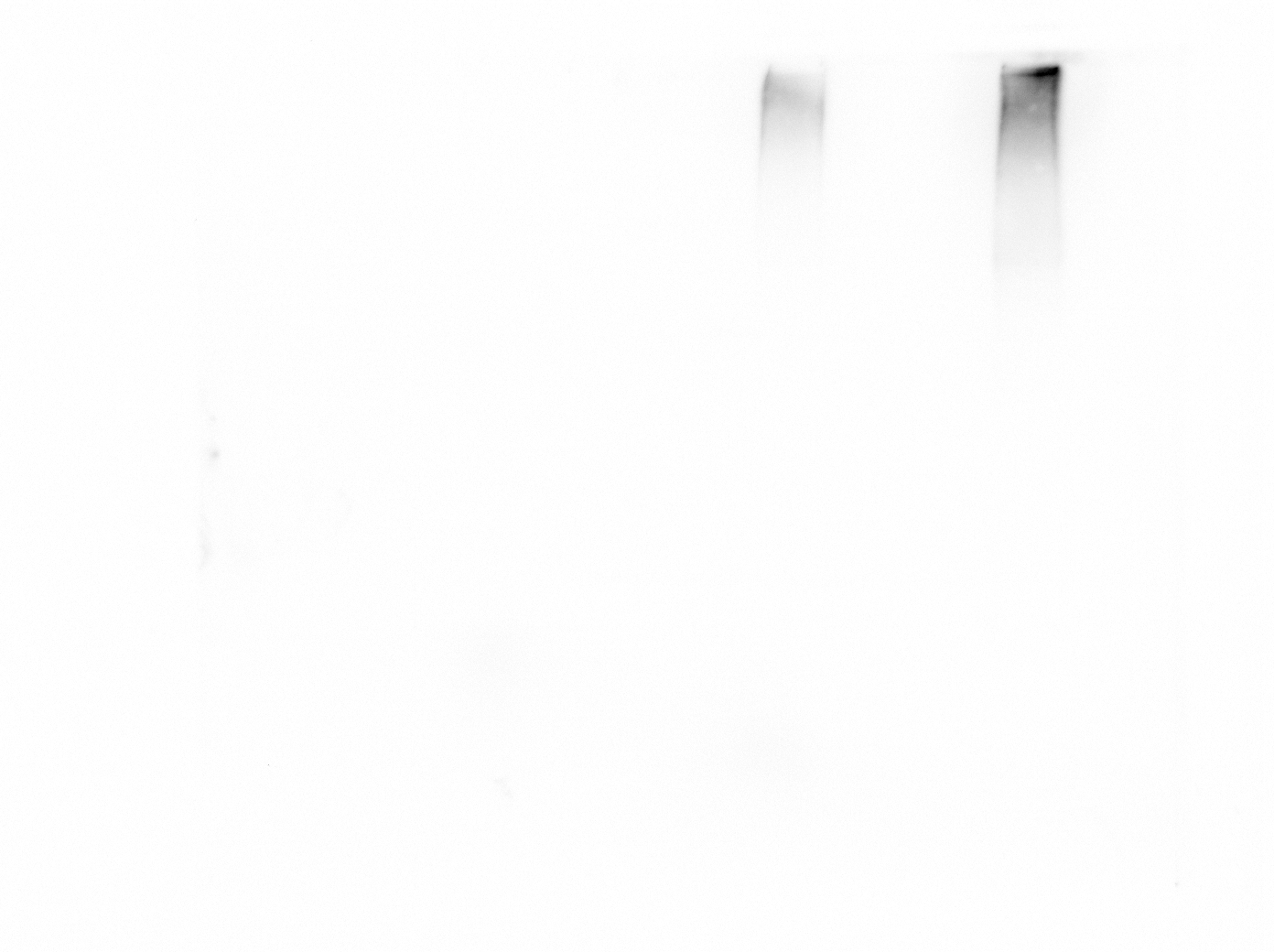

Supplement: Supplementary file 11 — Source Data [file 41467_2025_61224_MOESM11_ESM.zip › Source data/Uncropped scans of all blots and gels/Fig. 5/Fig. 5b/K63/K63.tif]

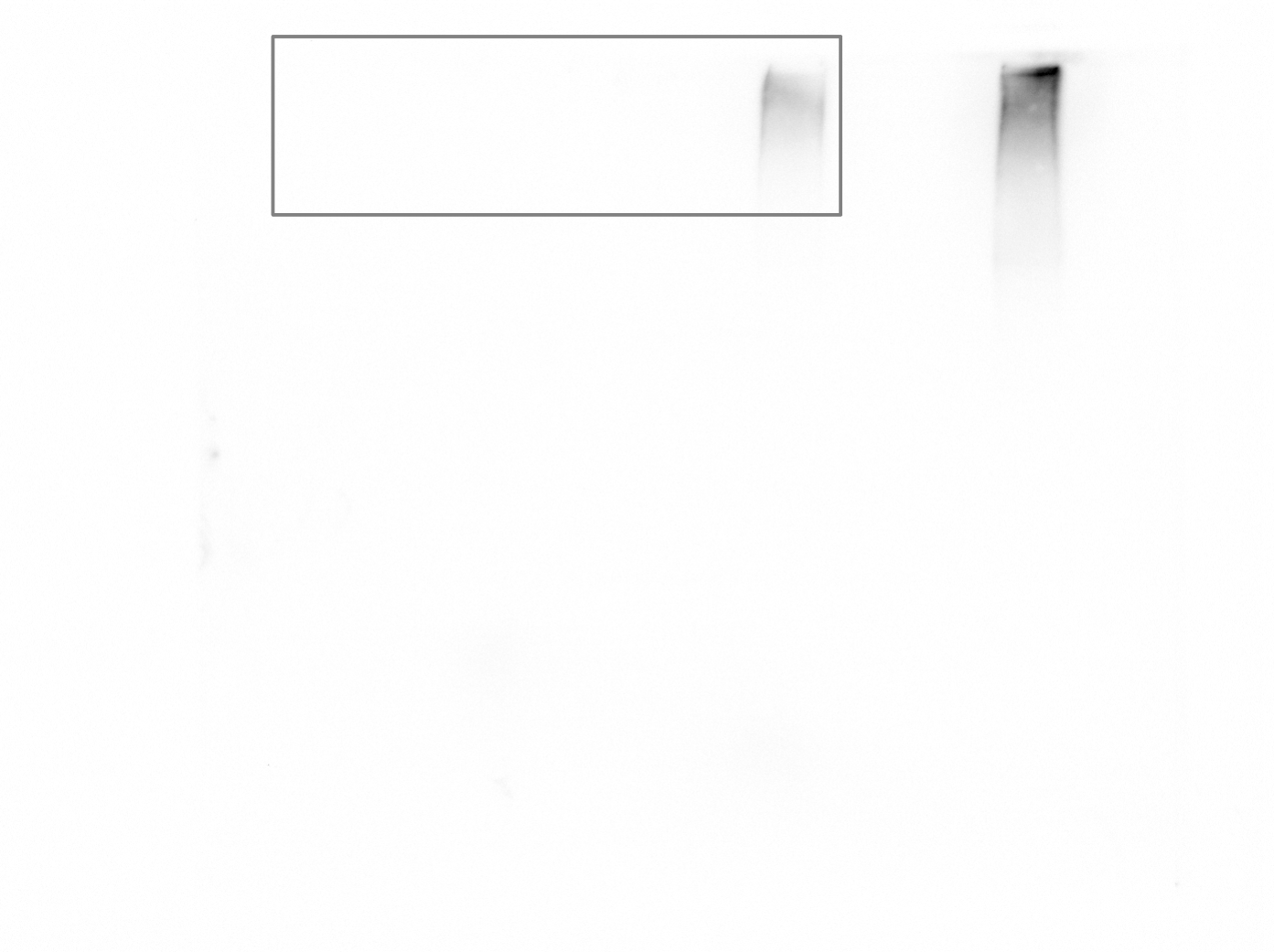

Supplement: Supplementary file 11 — Source Data [file 41467_2025_61224_MOESM11_ESM.zip › Source data/Uncropped scans of all blots and gels/Fig. 5/Fig. 5b/K63/K63_label.tiff]

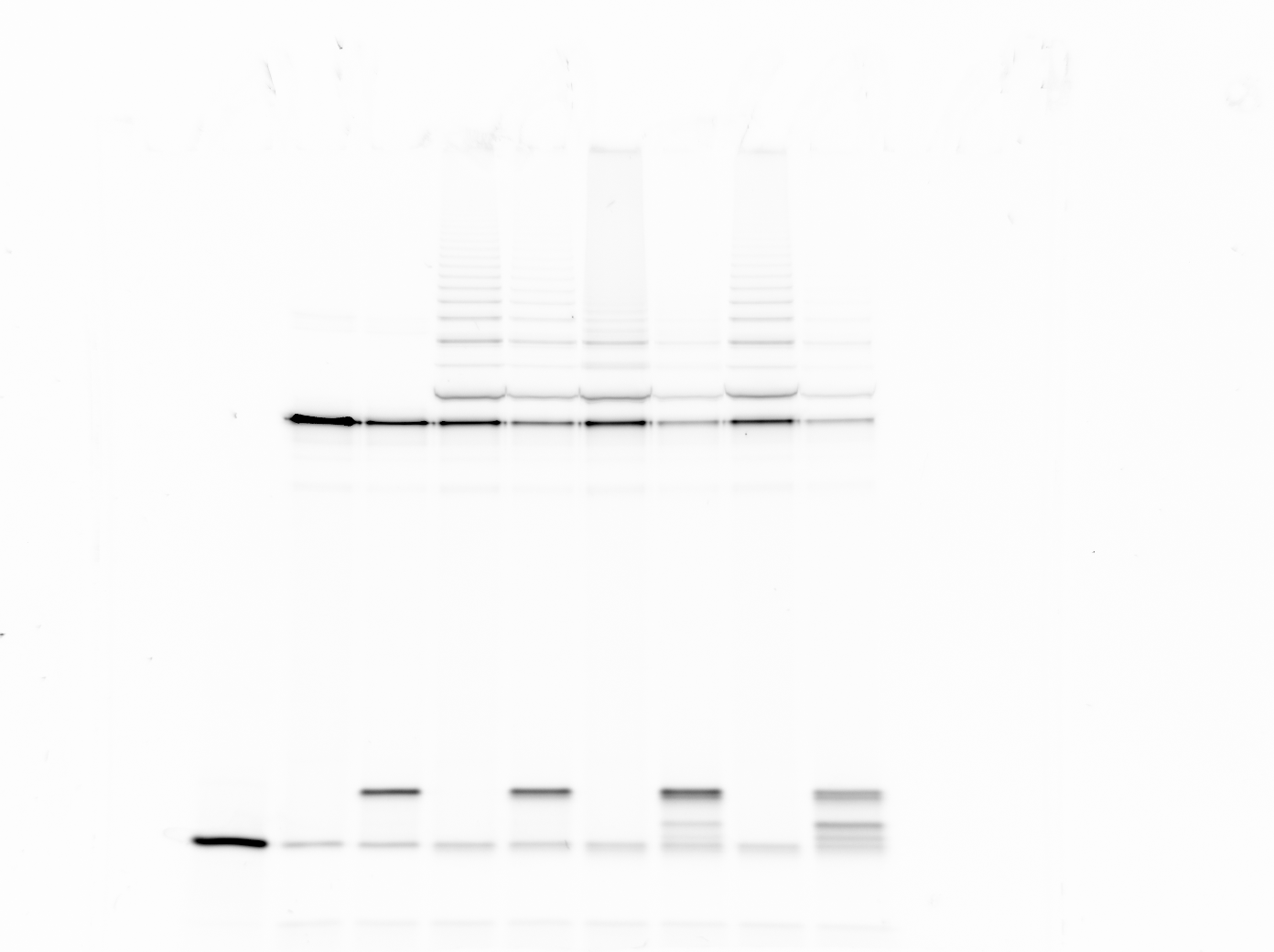

Supplement: Supplementary file 11 — Source Data [file 41467_2025_61224_MOESM11_ESM.zip › Source data/Uncropped scans of all blots and gels/Fig. 5/Fig. 5e/Cy5.tif]

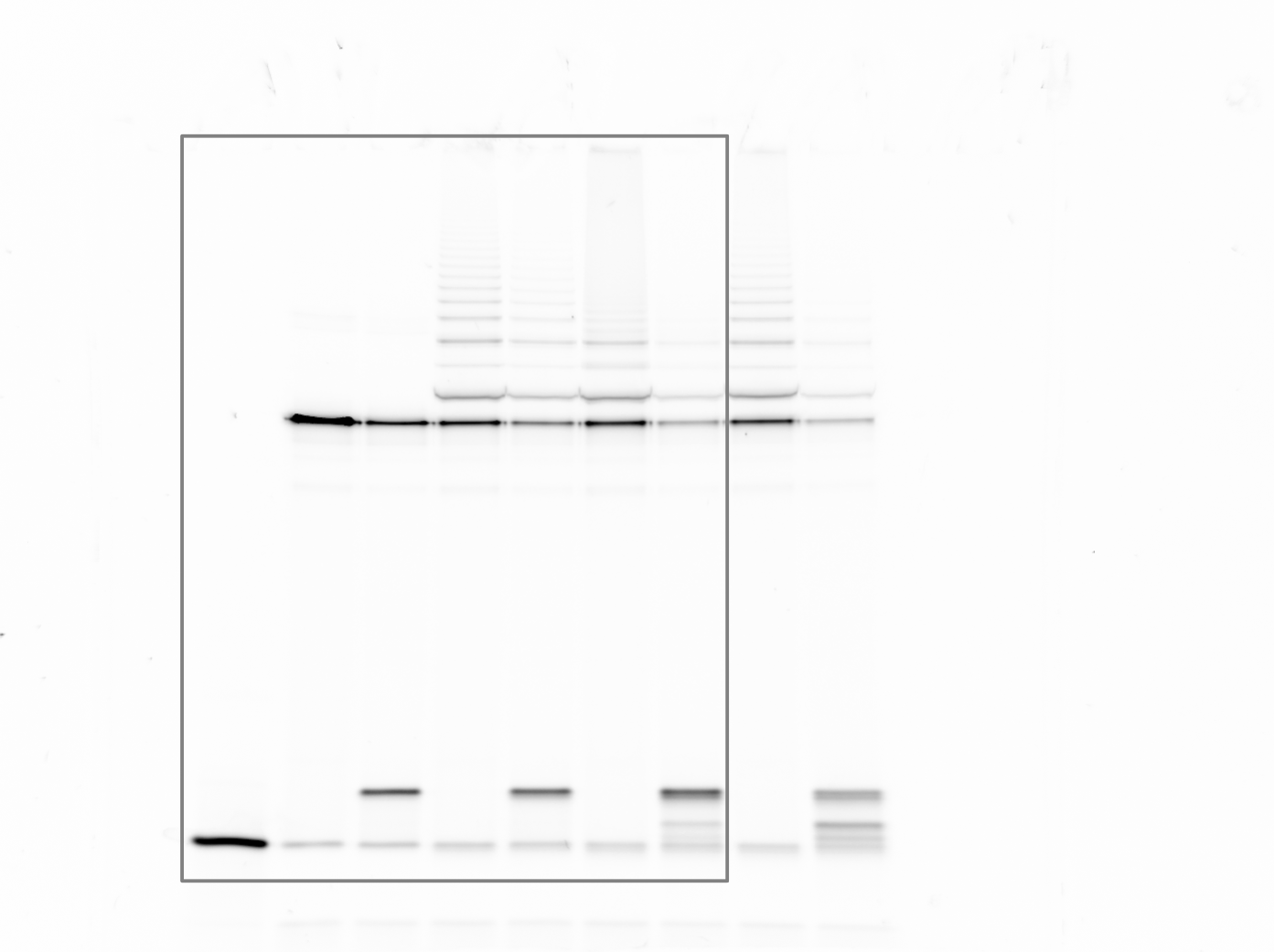

Supplement: Supplementary file 11 — Source Data [file 41467_2025_61224_MOESM11_ESM.zip › Source data/Uncropped scans of all blots and gels/Fig. 5/Fig. 5e/Cy5_label.tiff]

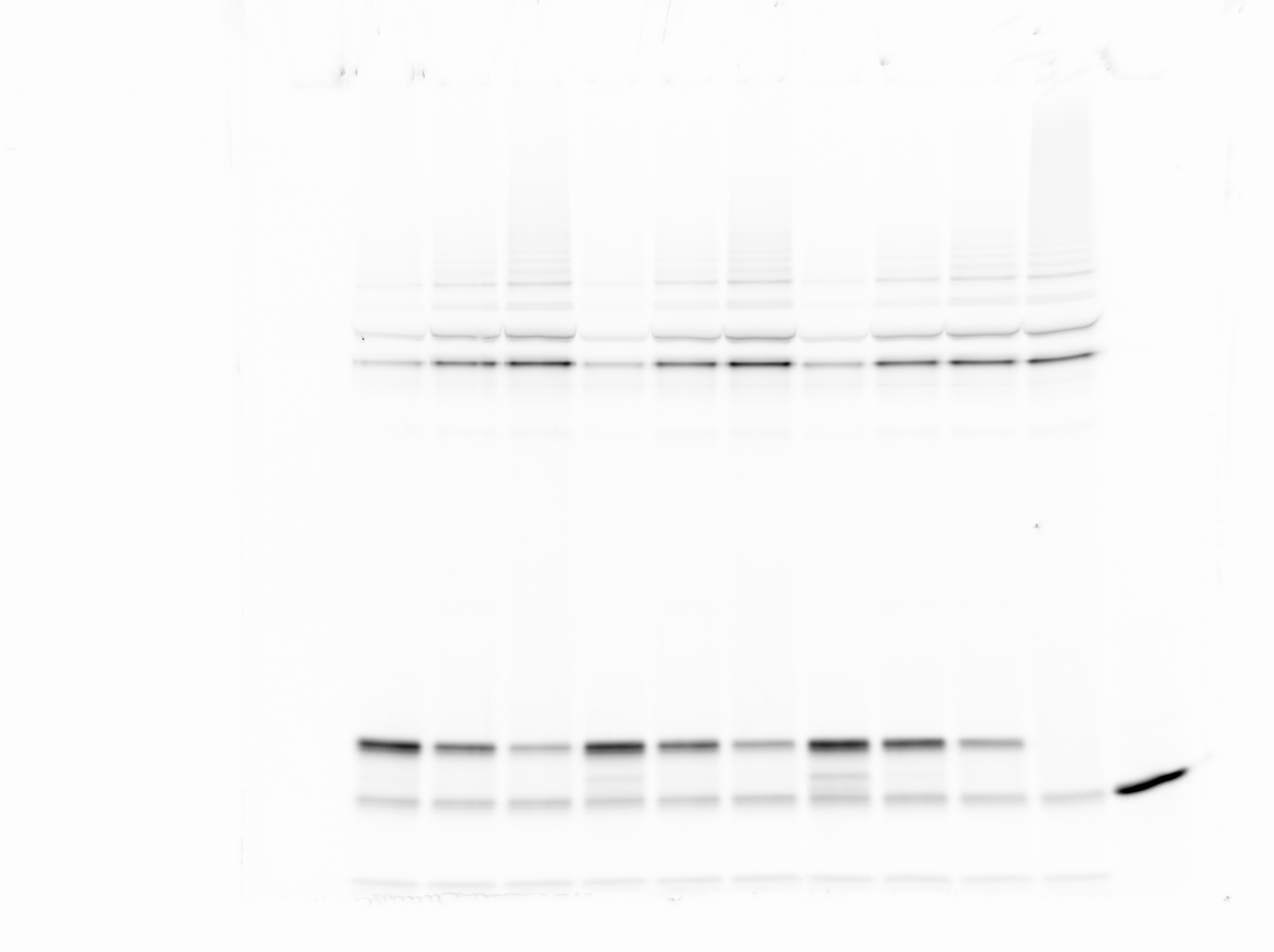

Supplement: Supplementary file 11 — Source Data [file 41467_2025_61224_MOESM11_ESM.zip › Source data/Uncropped scans of all blots and gels/Fig. 5/Fig. 5f/Cy5.tif]

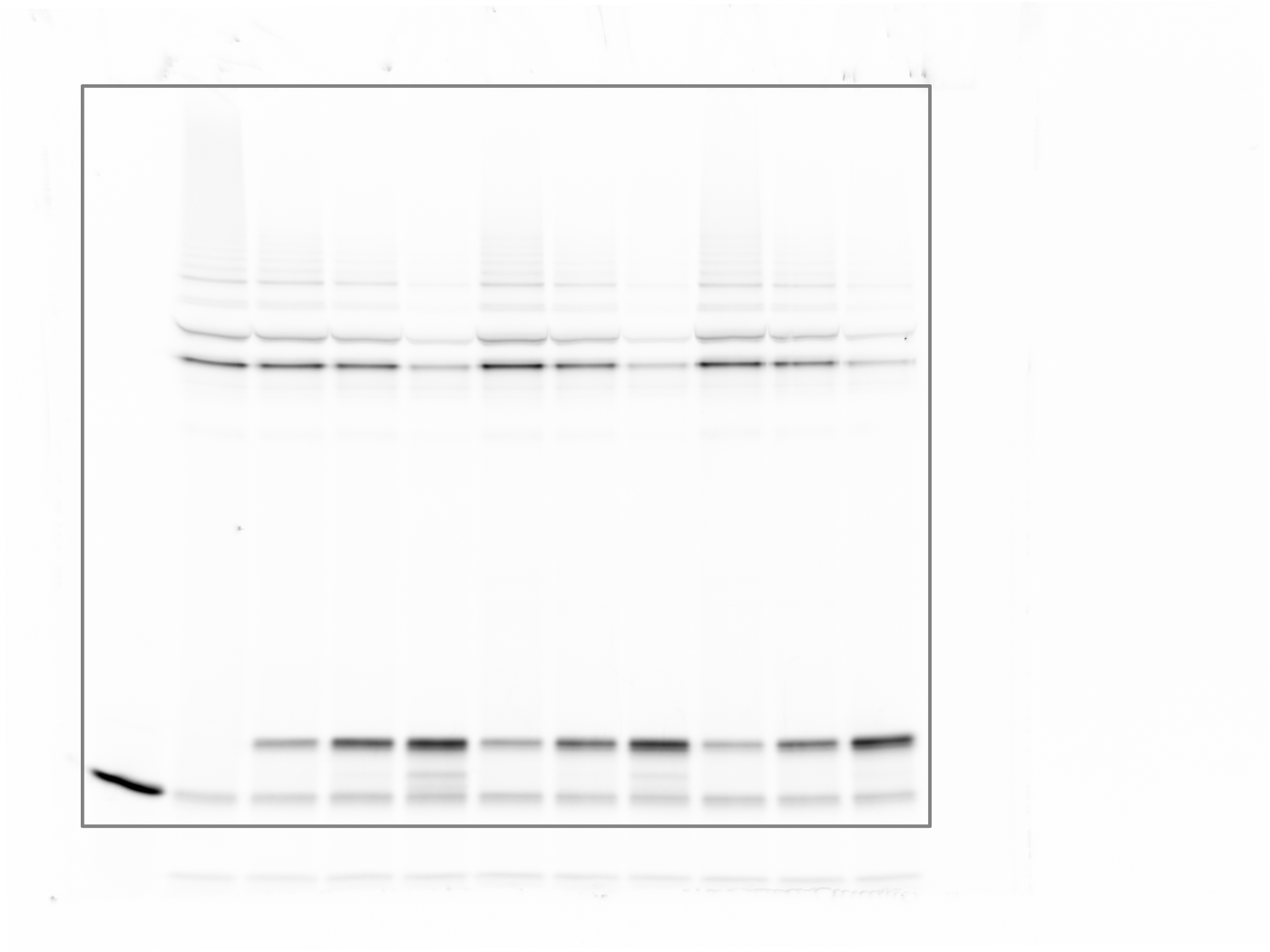

Supplement: Supplementary file 11 — Source Data [file 41467_2025_61224_MOESM11_ESM.zip › Source data/Uncropped scans of all blots and gels/Fig. 5/Fig. 5f/Cy5_label.tiff]

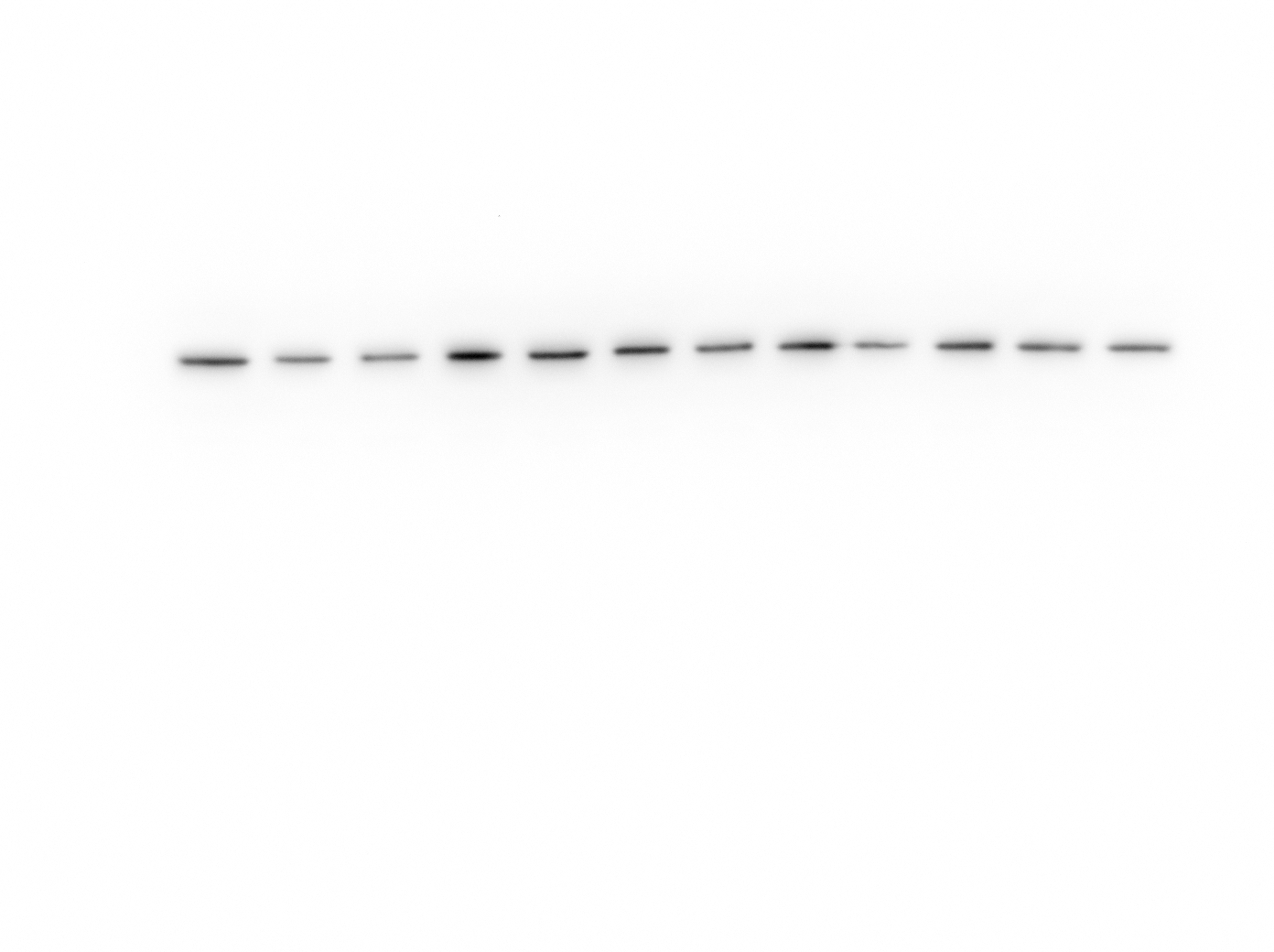

Supplement: Supplementary file 11 — Source Data [file 41467_2025_61224_MOESM11_ESM.zip › Source data/Uncropped scans of all blots and gels/Fig. 5/Fig. 5g/Actin/Actin.tif]

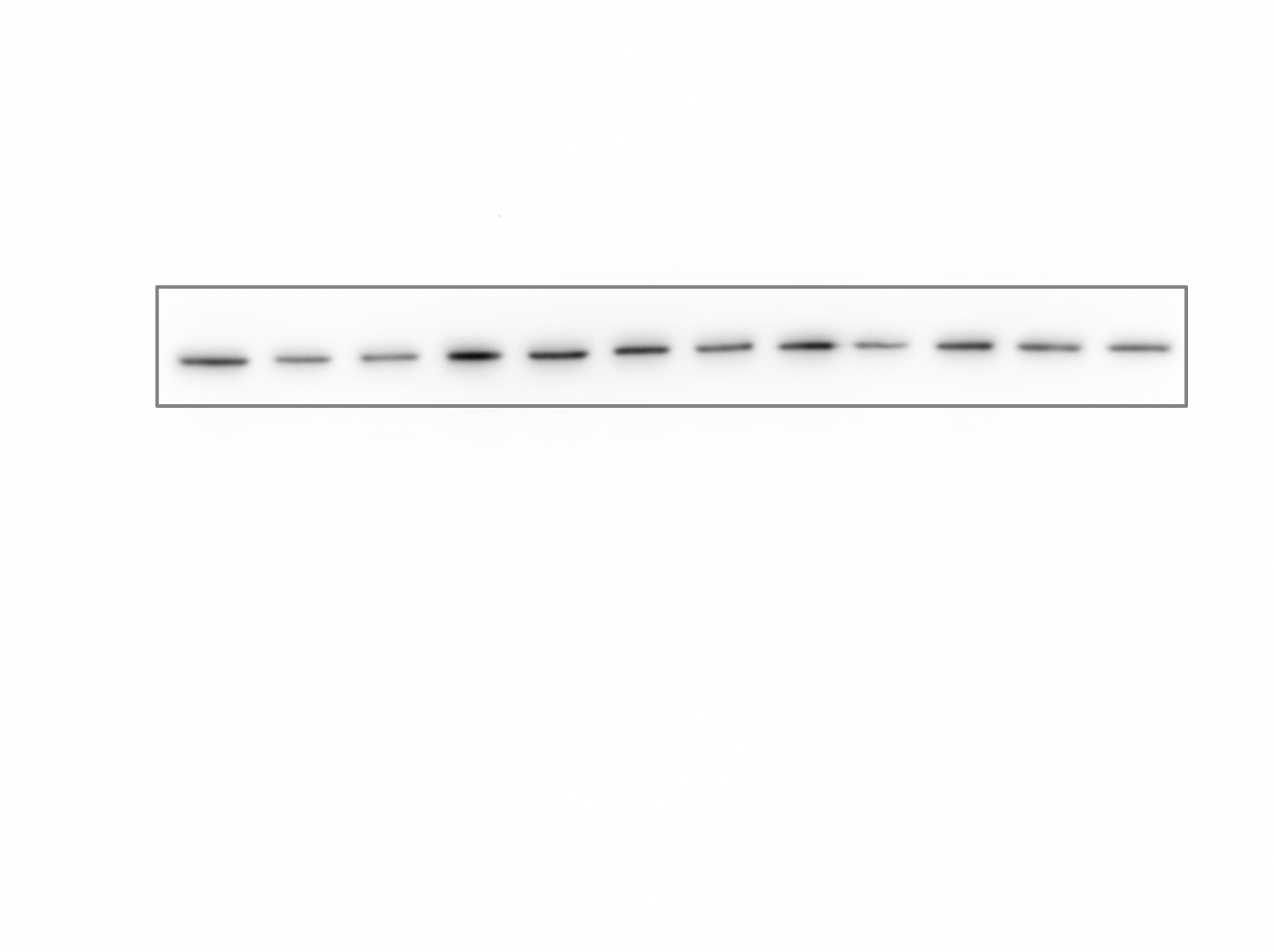

Supplement: Supplementary file 11 — Source Data [file 41467_2025_61224_MOESM11_ESM.zip › Source data/Uncropped scans of all blots and gels/Fig. 5/Fig. 5g/Actin/Actin_label.tiff]

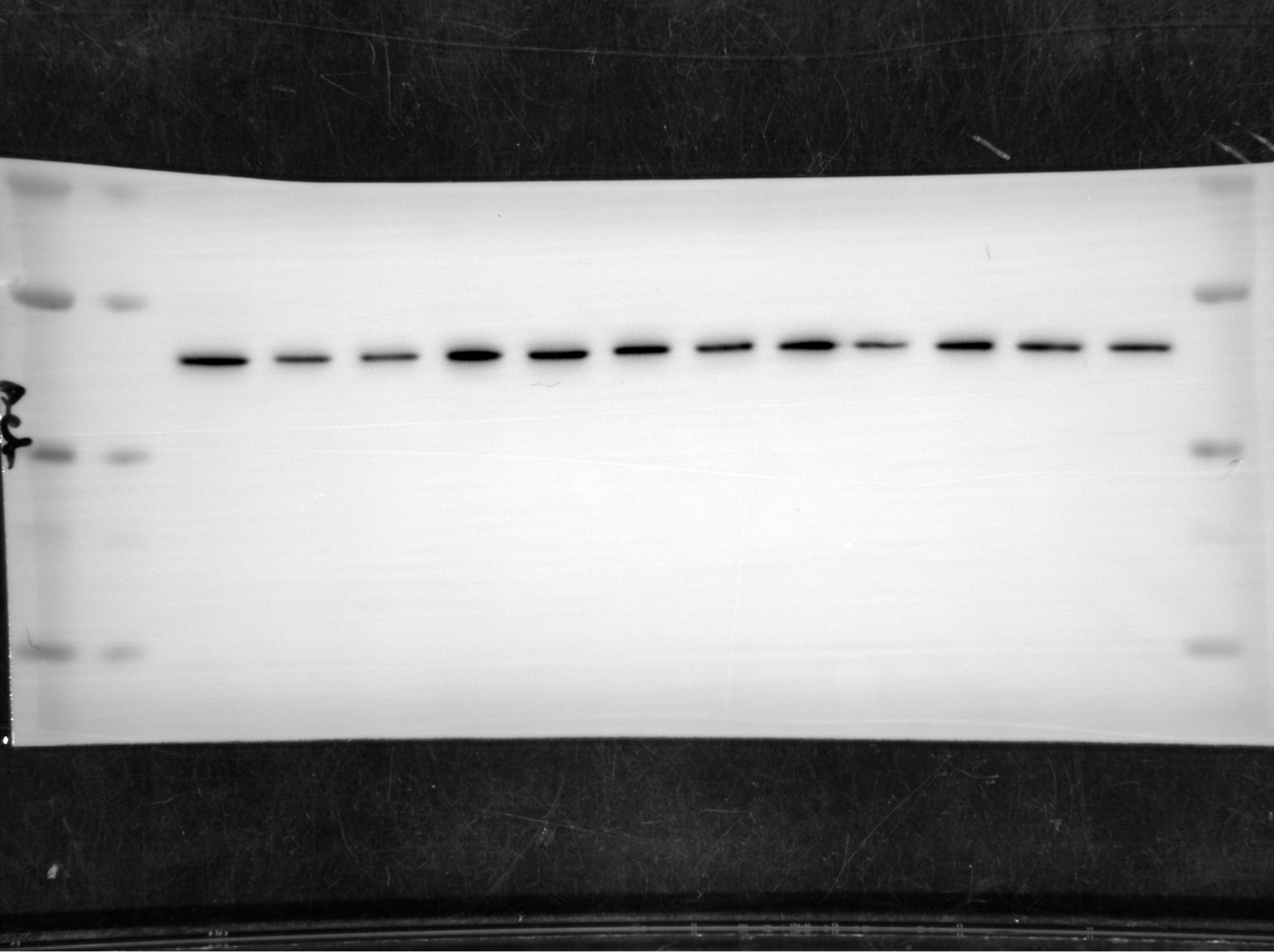

Supplement: Supplementary file 11 — Source Data [file 41467_2025_61224_MOESM11_ESM.zip › Source data/Uncropped scans of all blots and gels/Fig. 5/Fig. 5g/Actin/Actin+M.tif]

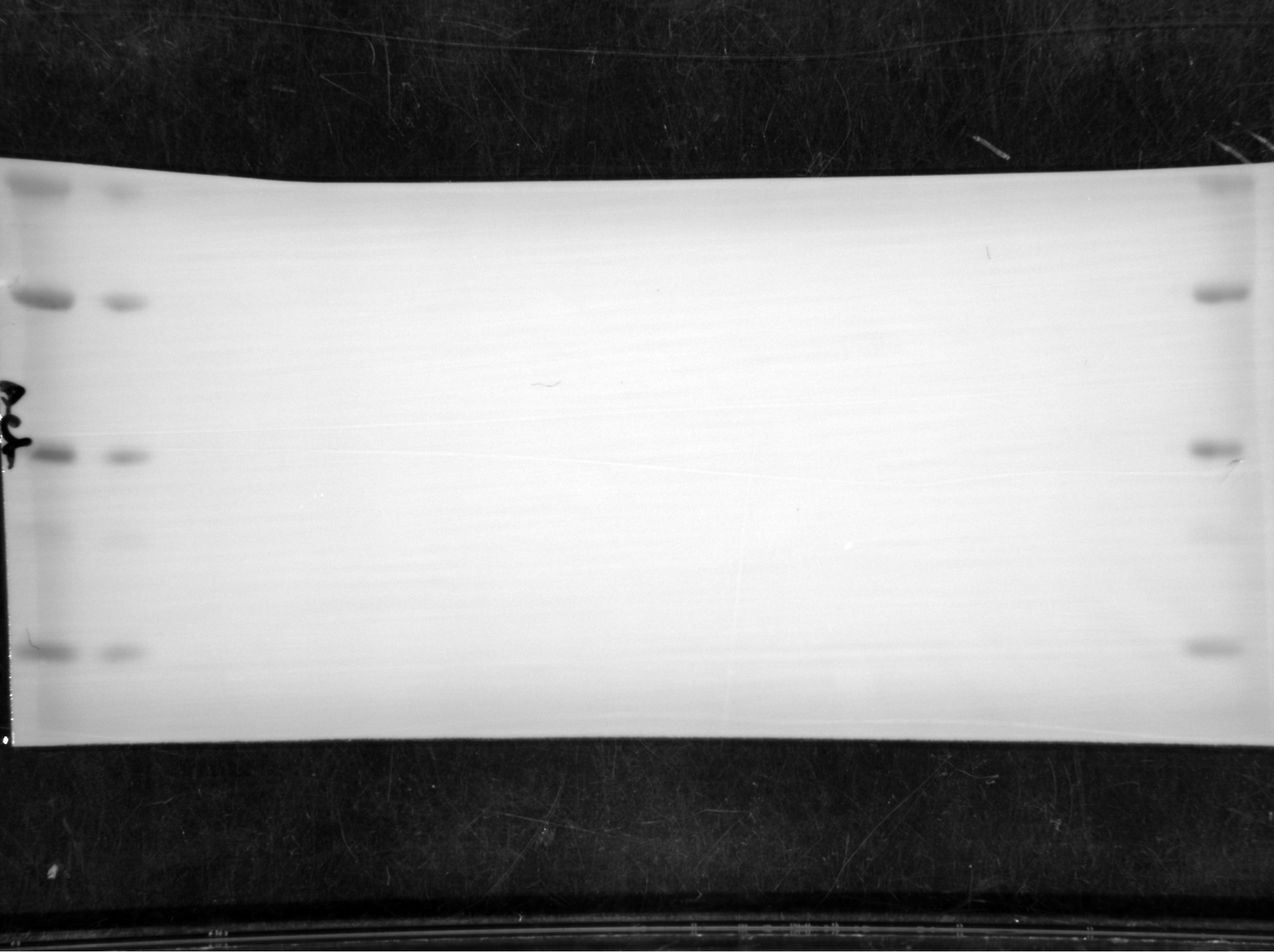

Supplement: Supplementary file 11 — Source Data [file 41467_2025_61224_MOESM11_ESM.zip › Source data/Uncropped scans of all blots and gels/Fig. 5/Fig. 5g/Actin/M.tif]

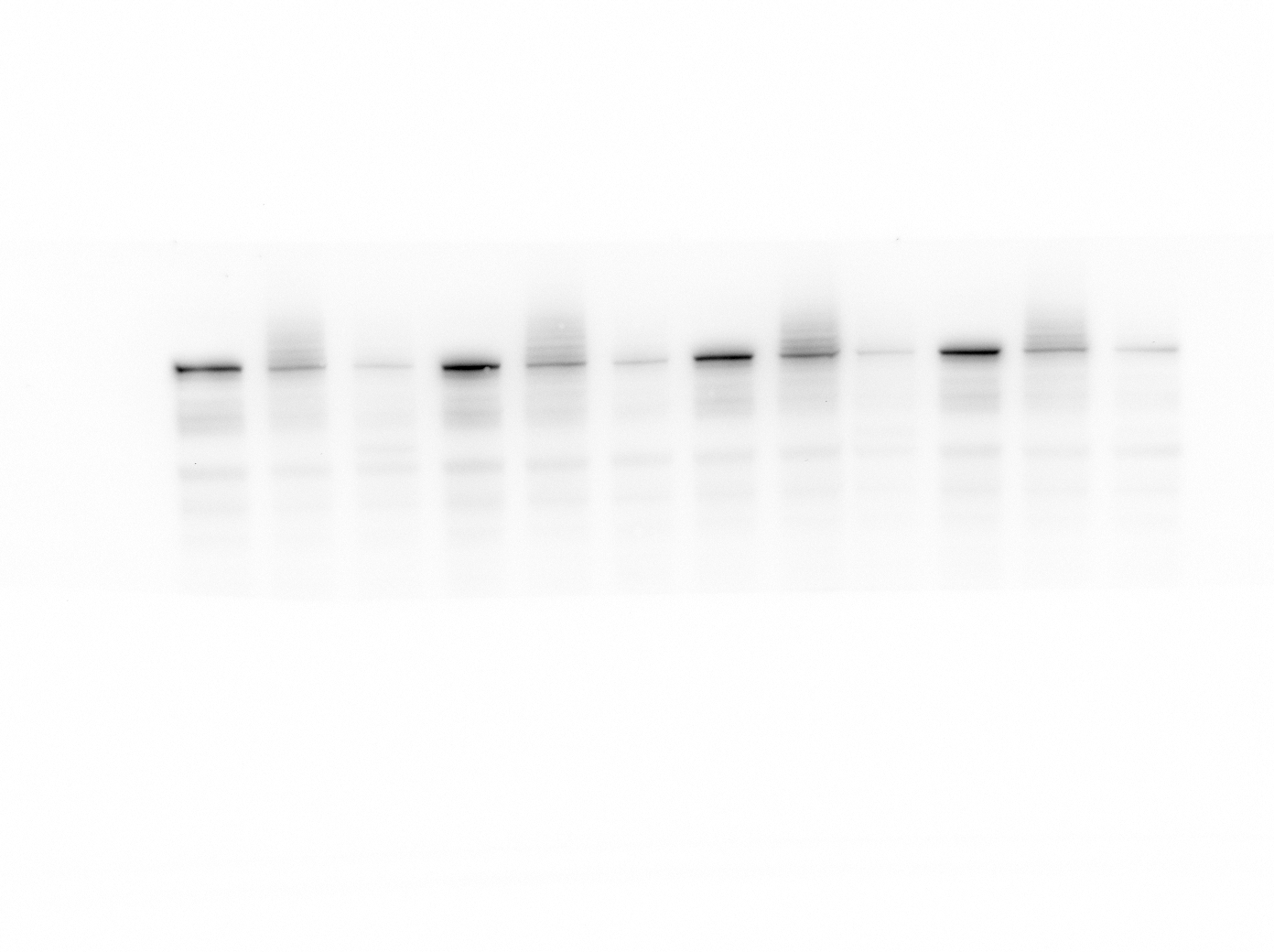

Supplement: Supplementary file 11 — Source Data [file 41467_2025_61224_MOESM11_ESM.zip › Source data/Uncropped scans of all blots and gels/Fig. 5/Fig. 5g/Input_DNMT1/DNMT1.tif]

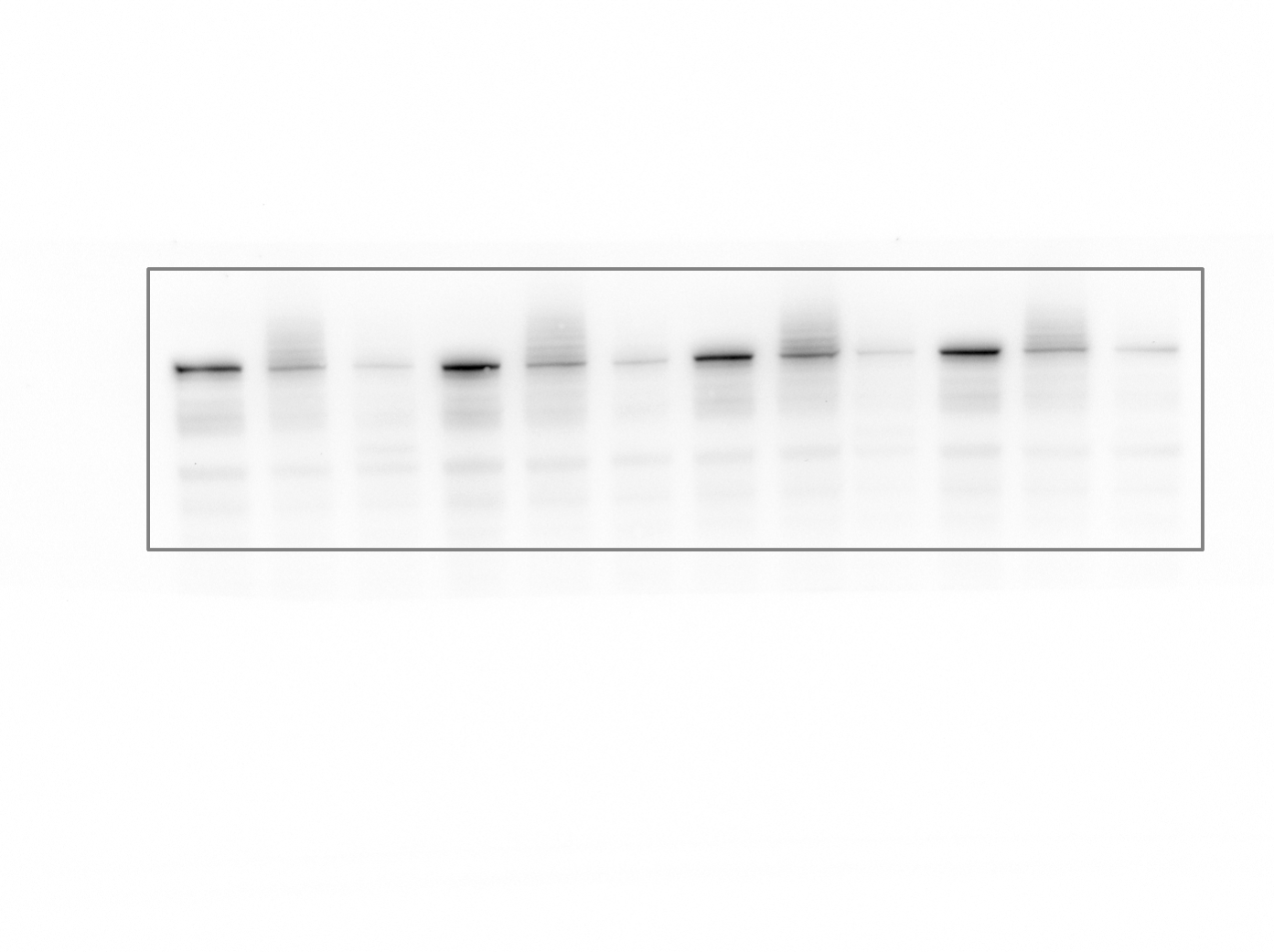

Supplement: Supplementary file 11 — Source Data [file 41467_2025_61224_MOESM11_ESM.zip › Source data/Uncropped scans of all blots and gels/Fig. 5/Fig. 5g/Input_DNMT1/DNMT1_label.tiff]

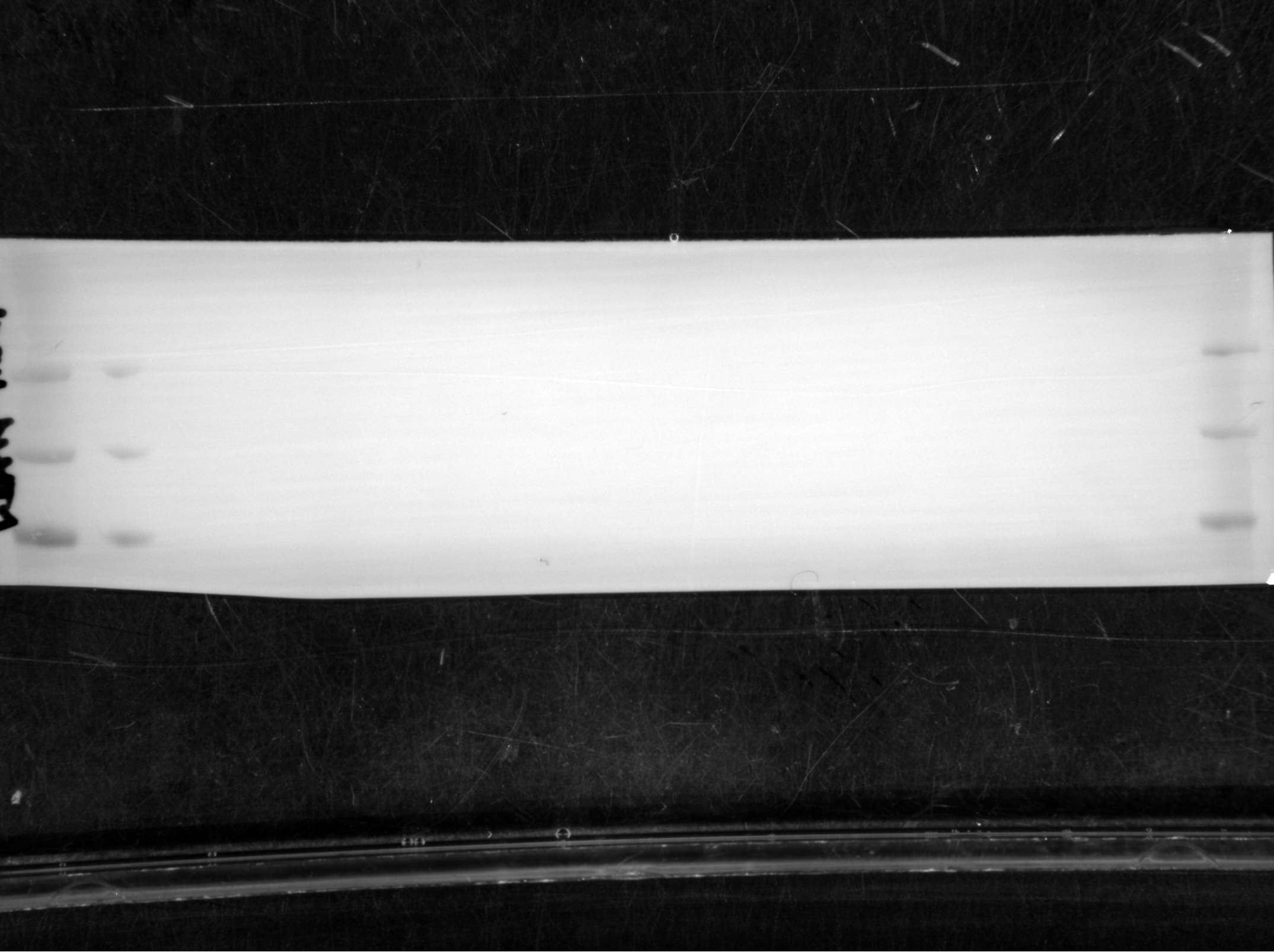

Supplement: Supplementary file 11 — Source Data [file 41467_2025_61224_MOESM11_ESM.zip › Source data/Uncropped scans of all blots and gels/Fig. 5/Fig. 5g/Input_DNMT1/M.tif]

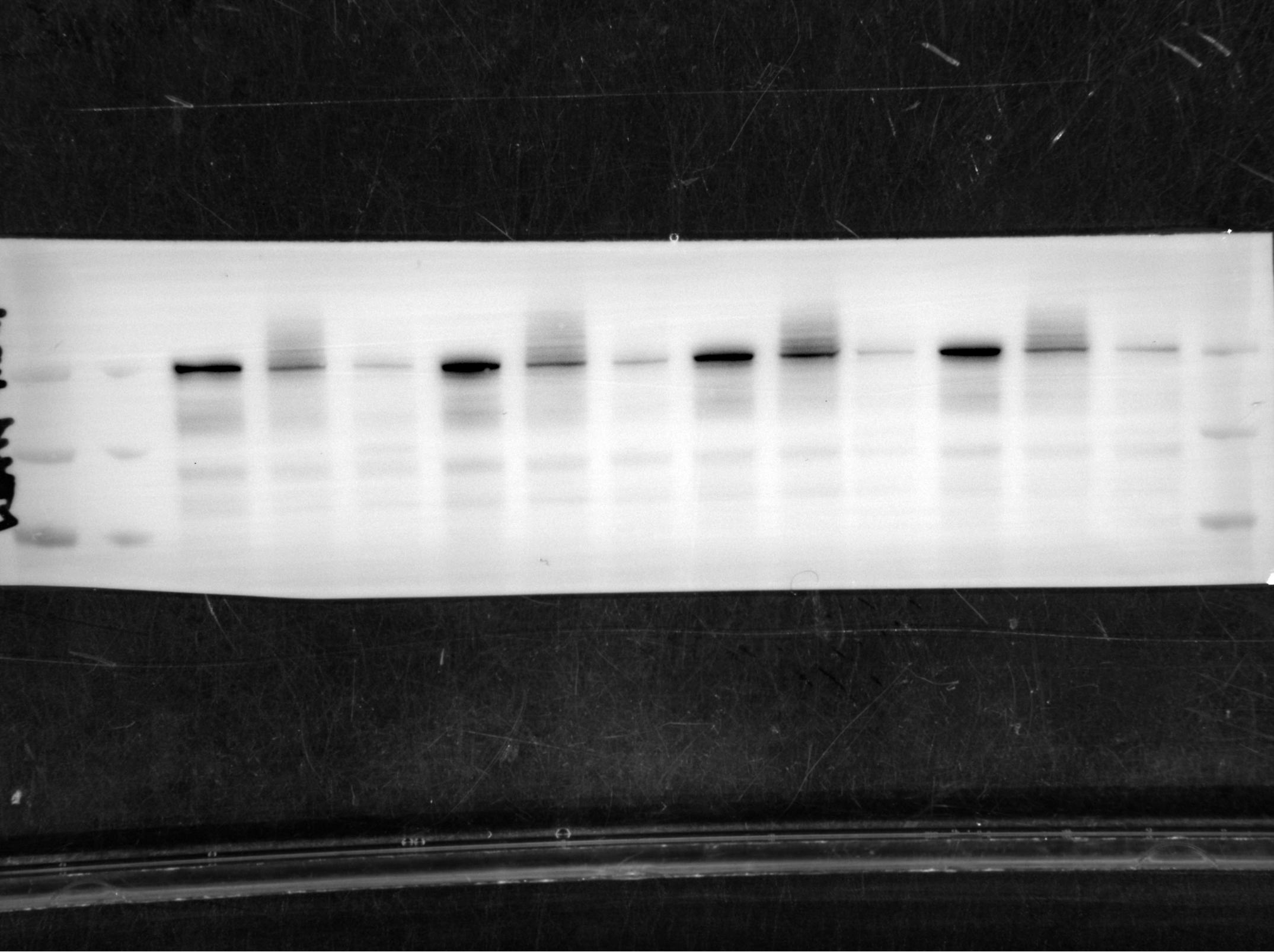

Supplement: Supplementary file 11 — Source Data [file 41467_2025_61224_MOESM11_ESM.zip › Source data/Uncropped scans of all blots and gels/Fig. 5/Fig. 5g/Input_DNMT1/M+DNMT1.tif]

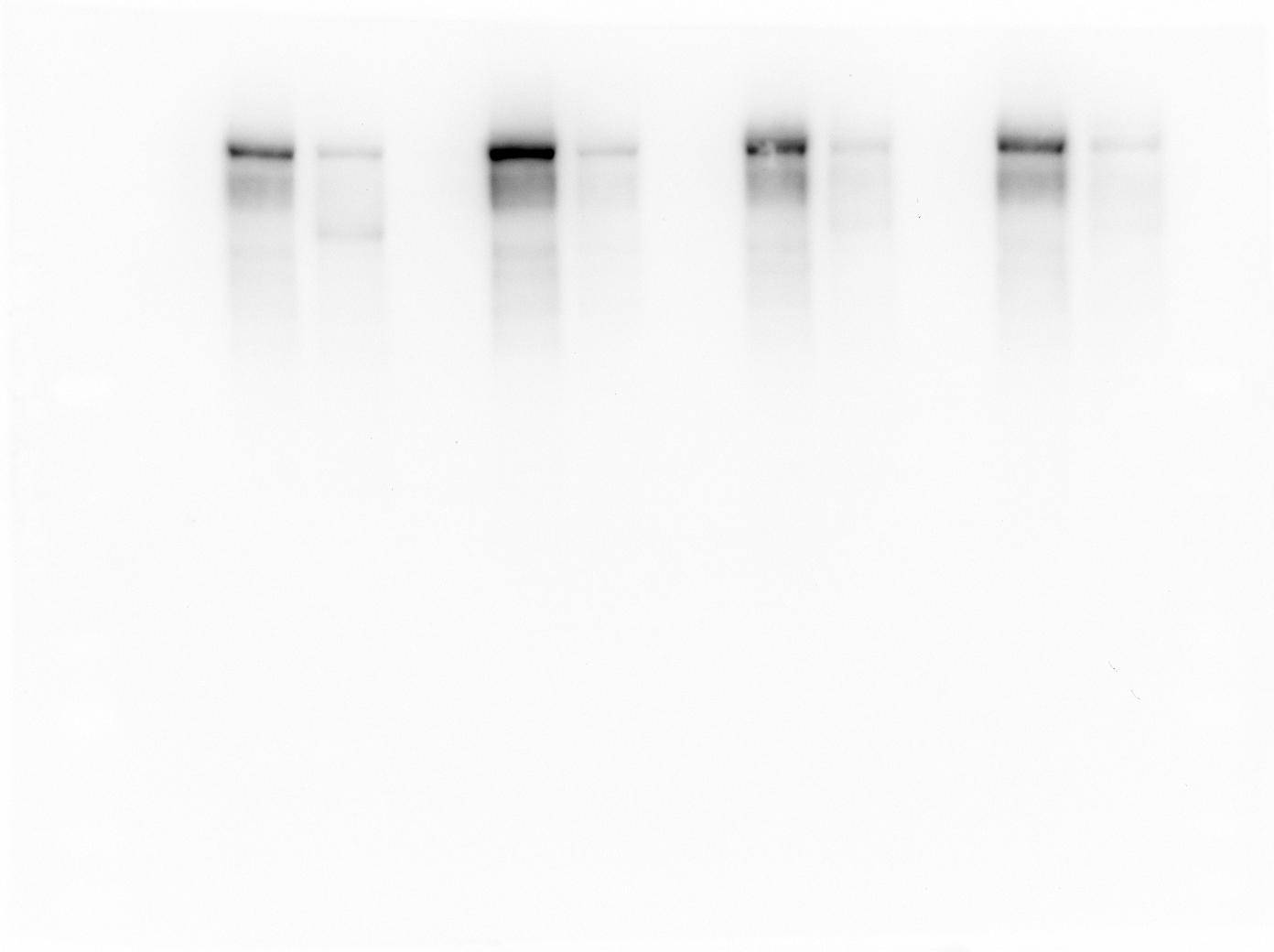

Supplement: Supplementary file 11 — Source Data [file 41467_2025_61224_MOESM11_ESM.zip › Source data/Uncropped scans of all blots and gels/Fig. 5/Fig. 5g/PxP_DNMT1/DNMT1.tif]

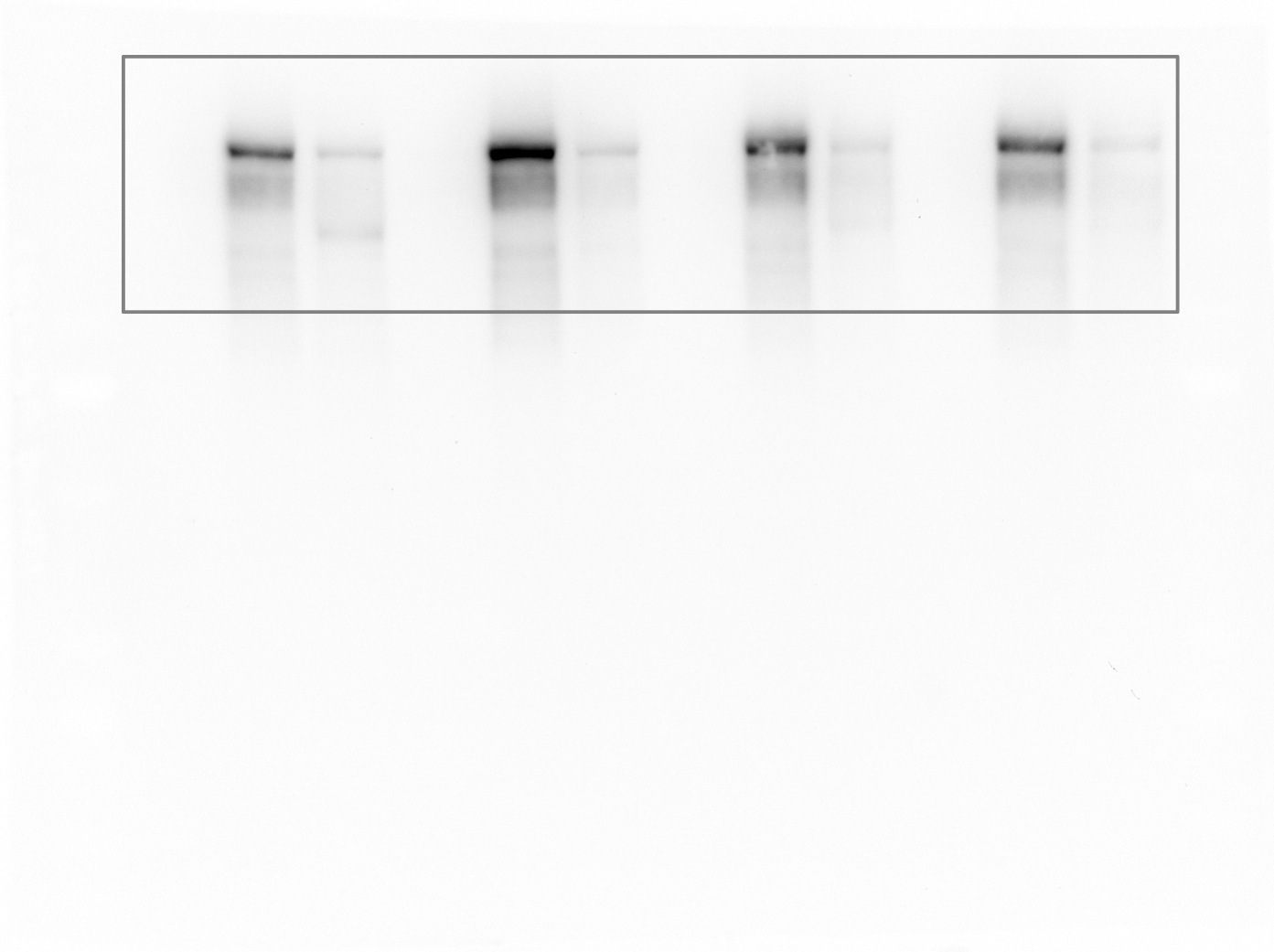

Supplement: Supplementary file 11 — Source Data [file 41467_2025_61224_MOESM11_ESM.zip › Source data/Uncropped scans of all blots and gels/Fig. 5/Fig. 5g/PxP_DNMT1/DNMT1_label.tiff]

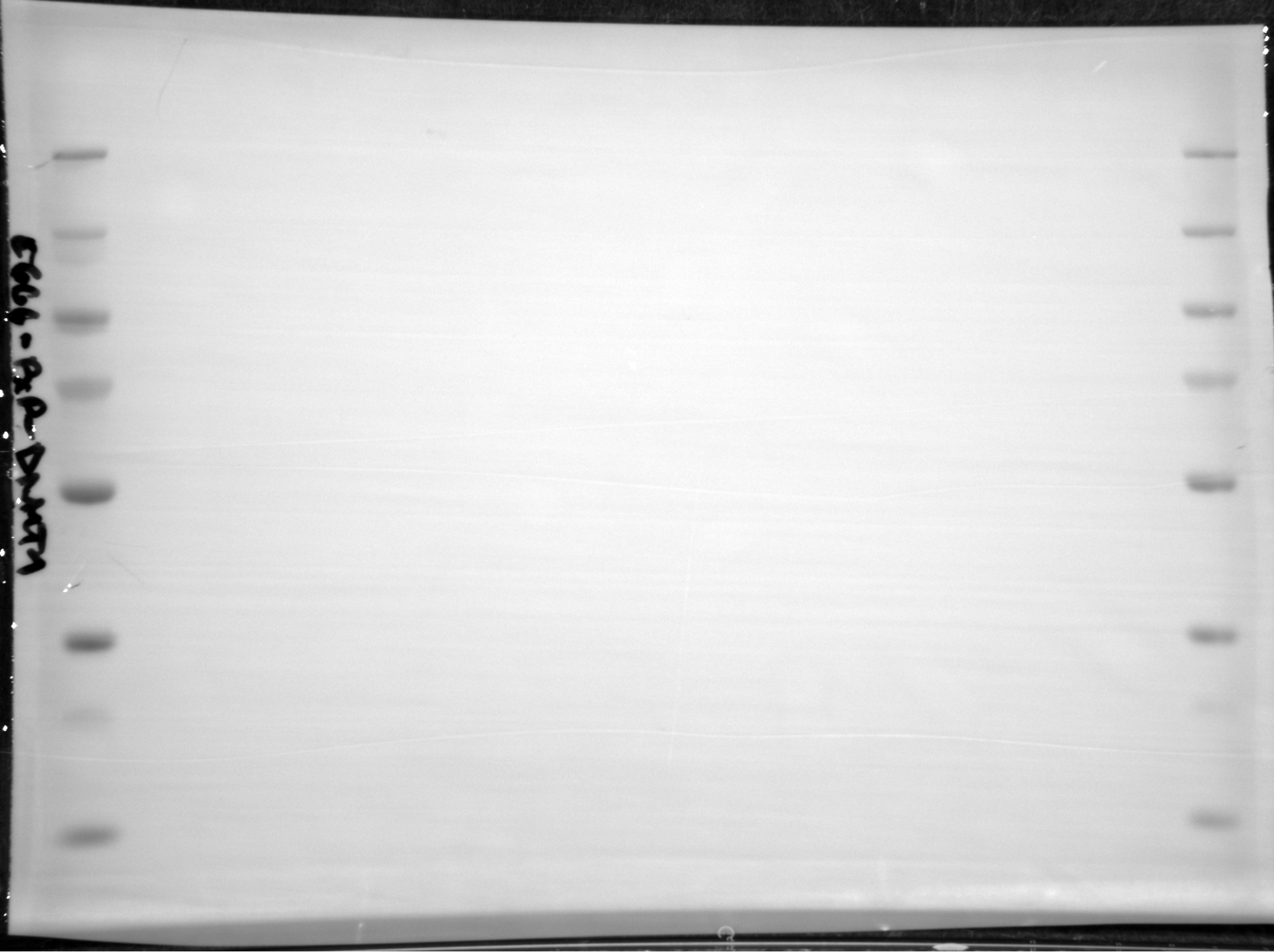

Supplement: Supplementary file 11 — Source Data [file 41467_2025_61224_MOESM11_ESM.zip › Source data/Uncropped scans of all blots and gels/Fig. 5/Fig. 5g/PxP_DNMT1/M.tif]

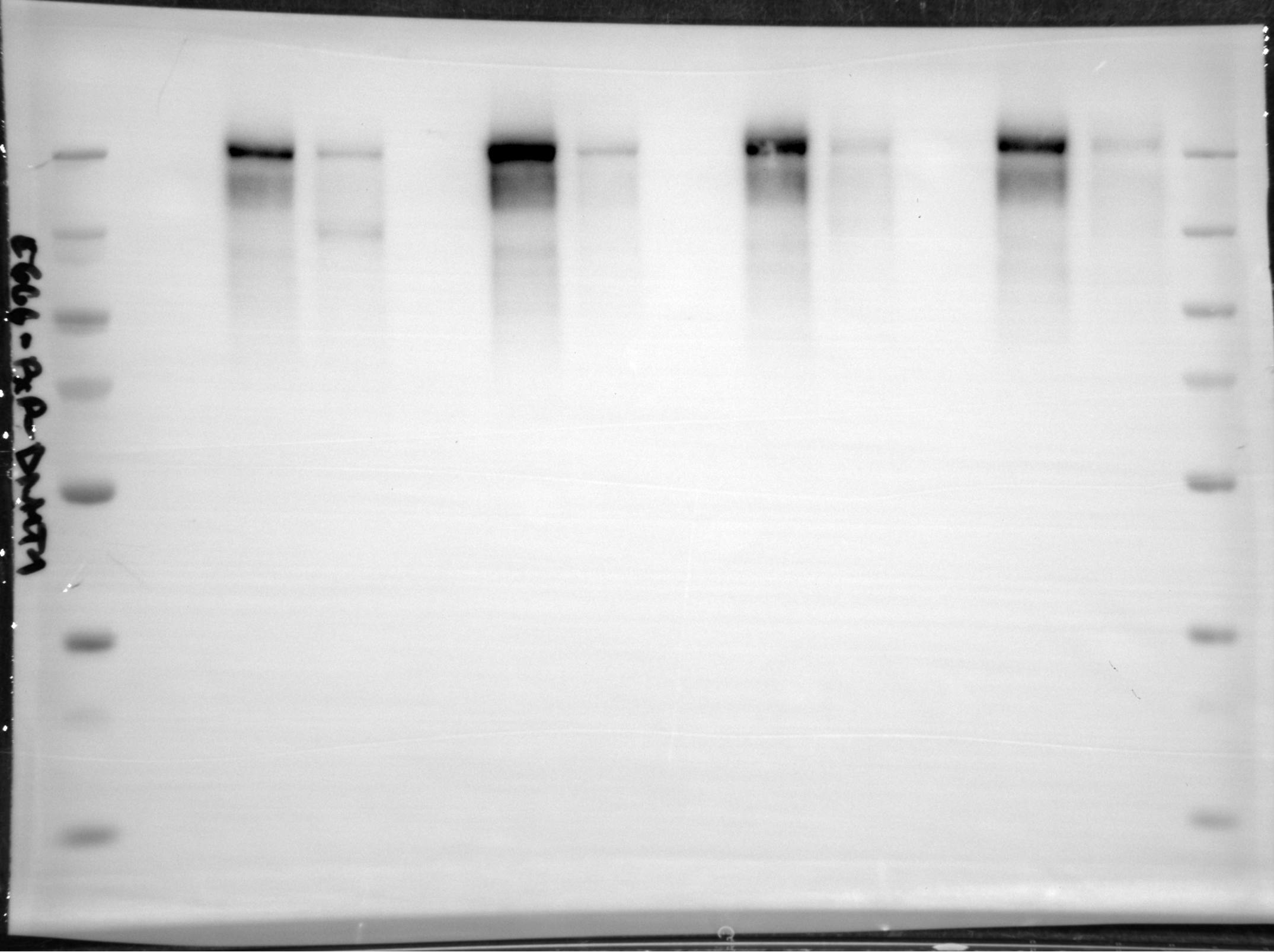

Supplement: Supplementary file 11 — Source Data [file 41467_2025_61224_MOESM11_ESM.zip › Source data/Uncropped scans of all blots and gels/Fig. 5/Fig. 5g/PxP_DNMT1/M+DNMT1.tif]

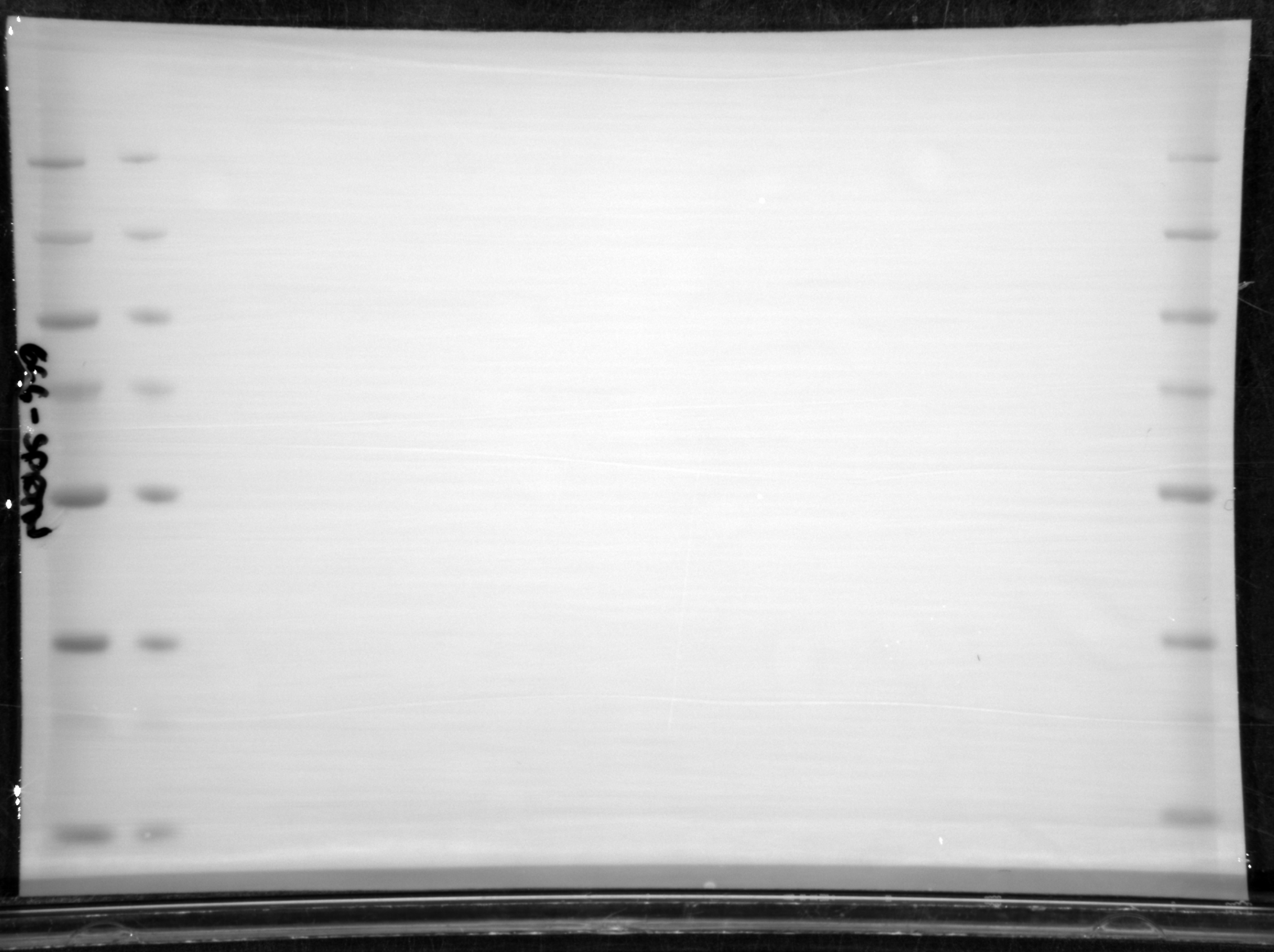

Supplement: Supplementary file 11 — Source Data [file 41467_2025_61224_MOESM11_ESM.zip › Source data/Uncropped scans of all blots and gels/Fig. 5/Fig. 5g/SPRTN/M.tif]

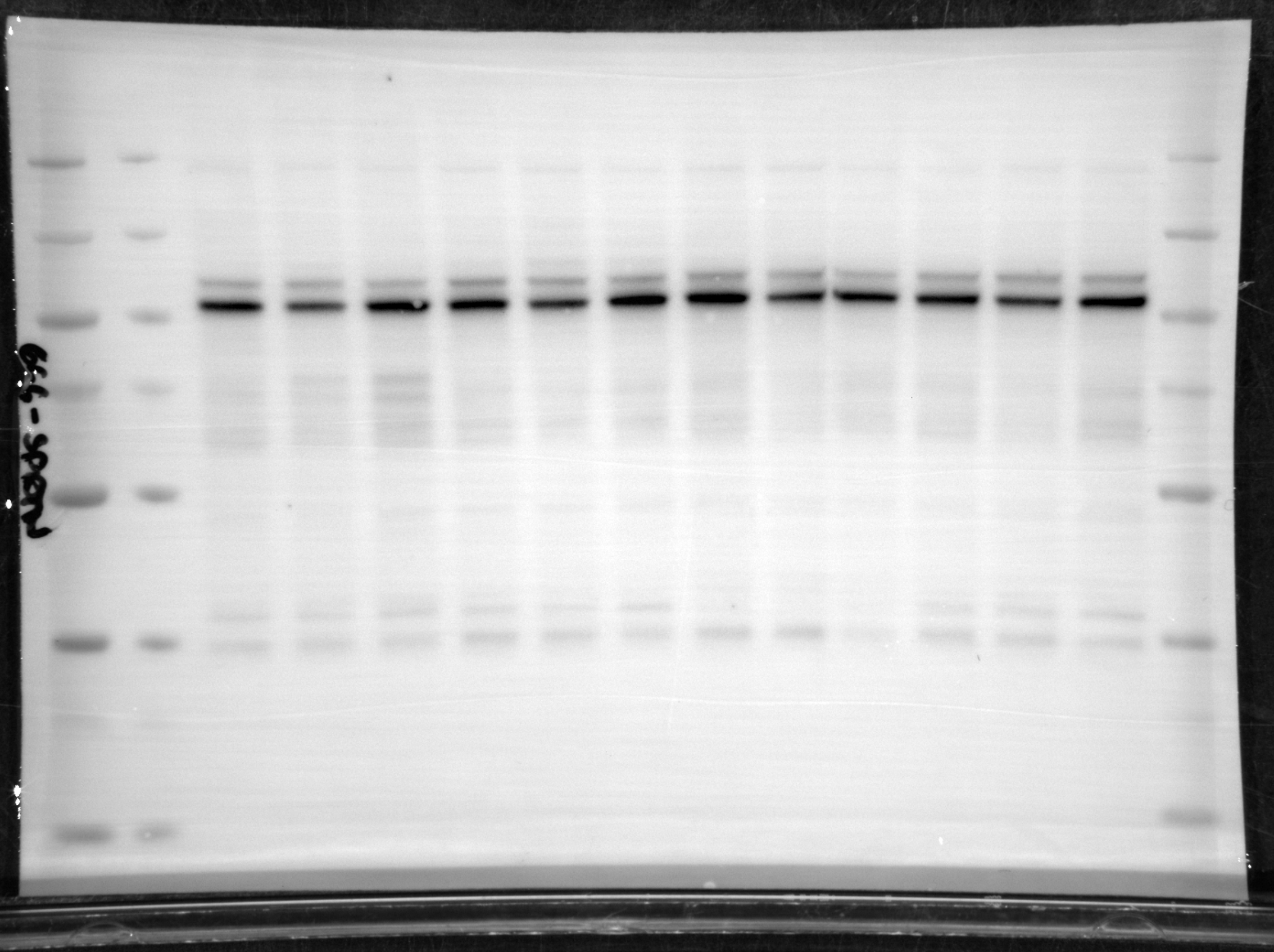

Supplement: Supplementary file 11 — Source Data [file 41467_2025_61224_MOESM11_ESM.zip › Source data/Uncropped scans of all blots and gels/Fig. 5/Fig. 5g/SPRTN/M+SPRTN.tif]

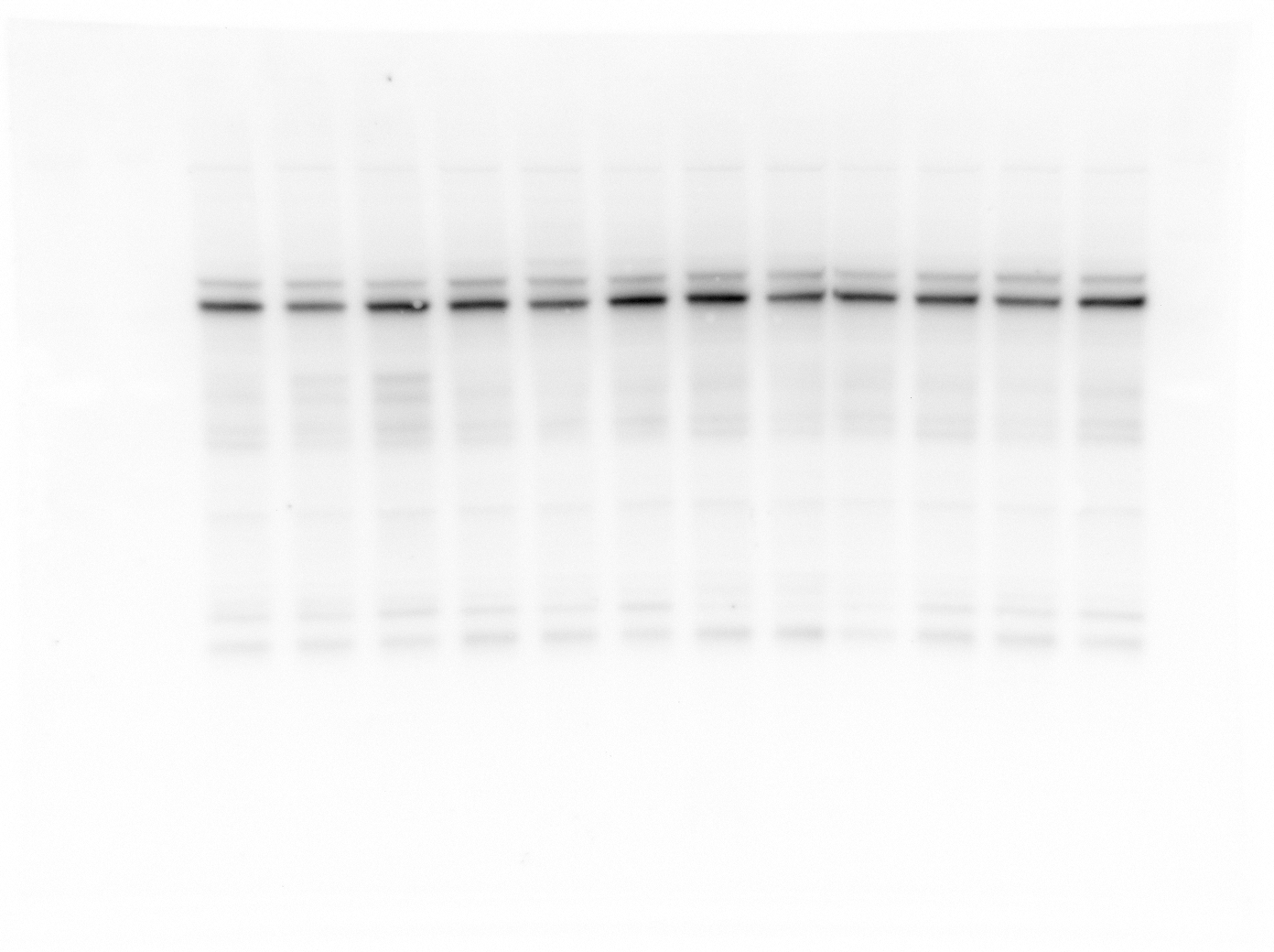

Supplement: Supplementary file 11 — Source Data [file 41467_2025_61224_MOESM11_ESM.zip › Source data/Uncropped scans of all blots and gels/Fig. 5/Fig. 5g/SPRTN/SPRTN.tif]

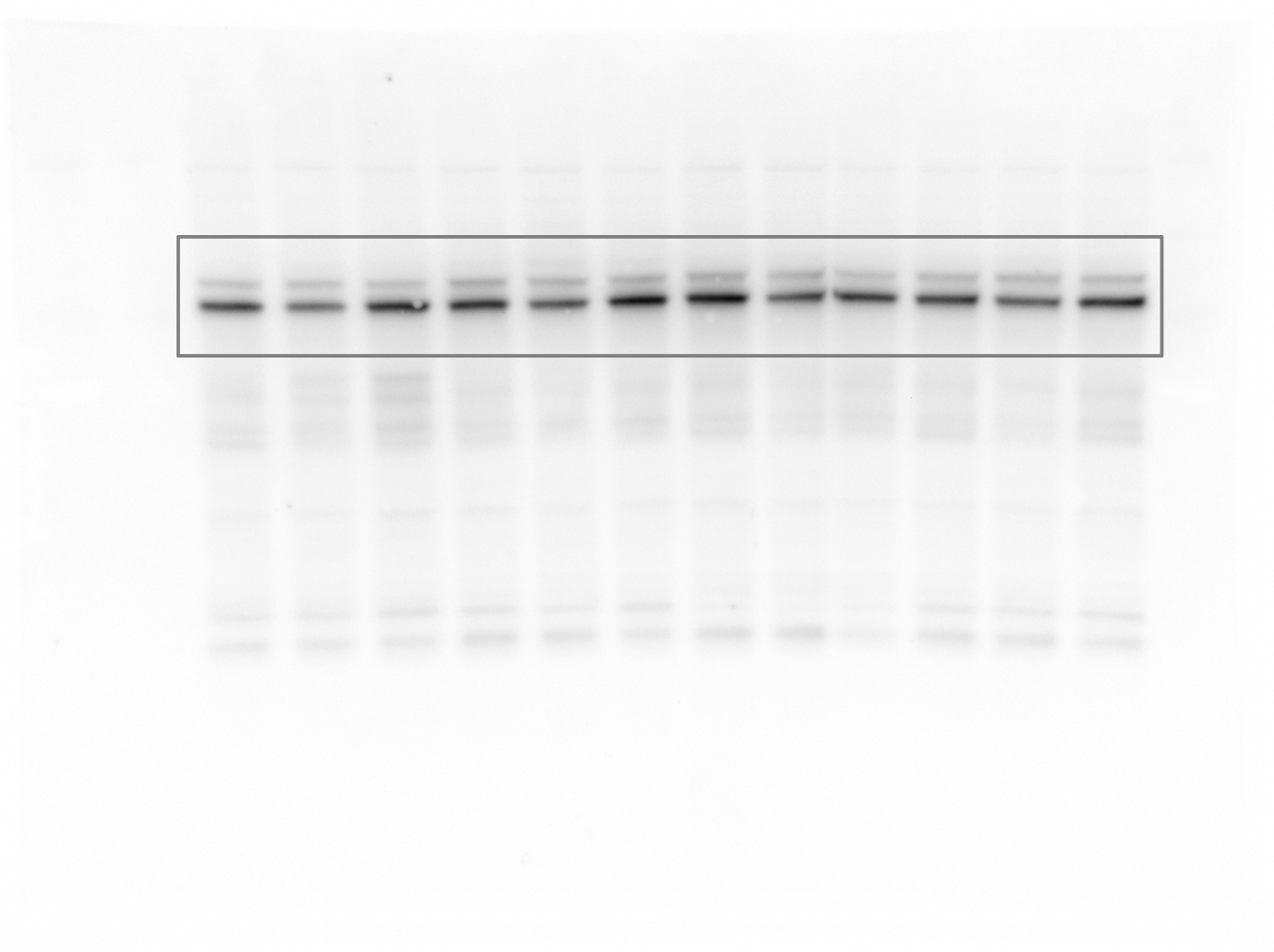

Supplement: Supplementary file 11 — Source Data [file 41467_2025_61224_MOESM11_ESM.zip › Source data/Uncropped scans of all blots and gels/Fig. 5/Fig. 5g/SPRTN/SPRTN_label.tiff]

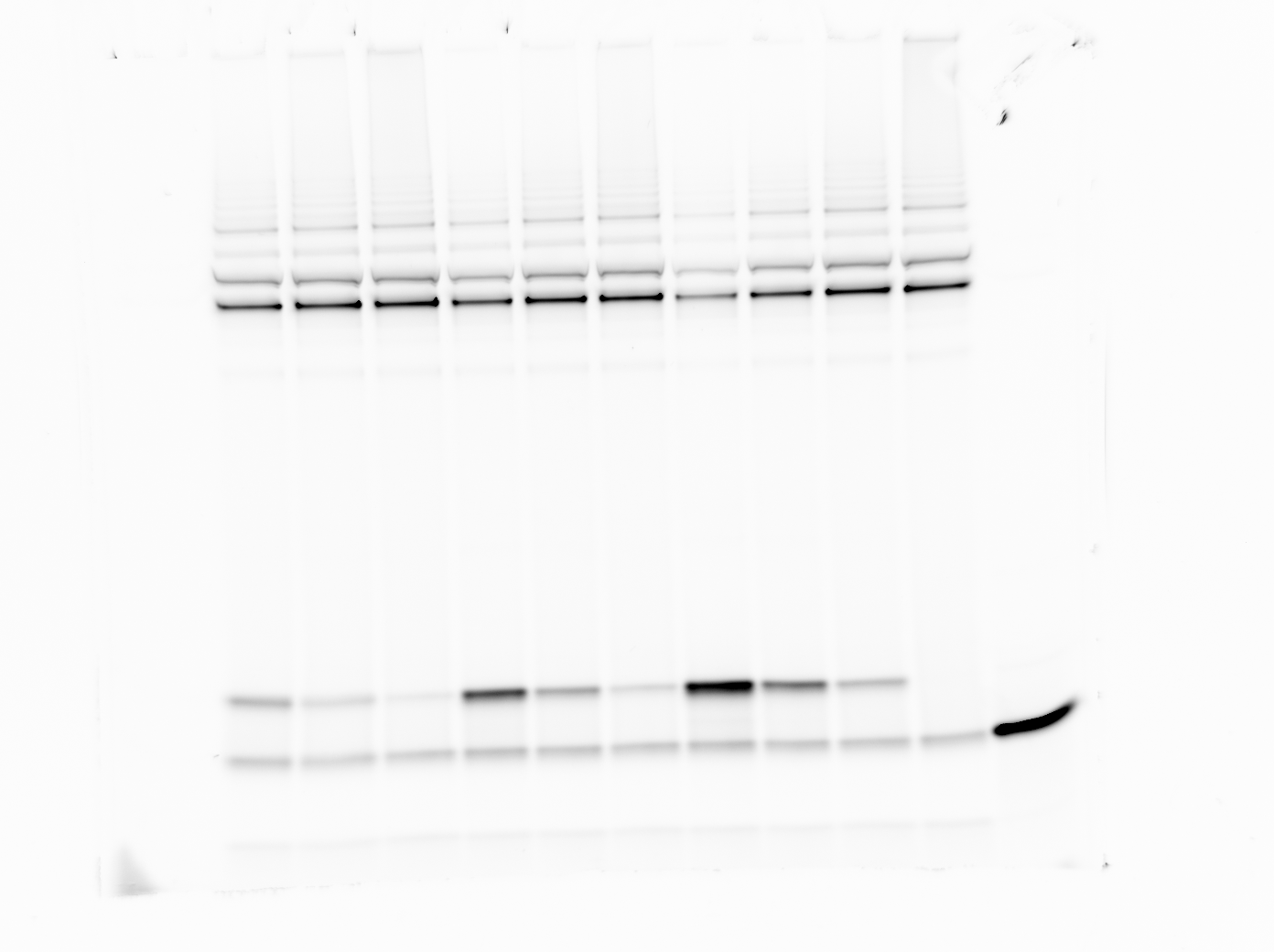

Supplement: Supplementary file 11 — Source Data [file 41467_2025_61224_MOESM11_ESM.zip › Source data/Uncropped scans of all blots and gels/Fig. 5/Fig. 5h/L99S_Cy5.tif]

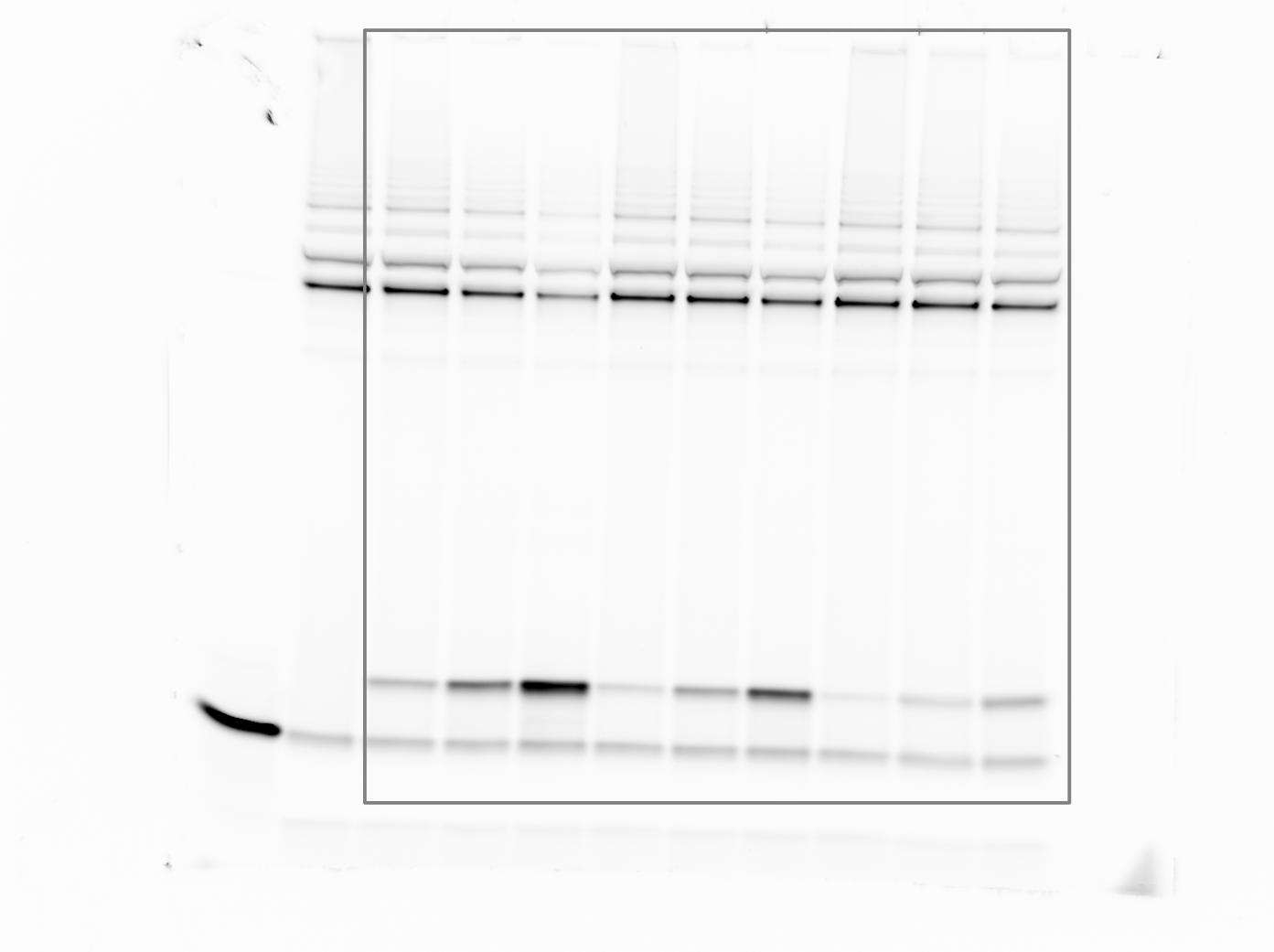

Supplement: Supplementary file 11 — Source Data [file 41467_2025_61224_MOESM11_ESM.zip › Source data/Uncropped scans of all blots and gels/Fig. 5/Fig. 5h/L99S_Cy5_label.tiff]

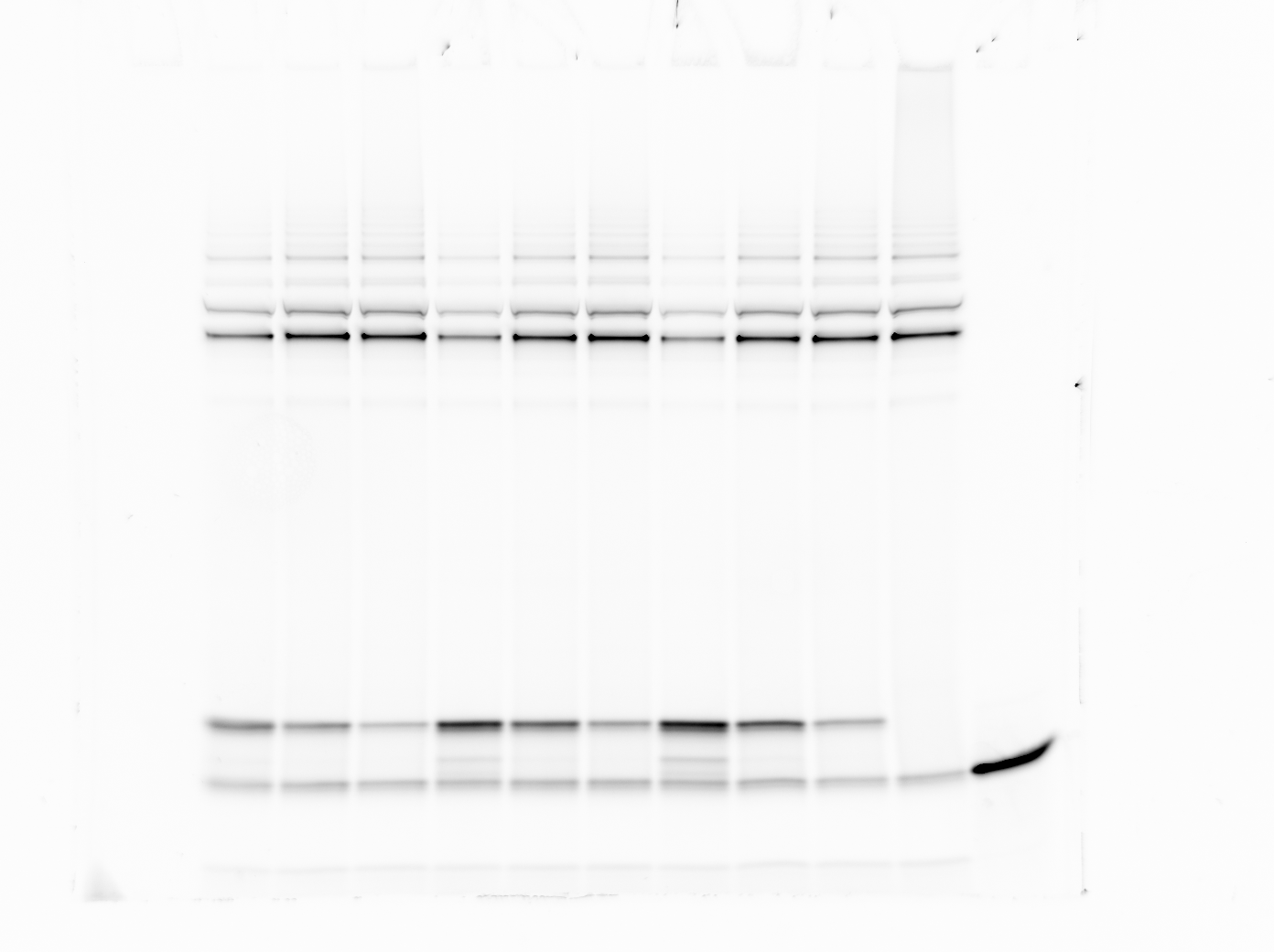

Supplement: Supplementary file 11 — Source Data [file 41467_2025_61224_MOESM11_ESM.zip › Source data/Uncropped scans of all blots and gels/Fig. 5/Fig. 5h/WT_Cy5.tif]

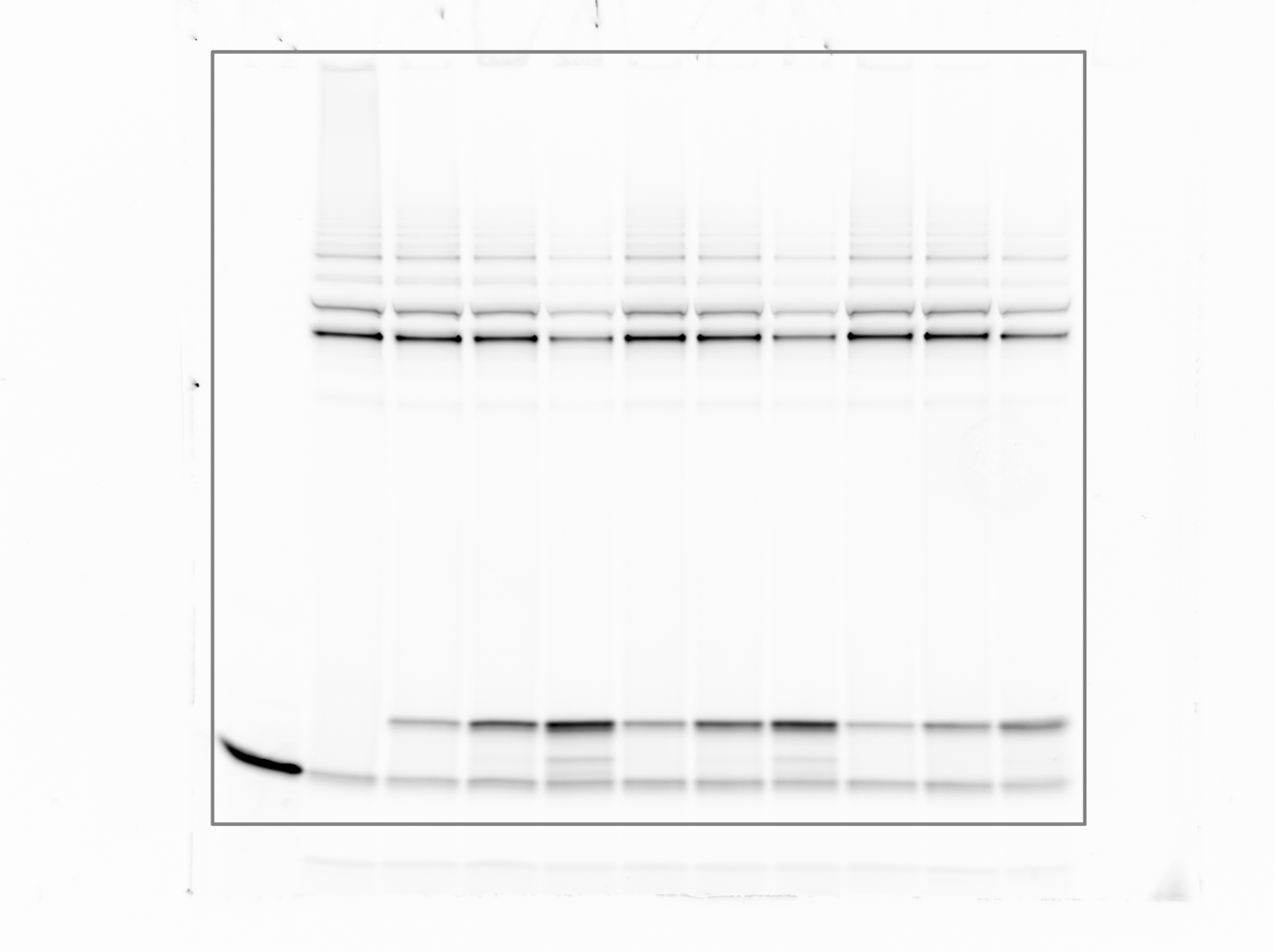

Supplement: Supplementary file 11 — Source Data [file 41467_2025_61224_MOESM11_ESM.zip › Source data/Uncropped scans of all blots and gels/Fig. 5/Fig. 5h/WT_Cy5_label.tiff]

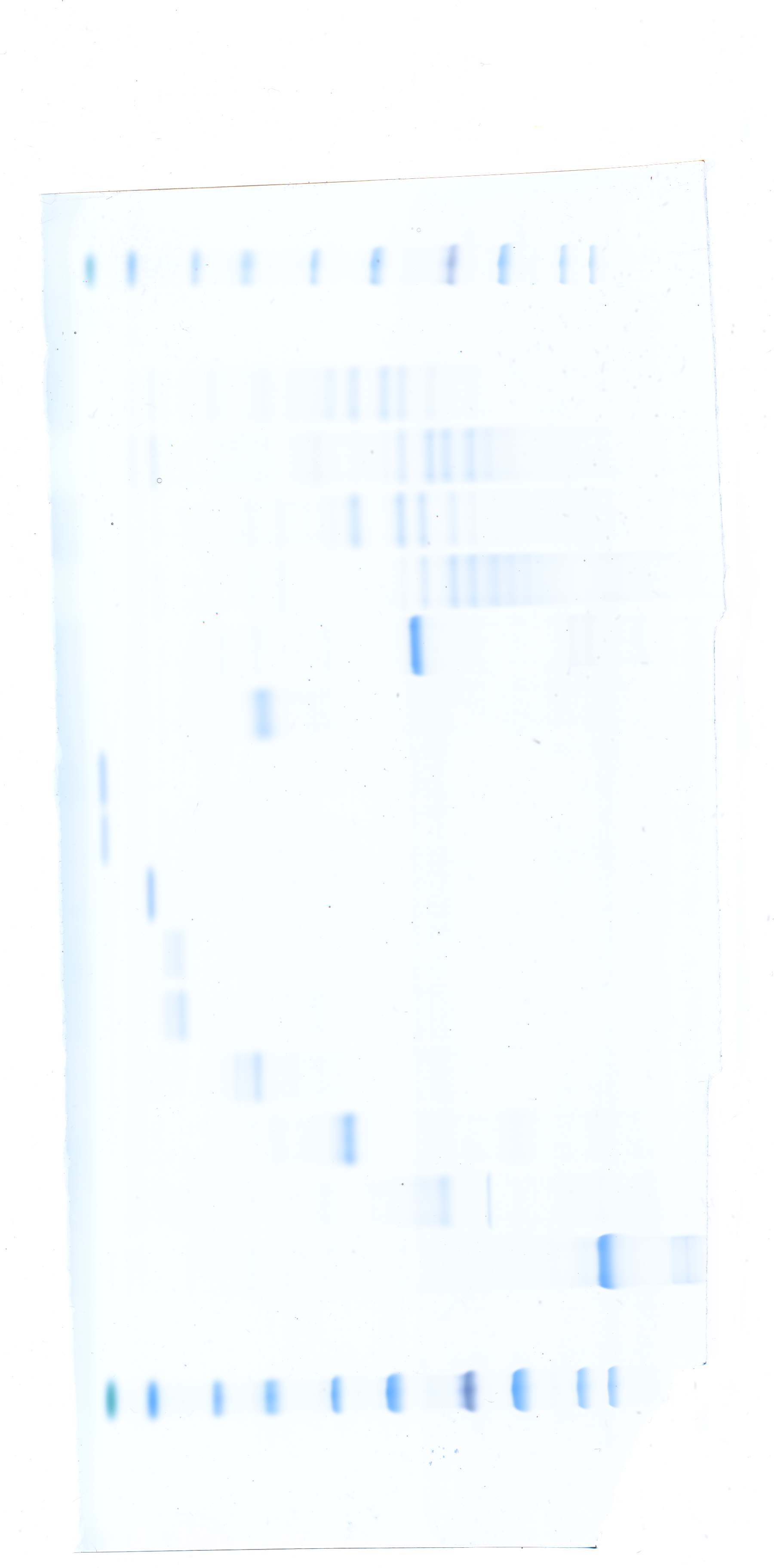

Supplement: Supplementary file 11 — Source Data [file 41467_2025_61224_MOESM11_ESM.zip › Source data/Uncropped scans of all blots and gels/Supplementary Fig. 1/Supplementary Fig. 1a/Left_Coomassie.png]

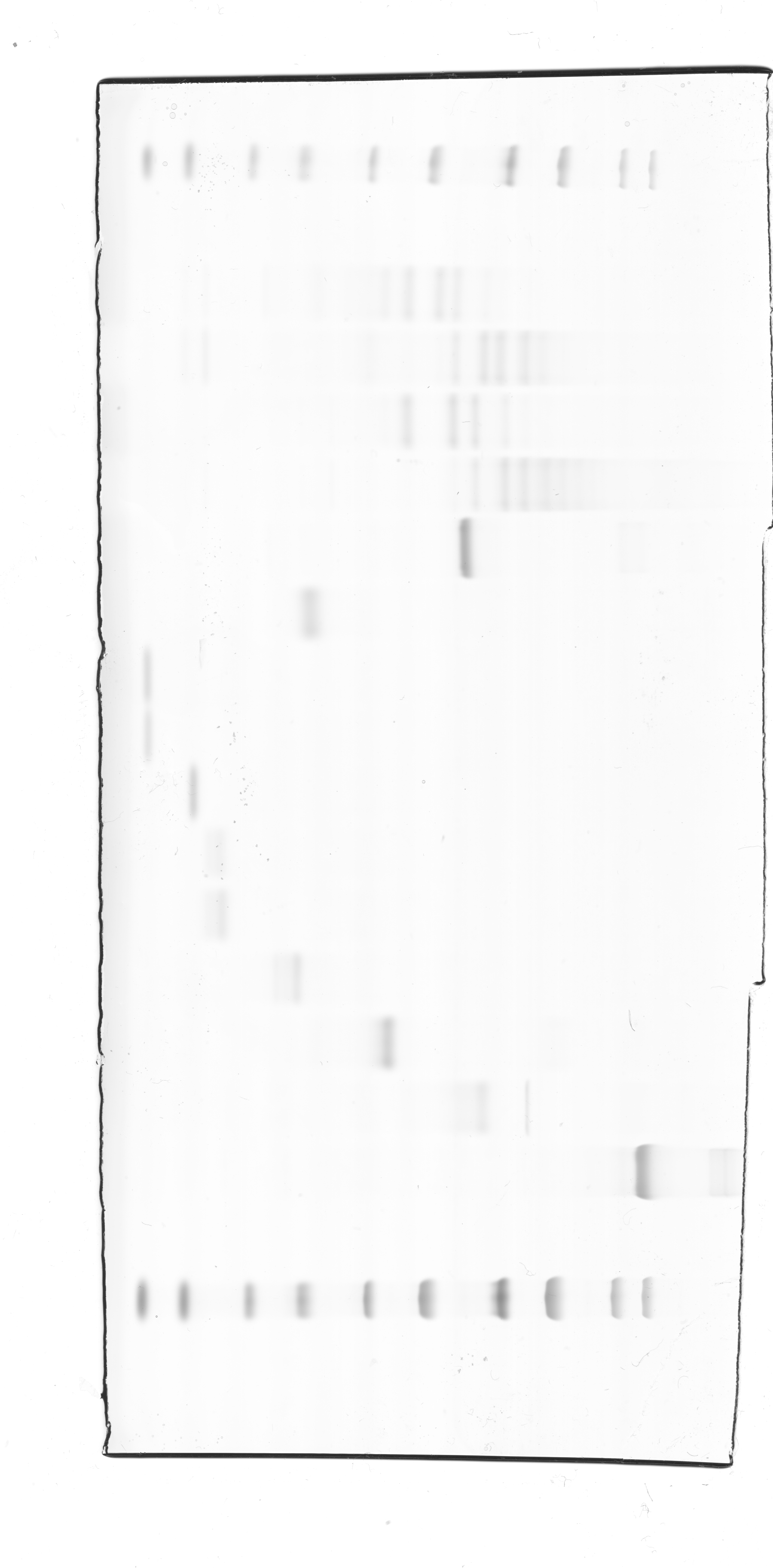

Supplement: Supplementary file 11 — Source Data [file 41467_2025_61224_MOESM11_ESM.zip › Source data/Uncropped scans of all blots and gels/Supplementary Fig. 1/Supplementary Fig. 1a/Left_Greyscales.png]

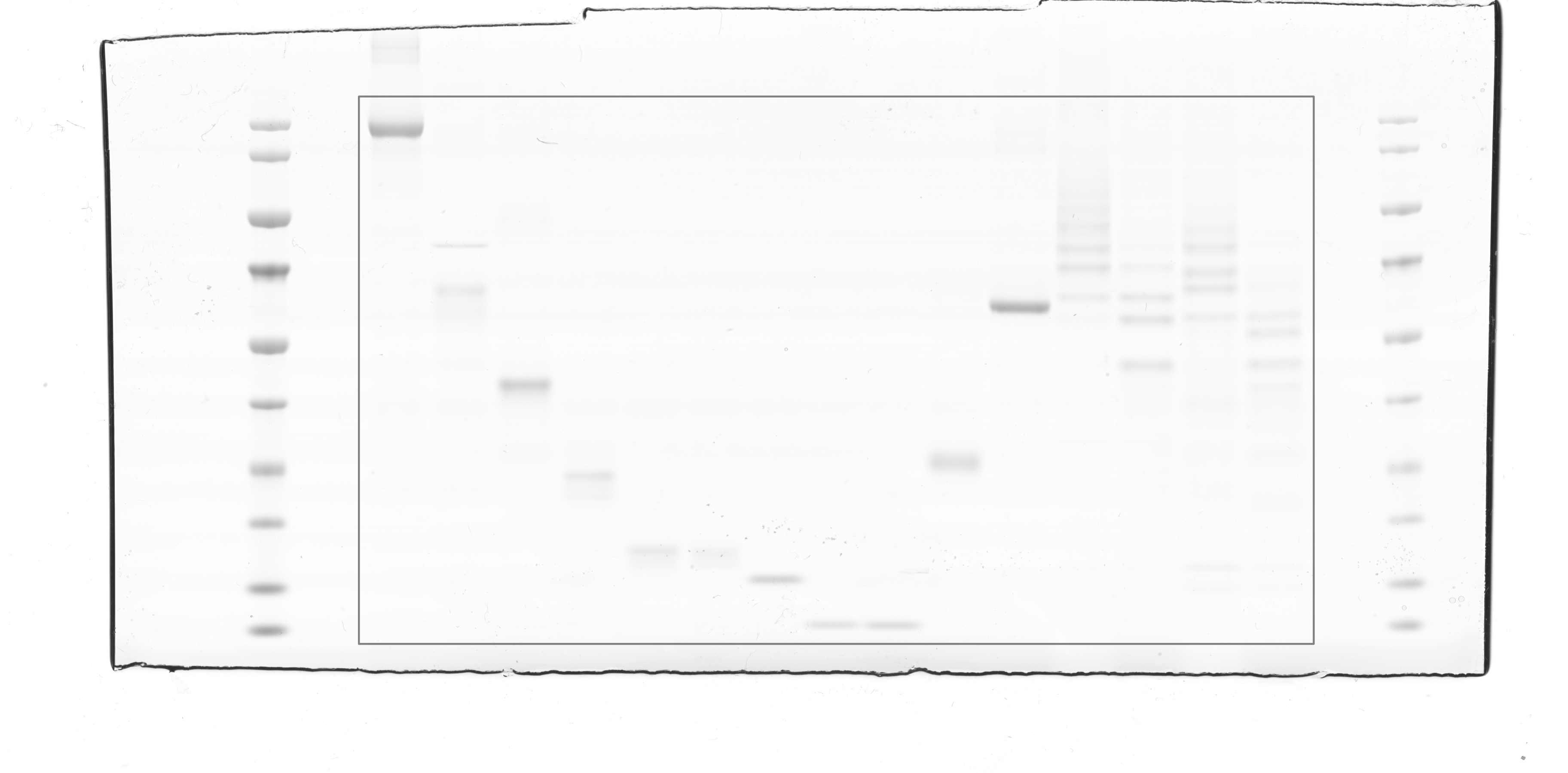

Supplement: Supplementary file 11 — Source Data [file 41467_2025_61224_MOESM11_ESM.zip › Source data/Uncropped scans of all blots and gels/Supplementary Fig. 1/Supplementary Fig. 1a/Left_Greyscales_label.tiff]

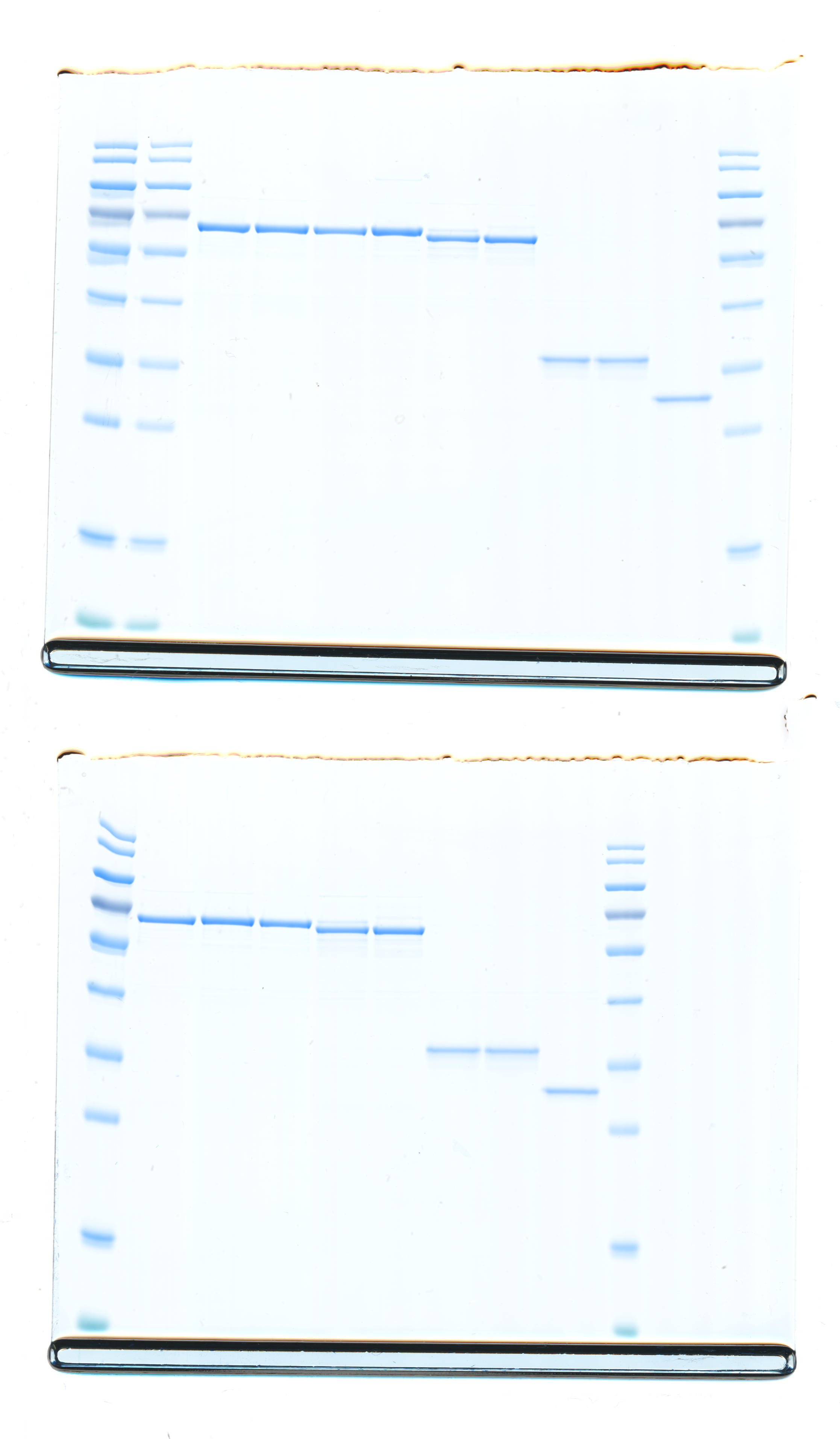

Supplement: Supplementary file 11 — Source Data [file 41467_2025_61224_MOESM11_ESM.zip › Source data/Uncropped scans of all blots and gels/Supplementary Fig. 1/Supplementary Fig. 1a/Right_Coomassie.png]

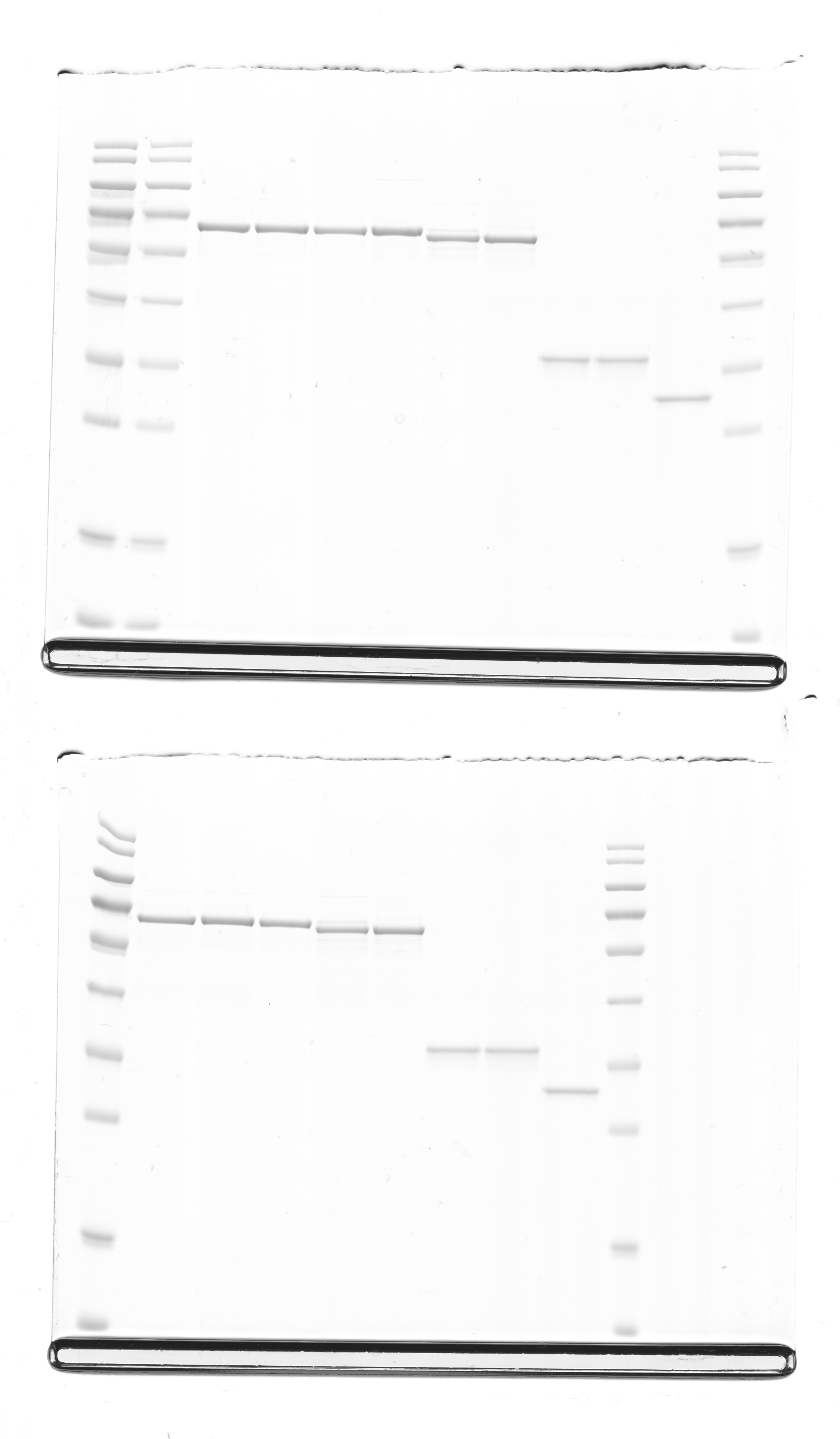

Supplement: Supplementary file 11 — Source Data [file 41467_2025_61224_MOESM11_ESM.zip › Source data/Uncropped scans of all blots and gels/Supplementary Fig. 1/Supplementary Fig. 1a/Right_Greyscales.png]

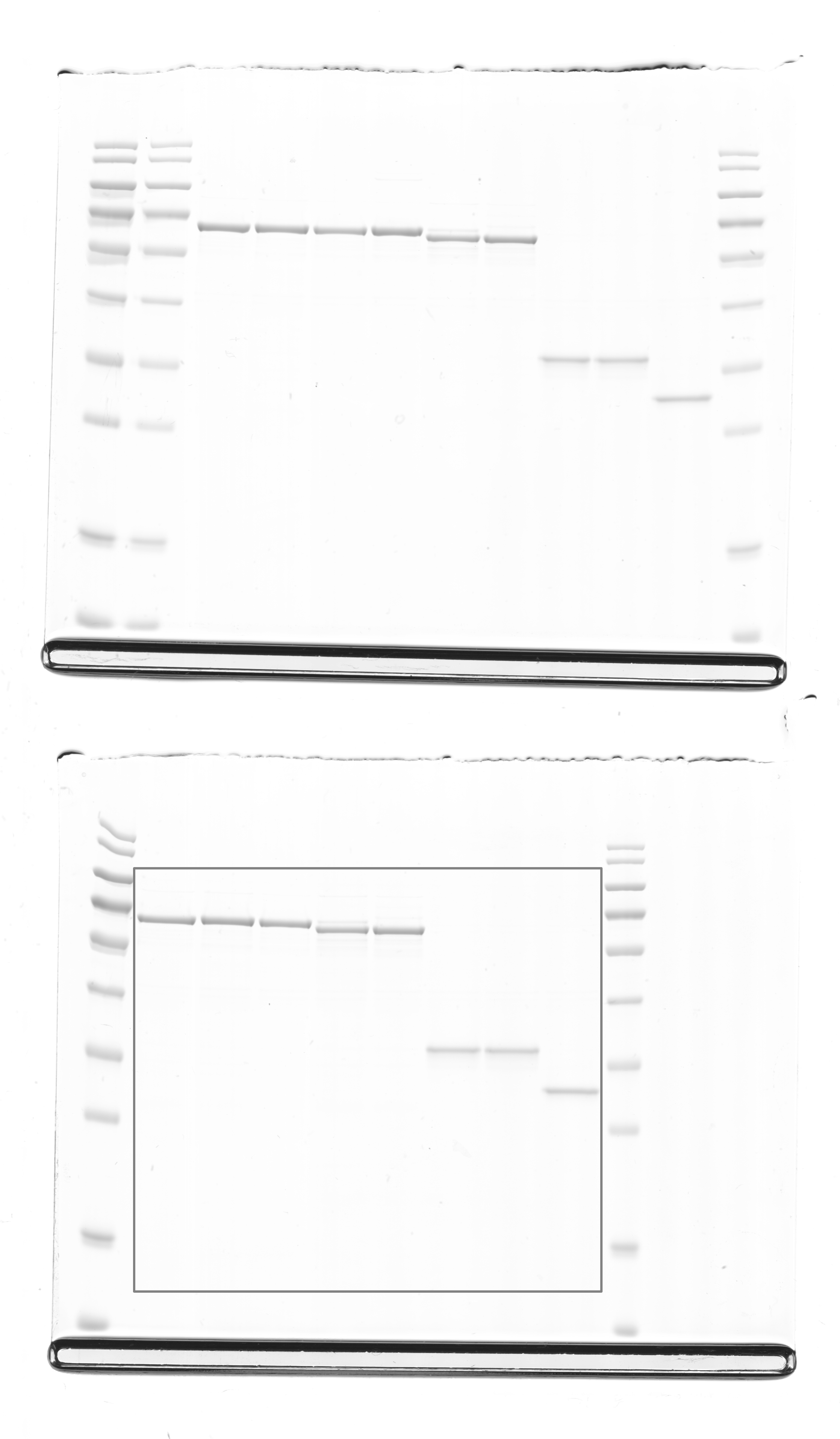

Supplement: Supplementary file 11 — Source Data [file 41467_2025_61224_MOESM11_ESM.zip › Source data/Uncropped scans of all blots and gels/Supplementary Fig. 1/Supplementary Fig. 1a/Right_Greyscales_label.tiff]

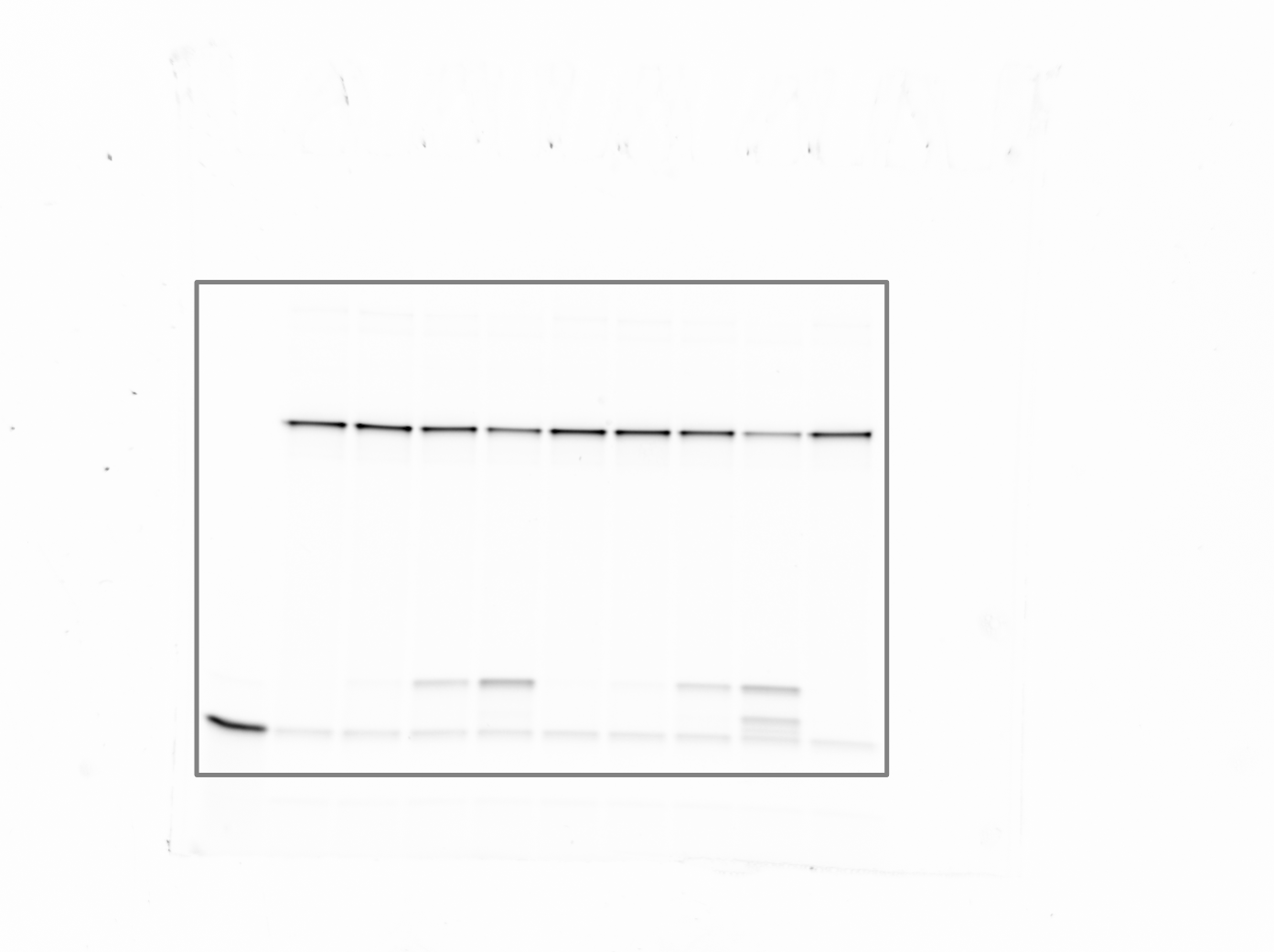

Supplement: Supplementary file 11 — Source Data [file 41467_2025_61224_MOESM11_ESM.zip › Source data/Uncropped scans of all blots and gels/Supplementary Fig. 1/Supplementary Fig. 1b/K48_Cy5_label.tiff]

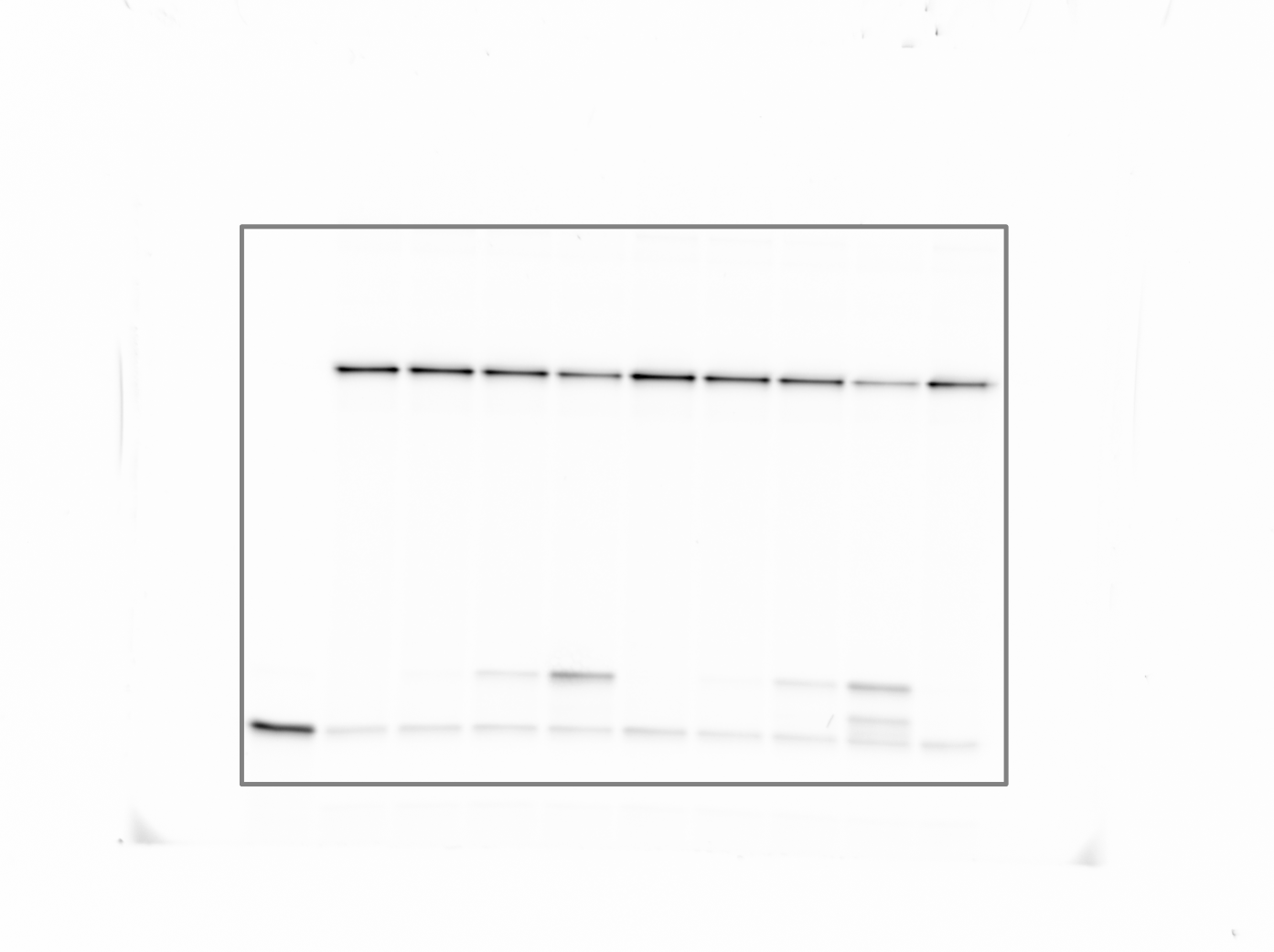

Supplement: Supplementary file 11 — Source Data [file 41467_2025_61224_MOESM11_ESM.zip › Source data/Uncropped scans of all blots and gels/Supplementary Fig. 1/Supplementary Fig. 1b/K63_Cy5_label.tiff]

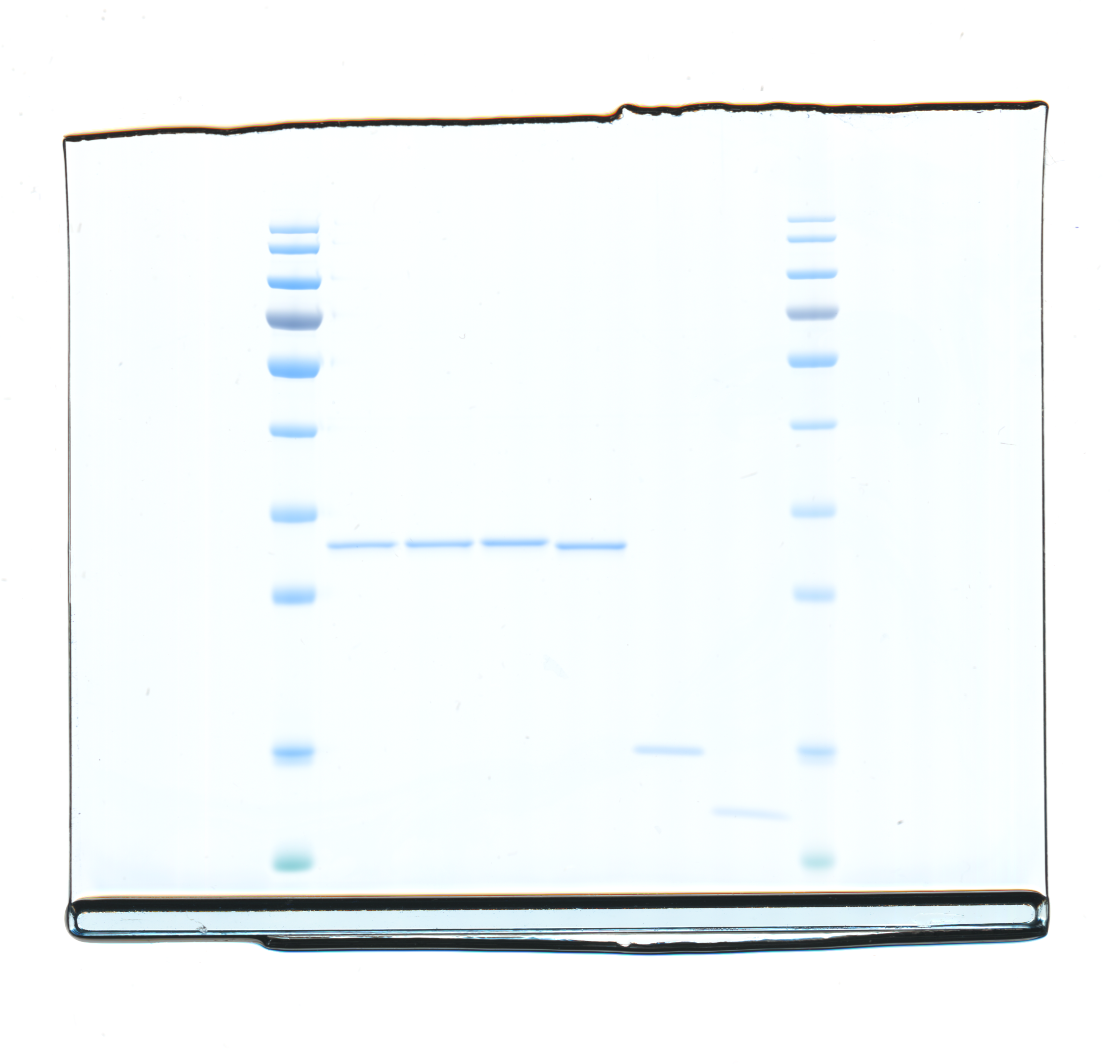

Supplement: Supplementary file 11 — Source Data [file 41467_2025_61224_MOESM11_ESM.zip › Source data/Uncropped scans of all blots and gels/Supplementary Fig. 3/Supplementary Fig. 3a/Coomassie.png]

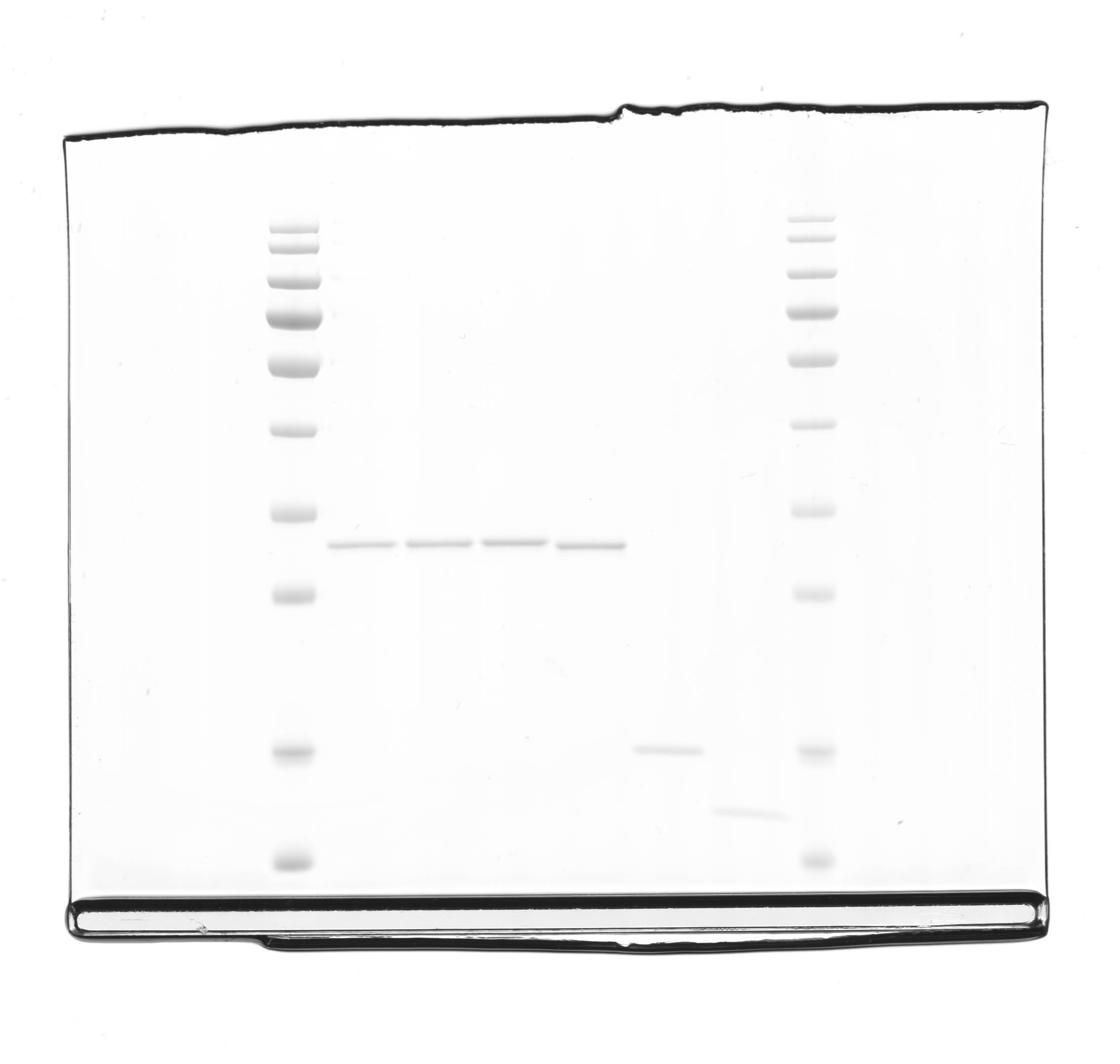

Supplement: Supplementary file 11 — Source Data [file 41467_2025_61224_MOESM11_ESM.zip › Source data/Uncropped scans of all blots and gels/Supplementary Fig. 3/Supplementary Fig. 3a/Greyscales.tif]

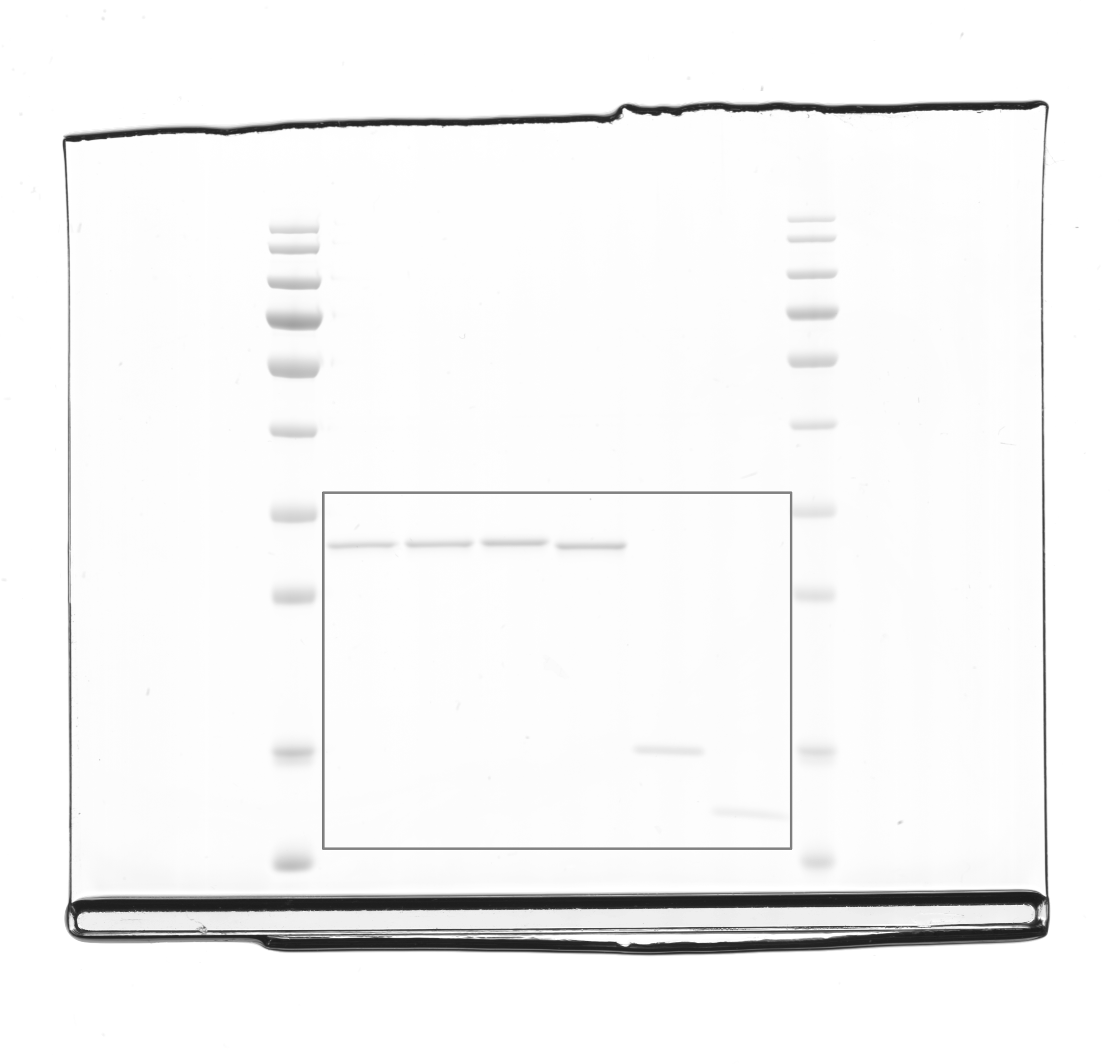

Supplement: Supplementary file 11 — Source Data [file 41467_2025_61224_MOESM11_ESM.zip › Source data/Uncropped scans of all blots and gels/Supplementary Fig. 3/Supplementary Fig. 3a/Greyscales_label.tiff]

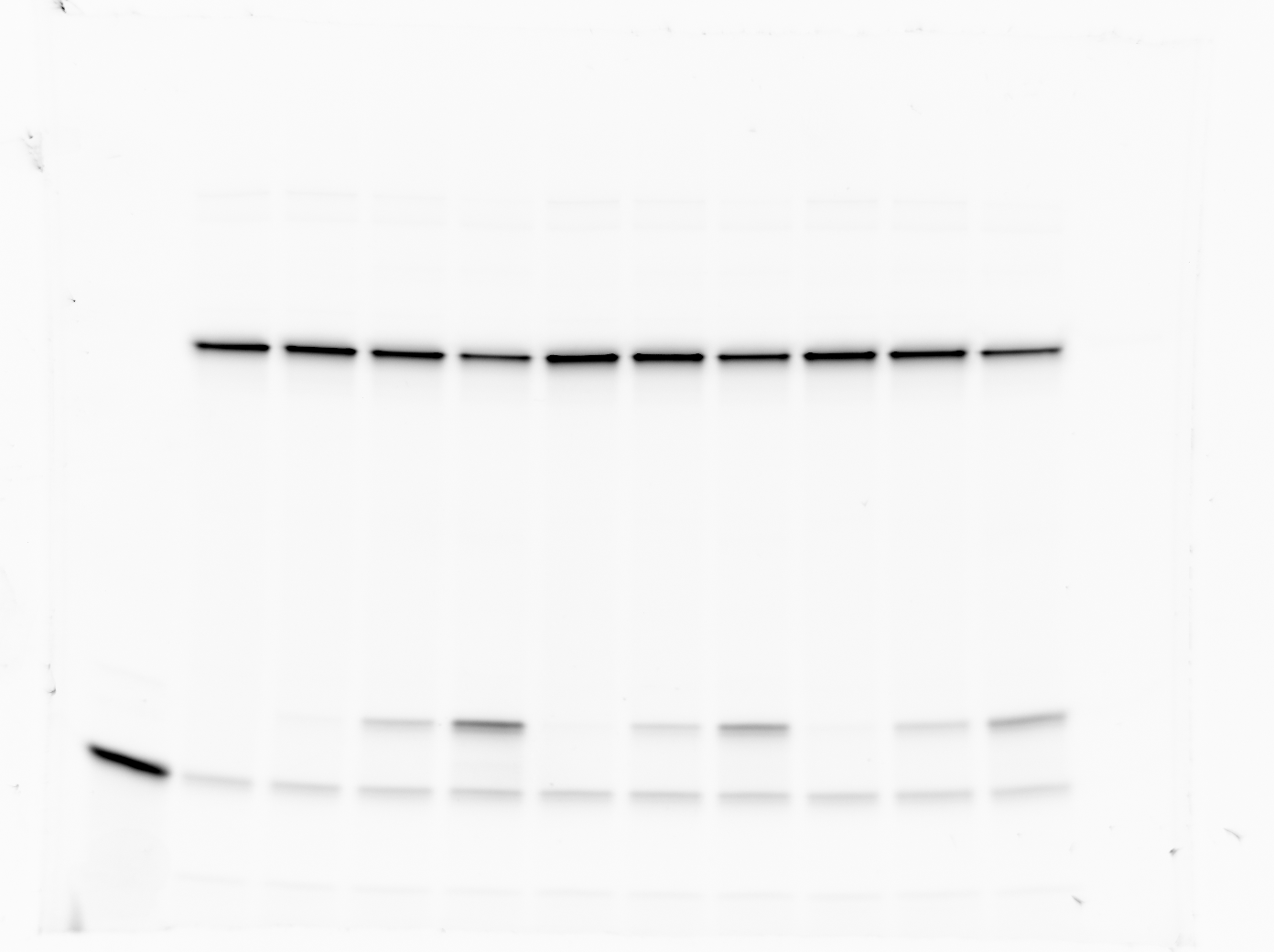

Supplement: Supplementary file 11 — Source Data [file 41467_2025_61224_MOESM11_ESM.zip › Source data/Uncropped scans of all blots and gels/Supplementary Fig. 5/Supplementary Fig. 5a/Cy5.tif]

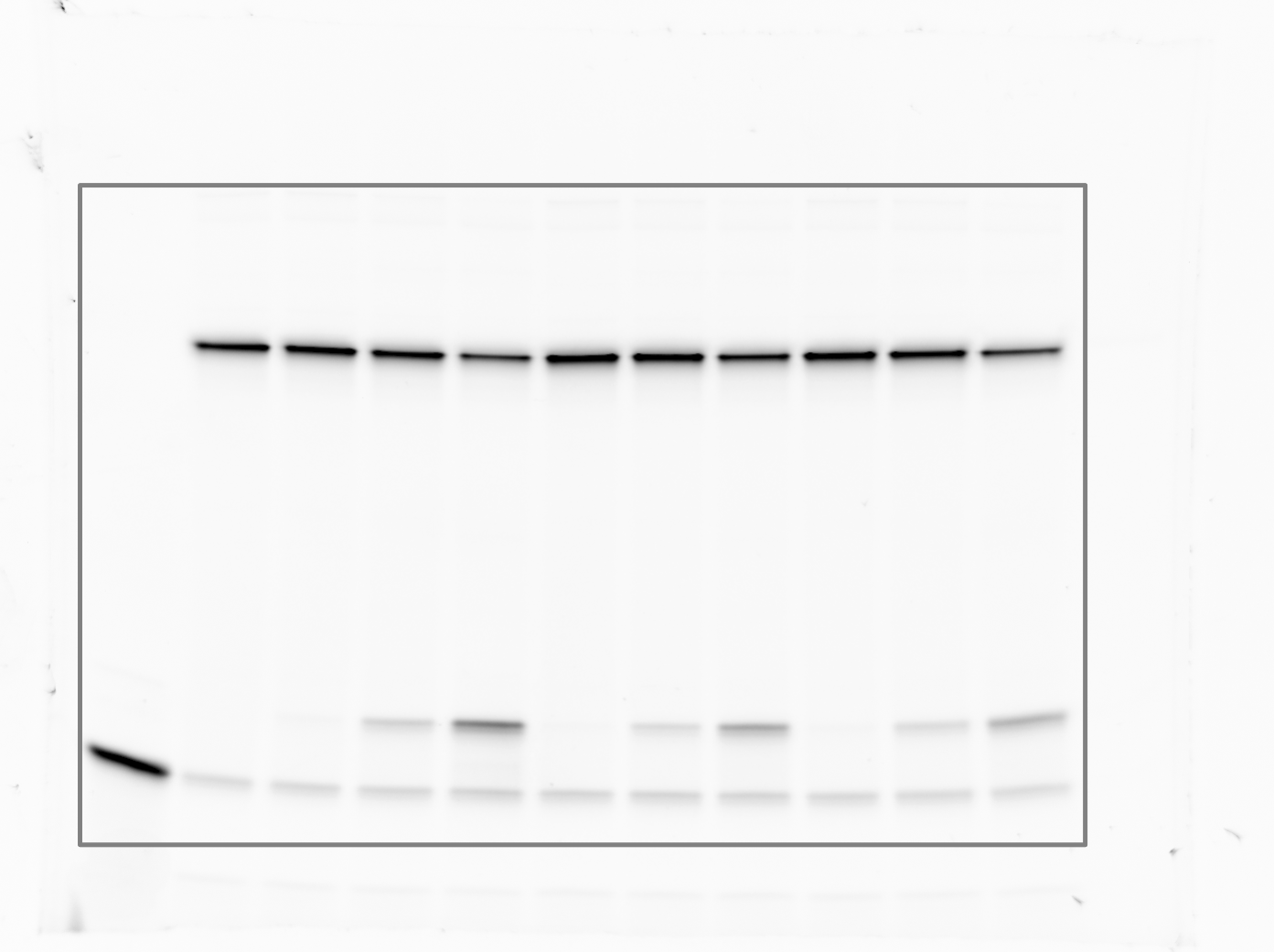

Supplement: Supplementary file 11 — Source Data [file 41467_2025_61224_MOESM11_ESM.zip › Source data/Uncropped scans of all blots and gels/Supplementary Fig. 5/Supplementary Fig. 5a/Cy5_label.tiff]

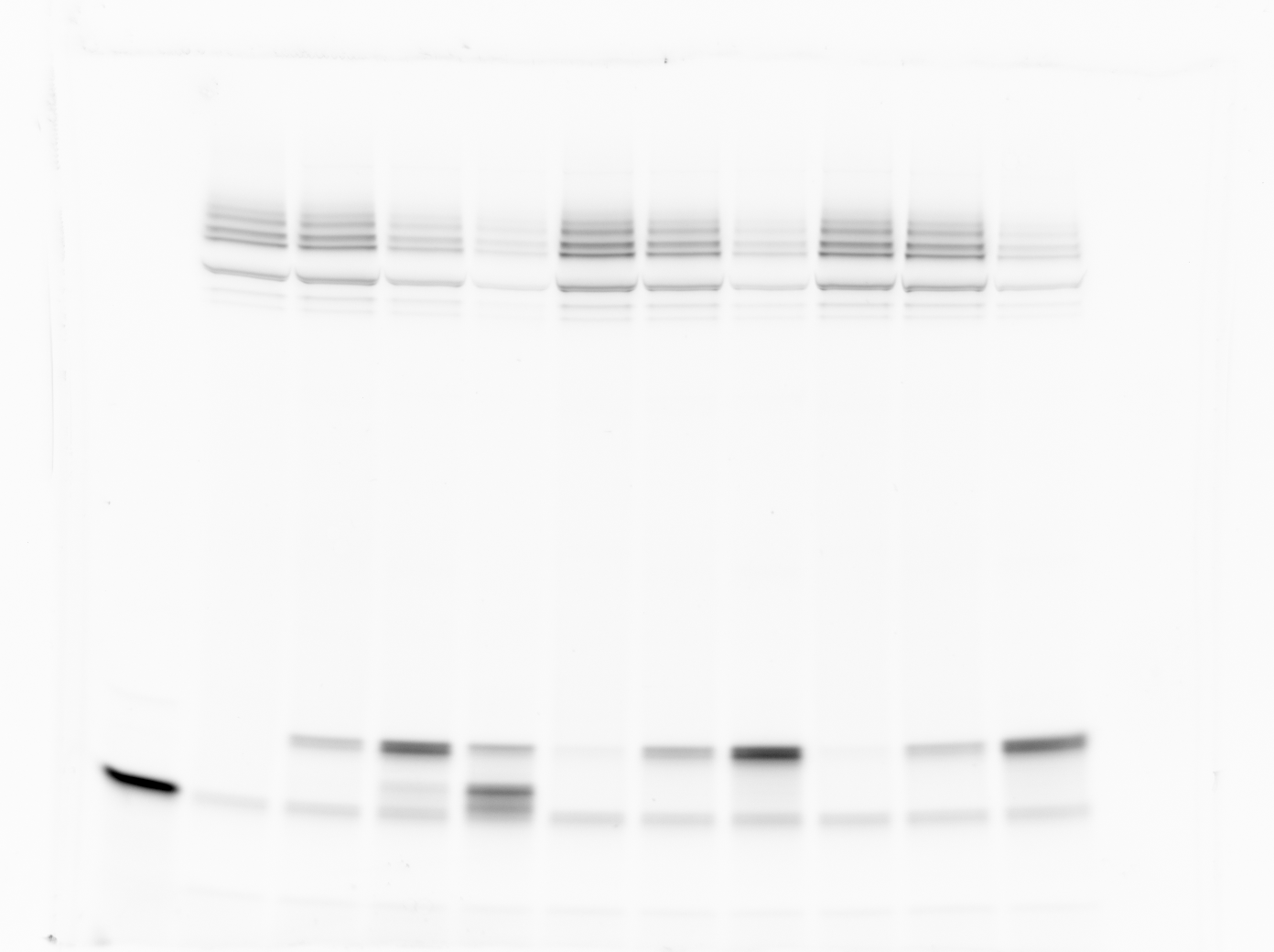

Supplement: Supplementary file 11 — Source Data [file 41467_2025_61224_MOESM11_ESM.zip › Source data/Uncropped scans of all blots and gels/Supplementary Fig. 5/Supplementary Fig. 5b/Cy5.tif]

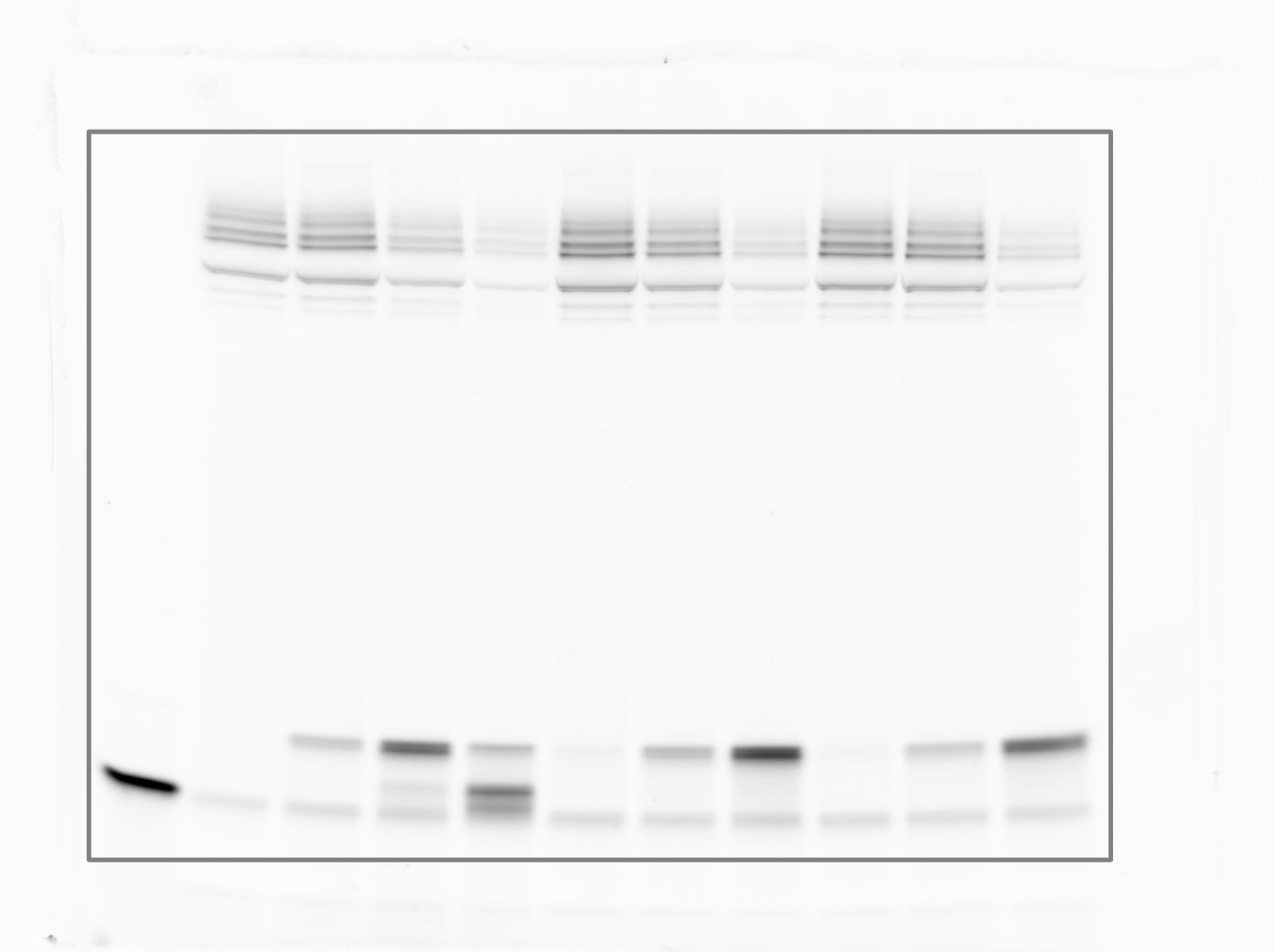

Supplement: Supplementary file 11 — Source Data [file 41467_2025_61224_MOESM11_ESM.zip › Source data/Uncropped scans of all blots and gels/Supplementary Fig. 5/Supplementary Fig. 5b/Cy5_label.tiff]

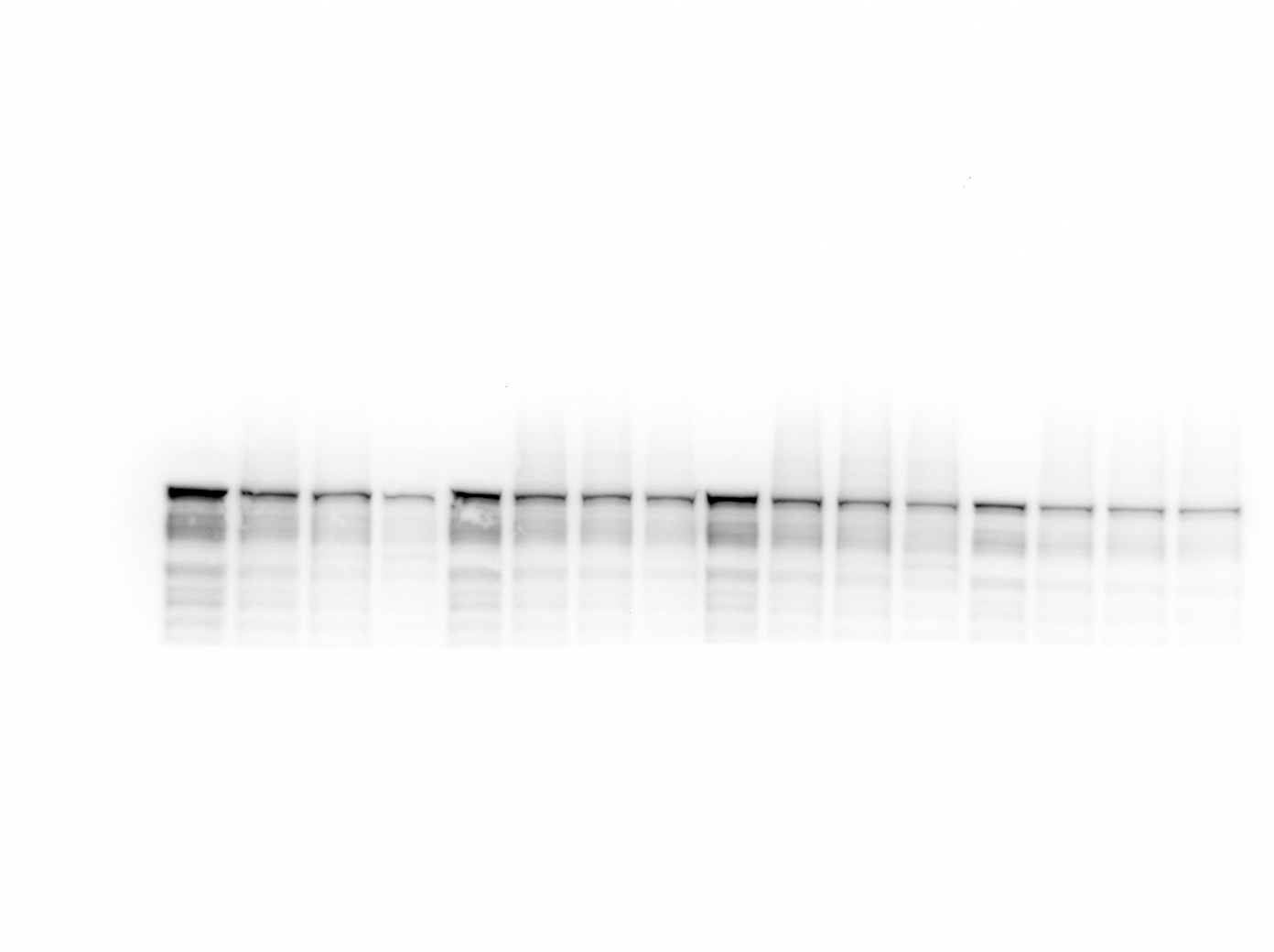

Supplement: Supplementary file 11 — Source Data [file 41467_2025_61224_MOESM11_ESM.zip › Source data/Uncropped scans of all blots and gels/Supplementary Fig. 6/Supplementary Fig. 6a/DNMT1/DNMT1.tif]

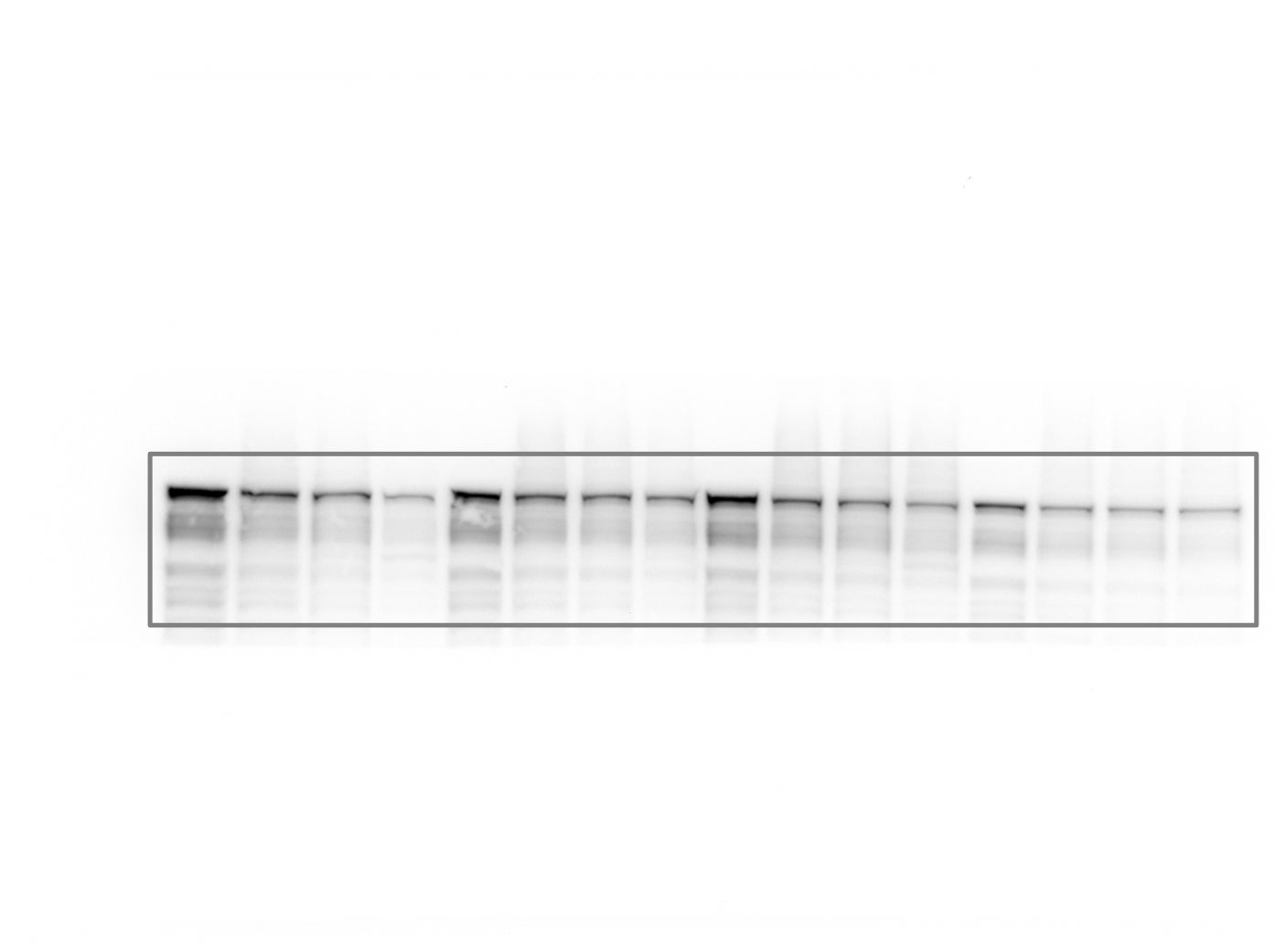

Supplement: Supplementary file 11 — Source Data [file 41467_2025_61224_MOESM11_ESM.zip › Source data/Uncropped scans of all blots and gels/Supplementary Fig. 6/Supplementary Fig. 6a/DNMT1/DNMT1_label.tiff]

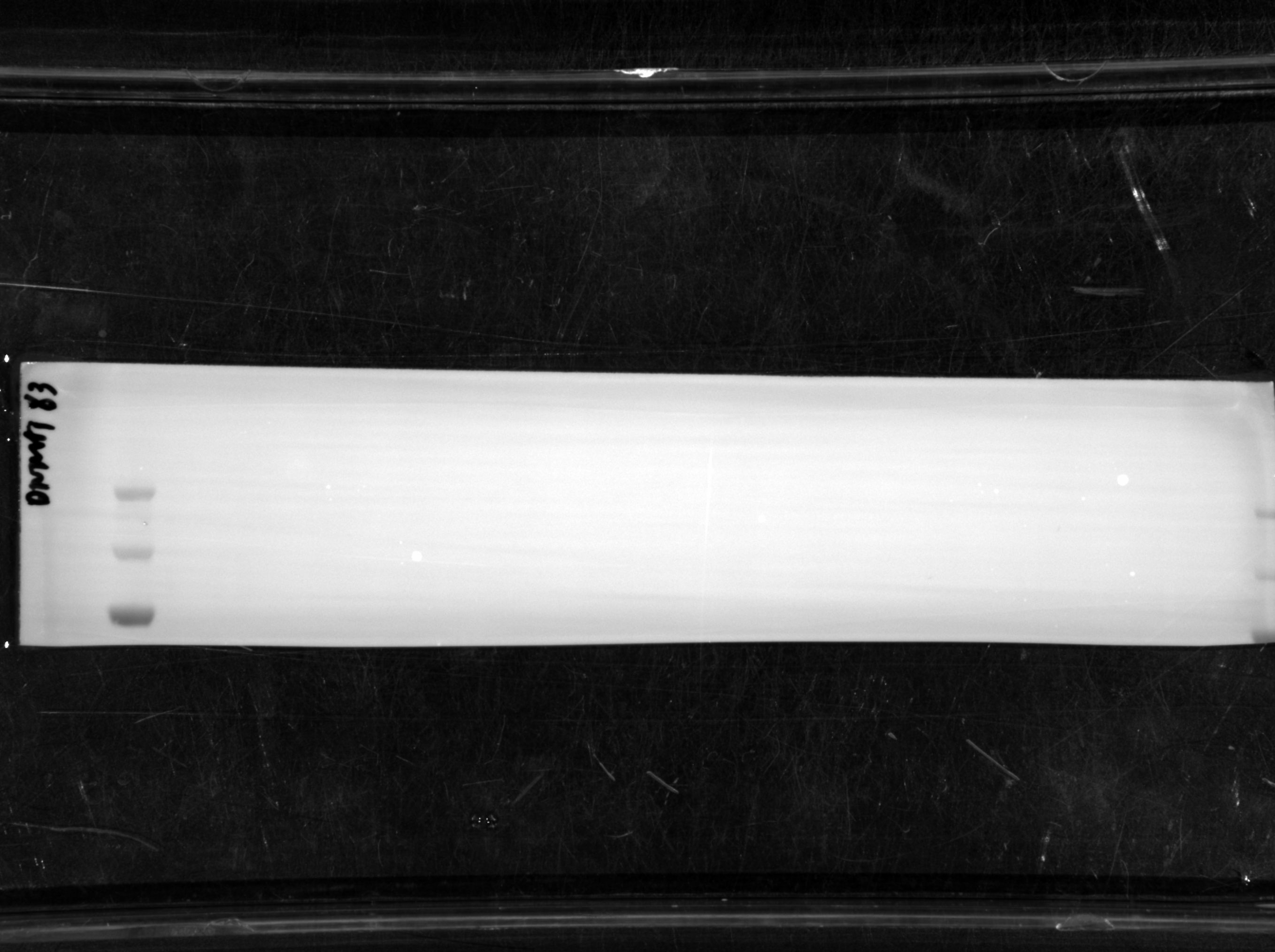

Supplement: Supplementary file 11 — Source Data [file 41467_2025_61224_MOESM11_ESM.zip › Source data/Uncropped scans of all blots and gels/Supplementary Fig. 6/Supplementary Fig. 6a/DNMT1/M.tif]

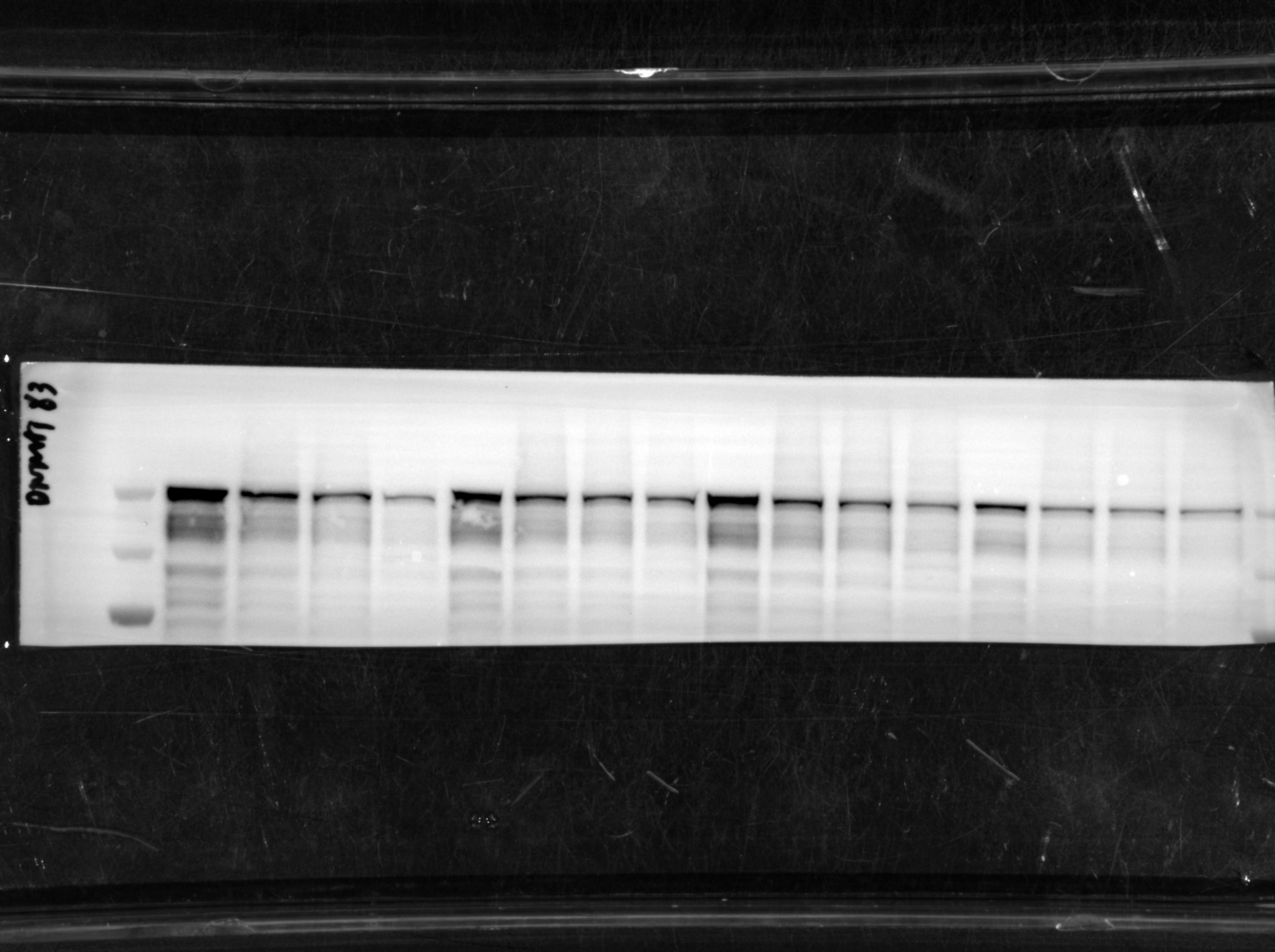

Supplement: Supplementary file 11 — Source Data [file 41467_2025_61224_MOESM11_ESM.zip › Source data/Uncropped scans of all blots and gels/Supplementary Fig. 6/Supplementary Fig. 6a/DNMT1/M+DNMT1.tif]

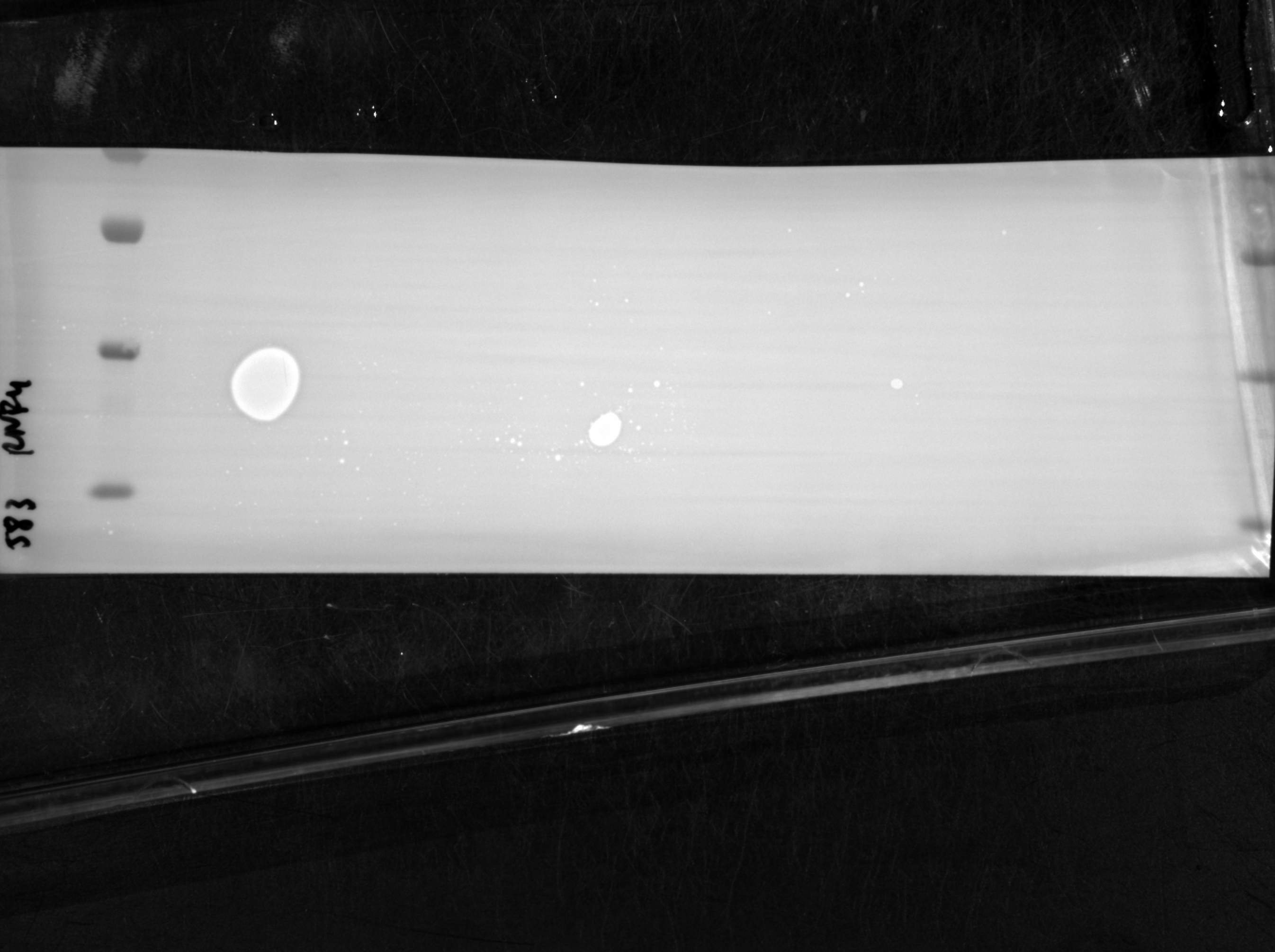

Supplement: Supplementary file 11 — Source Data [file 41467_2025_61224_MOESM11_ESM.zip › Source data/Uncropped scans of all blots and gels/Supplementary Fig. 6/Supplementary Fig. 6a/RNF4/M.tif]

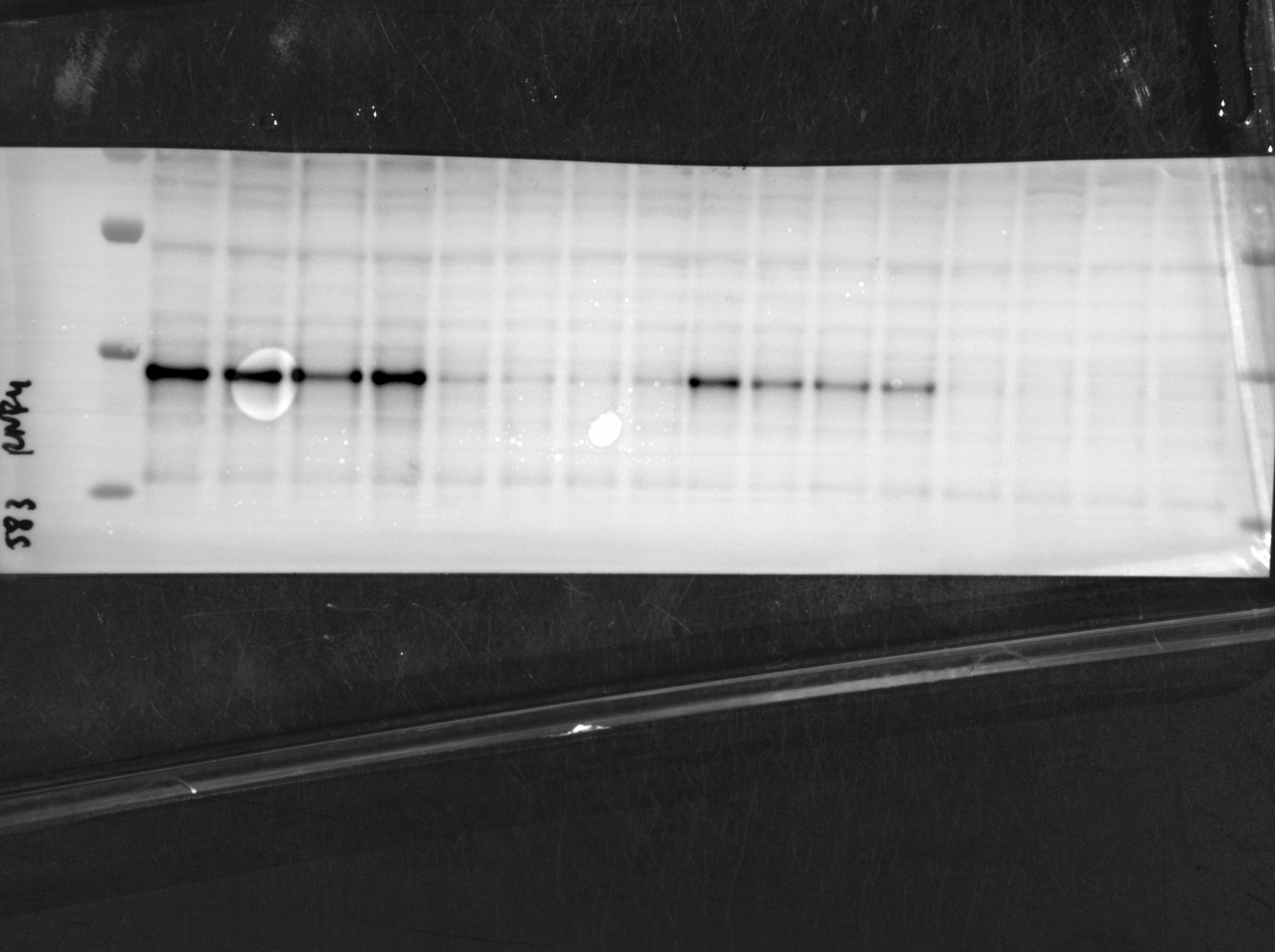

Supplement: Supplementary file 11 — Source Data [file 41467_2025_61224_MOESM11_ESM.zip › Source data/Uncropped scans of all blots and gels/Supplementary Fig. 6/Supplementary Fig. 6a/RNF4/M+RNF4.tif]

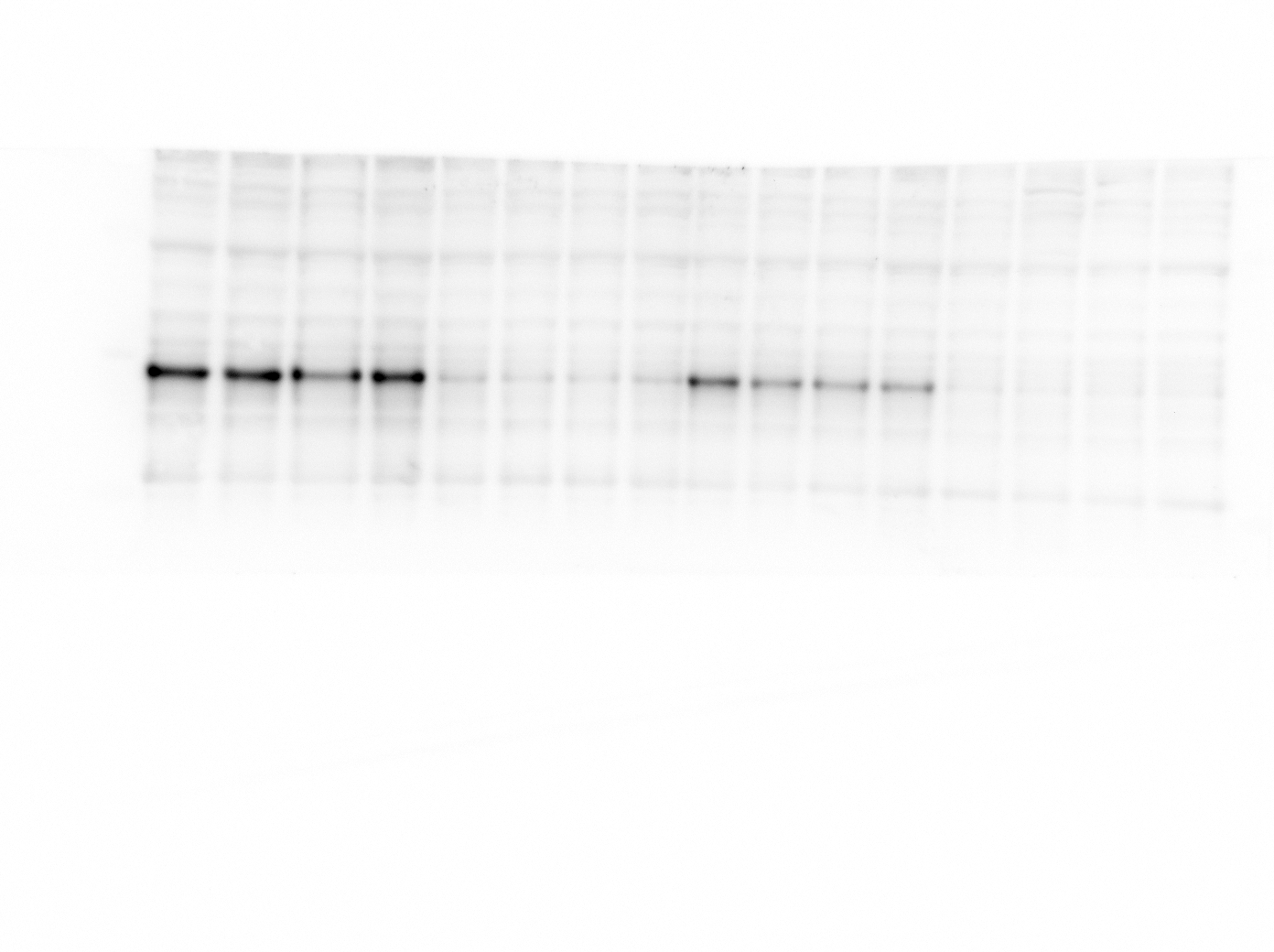

Supplement: Supplementary file 11 — Source Data [file 41467_2025_61224_MOESM11_ESM.zip › Source data/Uncropped scans of all blots and gels/Supplementary Fig. 6/Supplementary Fig. 6a/RNF4/RNF4.tif]

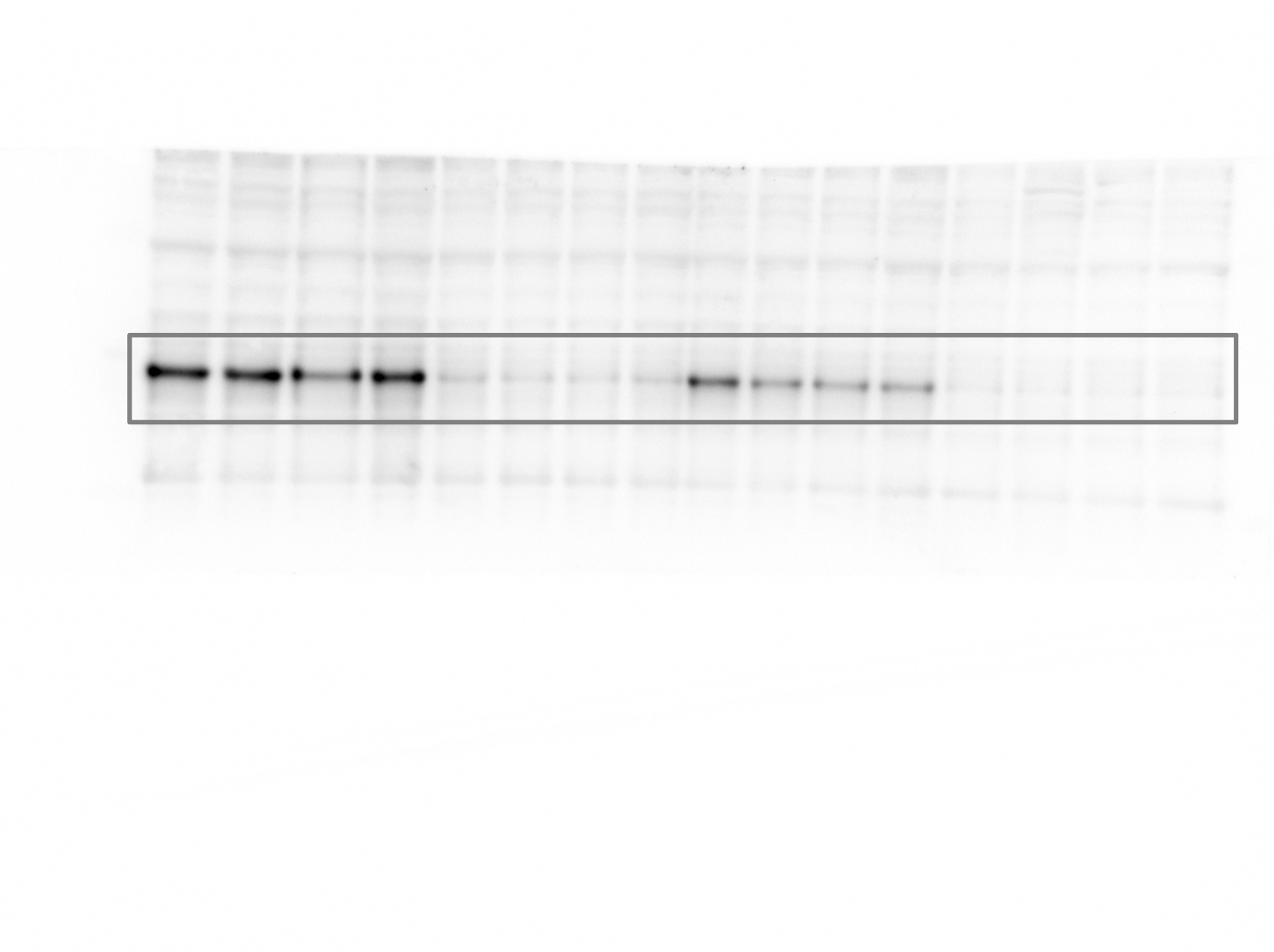

Supplement: Supplementary file 11 — Source Data [file 41467_2025_61224_MOESM11_ESM.zip › Source data/Uncropped scans of all blots and gels/Supplementary Fig. 6/Supplementary Fig. 6a/RNF4/RNF4_label.tiff]

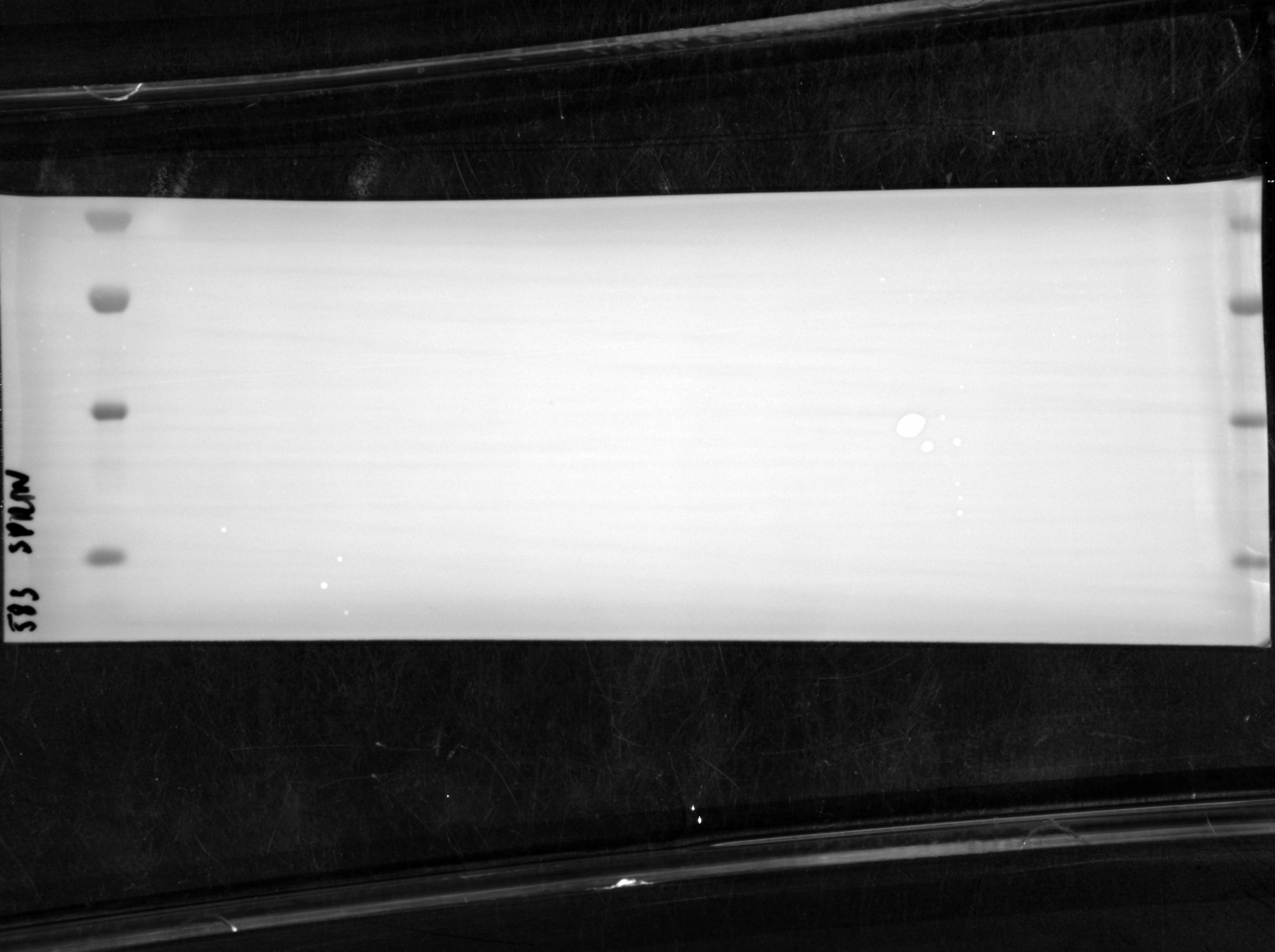

Supplement: Supplementary file 11 — Source Data [file 41467_2025_61224_MOESM11_ESM.zip › Source data/Uncropped scans of all blots and gels/Supplementary Fig. 6/Supplementary Fig. 6a/SPRTN/M.tif]

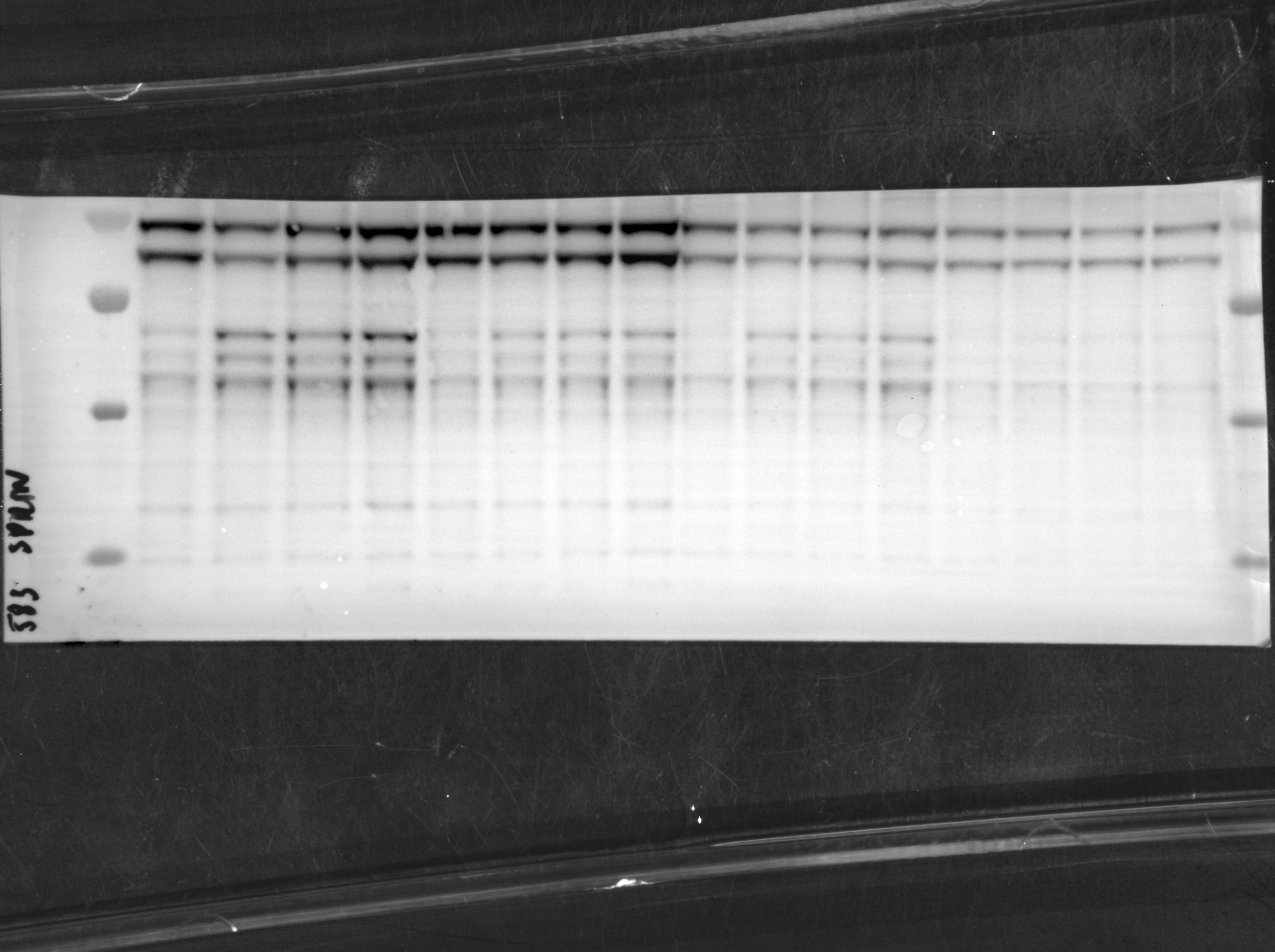

Supplement: Supplementary file 11 — Source Data [file 41467_2025_61224_MOESM11_ESM.zip › Source data/Uncropped scans of all blots and gels/Supplementary Fig. 6/Supplementary Fig. 6a/SPRTN/M+SPRTN.tif]

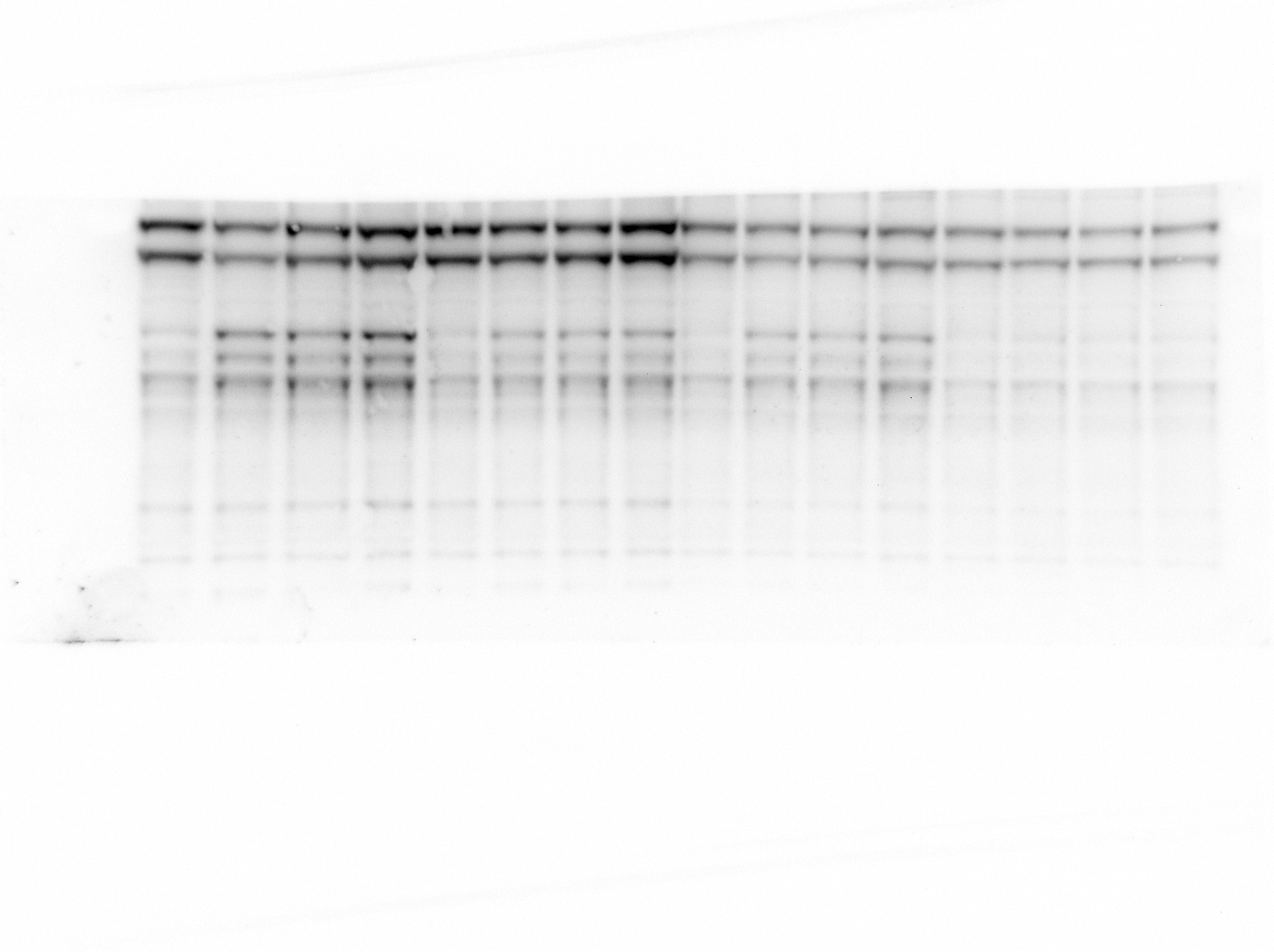

Supplement: Supplementary file 11 — Source Data [file 41467_2025_61224_MOESM11_ESM.zip › Source data/Uncropped scans of all blots and gels/Supplementary Fig. 6/Supplementary Fig. 6a/SPRTN/SPRTN.tif]

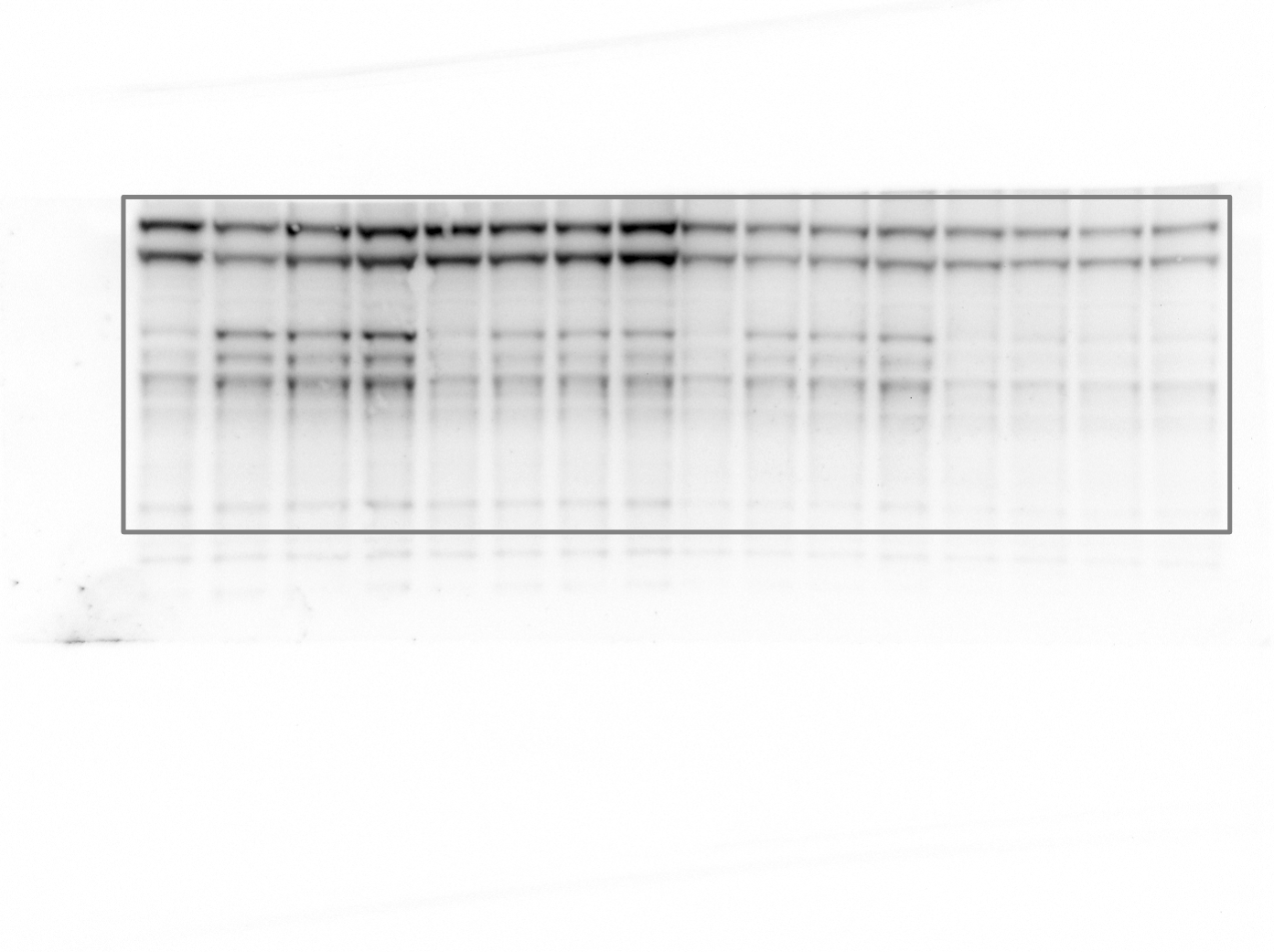

Supplement: Supplementary file 11 — Source Data [file 41467_2025_61224_MOESM11_ESM.zip › Source data/Uncropped scans of all blots and gels/Supplementary Fig. 6/Supplementary Fig. 6a/SPRTN/SPRTN_label.tiff]

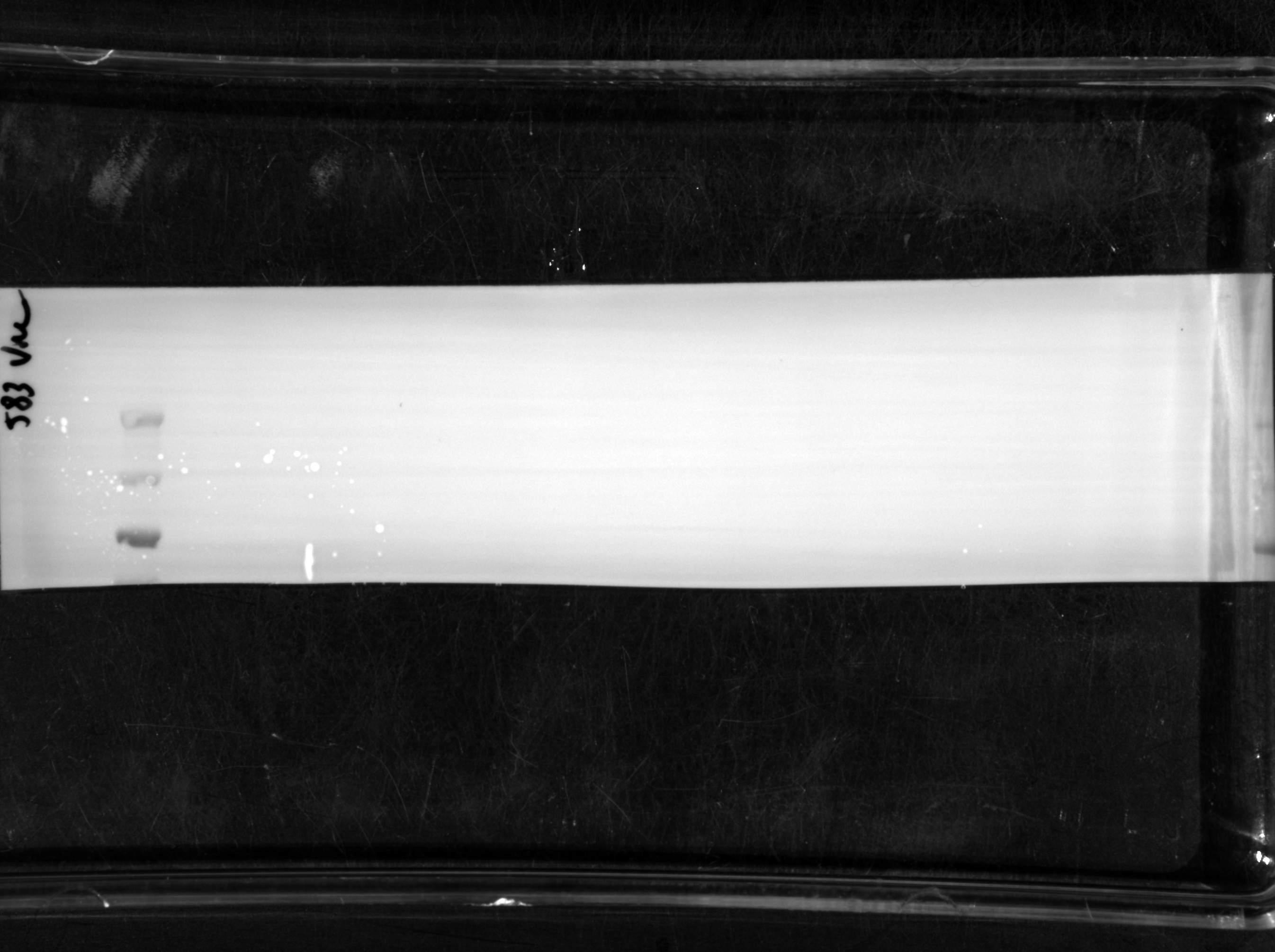

Supplement: Supplementary file 11 — Source Data [file 41467_2025_61224_MOESM11_ESM.zip › Source data/Uncropped scans of all blots and gels/Supplementary Fig. 6/Supplementary Fig. 6a/Vinculin/M.tif]

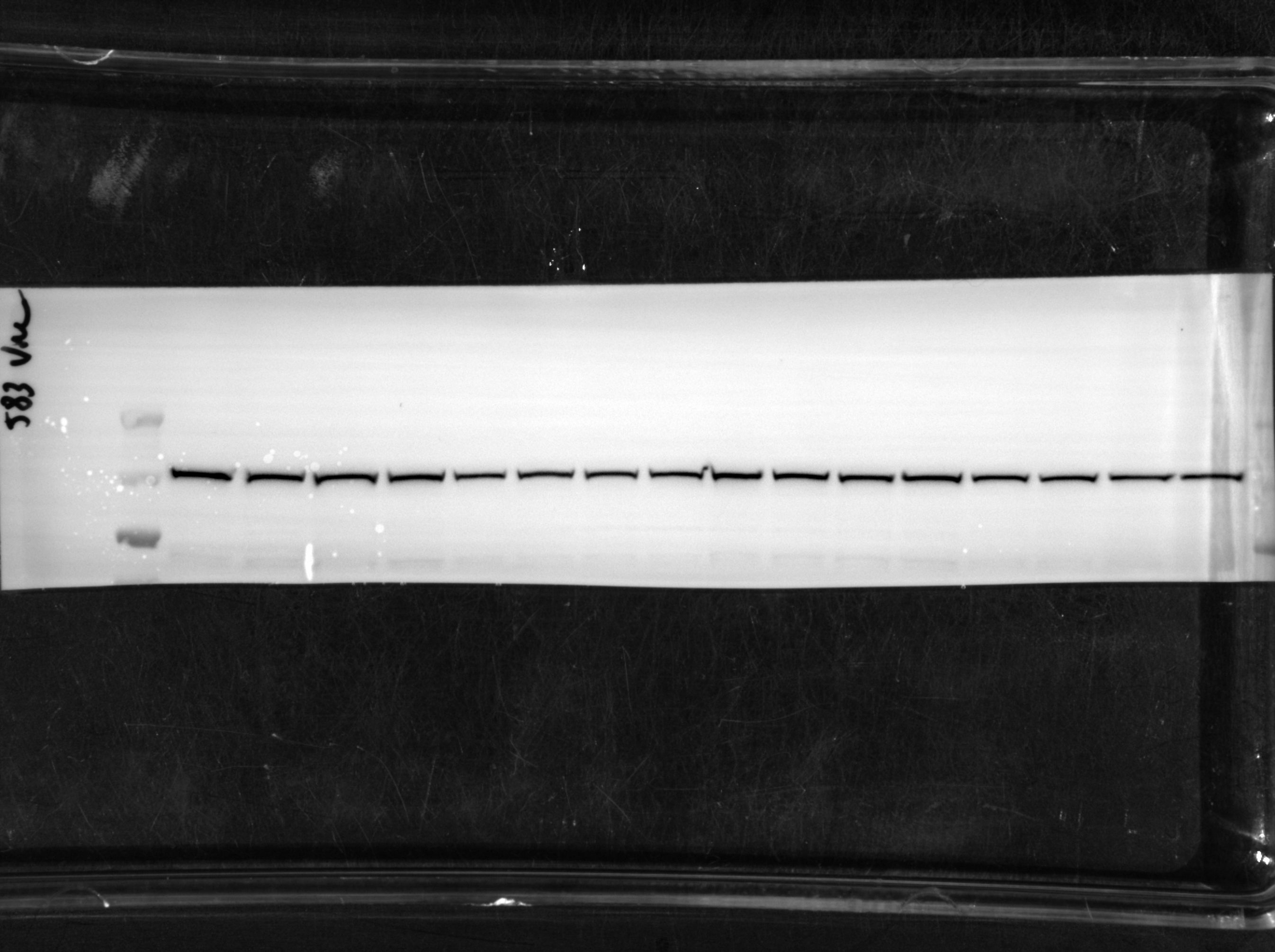

Supplement: Supplementary file 11 — Source Data [file 41467_2025_61224_MOESM11_ESM.zip › Source data/Uncropped scans of all blots and gels/Supplementary Fig. 6/Supplementary Fig. 6a/Vinculin/M+Vinculin.tif]

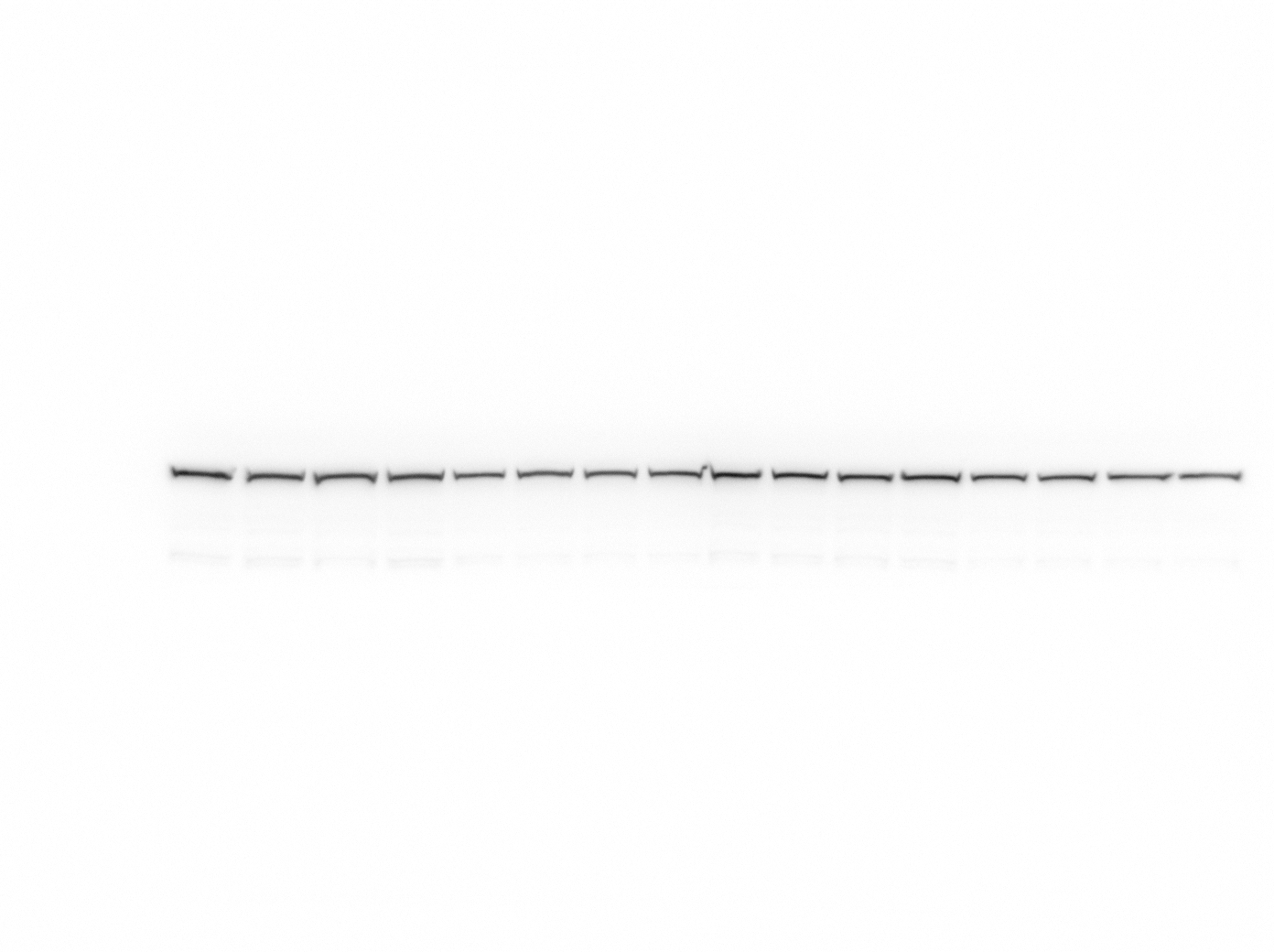

Supplement: Supplementary file 11 — Source Data [file 41467_2025_61224_MOESM11_ESM.zip › Source data/Uncropped scans of all blots and gels/Supplementary Fig. 6/Supplementary Fig. 6a/Vinculin/Vinculin.tif]

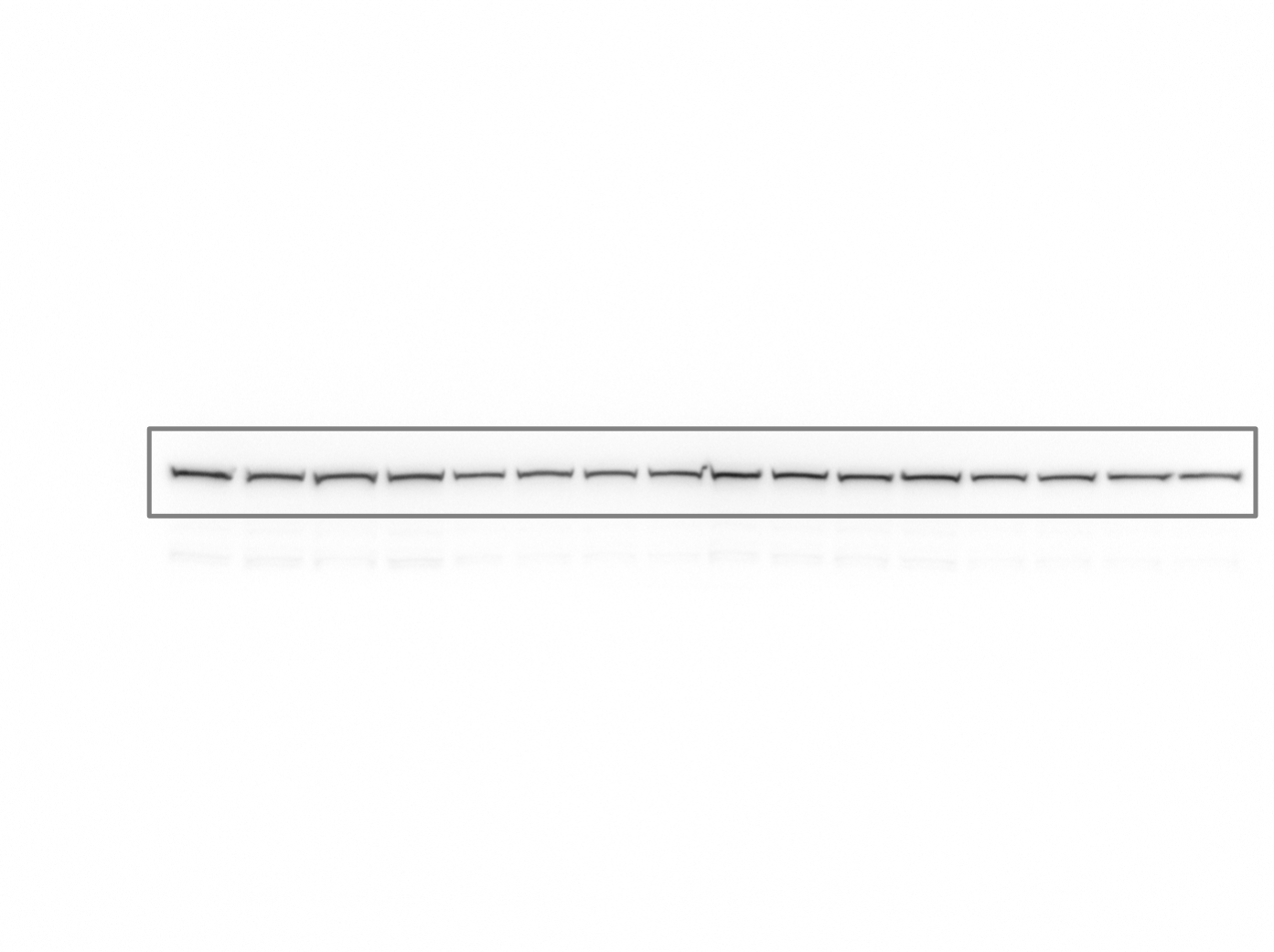

Supplement: Supplementary file 11 — Source Data [file 41467_2025_61224_MOESM11_ESM.zip › Source data/Uncropped scans of all blots and gels/Supplementary Fig. 6/Supplementary Fig. 6a/Vinculin/Vinculin_label.tiff]

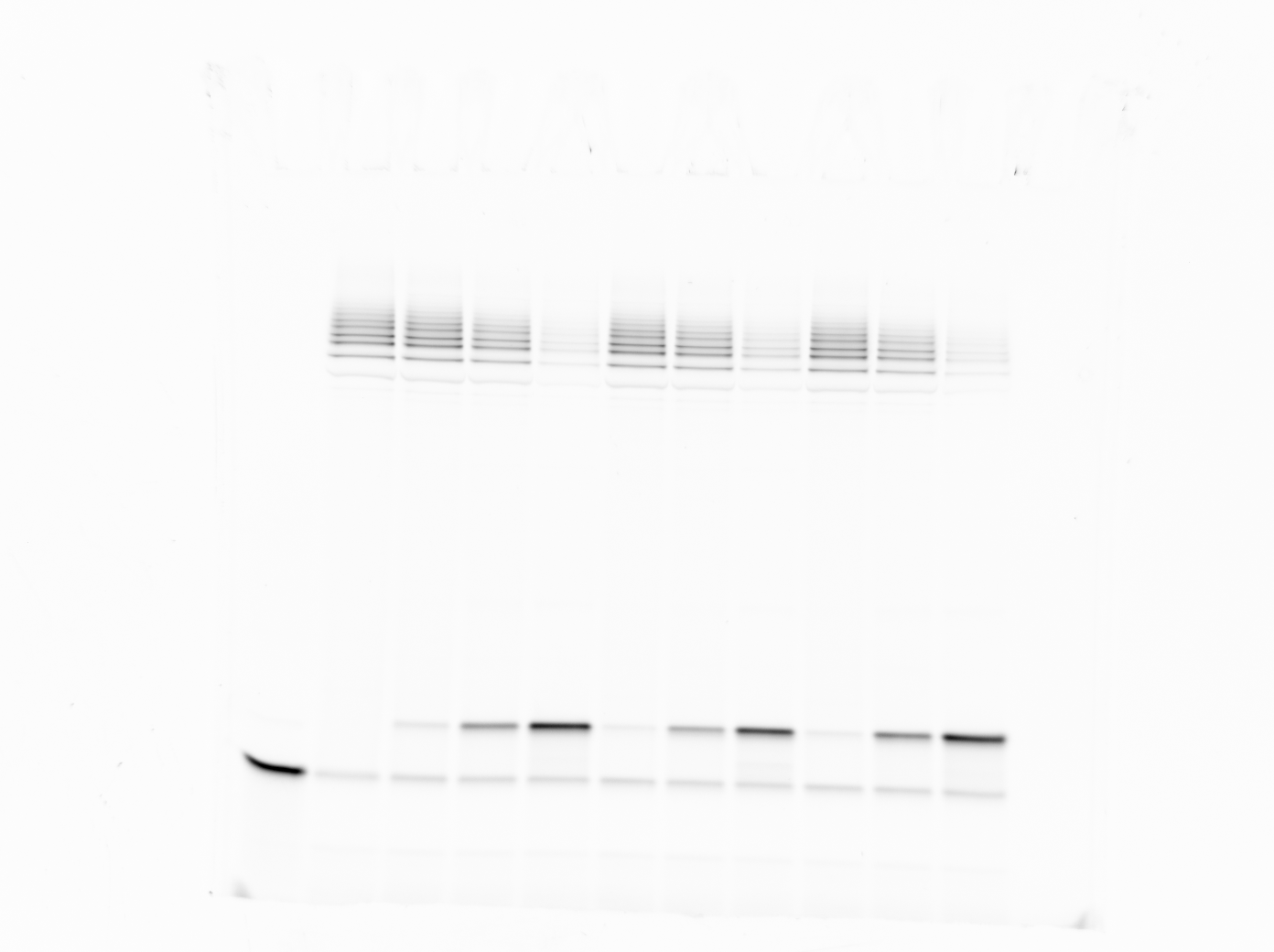

Supplement: Supplementary file 11 — Source Data [file 41467_2025_61224_MOESM11_ESM.zip › Source data/Uncropped scans of all blots and gels/Supplementary Fig. 6/Supplementary Fig. 6b/K48-L99S_Cy5.tif]

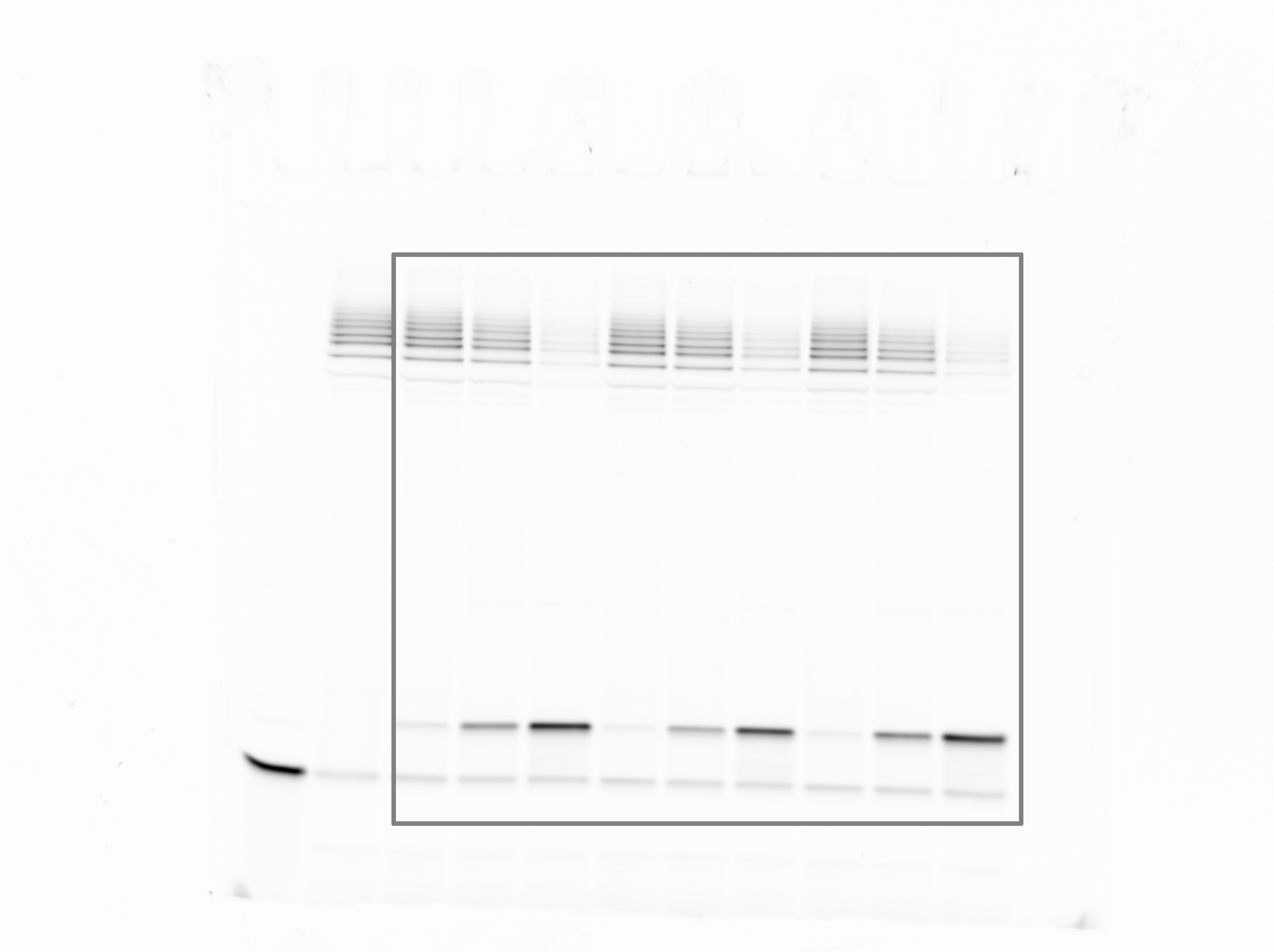

Supplement: Supplementary file 11 — Source Data [file 41467_2025_61224_MOESM11_ESM.zip › Source data/Uncropped scans of all blots and gels/Supplementary Fig. 6/Supplementary Fig. 6b/K48-L99S_Cy5_label.tiff]

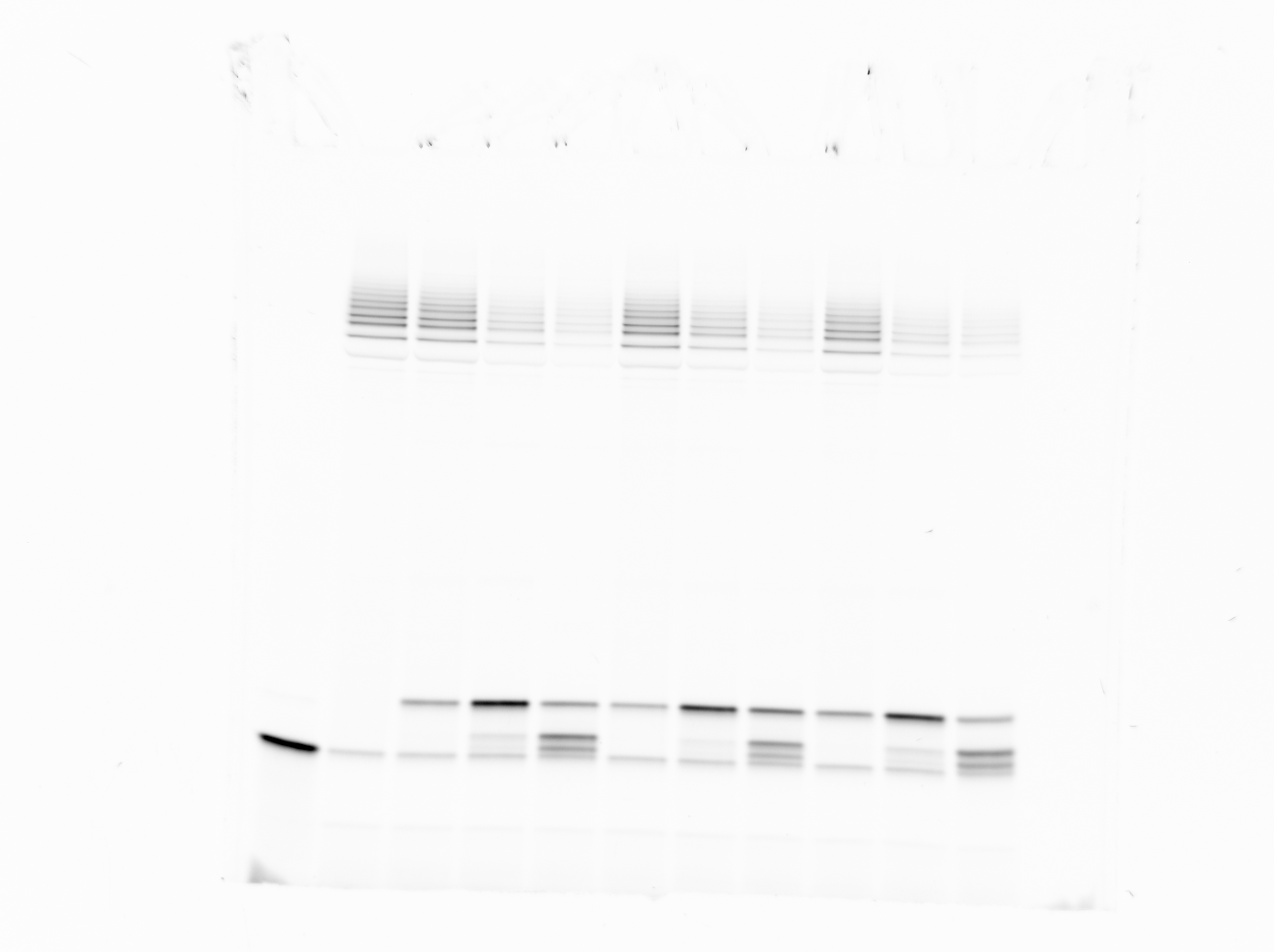

Supplement: Supplementary file 11 — Source Data [file 41467_2025_61224_MOESM11_ESM.zip › Source data/Uncropped scans of all blots and gels/Supplementary Fig. 6/Supplementary Fig. 6b/K48-WT_Cy5.tif]

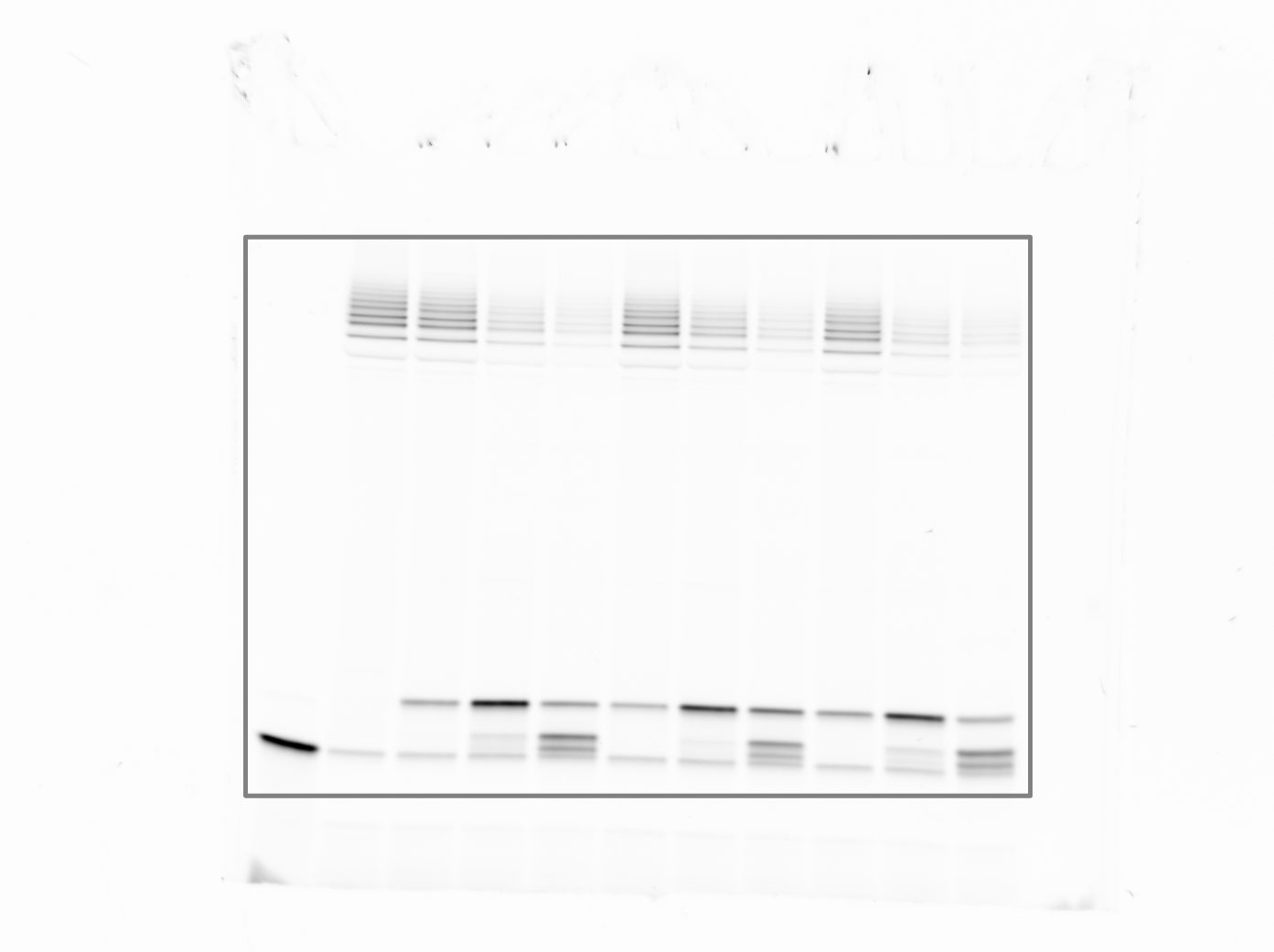

Supplement: Supplementary file 11 — Source Data [file 41467_2025_61224_MOESM11_ESM.zip › Source data/Uncropped scans of all blots and gels/Supplementary Fig. 6/Supplementary Fig. 6b/K48-WT_Cy5_label.tiff]

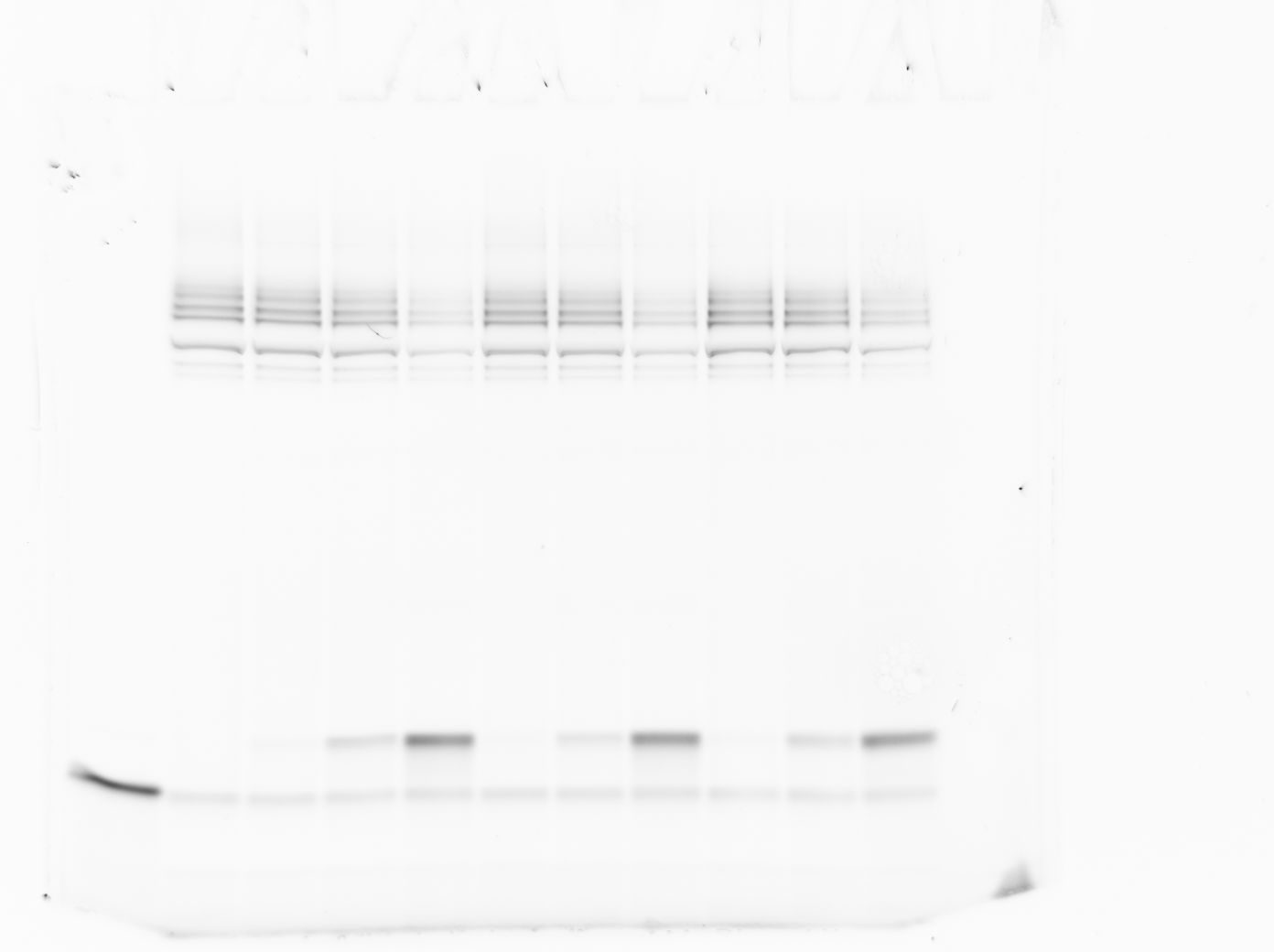

Supplement: Supplementary file 11 — Source Data [file 41467_2025_61224_MOESM11_ESM.zip › Source data/Uncropped scans of all blots and gels/Supplementary Fig. 6/Supplementary Fig. 6b/K63-L99S_Cy5.tif]

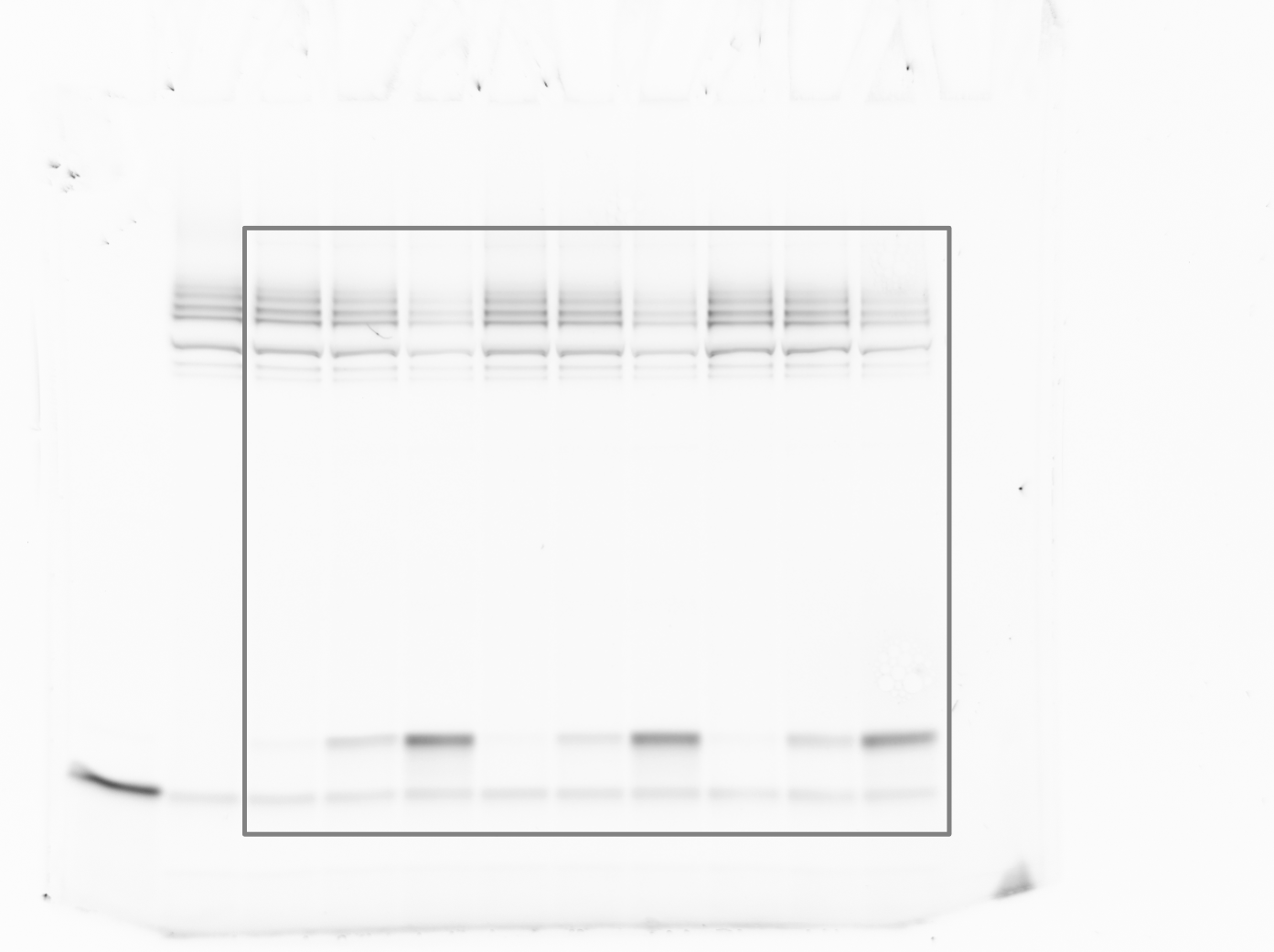

Supplement: Supplementary file 11 — Source Data [file 41467_2025_61224_MOESM11_ESM.zip › Source data/Uncropped scans of all blots and gels/Supplementary Fig. 6/Supplementary Fig. 6b/K63-L99S_Cy5_label.tiff]

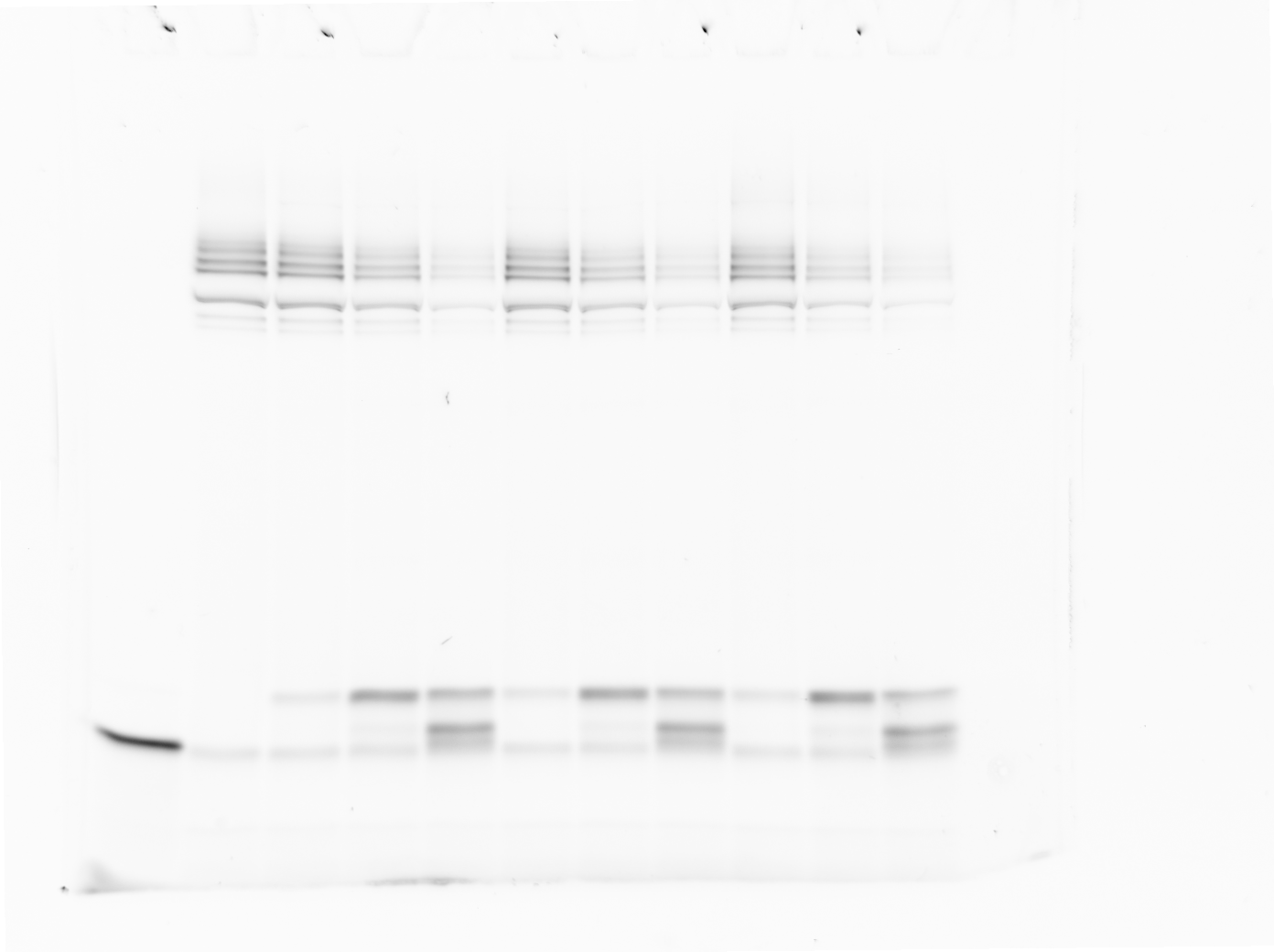

Supplement: Supplementary file 11 — Source Data [file 41467_2025_61224_MOESM11_ESM.zip › Source data/Uncropped scans of all blots and gels/Supplementary Fig. 6/Supplementary Fig. 6b/K63-WT_Cy5.tif]

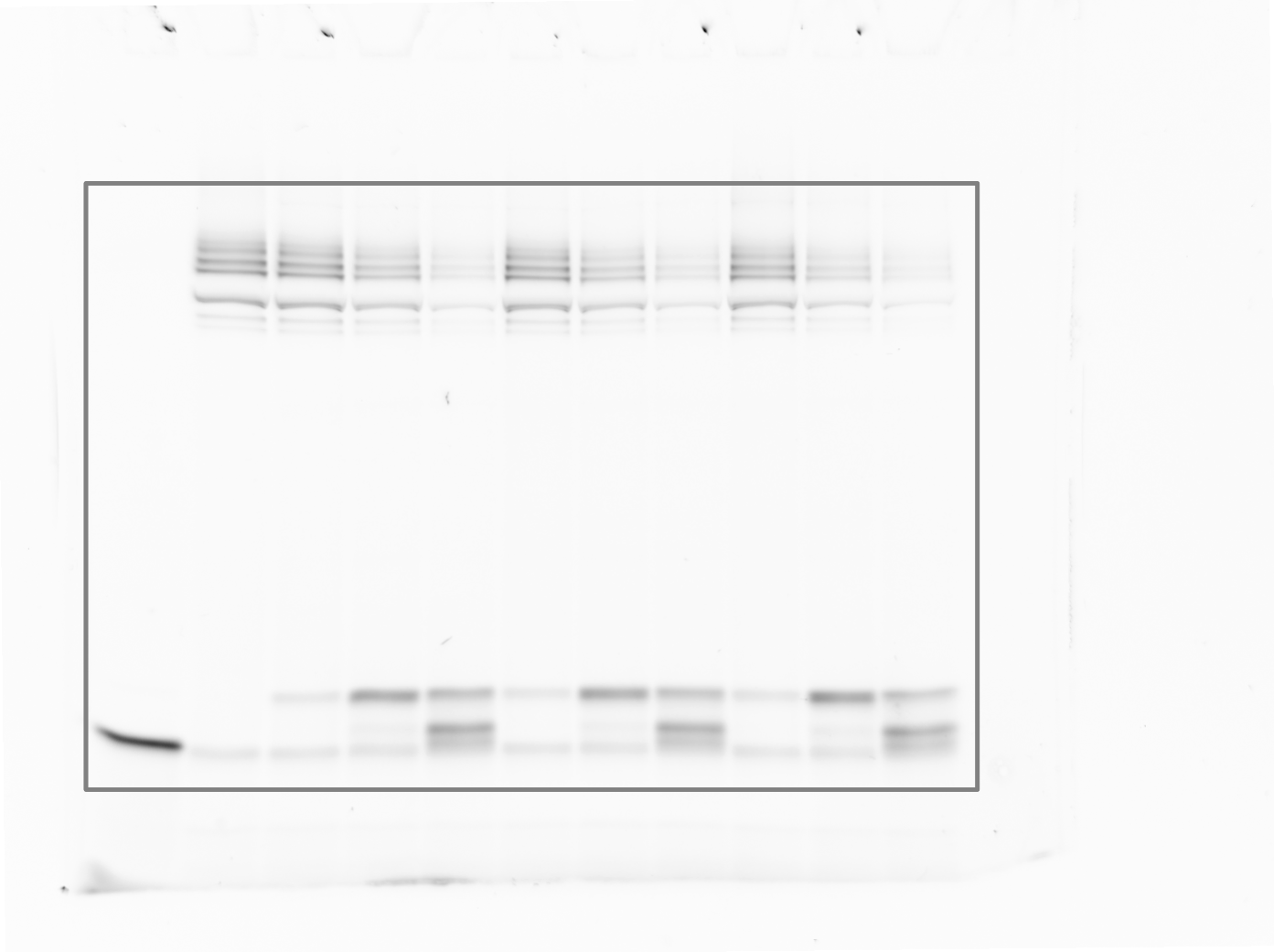

Supplement: Supplementary file 11 — Source Data [file 41467_2025_61224_MOESM11_ESM.zip › Source data/Uncropped scans of all blots and gels/Supplementary Fig. 6/Supplementary Fig. 6b/K63-WT_Cy5_label.tiff]

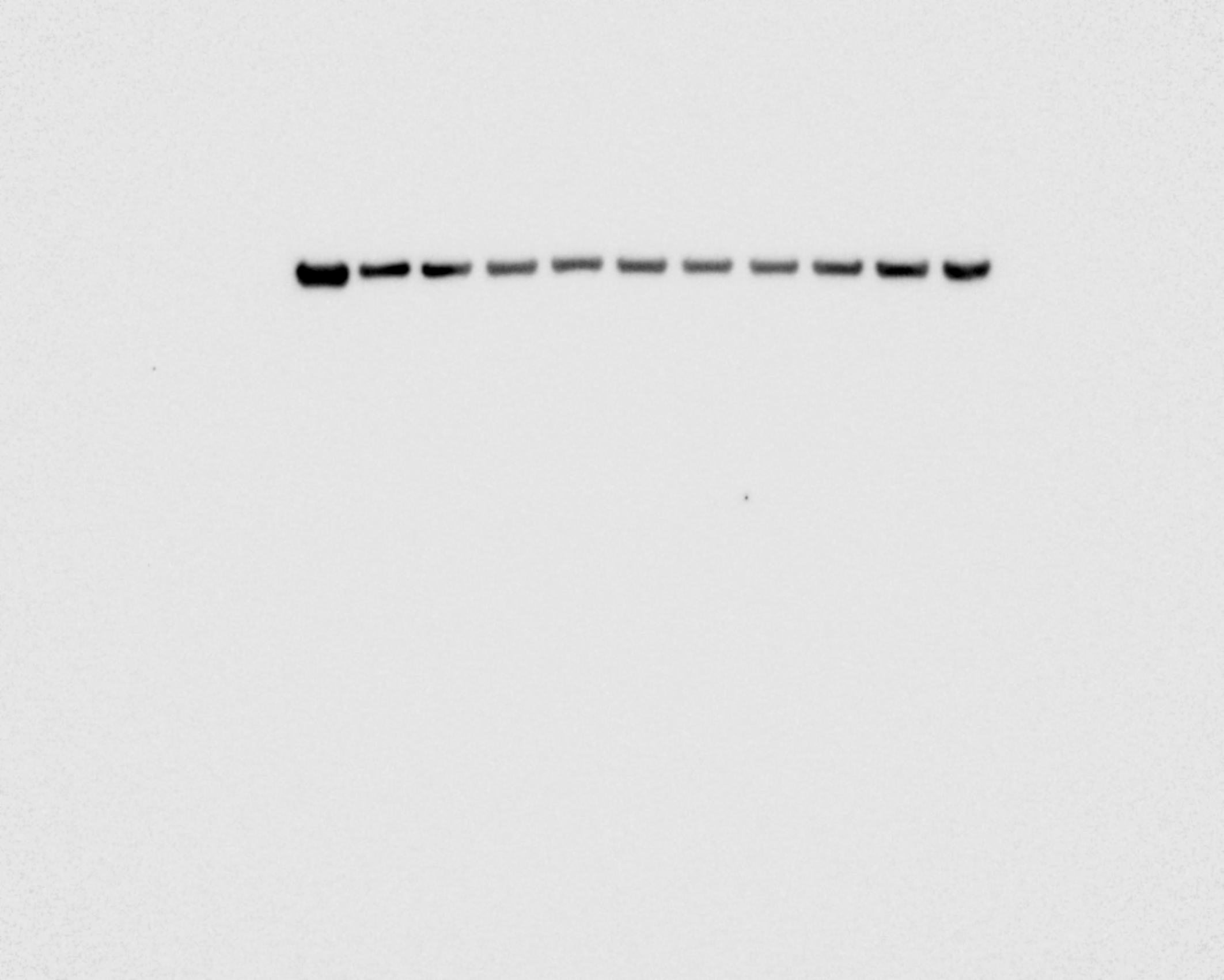

Supplement: Supplementary file 11 — Source Data [file 41467_2025_61224_MOESM11_ESM.zip › Source data/Uncropped scans of all blots and gels/Supplementary Fig. 7/Supplementary Fig. 7a/Actin/Actin.tif]

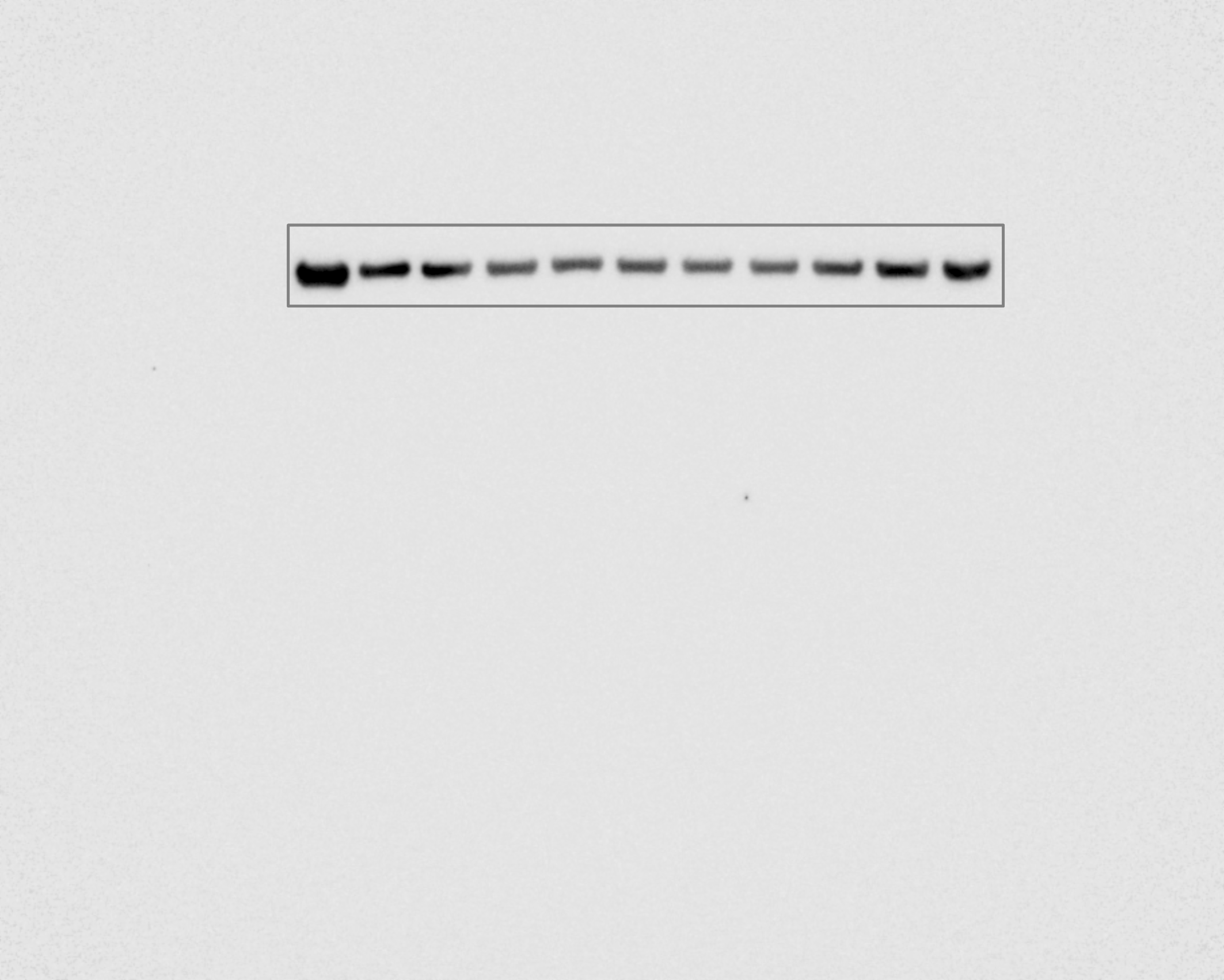

Supplement: Supplementary file 11 — Source Data [file 41467_2025_61224_MOESM11_ESM.zip › Source data/Uncropped scans of all blots and gels/Supplementary Fig. 7/Supplementary Fig. 7a/Actin/Actin_label.tiff]

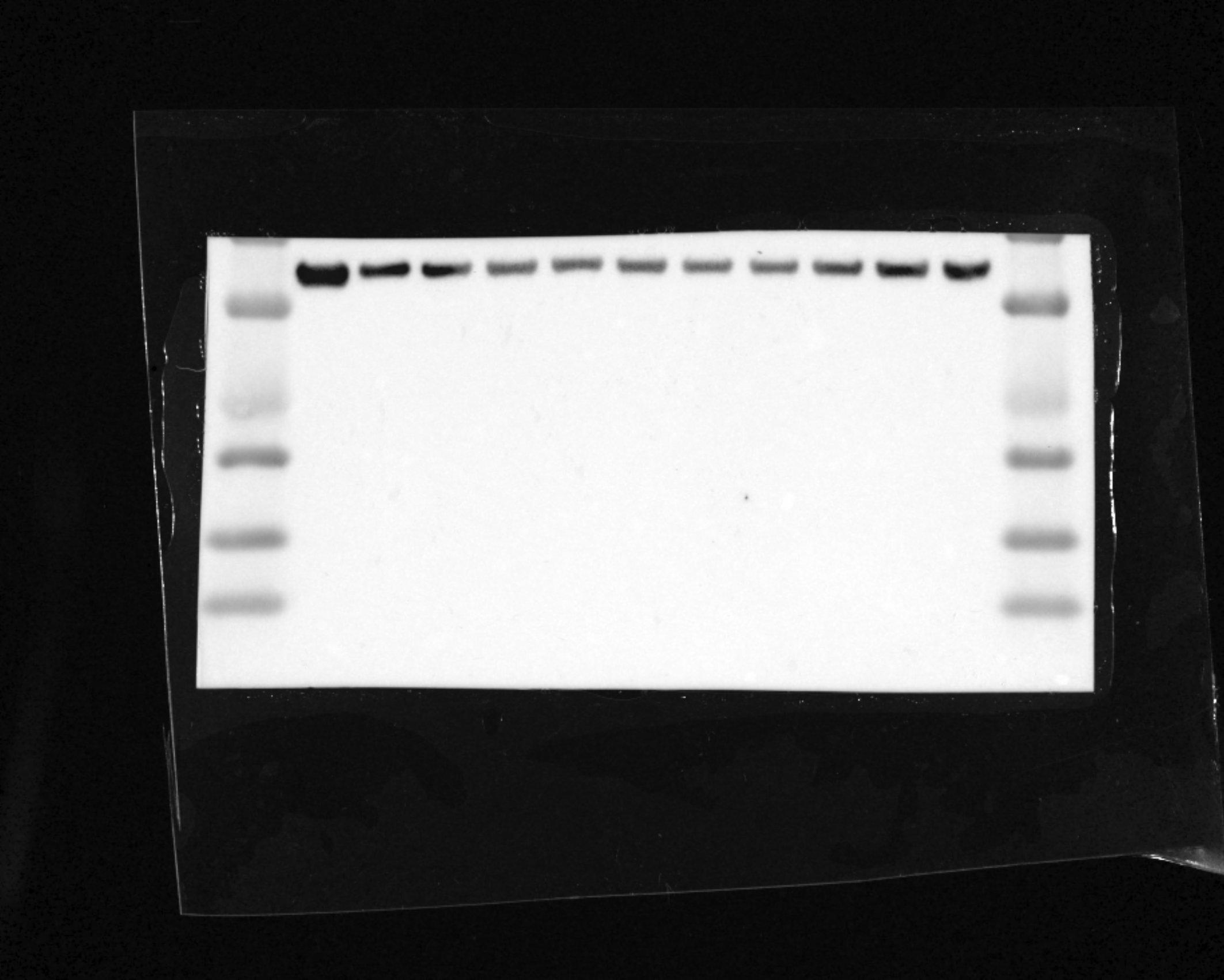

Supplement: Supplementary file 11 — Source Data [file 41467_2025_61224_MOESM11_ESM.zip › Source data/Uncropped scans of all blots and gels/Supplementary Fig. 7/Supplementary Fig. 7a/Actin/M+Actin.tif]

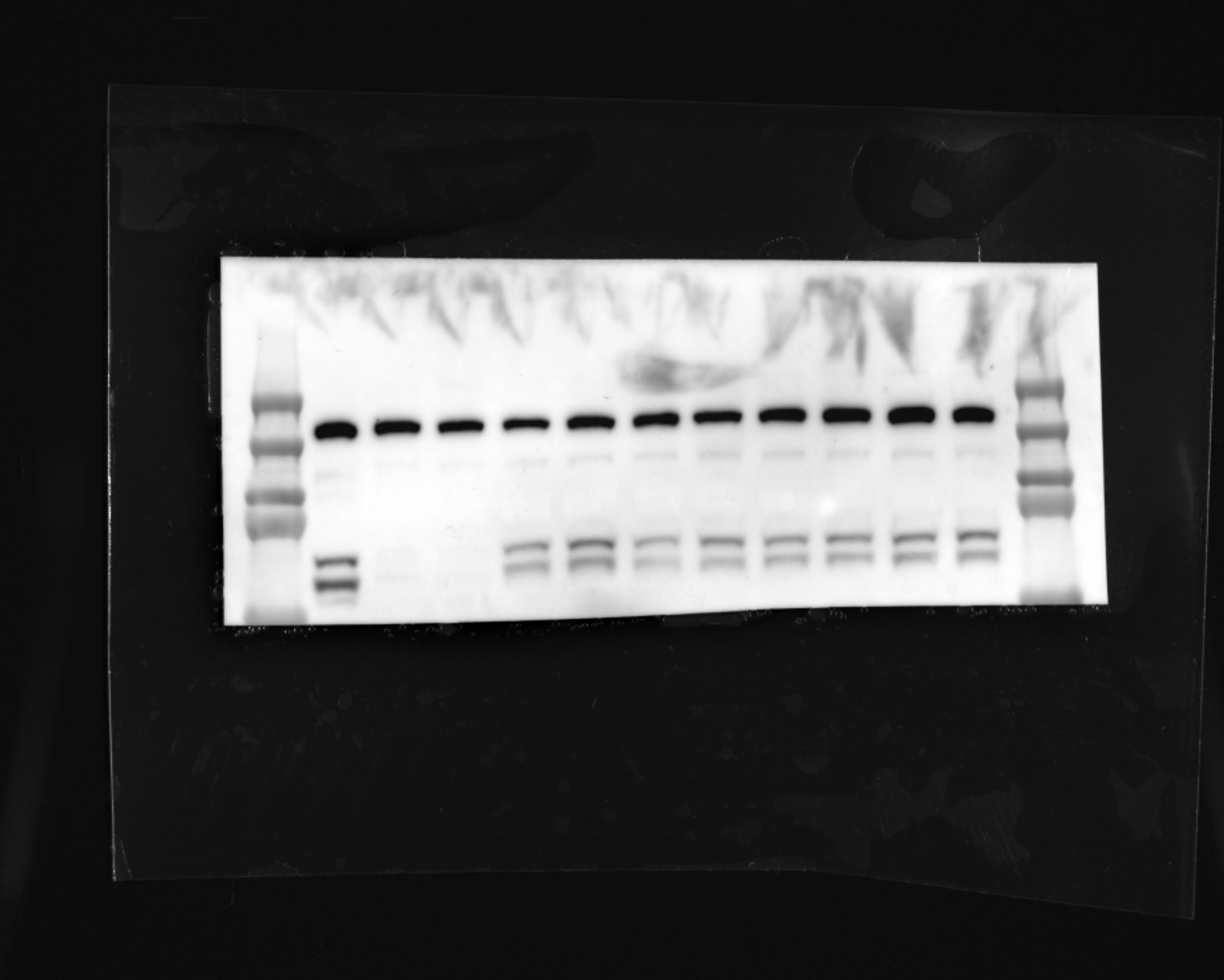

Supplement: Supplementary file 11 — Source Data [file 41467_2025_61224_MOESM11_ESM.zip › Source data/Uncropped scans of all blots and gels/Supplementary Fig. 7/Supplementary Fig. 7a/SPRTN/M+SPRTN.tif]

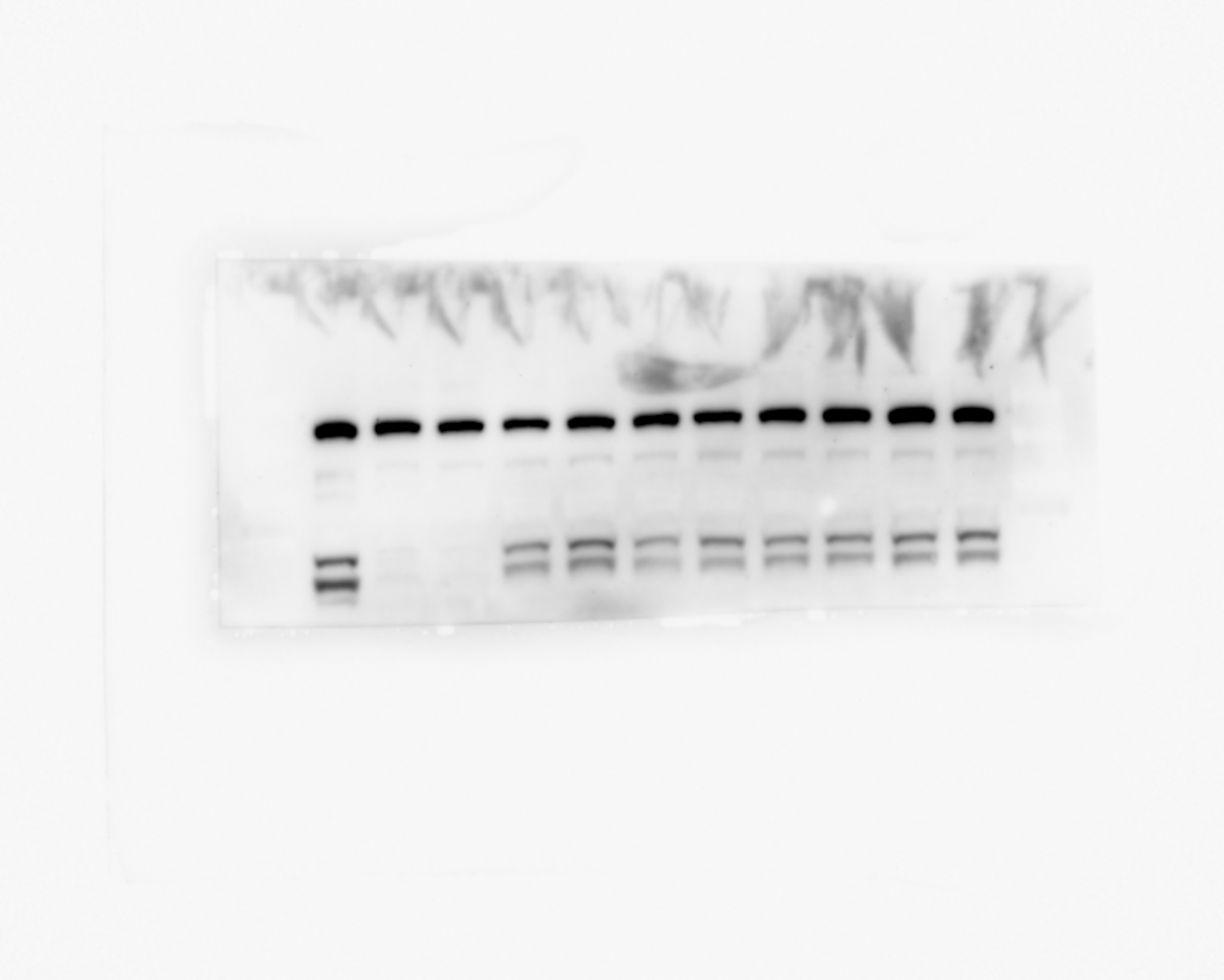

Supplement: Supplementary file 11 — Source Data [file 41467_2025_61224_MOESM11_ESM.zip › Source data/Uncropped scans of all blots and gels/Supplementary Fig. 7/Supplementary Fig. 7a/SPRTN/SPRTN.tif]

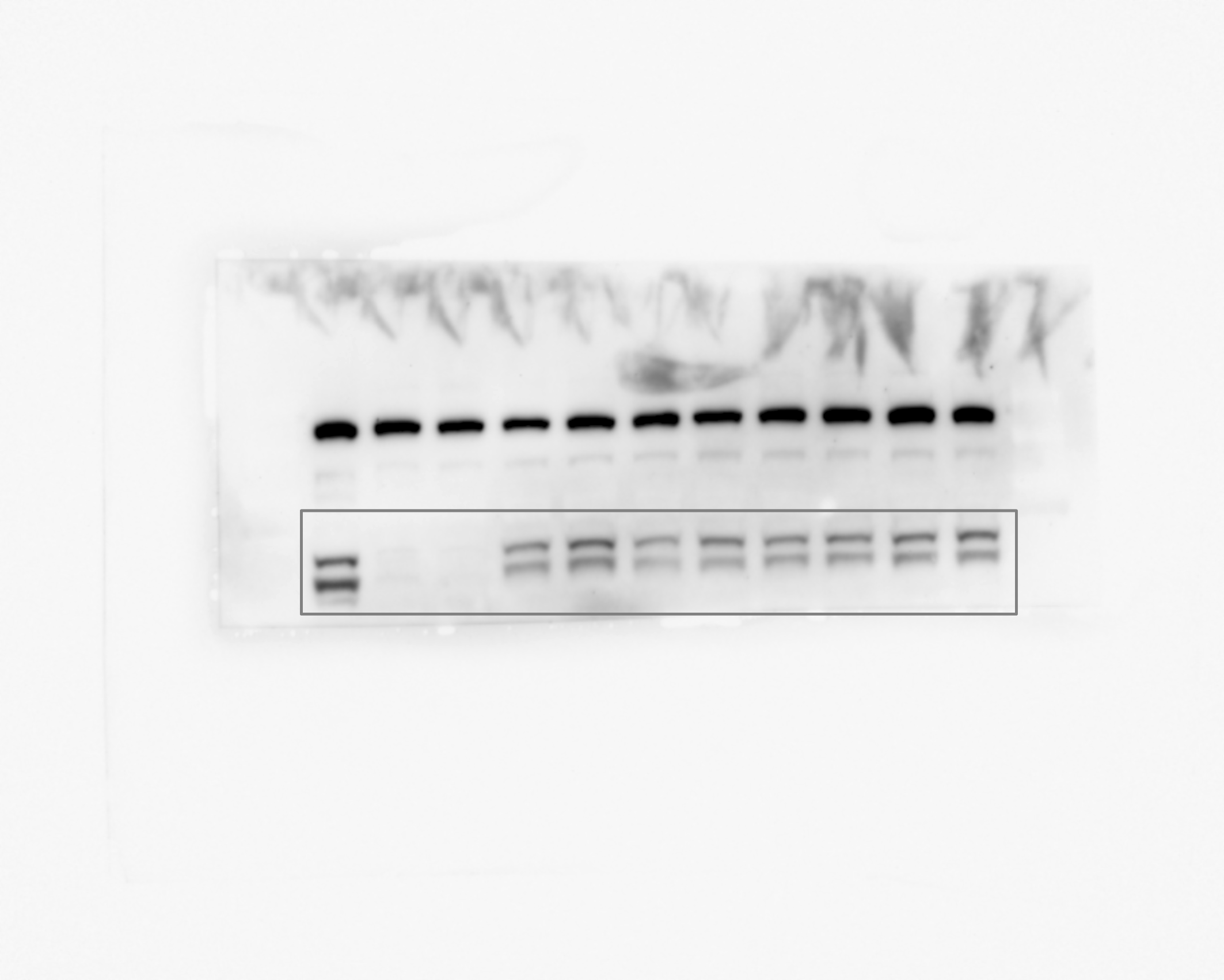

Supplement: Supplementary file 11 — Source Data [file 41467_2025_61224_MOESM11_ESM.zip › Source data/Uncropped scans of all blots and gels/Supplementary Fig. 7/Supplementary Fig. 7a/SPRTN/SPRTN_label.tiff]

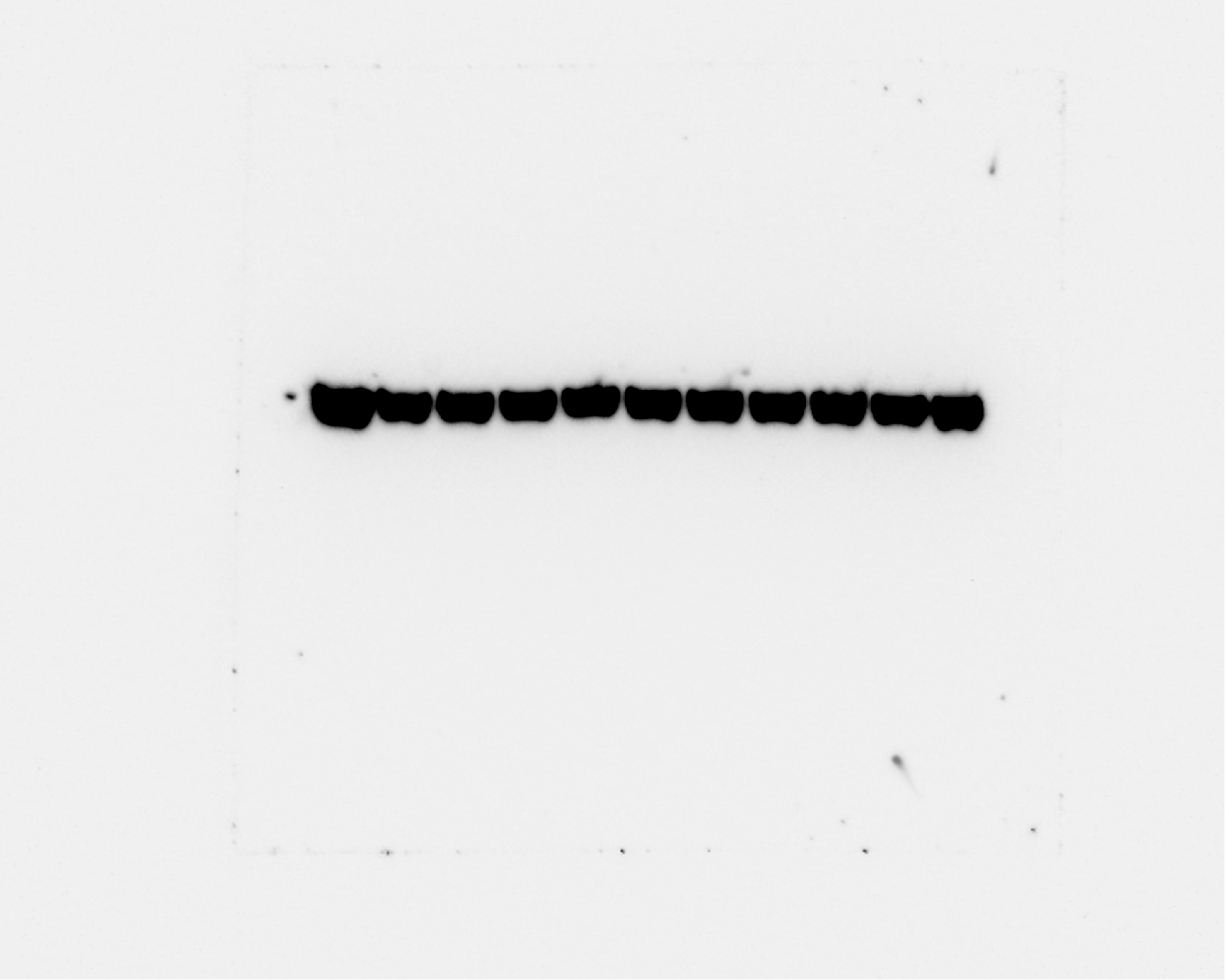

Supplement: Supplementary file 11 — Source Data [file 41467_2025_61224_MOESM11_ESM.zip › Source data/Uncropped scans of all blots and gels/Supplementary Fig. 7/Supplementary Fig. 7b/Actin/Actin.tif]

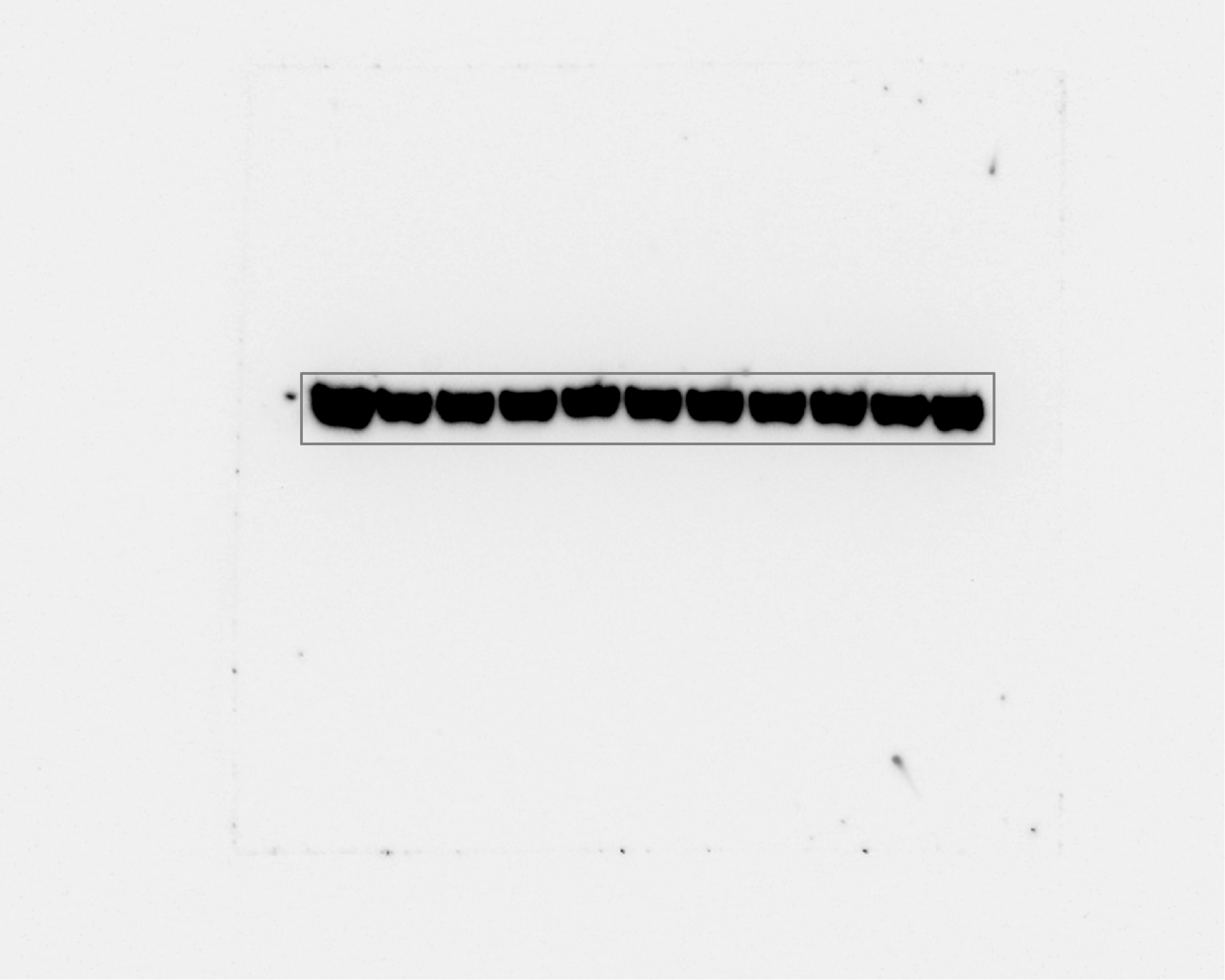

Supplement: Supplementary file 11 — Source Data [file 41467_2025_61224_MOESM11_ESM.zip › Source data/Uncropped scans of all blots and gels/Supplementary Fig. 7/Supplementary Fig. 7b/Actin/Actin_label.tiff]

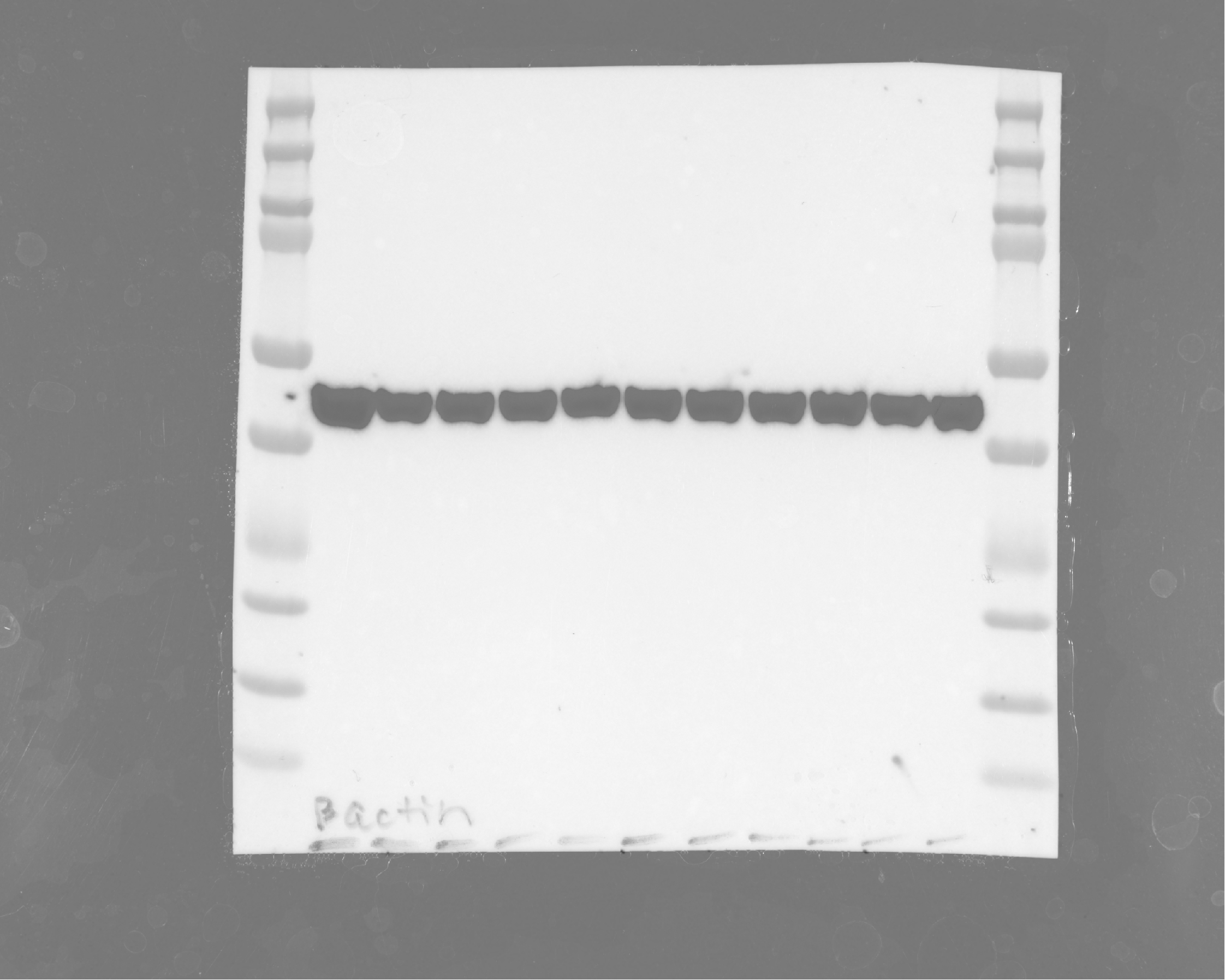

Supplement: Supplementary file 11 — Source Data [file 41467_2025_61224_MOESM11_ESM.zip › Source data/Uncropped scans of all blots and gels/Supplementary Fig. 7/Supplementary Fig. 7b/Actin/M+Actin.tif]

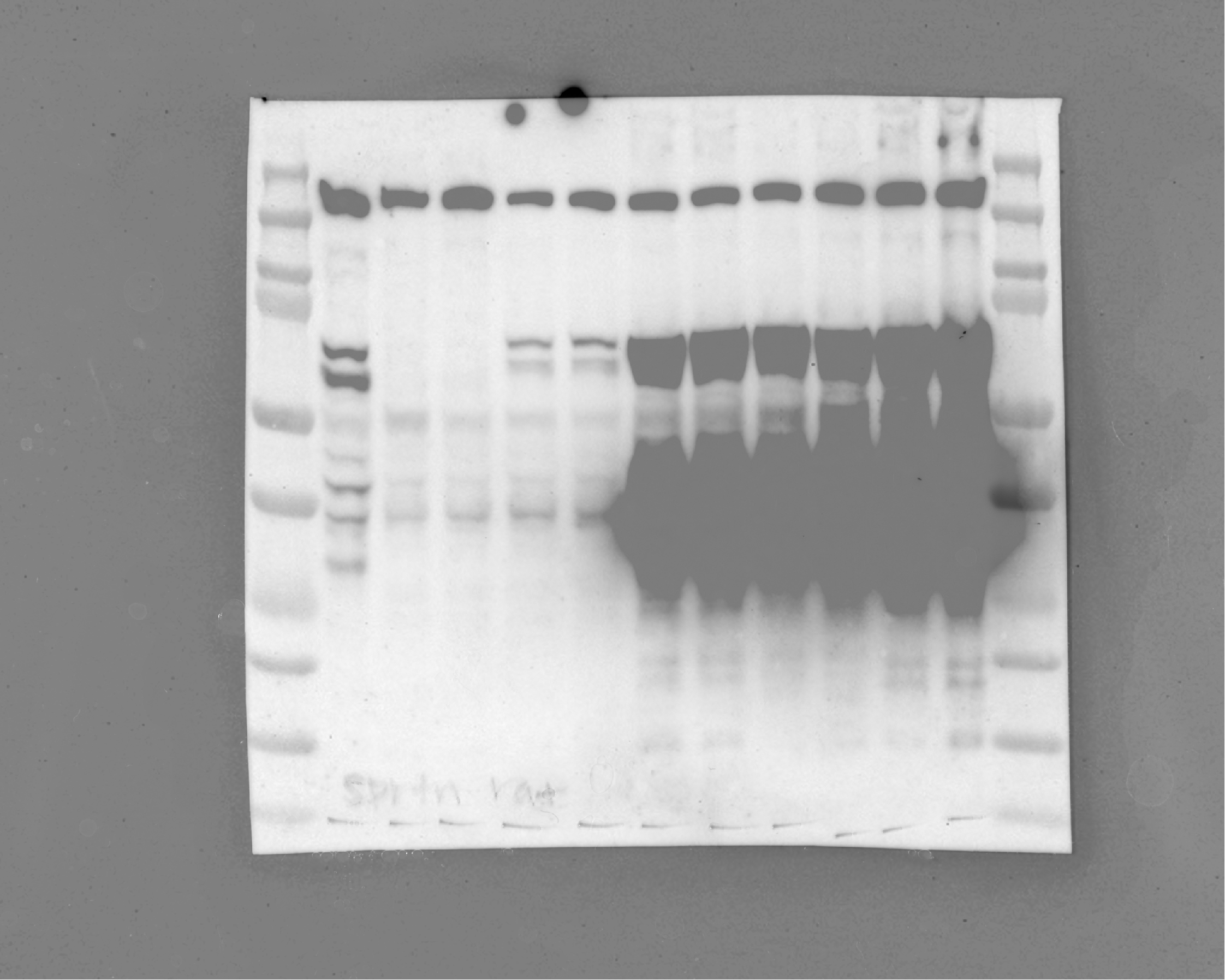

Supplement: Supplementary file 11 — Source Data [file 41467_2025_61224_MOESM11_ESM.zip › Source data/Uncropped scans of all blots and gels/Supplementary Fig. 7/Supplementary Fig. 7b/SPRTN/M+SPRTN_long.tif]

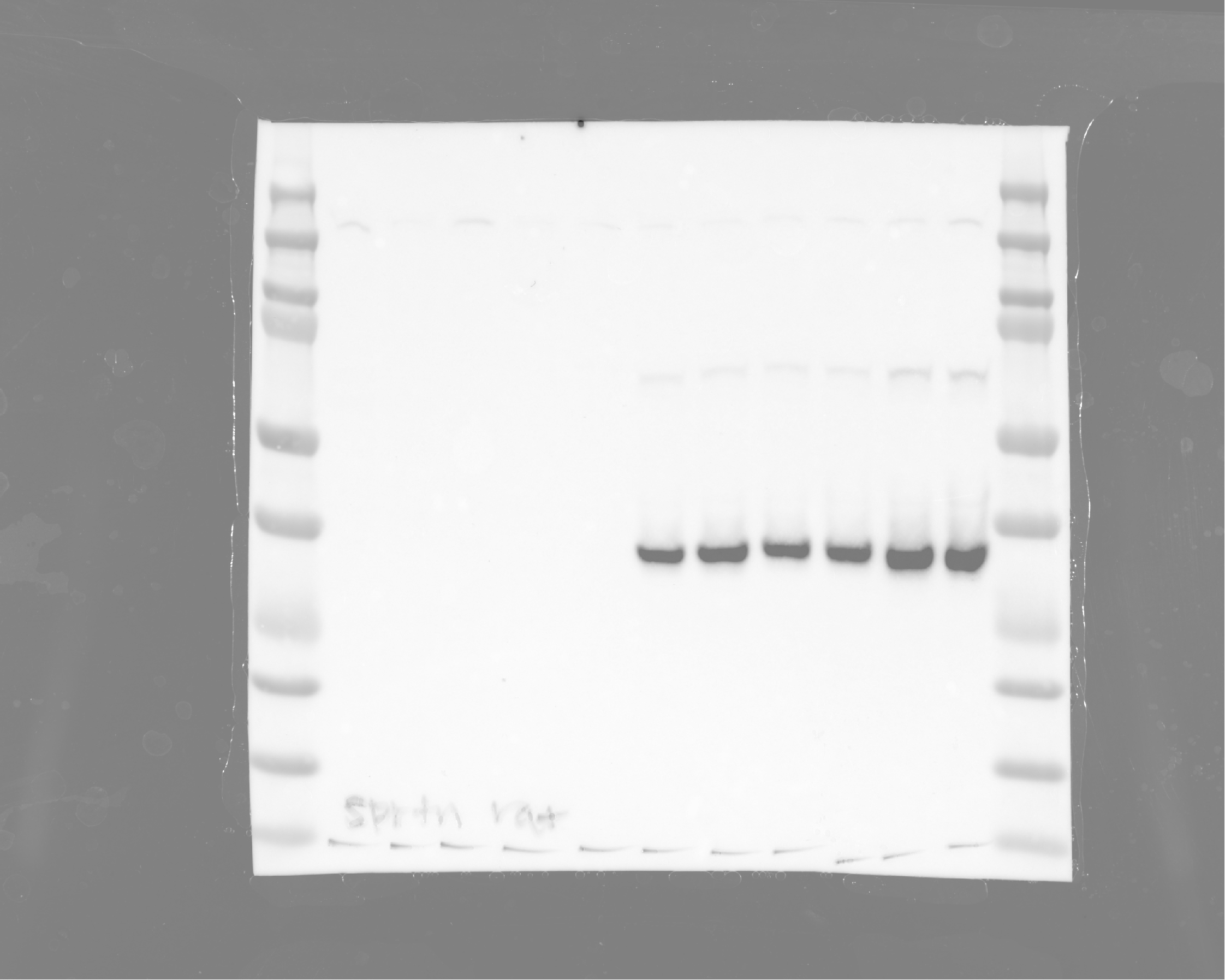

Supplement: Supplementary file 11 — Source Data [file 41467_2025_61224_MOESM11_ESM.zip › Source data/Uncropped scans of all blots and gels/Supplementary Fig. 7/Supplementary Fig. 7b/SPRTN/M+SPRTN_short.tif]
